# Genomic and transcriptional characterization of early esophageal squamous cell carcinoma

**DOI:** 10.1186/s12920-023-01588-7

**Published:** 2023-07-01

**Authors:** Jingjing Zhao, Xiya Jia, Qiaojuan Li, Hena Zhang, Jianjun Wang, Shenglin Huang, Zhixiang Hu, Caiping Li

**Affiliations:** 1 Department of Gastroenterology, Second Affiliated Hospital of Fujian Medical University, Quanzhou, China; 2 Department of Integrative Oncology, Fudan University Shanghai Cancer Center, and Shanghai Key Laboratory of Medical Epigenetics, Institutes of Biomedical Sciences, Fudan University, Shanghai, China; 3 Department of Pediatric Medicine, Gansu Provincial People’s Hospital, Lanzhou City, , Gansu Province China

**Keywords:** Early Esophageal squamous cell carcinoma, Whole-exome sequencing, Genetic variation, HOX family, Tumor immune microenvironment

## Abstract

**Background:**

Esophageal squamous cell carcinoma (ESCC) is a highly heterogeneous cancer that lacks comprehensive understanding and effective treatment. Although multi-omics study has revealed features and underlying drivers of advanced ESCC, research on molecular characteristics of the early stage ESCC is quite limited.

**Materials and methods:**

We presented characteristics of genomics and transcriptomics in 10 matched pairs of tumor and normal tissues of early ESCC patients in the China region.

**Results:**

We identified the specific patterns of cancer gene mutations and copy number variations. We also found a dramatic change in the transcriptome, with more than 4,000 genes upregulated in cancer. Among them, more than one-third of HOX family genes were specifically and highly expressed in early ESCC samples of China and validated by RT-qPCR. Gene regulation network analysis indicated that alteration of Hox family genes promoted the proliferation and metabolism remodeling of early ESCC.

**Conclusions:**

We characterized the genomic and transcriptomic landscape of 10 paired normal adjacent and early ESCC tissues in the China region, and provided a new perspective to understand the development of ESCC and insight into potential prevention and diagnostic targets for the management of early ESCC in China.

**Supplementary Information:**

The online version contains supplementary material available at 10.1186/s12920-023-01588-7.

## Background

Esophageal cancer (EC), one of the most lethal cancers, is the seventh common cancer type and the sixth leading cause of cancer-related death in the world [1]. In China, there will be approximately 346,600 people newly diagnosed and 323,600 people dying from EC in 2022 [2]. Esophageal squamous cell carcinoma (ESCC) and esophageal adenocarcinoma (EAC) are the two main subtypes of EC, and have almost completely distinct geographic patterns, and primary risk factors. ESCC accounts for the vast majority of EC cases worldwide, especially in Asia [3]. The risk factors of ESCC are complicated in different countries and regions and are thought to be possibly related to dietary habits (like hot foods, tobacco, alcoholic beverages, and betel quid) and exposure to carcinogens. On the contrary, EAC is the main subtype of EC in western countries, which develops from esophageal intestinal metaplasia caused by chronic gastroesophageal reflux disease [4]. Therefore, etiological factors may result in different genetic alterations and related therapeutic targets, especially in Chinese patients compared with patients from western countries.

The gradual progression from normal squamous epithelium to ESCC can be tracked by histological analysis, from basal cell hyperplasia (BCH), to mild dysplasia, moderate dysplasia, severe dysplasia, carcinoma in situ (CIS), and invasive carcinoma [5, 6]. Over the past decade, encouraging studies have shed light on the molecular basis of ESCC pathogenesis. As technology advances, new works have mapped the genetic and epigenomic characteristics of ESCC, including frequent high-level amplifications and homozygous deletions involving 11q13.2–q13.3 (locus of *CCND1*) and 9p21.3 (locus of *CDKN2A* and *CDKN2B*) [7]. Exome sequencing revealed prevalent mutations in the coding regions of *TP53* of patients with ESCC and, along with low-frequency mutations in some other genes (e.g., *NOTCH1*, *NFE2L2*, *KMT2D*, *CDKN2A*) [8], suggesting the involvement of additional risk factors like transcriptome remodeling in the development of ESCC. Meanwhile, ESCC is usually asymptomatic in its early stages, and is known for rapid progression and poor outcomes [9]. Therefore, the disease is usually at an advanced stage at the time of diagnosis. Genomic and transcriptional changes at an advanced stage of ESCC have been characterized in several studies, however, these changes were poorly understood in early-stage ESCC. Early stage (stage I or II) patients, for example, with various mutational loads did not predict a different prognosis [10]. Considering the difference of geographic patterns and primary risk factors of the disease, it is of great significance for the diagnosis and treatment of ESCC patients, especially in Chinese patients, to deeply understand the molecular etiology of early ESCC and explore the key driver genes.

In the current study, we aimed to systematically define genomic and transcriptomic alterations in Chinese patients with early stage of ESCC. We performed whole-exome sequencing (WES) and RNA sequencing of 10 matched pairs of tumor and normal tissues from patients with early ESCC. We next identified the somatic mutations and copy number variations in early ESCC with different genetic patterns compared to other countries. Then, focusing on specific up-regulation genes of early ESCC, we found most of Hox family genes were upregulated, which may play a key role in regulating cell cycle and promoting ESCC development. Finally, we explored the complex changes in the immune microenvironment.

## Materials and methods

### Sample collection and isolation

The tumor and adjacent normal tissues from 10 ESCC patients were obtained from biopsies from Second Affiliated Hospital of Fujian Medical University. Tumor tissues of EC patients were pathologically confirmed ESCC with early stage (stage I), and all samples were stored in RNAlater at -4 °C overnight and then transferred into -80 °C. Informed consent was obtained from all patients, and the study was approved by the Ethics Committee of Second Affiliated Hospital of Fujian Medical University (2020–246, 2020–05-22). The clinical data was shown in Additional file4:Table S3.

### Whole-exome sequencing

The whole-exome sequencing (WES) was performed on ESCC patients using DNA from both tumor samples and matched noncancerous tissues. Genomic DNA (200 ng) from frozen tissues was extracted using AllPrep DNA/RNA Kit (Qiagen) and sheared by Biorupter (Diagenode, Belgium) to acquire 200–300 bp fragments. The ends of DNA fragments were repaired and Illumina Adaptor was added (Fast Library Prep Kit, iGeneTech, Beijing, China). After the sequencing library was constructed, whole exomes were captured using the AIExomeV2 (T192V1T) Enrichment Kit (iGeneTech, Beijing, China) and sequenced on DNBSEQ-T7 platform (MGI, Shenzhen, China) with 150 bp paired-end reads.

### RNA sequencing (RNA-seq)

Total RNAs (500 ng) from ESCC tissues and matched normal tissues were extracted by Trizol® Reagent (Thermo, USA) and treated with DNase I (NEB) to remove DNA before constructing the RNA-seq libraries. Strand-specific RNA-seq libraries were prepared using the QIAseq FastSelect–rRNA HMR (Qiagen) and KAPA RNA HyperPrep (Roche) kits. Briefly, QIAseq FastSelect reagent was added to the RNA sample to fragment the RNA at 85 °C for 6 min and then stepwise cool the reaction from 75 °C to 25 °C for 14 min. First- and second-strand cDNA was synthesized with random hexamer primers, treated with DNA End Repair Kit (Qiagen) to repair the ends, then modified with Klenow to add an A at the 3’ end of the DNA fragments, and finally ligated to adapters. Purified dsDNA was further subjected to 11 cycles of PCR amplification. The libraries were quality controlled with Qubit (Thermo Fisher Scientific, USA) and Qsep100 (BiOptic, China) and sequenced by the Illumina sequencing platform (Nova) on a 150 bp paired-end run.

### Identification and annotation of somatic mutation

Raw reads were first filtered and trimmed adapters using fastp (v0.12.2). After quality control, reads were mapped to the human reference genome sequence (hg 19) by BWA-MEM (version 0.7.12) [11] followed by pre-processing steps, including duplicate making, indel realignment, and base recalibration using the Genome Analysis Toolkit (GATK version 4.1.9.0), generating analysis-ready BAM files. Next, GATK MuTect2 was performed to call somatic mutations and tumor-only mutations with frequency higher than 5% were retained for downstream. ANNOVAR (version 201,910) was used to annotate variants and annovarToMaf converted annovar annotations into MAF for visualization. Cancer genes were downloaded from NCG6.0 database (http://ncg.kcl.ac.uk/) [12].

### Tumor mutation burden analysis

Only Missense_Mutation was included and capture size was performed as 40 for estimation TMB.

### Data From TCGA

We obtained an extensive collection of genomic and transcriptomic data from patients diagnosed with esophageal cancer from the TCGA-ESCA project (https://gdc.xenahubs.net) and incorporated them into the TCGA-EC cohort. Subgroup of patients who met our stringent criteria for TCGA-ESCC-early cohort (i.e., those diagnosed with ESCC and with stage 1 or II) was defined. The result of mutect2 was used for the mutation of TCGA cohort and its annotations are derived from. The TMB of TCGA-ESCC-early cohort and TCGA-EC cohort was calculated by same method of Early ESCC cohort. To compare HOX family gene expression, we downloaded the FPKM from the TCGA-ESCC-early cohort and normal samples from https://gdc.xenahubs.net. We calculated the fold change (FC = mean(TCGA ESCC early)/mean(TCGA-ESCA normal)) for HOX gene expression..

### Tumor signature analysis

Tumor mutation signature was determined by NMF of maftools (version 2.6.5) [13] R package. Best possible value was 3 as the result of estimateSignatures. ‘legacy’ as database was to be compared with tumor mutation signature.

### Copy number variation analysis

Pre-processing, including duplicates and low base quality was the same as mutation workflow. CNVkit (version 0.9.8) software [14] was applied with the default strategy to obtain CNVs in each sample. To identify significant amplification and deletion focal regions, GISTIC2 (version 2.0.23) [15] was performed with confidence level 0.9, and significant focal regions were visualized by gisticChromPlot function of maftools.

We obtained gene copy number variation frequency from all_thresholded.by_genes, a result file of GISTIC2. Negative indicates deletion and positive indicates amplification.

### Transcriptome analysis

The raw data after quality control was aligned to human reference genome (GRCh38) by STAR (version 2.3.5a) [16], and gene expression was qualified by featureCounts (version 1.6.2) [17] with Genecode(version 29). Gene with rowSum of FPKM larger than 2 was retained for downstream analysis.

### Enrichment analysis

KEGG [18] and Gene ontology enrichment analysis was performed by DAVID [19], and top significant terms were displayed. Gene set enrichment analysis (GSEA) was carried out with MSigDB 6.2 background geneset.

### Regulation network analysis

Gene regulation network (GRN) of HOXC10 in tumor was predicted by GENIE3 (version 1.4.3) [20], which R packages for inferring GRN based on gene expression. GENIE3 object was created by default and threshold of weights was 0.1. Cytoscape was used to visualize top 30 target genes of HOXC10.

### Immune microenvironment enrichment analyses

xCell (version 1.1.0) [21] with default setting was used to evaluate cell type enrichment in each sample. Score was scaled across row between -1 and 1. twofold absolutely score was considered as significant difference cell type between tumor and adjacent normal tissues.

### RT-qPCR (Reverse Transcription Quantitative PCR) analysis

Total RNA was extracted using TRIzol (Life Technologies, Carlsbad, CA, USA), and the reverse transcription of complementary DNA was obtained by PrimeScript RT Reagent Kit (Takara Bio Inc., Dalian, China). Subsequently, the polymerase chain reaction (PCR) was performed using SYBR® Green Premix Pro Taq HS qPCR Kit (Accurate Biology, Hunan, China) on the Applied Biosystems QuantStudio 5 (Applied Biosystems, Foster City, CA, USA). The expression was calculated by the 2-ΔCt method and normalized by β-actin. The PCR primers used were shown in Additional file 4: Table S5.

### Statistical analyses

Student’s *t*-test was used to identify differentially expressed genes (DEGs) between tumor and normal tissues for RNA-seq. Genes with *P* value < 0.01 and twofold fold change were considered significant. The relative expression levels of HOX family gene obtained from RT-qPCR were analyzed using Prism 8.0.1 software with Student's paired two-tailed t-test. A *p*-value of < 0.05 was considered statistically significant.

## Results

### Mutation profiles in early ESCC

**Fig. 1:**
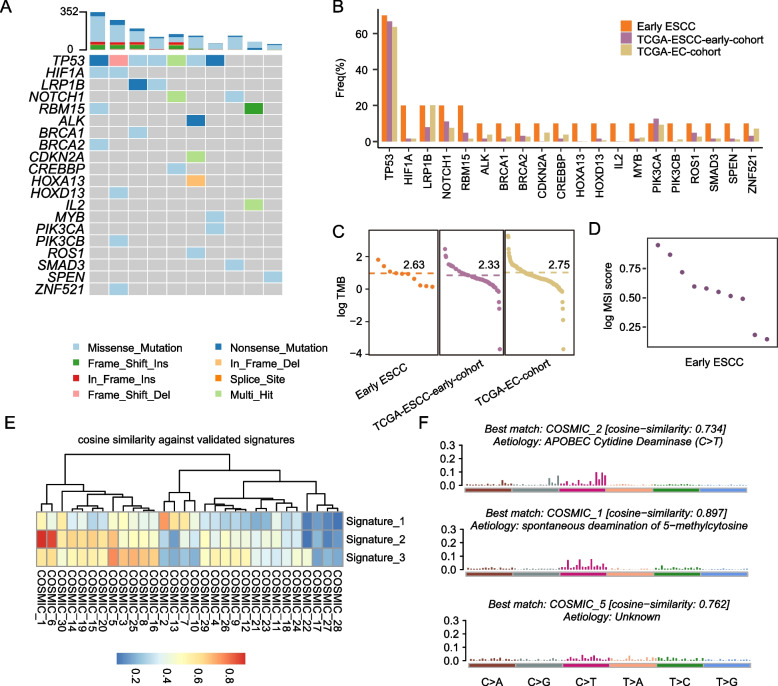
Mutation profiles in early ESCC. **A** Genetic alterations of cancer-related gene in early ESCA. **B** The gene mutation frequency in Early ESCC (*n* = 10), TCGA-ESCC-early-cohort(*n* = 63), and TCGA-EC cohort(*n* = 184). **C** The TMB in Early ESCC and TCGA-ESCC-early-cohort, and TCGA-EC cohort. **D** MSI score in early ESCC, each point means a sample. **E** Heatmap showing the similarity of mutational signatures against COSMIC signatures calculated by maftools. **F** The best match across COSMIC database of three mutational signatures

We obtained 10 paired tumor and adjacent normal tissues from early ESCC patients to subject WGS and RNA-seq. In total, 2,179 variations were detected after qualify control, including 386 short insert and deletion, 1,050 mismatch mutations (Additional file 2: Table S2). In order to investigate the characteristics of cancer gene in early ESCC, we identified genes alteration classification and frequency. Top 20 cancer-related genes were visualized by heatmap, including *TP53*, *HIF1A*, *NOTCH1*, *HOXA13* and *HOXD13* (Fig. 1A). Further, we found that the cancer-related genes with high-frequency mutations in Chinese early ESCC patients had a lower mutation frequency in TCGA samples, irrespective of whether they belonged to the early ESCC or EC cohorts (Fig. 1B). Additionally, we observed that the mean tumor mutational burden (TMB) for early ESCC patients was 2.63, while it was 2.33 and 2.75 for TCGA ESCC early cohort and TCGA EC cohort, respectively (Fig. 1C). Moreover, the mean of microsatellite instability (MSI) score was 0.975 (Fig. 1D), also much lower [22].

Next, mutation signatures were also identified by maftools package. Three significant signatures were defined and had similarities with COSMIC database signature (Fig. 1E). Signature1 was best matched with COSMIC_2 and characterized as ‘APOBEC Cytidine Deaminase’ (Fig. 1f), which is widespread in human cancer [23]. For signature2, associated with ‘spontaneous deamination of 5 − methylcytosine’ (Fig. 1F), appears to cause human inherited disease [24]. Signature3 was best matched with COSMIC_5 with higher C > T mutation and unknown function (Fig. 1F).

### Copy number variation in early ESCC

**Fig. 2:**
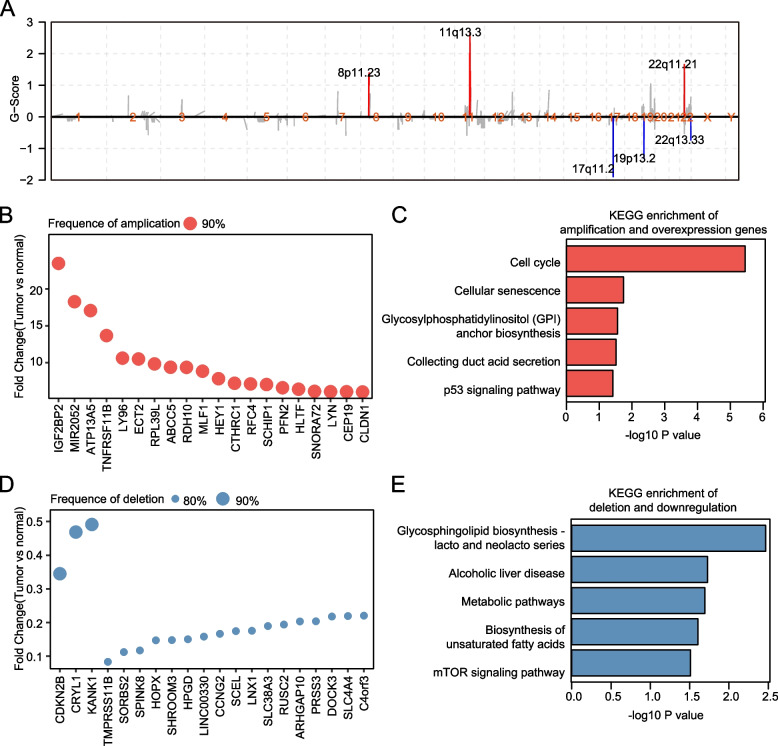
Copy number variation in early ESCC. **A** Significance of copy number variation detected by GISTIC, including amplification(red) and deletion peaks (blue). **B** Top 20 genes with the highest changes in amplification frequency and expression. **C** KEGG[18] enrichment of top 500 genes with the highest changes in amplification frequency and expression. **D** Top 20 genes with the highest changes in deletion frequency and expression. **E** KEGG[18] enrichment of top 500 genes with the highest changes in deletion frequency and expression

To observe the changes of copy number in early ESCC, we next obtained somatic CNVs in 10 tumor tissues after fitter paired adjacent normal tissues. The significant focal CNVs were identified by GISTIC2, including three amplifications (8p11.23, 11q13.3, 22q11.21) and three deletions (17q11.2, 19p13.2, 22q13.33; Fig. 2A). 8p11.23 commonly amplified in ESCC [25], and genes of this locus, such as *BAG4* (fold change = 2.91, *P* value = 0.02), *ZNF703* (fold change = 4.22, *P* value = 0.01) and *F4EBP1* (fold change = 2.36, *P* value = 0.03), were identified as up-regulated genes in ESCC at mRNA level. In addition, we explored the potential effect of 8p11.23 on the transcriptome. 8p11.23-amplification ESCC samples were enriched in G2M and TNFA signaling geneset, and related genes were also highly expressed in tumors (Additional file 1: Fig. 1). This finding implied that genes involved in proliferation and the immune system were affected by the amplification of 8p11.23. In order to further investigate which genes were affected by copy number amplification, we identified genes in the tumor that were both overexpressed and amplified. Top 20 genes with the highest amplification frequencies and fold change were shown, including *TNFRSF11B*, which played an important role in signaling receptor activity and cytokine activity (Fig. 2B). The result of KEGG enrichment analysis suggested that cell cycle related genes were amplified in ESCC, which may influence tumor progression (Fig. 2C). In contrast, genes with the highest deletion frequencies and fold change were shown in Fig. 2D. *CDKN2A* has been reported to have copy number deletion in ESCC [26]. However, in early ESCC, *CDKN2B*, which lied adjacent to *CDKN2A*, was detected in copy number deletion. Glycosphingolipid biosynthesis and metabolic pathway enriched by top 500 genes with highest changes in deletion frequency and expression using KEGG pathway analysis indicated dysregulation of metabolic pathway in ESCC (Fig. 2E).

### Dramatic changes of Hox family gene expression in early ESCC

**Fig. 3:**
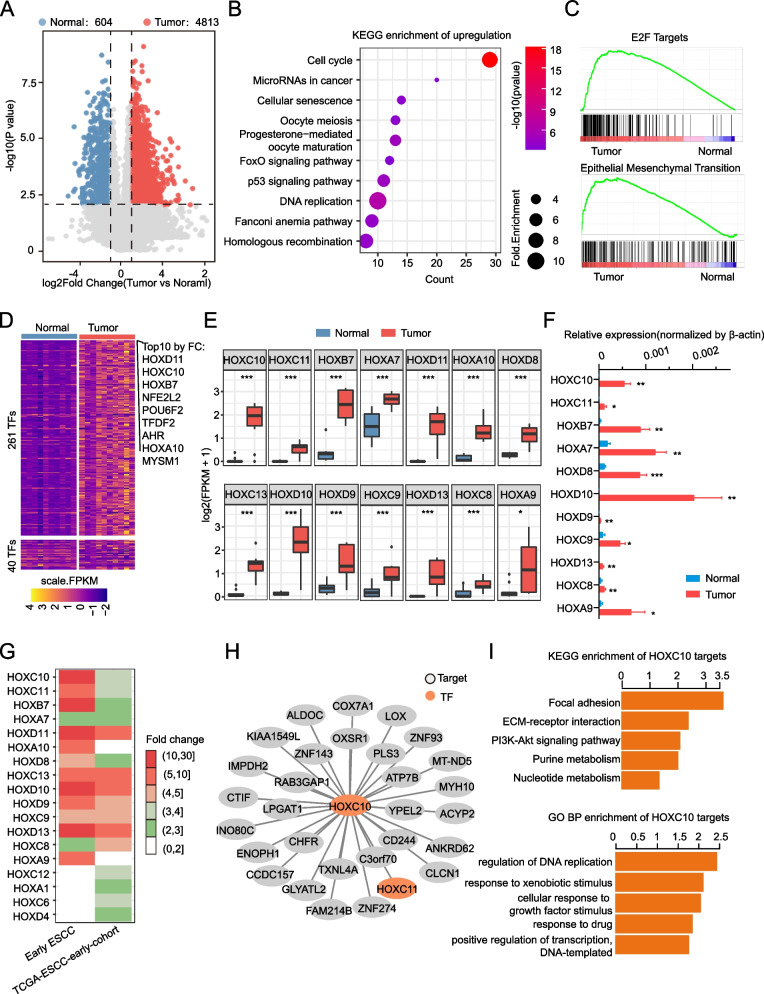
Dramatic changes of Hox family gene expression in early ESCC. **A** Volcano plot showing the DEGs between tumor and normal tissue in 10 pairs of early ESCC. **B** KEGG enrichment of top 500 upregulation in tumor tissue. **C** GSEA enrichment of E2F target and EMT geneset in tumor. **D** Heatmap showing the differentially expressed transcription factors; TF list was downloaded from AnimalTFDB database. **E** Boxplot showing the differentially expressed Hox family between tumor and normal tissue, T-test, *p < 0.05, **p < 0.01, ***p < 0.001. **F** The relative expression of HOX family genes was detected by RT-qPCR. * *p* < 0.05, ** *p* < 0.01. *** *p* < 0.001, average ± SEM, paired t test. **G** Heatmap showing the expression fold change (tumor vs normal) of Hox family in (**H**) in early ESCC and TCGA-ESCC-early cohort. **I** Regulated network of Hoxc10 predicted by GENIE3. **H** Barplot showing KEGG[18] and GO BP enrichment of top 500 predicted Hoxc10 target

In order to explore the difference in transcriptomics between tumor and adjacent normal tissues of early ESCC, we compared the gene expression after qualify control and identified 4,813 upregulated and 604 downregulated genes (Fig. 3A) (Additional file 3: Table S2). The KEGG enrichment of upregulated genes suggested that cell cycle and DNA replication were activated in tumor, matching with the results of amplification enrichment (Fig. 3B). Moreover, E2F and epithelial mesenchymal transition (EMT) genesets were also enriched in tumor by GSEA (Fig. 3C). The expression profile of transcriptional factors (TFs) was compared to reveal differences in transcriptional regulation between tumor and adjacent normal tissues. The heatmap showed differentially expressed TFs, including 261 upregulated and 40 downregulated TFs (Fig. 3D). Interestingly, the top 10 upregulated TFs contained three Hox family genes. Furthermore, we also found that 14/39 Hox family genes were significantly upregulated in tumor (Fig. 3E). To validate our results, we conducted RT-qPCR analysis, and found that the majority of them (11/14) showed consistent upregulation in the tumor tissue (as shown in Fig. 3F). Most Hox genes are associated with tumor development. For example, the upregulated *HOXC10* promotes ESCC proliferation both in vitro and in vivo [27], *HOXD13* has been reported to promote the malignant progression of colon cancer [28]. Interestingly, *HOXD13* was also identified with the mismatch mutation at exon 1. Compared with TCGA-ESCC-early cohort (Additional file 4: Table S4), we found that the fold changes (FC) of most HOX family genes were significantly higher in Chinese patients than in the TCGA-ESCC-early cohort. (Fig. 3G). These results suggested an important regulatory role of the Hox family in early ESCC progression, specifically, in Chinese patients. To further explore the genes regulated by Hox and their biological characteristics, we predicted the *HOXC10* target gene since it was the most significant difference in RNA-seq (*P* value = 2.61E-05) and constructed its regulatory network (Fig. 3H). The network showed top 30 target genes. Among them, *LOX* played a critical role in tumor immune microenvironment [29] and influenced tumor cell proliferation [30], was also upregulated in early ESCC (fold change = 6.39, *P* value = 0.01). The results of KEGG and Biological Process (BP) of Gene Ontology (GO) enrichment showed that nucleic acid metabolism and cell cycle related genes were regulated by *HOXC10* targets (Fig. 3I). In summary, this data suggested HOX family genes were higher expression in tumor tissue and may play an important role in Chinese early ESCC, which may regulate tumor development.

### Immune microenvironment analysis

**Fig. 4:**
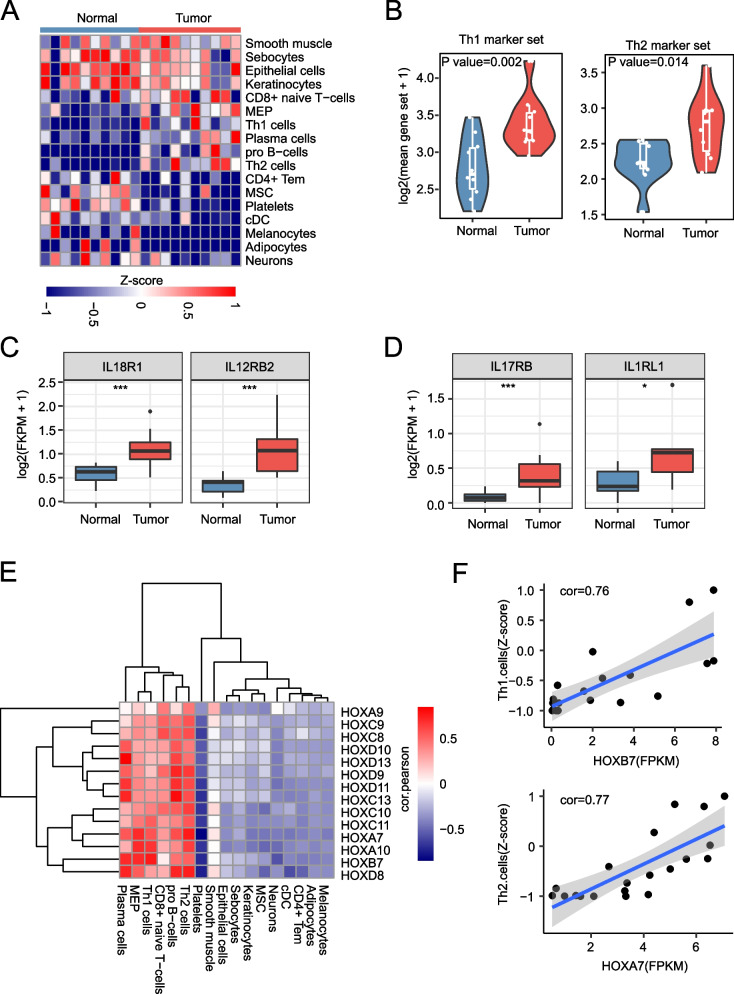
Immune microenvironment analysis. **A** Heatmap showing the differential immune score between tumor and normal, xcell scores were scaled across samples. **B** The violin plots showing the high expression of Th1 and Th2 geneset in tumor, geneset download form TISCH database. each point means a sample. **C**-**D**. Boxplot showing the expression of Th1 and Th2 marker genes in tumor and normal, T-test, *p < 0.05, **p < 0.01, ***p < 0.001. **E**: The heatmap showing the correlation between gene expression of HOX family gene (FPKM) and enrichment score of immune cells (z-score). **F**: The point plot showing the correlation between Th1 and HOXB7 (up), Th2 and HOXA7 (down)

Immune microenvironment provides tumor cell survival condition and has effect on tumor progression. Indeed, there was a significant difference of immune microenvironment between tumor and adjacent normal tissues as a result of xCell analysis. The scaled scores of CD8^+^ naive T and Th1/2 cell were higher in tumor (Fig. 4A). On the contrary, scores of CD4^+^ Tem and cDC cells were lower. Besides, not only the gene sets of Th1 and Th2 (Fig. 4B), but also their markers were highly expressed in tumor tissue of early ESCC (Fig. 4C-D). By correlation analysis, we found a positive correlation between the expression of HOX family genes and the enrichment scores of these cells. The enrichment scores of Th1 and Th2 cells were highly correlated with the expression of *HOXB7* and *HOXA7*, with a correlation coefficient of above 0.7. These results suggest that the HOX family is an important potential factor in regulating the microenvironment of early ESCC.

## Discussion

The systematic identification and discovery of new driver genes are crucial for cancer research of targeted drugs and therapeutic strategies. Traditional analysis at a single-omics such as a gene dataset may miss key drivers during tumorigenesis. Here, by integrating WES and transcriptomic analysis of 10 paired early ESCC patients, we established mutational and transcriptional landscape of early ESCC. We also found that a lower TMB and MSI score in Chinese early ESCC. Besides, there were six significant focal CNVs. In transcriptomic levels, the dramatic gene profile changes between tumor and adjacent normal tissues were identified, especially, in Hox family. Furthermore, we also found that immune microenvironment dysregulation, such as gene feature of Th1/2 enriched in tumor environment. These results can improve our understanding of the molecular characteristics of early ESCC, which provide potential mechanisms for early diagnosis and cancer development.

Lots of variations were identified by WES analysis in our study, such as *TP53* and *NOTCH1*, both of which may play an important role in development of ESCC [8, 26]. Interestingly, compared with TCGA-ESCC-early cohort, *HOXA13* and *IL2* were identified as novel mutations in Chinese early ESCC. In our study, we found the frameshift deletion occurred at positions 126–152 of exon 1 of *HOXA13* gene. Variants of *HOXA13* have been found to play important roles in diseases, for example, N372H and V375F were expected to reduce or eliminate the activity of *HOXA13* protein, and result in the human phenotype of Hand-foot-genital syndrome [31, 32]. The C insertion after base 1042 and/or a G to C substitution at base 1113 in intron 1 but not in exon of the *HOXA13* gene in cervical carcinoma cells has also been detected [33]. Another mutational gene, *IL2,* is a member of cytokine subfamily, whose mutation can also have effect on development of immune cells [34]. Two frameshift insertions were firstly observed in exon 1 of *IL2* (chr4:123,185,619 and chr4:123,185,617) in early ESCC sample. Although the mutations of *HOXA13* and *IL2* only exist in one patient due to sample size limitation, the mutation frequency of each gene is 10%. Whether the mutations of the genes are reliable and whether have biological functions in early ESCC needs further verification in a larger cohort.

Another significant difference is that compared with TCGA-EC cohort sequencing results, we found that TMB and MSI values were lower in early-ESCC samples from China. It is reported that TMB and MSI were associated with immune checkpoint inhibitors (ICI) response [35], which may provide an insight into ICI treatment for Chinese patients. In addition, a smaller number of significant focal CNVs were identified in early ESCC compared to other studies [26, 36]. Among them was the famous amplification of 8p11.23, 22q11.21 was also detected as a novel significant focal CNV in ESCC, which has been reported to associated with molecular and clinical profiling of acral melanoma [37]. Among genes in 22q11.21 region, *CECR2* as an epigenetic factor plays a vital role in DNA damage responses, which has also been reported to promote breast cancer metastasis via NF-kB and regulate macrophage [38]. Moreover, *CECR2* was also upregulated in our tumor samples, suggesting that the gene may act as a potential oncogene in early ESCC.

Through RNA sequencing analysis, we also unveiled the altered expression and transcriptional uniqueness of the transcriptome in early ESCC tissues compared to TCGA-ESCC-early cohort. Moreover, in addition to finding the cell cycle and EMT pathways enrichment in early ESCC samples using DAVID and GSEA analysis, we discovered several Hox genes were consistently upregulated in tumor. Hox family encodes TFs, a member of homeobox superfamily, which may have oncogenic effects on tumor development [39]. Our results found 12 of the 14 highly expressed Hox genes in early ESCC were also up-regulated in the TCGA-ESCC-early cohort. For instance, the expression of *HOXA9* (FC = 7.42) was significantly higher in Chinese patients and validated by RT-qPCR, while there were no significant changes in TCGA ESCC early cohort (*HOXA9*, FC = 1.87). Some previous studies have revealed the important role of *HOXA9* in ESCC. Jin Lv et al. detected higher expression levels of *HOXA9* in 70 ESCC than in adjacent tissues using RT-PCR and immunohistochemical staining, which was associated with the prognosis, pathological staging, and lymph node metastasis of ESCC patients [40]. Additionally, *HOXA9* binds to microRNA-186-5p to inhibit the proliferation and metastasis of esophageal cancer [41]. Chen et, al. [42]. also found significant upregulation of *HOXA9*, *HOXA7*, and *HOXC6* in 36 Chinese ESCC patients, suggesting that these genes may have potential oncogenic functions in the early development of ESCC. However, some HOX family genes were found to have high expression in the TCGA-ESCC-early cohort, but not in Chinese early ESCC samples, including *HOXC6* (TCGA ESCC early, FC = 3.32; early ESCC, FC = 1.31), despite its reported role in ESCC progression. For instance, studies by Chen et, al. [42]. and Li Tang have demonstrated that *HOXC6* is highly expressed in ESCC and promotes the malignant phenotype of ESCC cells [43]. Besides, Some HOX family genes, such as *HOXB13*, showed no significant fold changes in expression in both early ESCC and TCGA (early FC = 0.99; TCGA FC = 0.83). However, Erbao Zhang et al.have indicated that the upregulation of *HOXB13* promotes the growth and migration of cancer cells in ESCC [44]. This data suggested that the transcriptome specificity of early ESCC in China compared with foreign regions. It is limited to further confirm the genomic and transcriptomic influence because of lacking larger testing cohorts, which remains to investigate in the future. At the same time, related experiments need to further confirm the molecular biological mechanisms of overexpressed Hox genes in promoting early ESCC. For TME, the proportion of epithelial cells did not show a significant difference between tumor tissue and adjacent non-cancerous tissues of early-stage ESCC patients. However, according to Zhang's research, scRNA-seq analysis that included samples from patients with stage I to III ESCC showed that normal tissue had a higher enrichment of epithelial cells [45]. But our findings suggest that early-stage ESCC patients have a unique TME that differs from the adjacent non-cancerous tissue, particularly in terms of the significant enrichment of Th1 cells in tumor tissue. This is consistent with the results of Jishuai Zhang's study, which also found that Th1 cells were more enriched in ESCC [46]. Recently, there have been studies indicating that Th1 cells were predictive factors for pathological complete response (pCR) and play a role in regulating the chemosensitivity and radiosensitivity of ESCC [47]. Moreover, our study revealed a positive correlation (cor > 0.4) between the enrichment of Th1 cells and the expression of 4 HOX family genes (HOXB7, HOXA10, HOXA7, HOXC11, and HOXC10), especially HOXB7. These findings suggest that TME of early-stage ESCC is regulated by the HOX family of genes and provides potential markers for treatment response.

## Conclusion

Our study unmasked the unique genome and transcriptome in early ESCC of China region. We found that early ESCC displayed higher TMB status compared with TCGA-ESCC-early cohort. Three significant focal CNVs were identified, which had important implications for transcriptomics. Importantly, we also found that one-third of Hox family genes were significantly up-regulated in early ESCC, and validated by RT-qPCR analysis. Moreover, gene regulation network of HOX family was correlated with tumor development and metabolism remodeling. Together, we anticipate that these results could offer a fresh perspective on tumor initiation and give more guidance for developing precision medicine in early ESCC.

## Supplementary Information

**Additional file 1:** **Figure S1**. The 8p11.23 amplification impacted the transcriptome.

**Additional file 2:** **Table S1.** A summary of variations of early ESCC by WES.

**Additional file 3:** **Table S2.** The DEGs between tumor and normal.

**Additional file 4:** **TableS3.** Clinical data of the patients in this study. **Table S4.** TCGA samples used in the study. **Table S5.** Primers and RNA sequences used in this study.

## Data Availability

Raw data can be accessed form GSE213565.

## Abbreviation

ESCC: Esophageal squamous cell carcinoma
Early ESCC: Early Esophageal squamous cell carcinoma
WES: Whole-exome sequencing
TMB: Tumor mutational burden
MSI: Microsatellite instability
CNV: Copy number variation
GRN: Gene regulation network
