# Supplementary material for: Genomic and transcriptional characterization of early esophageal squamous cell carcinoma
Source: BMC Med Genomics. 2023 Jul 1;16:153. doi: 10.1186/s12920-023-01588-7 (PMC10315050; doi:10.1186/s12920-023-01588-7)
Supplement: Supplementary file 1 — Additional file 1: Figure S1. The 8p11.23 amplification impacted the transcriptome. [file 12920_2023_1588_MOESM1_ESM.docx]

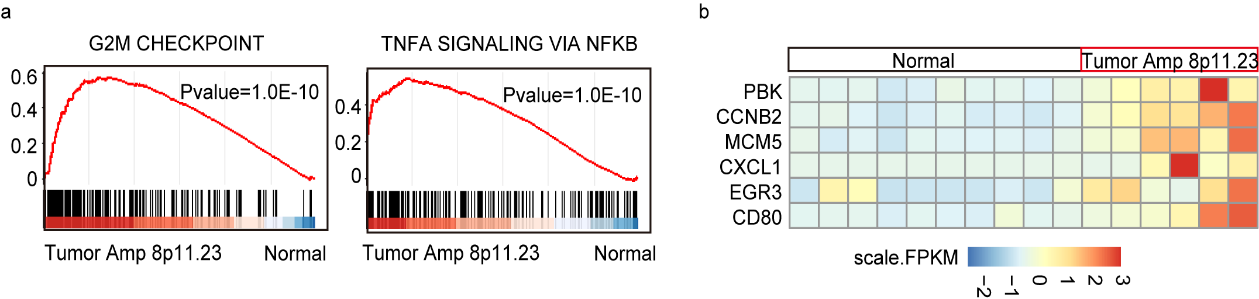


**Figure S1**. The 8p11.23 amplification impacted the transcriptome. (a)GSEA analysis for 8p11.23 -amplified tumor vs. normal. (b) heatmap shows that G2M and TNFA signaling via NFKB related-genes are highly expressed in 8p11.23-amplified tumors.
