# Supplementary material for: Genomic and transcriptional characterization of early esophageal squamous cell carcinoma
Source: BMC Med Genomics. 2023 Jul 1;16:153. doi: 10.1186/s12920-023-01588-7 (PMC10315050; doi:10.1186/s12920-023-01588-7)
Supplement: Supplementary file 2 — Additional file 2: Table S1. A summary of variations of early ESCC by WES. [file 12920_2023_1588_MOESM2_ESM.pdf]

Table S1: A summary of variations of early ESCC by WES

Chromosome	Start_Position	End_Position	Reference_A	Tumor_Seq_A	Tumor_Sample	Hugo_Symbol	Variant_Classification
chr1	101704924	101704924	G	C	ESCA15T_AF	S1PR1	Silent
chr1	103444443	103444443	T	A	ESCA20T_AF	COL11A1	Missense_Mutation
chr1	103491415	103491415	A	C	ESCA19T_AF	COL11A1	Missense_Mutation
chr1	10425480	10425480	G	T	ESCA10T_AF	KIF1B	Missense_Mutation
chr1	110051522	110051522	G	A	ESCA01T_AF	AMIGO1	Missense_Mutation
chr1	110883515	110883515	-	GCTTTCATA(	ESCA14T_AF	RBM15	Frame_Shift_Ins
chr1	110884434	110884434	A	G	ESCA16T_AF	RBM15	Missense_Mutation
chr1	11159823	11159823	T	C	ESCA16T_AF	EXOSC10	Silent
chr1	114133210	114133210	G	C	ESCA14T_AF	MAGI3	Missense_Mutation
chr1	114514460	114514460	A	-	ESCA15T_AF	HIPK1	Frame_Shift_Del
chr1	114516076	114516076	C	T	ESCA16T_AF	HIPK1	Missense_Mutation
chr1	11595632	11595632	G	A	ESCA07T_AF	DISP3	Silent
chr1	117150851	117150851	C	T	ESCA19T_AF	IGSF3	Missense_Mutation
chr1	11861224	11861224	G	A	ESCA15T_AF	MTHFR	Missense_Mutation
chr1	11888224	11888224	G	A	ESCA19T_AF	CLCN6	Missense_Mutation
chr1	119427454	119427454	G	A	ESCA16T_AF	TBX15	Silent
chr1	12262124	12262124	C	T	ESCA15T_AF	TNFRSF1B	Missense_Mutation
chr1	1267562	1267562	G	A	ESCA16T_AF	TAS1R3	Silent
chr1	12835823	12835823	A	C	ESCA10T_AF	PRAMEF12	Missense_Mutation
chr1	13001109	13001109	A	C	ESCA19T_AF	PRAMEF27;PRAI	Missense_Mutation
chr1	13036714	13036714	-	CTGAGGAG(	ESCA16T_AF	PRAMEF18	In_Frame_Ins
chr1	13183834	13183834	G	A	ESCA15T_AF	HNRNPCL2	Silent
chr1	1374932	1374932	-	GCGGCCCG	ESCA10T_AF	VWA1	Frame_Shift_Ins
chr1	144881533	144881533	T	G	ESCA17T_AF	PDE4DIP	Silent
chr1	145414849	145414849	T	G	ESCA18T_AF	HJV	Missense_Mutation
chr1	145440300	145440300	G	C	ESCA17T_AF	TXNIP	Missense_Mutation
chr1	149921607	149921607	G	C	ESCA16T_AF	OTUD7B	Missense_Mutation
chr1	149921612	149921612	G	T	ESCA16T_AF	OTUD7B	Missense_Mutation
chr1	150484274	150484274	-	CAGGGGAA(	ESCA15T_AF	ECM1	Frame_Shift_Ins
chr1	151107771	151107771	C	T	ESCA07T_AF	SEMA6C	Missense_Mutation
chr1	151373784	151373784	C	T	ESCA20T_AF	PSMB4	Missense_Mutation
chr1	151754048	151754048	-	TGATCTGTG(	ESCA07T_AF	TDRKH	In_Frame_Ins
chr1	151754048	151754048	-	TGATCTGTG(	ESCA16T_AF	TDRKH	In_Frame_Ins
chr1	151789193	151789193	C	T	ESCA07T_AF	RORC	Missense_Mutation
chr1	152286107	152286107	C	T	ESCA20T_AF	FLG	Missense_Mutation
chr1	153749168	153749168	A	G	ESCA15T_AF	SLC27A3	Missense_Mutation
chr1	153749175	153749175	C	T	ESCA15T_AF	SLC27A3	Silent
chr1	15386730	15386730	G	A	ESCA19T_AF	KAZN	Missense_Mutation
chr1	153926788	153926788	-	TCACATTGA(	ESCA14T_AF	CRTC2	Nonsense_Mutation
chr1	154437648	154437648	G	T	ESCA20T_AF	IL6R	Missense_Mutation
chr1	154962116	154962116	TG	-	ESCA17T_AF	FLAD1	Frame_Shift_Del
chr1	155178787	155178787	G	A	ESCA15T_AF	MTX1	Silent
chr1	155448032	155448032	G	A	ESCA15T_AF	ASH1L	Silent
chr1	155582089	155582089	T	A	ESCA16T_AF	MSTO1	Silent
chr1	156106058	156106058	C	T	ESCA07T_AF	LMNA	Missense_Mutation
chr1	156902259	156902259	G	A	ESCA16T_AF	LRRC71	Silent
chr1	157489535	157489535	G	A	ESCA16T_AF	FCRL5	Silent
chr1	15809876	15809876	G	A	ESCA16T_AF	CELA2B	Silent
chr1	158549500	158549500	-	ACCCATGAT	ESCA10T_AF	OR10X1	In_Frame_Ins
chr1	158612618	158612618	C	T	ESCA01T_AF	SPTA1	Missense_Mutation
chr1	159783343	159783343	C	T	ESCA19T_AF	FCRL6	Silent
chr1	159842937	159842937	C	T	ESCA07T_AF	CFAP45	Silent
chr1	159904585	159904585	T	A	ESCA19T_AF	IGSF9	Missense_Mutation
chr1	159907581	159907581	G	A	ESCA07T_AF	IGSF9	Missense_Mutation
chr1	160001408	160001408	C	T	ESCA01T_AF	PIGM	Missense_Mutation
chr1	160054364	160054364	C	T	ESCA17T_AF	KCNJ9	Missense_Mutation

chr1	160063077	160063077	-	TGGTGAACG	ESCA16T_AF	IGSF8	In_Frame_Ins
chr1	16054769	16054769	G	A	ESCA19T_AF	PLEKHM2	Missense_Mutation
chr1	161026217	161026217	C	T	ESCA07T_AF	ARHGAP30	Silent
chr1	161161951	161161951	C	T	ESCA14T_AF	ADAMTS4	Missense_Mutation
chr1	161168270	161168270	G	T	ESCA14T_AF	ADAMTS4	Silent
chr1	161681252	161681252	A	C	ESCA10T_AF	FCRLA	Missense_Mutation
chr1	161681256	161681256	-	CTTGGACTG	ESCA10T_AF	FCRLA	Frame_Shift_Ins
chr1	161987209	161987209	C	T	ESCA15T_AF	OLFML2B	Missense_Mutation
chr1	162343963	162343963	C	T	ESCA07T_AF	SPATA46	Missense_Mutation
chr1	162344102	162344102	C	T	ESCA16T_AF	SPATA46	Silent
chr1	16263707	16263707	C	G	ESCA01T_AF	SPEN	Missense_Mutation
chr1	169515788	169515788	C	T	ESCA15T_AF	F5	Missense_Mutation
chr1	169798523	169798523	A	G	ESCA14T_AF	C1orf112	Missense_Mutation
chr1	176564578	176564578	G	A	ESCA17T_AF	PAPPA2	Missense_Mutation
chr1	176853564	176853564	G	A	ESCA15T_AF	ASTN1	Missense_Mutation
chr1	177915649	177915649	-	CCGCAGCTG	ESCA07T_AF	CRYZL2P-SEC16	Frame_Shift_Ins
chr1	177916977	177916977	G	A	ESCA16T_AF	CRYZL2P-SEC16	Missense_Mutation
chr1	180062484	180062484	A	T	ESCA18T_AF	CEP350	Missense_Mutation
chr1	181452978	181452978	C	T	ESCA15T_AF	CACNA1E	Missense_Mutation
chr1	181479711	181479711	G	A	ESCA14T_AF	CACNA1E	Missense_Mutation
chr1	183197551	183197551	C	T	ESCA16T_AF	LAMC2	Missense_Mutation
chr1	183208678	183208678	-	T	ESCA18T_AF	LAMC2	Frame_Shift_Ins
chr1	183209484	183209484	C	G	ESCA15T_AF	LAMC2	Missense_Mutation
chr1	183895363	183895363	G	C	ESCA16T_AF	RGL1	Missense_Mutation
chr1	185153382	185153382	T	G	ESCA17T_AF	SWT1	Missense_Mutation
chr1	185959558	185959558	C	T	ESCA19T_AF	HMCN1	Silent
chr1	185984569	185984569	-	TT	ESCA15T_AF	HMCN1	Frame_Shift_Ins
chr1	186047322	186047322	G	A	ESCA15T_AF	HMCN1	Missense_Mutation
chr1	186646856	186646856	G	A	ESCA14T_AF	PTGS2	Silent
chr1	19062313	19062313	G	A	ESCA15T_AF	PAX7	Missense_Mutation
chr1	193150320	193150320	C	T	ESCA15T_AF	B3GALT2	Missense_Mutation
chr1	196749002	196749002	C	G	ESCA19T_AF	CFHR3	Missense_Mutation
chr1	197111819	197111819	G	A	ESCA15T_AF	ASPM	Silent
chr1	197115373	197115373	C	T	ESCA19T_AF	ASPM	Silent
chr1	200610321	200610321	AC	-	ESCA17T_AF	DDX59	Frame_Shift_Del
chr1	200633100	200633100	C	A	ESCA15T_AF	DDX59	Missense_Mutation
chr1	200950215	200950215	G	C	ESCA18T_AF	KIF21B	Missense_Mutation
chr1	201177424	201177424	G	A	ESCA10T_AF	IGFN1	Missense_Mutation
chr1	201181994	201181994	A	G	ESCA16T_AF	IGFN1	Missense_Mutation
chr1	202127381	202127381	G	A	ESCA14T_AF	PTPN7	Silent
chr1	204159845	204159845	C	A	ESCA19T_AF	KISS1	Missense_Mutation
chr1	20490564	20490564	C	G	ESCA18T_AF	PLA2G2C	Missense_Mutation
chr1	204970302	204970302	T	C	ESCA16T_AF	NFASC	Silent
chr1	205022316	205022316	G	-	ESCA15T_AF	CNTN2	Frame_Shift_Del
chr1	205631993	205631993	G	A	ESCA17T_AF	SLC45A3	Missense_Mutation
chr1	205764108	205764108	C	T	ESCA01T_AF	SLC41A1	Missense_Mutation
chr1	205890889	205890889	G	A	ESCA15T_AF	SLC26A9	Silent
chr1	206225152	206225152	C	T	ESCA15T_AF	AVPR1B	Missense_Mutation
chr1	21009233	21009233	G	A	ESCA17T_AF	KIF17	Silent
chr1	212148698	212148698	G	A	ESCA18T_AF	INTS7	Missense_Mutation
chr1	212274021	212274021	G	T	ESCA15T_AF	DTL	Silent
chr1	213405563	213405563	A	T	ESCA16T_AF	RPS6KC1	Missense_Mutation
chr1	216591912	216591912	T	A	ESCA10T_AF	USH2A	Nonsense_Mutation
chr1	216850824	216850824	G	T	ESCA16T_AF	ESRRG	Nonsense_Mutation
chr1	22150160	22150160	G	T	ESCA16T_AF	HSPG2	Silent
chr1	22201409	22201409	-	GTGGGCAGC	ESCA07T_AF	HSPG2	In_Frame_Ins
chr1	222802837	222802837	C	T	ESCA01T_AF	MIA3	Nonsense_Mutation
chr1	222892358	222892358	TTCTC	-	ESCA10T_AF	BROX	Frame_Shift_Del

chr1	2237630	2237630	-	CCCGCCTCG	ESCA10T_AF	SKI	Frame_Shift_Ins
chr1	223951856	223951856	C	T	ESCA17T_AF	CAPN2	Missense_Mutation
chr1	225533684	225533684	A	G	ESCA16T_AF	DNAH14	Missense_Mutation
chr1	226475434	226475434	C	T	ESCA16T_AF	LIN9	Missense_Mutation
chr1	228336527	228336527	-	CCAGGATGT	ESCA14T_AF	GUK1	In_Frame_Ins
chr1	228596285	228596285	GCCTCCCAC	-	ESCA20T_AF	TRIM17	Frame_Shift_Del
chr1	231488524	231488524	C	T	ESCA16T_AF	SPRTN	Missense_Mutation
chr1	232144583	232144583	C	T	ESCA15T_AF	DISC1	Nonsense_Mutation
chr1	232561348	232561348	C	G	ESCA19T_AF	SIPA1L2	Missense_Mutation
chr1	233515413	233515413	G	A	ESCA16T_AF	MAP3K21	Silent
chr1	234584231	234584231	C	T	ESCA16T_AF	TARBP1	Silent
chr1	234744384	234744384	C	T	ESCA19T_AF	IRF2BP2	Missense_Mutation
chr1	236721674	236721674	A	T	ESCA20T_AF	HEATR1	Missense_Mutation
chr1	240601407	240601407	G	A	ESCA17T_AF	FMN2	Missense_Mutation
chr1	241032117	241032117	C	T	ESCA17T_AF	RGS7	Silent
chr1	24125491	24125491	-	TGCCAGG	ESCA16T_AF	GALE	Frame_Shift_Ins
chr1	241665851	241665851	C	T	ESCA10T_AF	FH	Silent
chr1	241669312	241669312	C	A	ESCA16T_AF	FH	Missense_Mutation
chr1	242253230	242253230	G	C	ESCA07T_AF	PLD5	Missense_Mutation
chr1	247588053	247588053	C	T	ESCA16T_AF	NLRP3	Silent
chr1	247614762	247614762	G	A	ESCA19T_AF	OR2B11	Missense_Mutation
chr1	248569618	248569618	C	T	ESCA19T_AF	OR2T1	Missense_Mutation
chr1	248616229	248616229	T	G	ESCA16T_AF	OR2T2	Missense_Mutation
chr1	249211965	249211965	GAGGGG	-	ESCA16T_AF	PGBD2	In_Frame_Del
chr1	249211970	249211970	-	CCCCTC	ESCA16T_AF	PGBD2	In_Frame_Ins
chr1	26355686	26355686	C	T	ESCA17T_AF	EXTL1	Missense_Mutation
chr1	26358042	26358042	C	T	ESCA10T_AF	EXTL1	Silent
chr1	26596094	26596094	C	T	ESCA10T_AF	CEP85	Silent
chr1	27480785	27480785	C	T	ESCA17T_AF	SLC9A1	Missense_Mutation
chr1	27874217	27874217	-	ATTGGTACC	ESCA15T_AF	AHDC1	Nonsense_Mutation
chr1	2938854	2938854	C	T	ESCA17T_AF	ACTRT2	Missense_Mutation
chr1	32196718	32196718	G	A	ESCA14T_AF	ADGRB2	Missense_Mutation
chr1	32669583	32669583	G	A	ESCA07T_AF	CCDC28B	Missense_Mutation
chr1	33160973	33160973	G	A	ESCA01T_AF	SYNC	Silent
chr1	33430157	33430157	G	C	ESCA19T_AF	RNF19B	Missense_Mutation
chr1	33480189	33480189	A	-	ESCA19T_AF	AK2	Frame_Shift_Del
chr1	3548209	3548209	-	TCCCAATGC	ESCA10T_AF	WRAP73	Frame_Shift_Ins
chr1	36354042	36354042	C	G	ESCA20T_AF	AGO1	Missense_Mutation
chr1	36557639	36557639	C	A	ESCA16T_AF	ADPRHL2	Silent
chr1	36557645	36557645	-	ATCACCCTC	ESCA16T_AF	ADPRHL2	Frame_Shift_Ins
chr1	36935377	36935377	C	T	ESCA07T_AF	CSF3R	Silent
chr1	3774176	3774176	-	AAGCCCAAC	ESCA07T_AF	DFFB	Frame_Shift_Ins
chr1	39818902	39818902	A	G	ESCA01T_AF	MACF1	Missense_Mutation
chr1	40254057	40254057	C	T	ESCA01T_AF	BMP8B	Missense_Mutation
chr1	40775834	40775834	G	A	ESCA19T_AF	COL9A2	Silent
chr1	41847704	41847704	C	T	ESCA17T_AF	FOXO6	Silent
chr1	41949800	41949800	G	A	ESCA18T_AF	EDN2	Missense_Mutation
chr1	42915617	42915617	T	G	ESCA07T_AF	ZMYND12	Missense_Mutation
chr1	43908526	43908526	G	A	ESCA15T_AF	SZT2	Missense_Mutation
chr1	43917516	43917516	C	T	ESCA16T_AF	HYI	Missense_Mutation
chr1	44365395	44365395	-	CTTACCACT	ESCA16T_AF	ST3GAL3	Frame_Shift_Ins
chr1	44804991	44804991	A	T	ESCA10T_AF	ERI3	Nonsense_Mutation
chr1	45163739	45163739	C	T	ESCA16T_AF	ARMH1	Missense_Mutation
chr1	45476663	45476663	G	A	ESCA16T_AF	HECTD3	Silent
chr1	46871402	46871402	-	GATGCC	ESCA16T_AF	FAAH	In_Frame_Ins
chr1	46871405	46871405	-	GAAGGAGG	ESCA16T_AF	FAAH	In_Frame_Ins
chr1	47746035	47746035	-	CTGTGGGTC	ESCA10T_AF	STIL	Frame_Shift_Ins
chr1	50884781	50884781	G	A	ESCA17T_AF	DMRTA2	Silent

chr1	52260496	52260496	C	A	ESCA07T_AF	NRDC	Missense_Mutation
chr1	53222285	53222285	-	AAAAGCCAT	ESCA15T_AF	ZYG11B	Frame_Shift_Ins
chr1	53553854	53553854	C	T	ESCA16T_AF	SLC1A7	Missense_Mutation
chr1	53558357	53558357	C	G	ESCA16T_AF	SLC1A7	Silent
chr1	55223585	55223585	-	ACGACAGGA	ESCA07T_AF	PARS2	Frame_Shift_Ins
chr1	55223587	55223587	-	TGACCATCG	ESCA07T_AF	PARS2	Frame_Shift_Ins
chr1	55282720	55282720	G	A	ESCA17T_AF	LEXM	Missense_Mutation
chr1	56990113	56990113	-	CAGCCAAAC	ESCA16T_AF	PLPP3	Nonsense_Mutation
chr1	58971758	58971758	C	G	ESCA07T_AF	OMA1	Missense_Mutation
chr1	5926507	5926507	T	C	ESCA16T_AF	NPHP4	Silent
chr1	6206893	6206893	-	CGGATTCTA	ESCA14T_AF	CHD5	In_Frame_Ins
chr1	62675846	62675846	C	A	ESCA17T_AF	L1TD1	Missense_Mutation
chr1	63789782	63789782	C	A	ESCA16T_AF	FOXD3	Silent
chr1	65131823	65131823	-	TGATTGTCAC	ESCA16T_AF	CACHD1	In_Frame_Ins
chr1	6534647	6534647	-	TGTCCTGGG	ESCA07T_AF	PLEKHG5	In_Frame_Ins
chr1	66384345	66384345	G	A	ESCA07T_AF	PDE4B	Silent
chr1	68903942	68903942	C	T	ESCA16T_AF	RPE65	Silent
chr1	76384652	76384652	C	A	ESCA20T_AF	ASB17	Silent
chr1	77093180	77093180	C	A	ESCA16T_AF	ST6GALNAC3	Missense_Mutation
chr1	84864256	84864256	G	C	ESCA16T_AF	DNASE2B	Missense_Mutation
chr1	84945169	84945169	C	T	ESCA14T_AF	RPF1	Missense_Mutation
chr1	86947957	86947957	A	G	ESCA14T_AF	CLCA1	Silent
chr1	89225922	89225922	G	C	ESCA15T_AF	PKN2	Missense_Mutation
chr1	89616175	89616175	C	G	ESCA15T_AF	GBP7	Missense_Mutation
chr1	89730634	89730634	-	CCAGGTTCT	ESCA10T_AF	GBP5	Nonsense_Mutation
chr1	92647110	92647110	T	G	ESCA18T_AF	KIAA1107	Silent
chr1	93101786	93101786	C	A	ESCA07T_AF	EVI5	Missense_Mutation
chr1	9322121	9322121	G	A	ESCA16T_AF	H6PD	Missense_Mutation
chr1	94674444	94674444	T	A	ESCA18T_AF	ARHGAP29	Missense_Mutation
chr10	100503735	100503735	C	T	ESCA16T_AF	HPSE2	Missense_Mutation
chr10	102510633	102510633	T	C	ESCA18T_AF	PAX2	Missense_Mutation
chr10	102738099	102738099	G	A	ESCA01T_AF	SEMA4G	Silent
chr10	102738104	102738104	G	C	ESCA01T_AF	SEMA4G	Missense_Mutation
chr10	102749139	102749139	G	A	ESCA18T_AF	TWNK	Missense_Mutation
chr10	102770056	102770056	T	A	ESCA10T_AF	PDZD7	Missense_Mutation
chr10	103454423	103454423	G	T	ESCA16T_AF	FBXW4	Missense_Mutation
chr10	103761779	103761779	G	C	ESCA18T_AF	ARMH3	Missense_Mutation
chr10	105778665	105778665	-	ATTTTCCTTT	ESCA16T_AF	SLK	Nonsense_Mutation
chr10	107022161	107022161	C	A	ESCA19T_AF	SORCS3	Missense_Mutation
chr10	11207528	11207528	G	A	ESCA16T_AF	CELF2	Missense_Mutation
chr10	112581411	112581411	G	A	ESCA07T_AF	RBM20	Missense_Mutation
chr10	115343107	115343107	C	-	ESCA14T_AF	HABP2	Frame_Shift_Del
chr10	118236218	118236218	C	T	ESCA16T_AF	PNLIPRP3	Silent
chr10	120820273	120820273	G	A	ESCA15T_AF	EIF3A	Silent
chr10	124742281	124742281	G	A	ESCA15T_AF	PSTK	Silent
chr10	124922309	124922309	-	AGG	ESCA10T_AF	BUB3	In_Frame_Ins
chr10	126517989	126517989	G	A	ESCA16T_AF	ABRAXAS2	Silent
chr10	129903016	129903016	T	C	ESCA16T_AF	MKI67	Missense_Mutation
chr10	13230950	13230950	C	T	ESCA16T_AF	MCM10	Silent
chr10	132902536	132902536	G	A	ESCA14T_AF	TCERG1L	Nonsense_Mutation
chr10	134121106	134121106	G	A	ESCA15T_AF	STK32C	Missense_Mutation
chr10	134218772	134218772	-	CTTGCCCTG	ESCA15T_AF	PWWP2B	Frame_Shift_Ins
chr10	134755215	134755215	-	TGCAGATGA	ESCA15T_AF	CFAP46	Frame_Shift_Ins
chr10	134996953	134996953	C	T	ESCA19T_AF	KNDC1	Missense_Mutation
chr10	135116307	135116307	G	T	ESCA14T_AF	TUBGCP2	Missense_Mutation
chr10	135141519	135141519	C	T	ESCA10T_AF	CALY	Missense_Mutation
chr10	135369156	135369156	G	A	ESCA18T_AF	SYCE1	Nonsense_Mutation
chr10	16967622	16967622	T	G	ESCA19T_AF	CUBN	Silent

chr10	18690934	18690934	G	A	ESCA17T_AF	CACNB2	Missense_Mutation
chr10	18823074	18823074	C	T	ESCA17T_AF	CACNB2	Missense_Mutation
chr10	19498358	19498358	A	T	ESCA19T_AF	MALRD1	Missense_Mutation
chr10	21097452	21097452	C	T	ESCA10T_AF	NEBL	Silent
chr10	23297301	23297301	C	T	ESCA16T_AF	ARMC3	Silent
chr10	24508676	24508676	A	T	ESCA07T_AF	KIAA1217	Silent
chr10	24508677	24508677	-	GGGTGTGTC	ESCA07T_AF	KIAA1217	In_Frame_Ins
chr10	24833137	24833137	C	T	ESCA16T_AF	KIAA1217	Silent
chr10	25313207	25313207	A	G	ESCA17T_AF	THNSL1	Missense_Mutation
chr10	25885710	25885710	G	T	ESCA10T_AF	GPR158	Missense_Mutation
chr10	25885712	25885712	-	TGGCATTAC	ESCA10T_AF	GPR158	In_Frame_Ins
chr10	27381349	27381349	T	C	ESCA16T_AF	ANKRD26	Silent
chr10	27409411	27409411	C	T	ESCA16T_AF	YME1L1	Missense_Mutation
chr10	27434483	27434483	G	A	ESCA16T_AF	YME1L1	Silent
chr10	292901	292901	-	TGCTCATTA(	ESCA10T_AF	ZMYND11	Frame_Shift_Ins
chr10	292904	292904	T	G	ESCA10T_AF	ZMYND11	Missense_Mutation
chr10	29811461	29811461	C	A	ESCA19T_AF	SVIL	Silent
chr10	30316872	30316872	T	C	ESCA16T_AF	JCAD	Silent
chr10	31137565	31137565	-	GCACTTCTT	ESCA15T_AF	ZNF438	Nonsense_Mutation
chr10	3149413	3149413	C	T	ESCA20T_AF	PFKP	Missense_Mutation
chr10	3208544	3208544	-	GGTGTTCCT(	ESCA07T_AF	PITRM1	In_Frame_Ins
chr10	32856773	32856773	A	T	ESCA16T_AF	CCDC7	Missense_Mutation
chr10	33502344	33502344	C	T	ESCA15T_AF	NRP1	Missense_Mutation
chr10	34630574	34630574	T	C	ESCA14T_AF	PARD3	Missense_Mutation
chr10	35897350	35897350	-	TTTTCGCTG(	ESCA14T_AF	GJD4	In_Frame_Ins
chr10	37451712	37451712	T	G	ESCA19T_AF	ANKRD30A	Silent
chr10	48385935	48385935	C	T	ESCA18T_AF	RBP3	Missense_Mutation
chr10	49939168	49939168	G	C	ESCA15T_AF	WDFY4	Missense_Mutation
chr10	50732152	50732152	C	T	ESCA10T_AF	ERCC6	Missense_Mutation
chr10	53458210	53458210	-	TTATCTGGG	ESCA15T_AF	CSTF2T	Frame_Shift_Ins
chr10	5945115	5945115	-	CTCTTTTTGT	ESCA14T_AF	FBH1	Frame_Shift_Ins
chr10	61830077	61830077	-	TGAAGTAAG	ESCA10T_AF	ANK3	Nonsense_Mutation
chr10	63978060	63978060	TCTA	-	ESCA07T_AF	RTKN2	Frame_Shift_Del
chr10	69991075	69991075	GTGGTCGC(	-	ESCA18T_AF	ATOH7	Frame_Shift_Del
chr10	70243222	70243222	G	A	ESCA07T_AF	SLC25A16	Silent
chr10	71905232	71905232	G	A	ESCA19T_AF	TYSND1	Missense_Mutation
chr10	71906150	71906150	T	C	ESCA16T_AF	TYSND1	Missense_Mutation
chr10	73405673	73405673	C	T	ESCA10T_AF	CDH23	Missense_Mutation
chr10	73574843	73574843	G	A	ESCA16T_AF	CDH23	Silent
chr10	75407991	75407991	-	TTTACCCCG	ESCA16T_AF	SYNPO2L	In_Frame_Ins
chr10	75874094	75874094	G	A	ESCA16T_AF	VCL	Silent
chr10	75874100	75874100	-	GGTTCTAAT(	ESCA16T_AF	VCL	Frame_Shift_Ins
chr10	76789986	76789986	G	T	ESCA17T_AF	KAT6B	Missense_Mutation
chr10	79589985	79589985	C	A	ESCA19T_AF	DLG5	Missense_Mutation
chr10	859125	859125	-	GCCCAGCTA	ESCA15T_AF	LARP4B	In_Frame_Ins
chr10	859127	859127	-	AGAGAAGG/	ESCA15T_AF	LARP4B	Nonsense_Mutation
chr10	85958855	85958855	C	G	ESCA10T_AF	CDHR1	Missense_Mutation
chr10	88232033	88232033	G	C	ESCA15T_AF	WAPL	Missense_Mutation
chr10	91497912	91497912	A	G	ESCA16T_AF	KIF20B	Missense_Mutation
chr10	93999487	93999487	G	A	ESCA17T_AF	CPEB3	Silent
chr10	94268534	94268534	G	A	ESCA16T_AF	IDE	Silent
chr10	95111022	95111022	G	C	ESCA15T_AF	MYOF	Missense_Mutation
chr10	98741293	98741293	A	G	ESCA16T_AF	LCOR	Missense_Mutation
chr10	98742021	98742021	C	T	ESCA16T_AF	LCOR	Silent
chr10	99197506	99197506	-	CCTGTAAAG	ESCA07T_AF	EXOSC1	Nonsense_Mutation
chr10	99338328	99338328	T	C	ESCA14T_AF	ANKRD2	Missense_Mutation
chr10	99338334	99338334	-	CCCCCCGTC	ESCA14T_AF	ANKRD2	In_Frame_Ins
chr10	99350079	99350079	C	A	ESCA19T_AF	C10orf62	Missense_Mutation

chr11	1017467	1017467	G	T	ESCA15T_AF	MUC6	Silent
chr11	102826138	102826138	G	A	ESCA14T_AF	MMP13	Nonsense_Mutation
chr11	108264047	108264047	G	A	ESCA15T_AF	C11orf65	Missense_Mutation
chr11	108593896	108593896	-	CCTTTTTGA/	ESCA14T_AF	DDX10	In_Frame_Ins
chr11	1101602	1101602	C	A	ESCA16T_AF	MUC2	Missense_Mutation
chr11	1103242	1103242	G	A	ESCA16T_AF	MUC2	Silent
chr11	111431178	111431178	-	TC	ESCA14T_AF	LAYN	Frame_Shift_Ins
chr11	111746424	111746424	G	C	ESCA15T_AF	FDXACB1	Nonsense_Mutation
chr11	113194168	113194168	A	C	ESCA16T_AF	TTC12	Missense_Mutation
chr11	113270015	113270015	G	C	ESCA16T_AF	ANKK1	Missense_Mutation
chr11	116692324	116692324	G	A	ESCA19T_AF	APOA4	Silent
chr11	116706774	116706774	-	CGACCGCG(	ESCA15T_AF	APOA1	Frame_Shift_Ins
chr11	117090422	117090422	G	T	ESCA18T_AF	PCSK7	Missense_Mutation
chr11	117261921	117261921	A	T	ESCA15T_AF	CEP164	Missense_Mutation
chr11	117261925	117261925	-	TCCCCACTT(	ESCA15T_AF	CEP164	Nonsense_Mutation
chr11	117982582	117982582	-	AGTGGGGG1	ESCA15T_AF	TMPRSS4	Frame_Shift_Ins
chr11	118889996	118889996	G	A	ESCA15T_AF	TRAPPC4	Missense_Mutation
chr11	119229838	119229838	-	TCCTTCCTTT	ESCA16T_AF	USP2	Nonsense_Mutation
chr11	121000804	121000804	G	A	ESCA19T_AF	TECTA	Missense_Mutation
chr11	121016604	121016604	C	T	ESCA18T_AF	TECTA	Missense_Mutation
chr11	122774868	122774868	G	C	ESCA07T_AF	JHY	Missense_Mutation
chr11	122954423	122954423	C	G	ESCA18T_AF	CLMP	Missense_Mutation
chr11	123596946	123596946	-	CGCTGCTGA	ESCA14T_AF	ZNF202	Frame_Shift_Ins
chr11	123777817	123777817	C	T	ESCA19T_AF	OR8D4	Missense_Mutation
chr11	123813823	123813823	G	A	ESCA16T_AF	OR6T1	Silent
chr11	123813940	123813940	G	C	ESCA16T_AF	OR6T1	Silent
chr11	123814489	123814489	A	G	ESCA16T_AF	OR6T1	Silent
chr11	124252718	124252718	G	A	ESCA16T_AF	OR8B2	Silent
chr11	124252750	124252750	G	A	ESCA16T_AF	OR8B2	Missense_Mutation
chr11	124481558	124481558	C	T	ESCA07T_AF	PANX3	Missense_Mutation
chr11	124749741	124749741	G	A	ESCA17T_AF	ROBO3	Silent
chr11	12499398	12499398	T	C	ESCA19T_AF	PARVA	Missense_Mutation
chr11	126277222	126277222	G	T	ESCA19T_AF	ST3GAL4	Silent
chr11	1268824	1268824	G	A	ESCA20T_AF	MUC5B	Missense_Mutation
chr11	128354827	128354827	CGAGGGG	-	ESCA10T_AF	ETS1	Nonsense_Mutation
chr11	128354835	128354835	-	CCCCTCG	ESCA10T_AF	ETS1	Frame_Shift_Ins
chr11	128781960	128781960	C	T	ESCA01T_AF	KCNJ5	Silent
chr11	128844151	128844151	G	C	ESCA14T_AF	ARHGAP32	Missense_Mutation
chr11	129996680	129996680	G	T	ESCA16T_AF	APLP2	Missense_Mutation
chr11	130784918	130784918	G	A	ESCA15T_AF	SNX19	Missense_Mutation
chr11	132177683	132177683	G	A	ESCA01T_AF	NTM	Silent
chr11	133712447	133712447	T	C	ESCA17T_AF	SPATA19	Missense_Mutation
chr11	134022871	134022871	G	T	ESCA19T_AF	NCAPD3	Missense_Mutation
chr11	14992705	14992705	G	A	ESCA15T_AF	CALCA	Missense_Mutation
chr11	19177450	19177450	-	T	ESCA16T_AF	ZDHHC13	Frame_Shift_Ins
chr11	212845	212845	T	-	ESCA14T_AF	RIC8A	Frame_Shift_Del
chr11	290715	290715	G	A	ESCA20T_AF	PGGHG	Missense_Mutation
chr11	30255338	30255338	-	TCCACTGAT(	ESCA16T_AF	FSHB	Frame_Shift_Ins
chr11	31329294	31329294	T	A	ESCA01T_AF	DCDC1	Missense_Mutation
chr11	32636041	32636041	G	T	ESCA07T_AF	CCDC73	Missense_Mutation
chr11	34515238	34515238	G	A	ESCA15T_AF	ELF5	Missense_Mutation
chr11	34909975	34909975	C	G	ESCA16T_AF	APIP	Missense_Mutation
chr11	35456297	35456297	C	T	ESCA18T_AF	PAMR1	Silent
chr11	36597820	36597820	A	C	ESCA18T_AF	RAG1	Missense_Mutation
chr11	40136143	40136143	T	C	ESCA07T_AF	LRRC4C	Missense_Mutation
chr11	40136793	40136793	T	A	ESCA16T_AF	LRRC4C	Silent
chr11	4406701	4406701	C	T	ESCA15T_AF	TRIM21	Silent
chr11	45245907	45245907	C	T	ESCA17T_AF	PRDM11	Silent

chr11	4608547	4608547	C	T	ESCA15T_AF	OR52I2	Nonsense_Mutation
chr11	46331616	46331616	-	TACCCGGTG	ESCA10T_AF	CREB3L1	In_Frame_Ins
chr11	46406767	46406767	G	A	ESCA16T_AF	CHRM4	Silent
chr11	46407750	46407750	C	T	ESCA16T_AF	CHRM4	Missense_Mutation
chr11	4673936	4673936	G	T	ESCA16T_AF	OR51E1	Missense_Mutation
chr11	46804824	46804824	C	T	ESCA20T_AF	CKAP5	Missense_Mutation
chr11	48149511	48149511	C	T	ESCA10T_AF	PTPRJ	Missense_Mutation
chr11	4928921	4928921	ACTGT	-	ESCA15T_AF	OR51A7	Frame_Shift_Del
chr11	4928929	4928929	-	ACAGT	ESCA15T_AF	OR51A7	Frame_Shift_Ins
chr11	4929049	4929049	T	A	ESCA16T_AF	OR51A7	Silent
chr11	51411459	51411459	C	T	ESCA07T_AF	OR4A5	Missense_Mutation
chr11	55135393	55135393	A	T	ESCA17T_AF	OR4A15	Missense_Mutation
chr11	55135856	55135856	G	A	ESCA19T_AF	OR4A15	Missense_Mutation
chr11	55419127	55419127	G	A	ESCA18T_AF	OR4S2	Missense_Mutation
chr11	55681757	55681757	-	TGCTGGT	ESCA16T_AF	OR5W2	Frame_Shift_Ins
chr11	55681758	55681758	-	GATCTACTT(	ESCA16T_AF	OR5W2	Frame_Shift_Ins
chr11	55926971	55926971	A	G	ESCA16T_AF	OR8K5	Missense_Mutation
chr11	56185689	56185689	A	G	ESCA16T_AF	OR5R1	Missense_Mutation
chr11	56230746	56230746	G	T	ESCA16T_AF	OR5M9	Silent
chr11	56380263	56380263	G	T	ESCA17T_AF	OR5M1	Missense_Mutation
chr11	57575884	57575884	C	G	ESCA17T_AF	CTNND1	Missense_Mutation
chr11	5776823	5776823	C	T	ESCA16T_AF	OR52N4	Missense_Mutation
chr11	57886045	57886045	-	TGTAGATCA	ESCA16T_AF	OR9I1	Nonsense_Mutation
chr11	57886528	57886528	-	GTGATGGCC	ESCA07T_AF	OR9I1	In_Frame_Ins
chr11	58035053	58035053	G	A	ESCA19T_AF	OR10W1	Missense_Mutation
chr11	5878339	5878339	G	A	ESCA16T_AF	OR52E8	Silent
chr11	5878401	5878401	G	A	ESCA15T_AF	OR52E8	Missense_Mutation
chr11	59224489	59224489	G	A	ESCA20T_AF	OR4D6	Missense_Mutation
chr11	59426347	59426347	G	A	ESCA19T_AF	PATL1	Missense_Mutation
chr11	60642604	60642604	C	T	ESCA16T_AF	ZP1	Nonsense_Mutation
chr11	60666729	60666729	C	T	ESCA19T_AF	PRPF19	Silent
chr11	61898026	61898026	A	G	ESCA16T_AF	INCENP	Missense_Mutation
chr11	6191058	6191058	G	A	ESCA14T_AF	OR52B2	Missense_Mutation
chr11	62396420	62396420	A	G	ESCA16T_AF	GANAB	Silent
chr11	62415278	62415278	T	A	ESCA10T_AF	INTS5	Missense_Mutation
chr11	62415285	62415285	-	CCAA	ESCA10T_AF	INTS5	Frame_Shift_Ins
chr11	6244103	6244103	G	T	ESCA07T_AF	FAM160A2	Missense_Mutation
chr11	6244311	6244311	T	C	ESCA07T_AF	FAM160A2	Missense_Mutation
chr11	62520970	62520970	C	A	ESCA16T_AF	ZBTB3	Missense_Mutation
chr11	62558325	62558325	G	A	ESCA15T_AF	TMEM223	Nonsense_Mutation
chr11	6261701	6261701	-	TGTAGTCAG	ESCA15T_AF	CNGA4	Nonsense_Mutation
chr11	63531245	63531245	G	A	ESCA18T_AF	C11orf95	Missense_Mutation
chr11	64088504	64088504	-	CCAAAGATG	ESCA15T_AF	PRDX5	Frame_Shift_Ins
chr11	64088504	64088504	-	CCAAAGATG	ESCA10T_AF	PRDX5	Frame_Shift_Ins
chr11	64088506	64088506	-	GAATCATCT.	ESCA15T_AF	PRDX5	Nonsense_Mutation
chr11	64674838	64674838	T	G	ESCA14T_AF	ATG2A	Silent
chr11	64897626	64897626	-	TTCAGCAGC	ESCA16T_AF	SYVN1	Frame_Shift_Ins
chr11	65386435	65386435	T	A	ESCA10T_AF	PCNX3	Silent
chr11	65401622	65401622	C	A	ESCA18T_AF	PCNX3	Nonsense_Mutation
chr11	65401644	65401644	C	T	ESCA18T_AF	PCNX3	Silent
chr11	65402018	65402018	C	T	ESCA18T_AF	PCNX3	Nonsense_Mutation
chr11	65402091	65402091	C	T	ESCA18T_AF	PCNX3	Missense_Mutation
chr11	65402913	65402913	C	T	ESCA18T_AF	PCNX3	Silent
chr11	65403089	65403089	C	T	ESCA18T_AF	PCNX3	Silent
chr11	65403207	65403207	C	T	ESCA18T_AF	PCNX3	Missense_Mutation
chr11	65403242	65403242	C	T	ESCA18T_AF	PCNX3	Silent
chr11	65403698	65403698	C	T	ESCA18T_AF	PCNX3	Missense_Mutation
chr11	65403975	65403975	C	A	ESCA18T_AF	PCNX3	Missense_Mutation

chr11	65404413	65404413 C	T	ESCA18T_AF	PCNX3	Silent
chr11	6550180	6550180 T	G	ESCA17T_AF	DNHD1	Missense_Mutation
chr11	65545980	65545980 -	TTTGTGGCA(	ESCA16T_AF	AP5B1	In_Frame_Ins
chr11	65546857	65546857 A	C	ESCA16T_AF	AP5B1	Silent
chr11	65547455	65547455 C	A	ESCA16T_AF	AP5B1	Missense_Mutation
chr11	66204734	66204734 CCTGGGAG(	-	ESCA18T_AF	MRPL11	Frame_Shift_Del
chr11	6632238	6632238 C	T	ESCA16T_AF	TAF10	Missense_Mutation
chr11	66358357	66358357 -	TGGACATGA	ESCA14T_AF	CCDC87	Frame_Shift_Ins
chr11	66358359	66358359 -	CCTGA	ESCA14T_AF	CCDC87	Frame_Shift_Ins
chr11	66411363	66411363 -	GCTGCAGTA	ESCA15T_AF	RBM14-RBM4;R	In_Frame_Ins
chr11	66411375	66411375 -	GCAGCAGCT	ESCA15T_AF	RBM14-RBM4;R	In_Frame_Ins
chr11	6653811	6653811 G	A	ESCA14T_AF	DCHS1	Missense_Mutation
chr11	67067047	67067047 A	C	ESCA16T_AF	ANKRD13D	Missense_Mutation
chr11	67067053	67067053 -	GGGTTTACC	ESCA16T_AF	ANKRD13D	In_Frame_Ins
chr11	67810470	67810470 G	C	ESCA07T_AF	TCIRG1	Missense_Mutation
chr11	67810477	67810477 -	TGGA	ESCA07T_AF	TCIRG1	Frame_Shift_Ins
chr11	67888461	67888461 G	A	ESCA16T_AF	CHKA	Missense_Mutation
chr11	68191088	68191088 G	C	ESCA16T_AF	LRP5	Silent
chr11	68530177	68530177 C	T	ESCA17T_AF	CPT1A	Missense_Mutation
chr11	68665478	68665478 T	C	ESCA16T_AF	MRPL21	Missense_Mutation
chr11	69489997	69489997 -	TGCCGCTAT	ESCA14T_AF	LTO1	In_Frame_Ins
chr11	69625389	69625389 C	T	ESCA16T_AF	FGF3	Missense_Mutation
chr11	70277336	70277336 C	T	ESCA07T_AF	CTTN	Missense_Mutation
chr11	7110363	7110363 G	A	ESCA14T_AF	RBMXL2	Silent
chr11	7110800	7110800 G	A	ESCA15T_AF	RBMXL2	Missense_Mutation
chr11	71819784	71819784 C	T	ESCA15T_AF	LRTOMT	Missense_Mutation
chr11	75439892	75439892 C	T	ESCA19T_AF	MOGAT2	Silent
chr11	76827336	76827336 G	A	ESCA07T_AF	CAPN5	Missense_Mutation
chr11	76858990	76858990 C	T	ESCA16T_AF	MYO7A	Silent
chr11	77340925	77340925 C	T	ESCA01T_AF	CLNS1A	Missense_Mutation
chr11	77727917	77727917 G	A	ESCA18T_AF	KCTD14	Missense_Mutation
chr11	77835193	77835193 A	G	ESCA19T_AF	ALG8	Missense_Mutation
chr11	78468030	78468030 A	G	ESCA01T_AF	TENM4	Missense_Mutation
chr11	794786	794786 -	CACGAGCAT	ESCA16T_AF	SLC25A22	In_Frame_Ins
chr11	82880228	82880228 G	A	ESCA10T_AF	PCF11	Missense_Mutation
chr11	89896163	89896163 T	C	ESCA14T_AF	NAALAD2	Missense_Mutation
chr11	93088565	93088565 G	C	ESCA01T_AF	DEUP1	Missense_Mutation
chr11	93097392	93097392 C	T	ESCA17T_AF	DEUP1	Nonsense_Mutation
chr11	93432956	93432956 AAAC	-	ESCA20T_AF	CEP295	Frame_Shift_Del
chr11	9435934	9435934 -	AAGAGCATA	ESCA07T_AF	IPO7	Frame_Shift_Ins
chr11	95528716	95528716 G	C	ESCA20T_AF	CEP57	Missense_Mutation
chr11	96075054	96075054 -	GGTAAGATG	ESCA14T_AF	MAML2	Frame_Shift_Ins
chr12	100200254	100200254 -	CCACATTCA	ESCA14T_AF	ANKS1B	Nonsense_Mutation
chr12	100441374	100441374 G	T	ESCA14T_AF	UHRF1BP1L	Missense_Mutation
chr12	100676798	100676798 -	ACTAGTGAC	ESCA10T_AF	SCYL2	In_Frame_Ins
chr12	10145860	10145860 C	A	ESCA15T_AF	CLEC1B	Missense_Mutation
chr12	101705864	101705864 A	T	ESCA15T_AF	UTP20	Missense_Mutation
chr12	104118764	104118764 C	T	ESCA16T_AF	STAB2	Silent
chr12	105600935	105600935 G	A	ESCA16T_AF	APPL2	Silent
chr12	106740194	106740194 -	ATTGTGAAT(	ESCA15T_AF	TCP11L2	Nonsense_Mutation
chr12	107975013	107975013 -	GTCGTCGCT	ESCA14T_AF	BTBD11	Frame_Shift_Ins
chr12	108133277	108133277 G	A	ESCA20T_AF	PRDM4	Missense_Mutation
chr12	108985785	108985785 C	T	ESCA16T_AF	TMEM119	Silent
chr12	110206600	110206600 C	T	ESCA14T_AF	FAM222A	Missense_Mutation
chr12	110983861	110983861 -	GCCTGCATT	ESCA16T_AF	PPTC7	Frame_Shift_Ins
chr12	112036797	112036797 C	-	ESCA15T_AF	ATXN2	Frame_Shift_Del
chr12	112036797	112036797 C	-	ESCA17T_AF	ATXN2	Frame_Shift_Del
chr12	112036799	112036799 GC	-	ESCA15T_AF	ATXN2	Frame_Shift_Del

chr12	112036799	112036799	GC	-	ESCA17T_AF	ATXN2	Frame_Shift_Del
chr12	112097099	112097099	G	A	ESCA16T_AF	BRAP	Silent
chr12	112578959	112578959	G	A	ESCA15T_AF	TRAFD1	Missense_Mutation
chr12	112674895	112674895	-	AAGGTGGCT	ESCA07T_AF	HECTD4	Frame_Shift_Ins
chr12	112674899	112674899	C	G	ESCA07T_AF	HECTD4	Missense_Mutation
chr12	112746595	112746595	G	A	ESCA17T_AF	HECTD4	Missense_Mutation
chr12	115109670	115109670	G	T	ESCA14T_AF	TBX3	Missense_Mutation
chr12	119583486	119583486	-	CTC	ESCA16T_AF	SRRM4	In_Frame_Ins
chr12	120661992	120661992	G	A	ESCA07T_AF	PXN	Nonsense_Mutation
chr12	120884328	120884328	C	T	ESCA16T_AF	GATC	Silent
chr12	121017199	121017199	-	ATAT	ESCA14T_AF	POP5	Frame_Shift_Ins
chr12	121098908	121098908	G	T	ESCA16T_AF	CABP1	Missense_Mutation
chr12	121622565	121622565	A	C	ESCA16T_AF	P2RX7	Missense_Mutation
chr12	122277657	122277657	T	C	ESCA01T_AF	HPD	Missense_Mutation
chr12	123200314	123200314	T	C	ESCA07T_AF	HCAR3	Missense_Mutation
chr12	123687482	123687482	-	AGTCCCTCT	ESCA15T_AF	MPHOSPH9	Nonsense_Mutation
chr12	124017850	124017850	G	C	ESCA16T_AF	RILPL1	Silent
chr12	124337801	124337801	C	T	ESCA18T_AF	DNAH10	Silent
chr12	124345707	124345707	-	T	ESCA07T_AF	DNAH10	Frame_Shift_Ins
chr12	124345708	124345708	-	CTCACTTTC	ESCA07T_AF	DNAH10	Frame_Shift_Ins
chr12	124817757	124817757	G	A	ESCA19T_AF	NCOR2	Missense_Mutation
chr12	129181895	129181895	G	A	ESCA07T_AF	TMEM132C	Missense_Mutation
chr12	130184654	130184654	G	T	ESCA20T_AF	TMEM132D	Silent
chr12	130884347	130884347	A	G	ESCA16T_AF	RIMBP2	Silent
chr12	132335719	132335719	C	T	ESCA17T_AF	MMP17	Missense_Mutation
chr12	132502117	132502117	G	A	ESCA19T_AF	EP400	Missense_Mutation
chr12	133202748	133202748	G	A	ESCA19T_AF	POLE	Silent
chr12	13716024	13716024	G	A	ESCA18T_AF	GRIN2B	Missense_Mutation
chr12	15722365	15722365	A	C	ESCA17T_AF	PTPRO	Missense_Mutation
chr12	15807083	15807083	C	A	ESCA01T_AF	EPS8	Missense_Mutation
chr12	16397617	16397617	T	G	ESCA17T_AF	SLC15A5	Missense_Mutation
chr12	1755093	1755093	C	T	ESCA01T_AF	WNT5B	Missense_Mutation
chr12	20806947	20806947	G	T	ESCA15T_AF	PDE3A	Missense_Mutation
chr12	21030836	21030836	C	T	ESCA10T_AF	SLCO1B3;SLCO1	Silent
chr12	25261034	25261034	-	CAGATACTG	ESCA07T_AF	LRMP	Nonsense_Mutation
chr12	266277	266277	A	G	ESCA17T_AF	IQSEC3	Missense_Mutation
chr12	2788662	2788662	G	A	ESCA16T_AF	CACNA1C	Missense_Mutation
chr12	2794961	2794961	A	T	ESCA07T_AF	CACNA1C	Missense_Mutation
chr12	27950909	27950909	C	T	ESCA15T_AF	KLHL42	Missense_Mutation
chr12	32135715	32135715	C	G	ESCA15T_AF	RESF1	Missense_Mutation
chr12	351920	351920	-	AGTTTCCCC	ESCA07T_AF	SLC6A13	Frame_Shift_Ins
chr12	39713776	39713776	C	A	ESCA16T_AF	KIF21A	Missense_Mutation
chr12	39760191	39760191	G	A	ESCA16T_AF	KIF21A	Silent
chr12	40713834	40713834	C	A	ESCA16T_AF	LRRK2	Silent
chr12	40717044	40717044	G	A	ESCA18T_AF	LRRK2	Silent
chr12	40761490	40761490	G	A	ESCA19T_AF	LRRK2	Missense_Mutation
chr12	41422933	41422933	C	T	ESCA14T_AF	CNTN1	Silent
chr12	48535143	48535143	G	A	ESCA10T_AF	PFKM	Missense_Mutation
chr12	48962310	48962310	-	TCCTGGATA	ESCA07T_AF	LALBA	Nonsense_Mutation
chr12	49089603	49089603	T	C	ESCA19T_AF	CCNT1	Missense_Mutation
chr12	49438067	49438067	G	A	ESCA16T_AF	KMT2D	Nonsense_Mutation
chr12	49689009	49689009	G	A	ESCA16T_AF	PRPH	Missense_Mutation
chr12	49916583	49916583	G	A	ESCA07T_AF	SPATS2	Missense_Mutation
chr12	49934914	49934914	C	A	ESCA07T_AF	KCNH3	Missense_Mutation
chr12	49949688	49949688	C	T	ESCA18T_AF	KCNH3	Missense_Mutation
chr12	51882612	51882612	G	A	ESCA15T_AF	SLC4A8	Missense_Mutation
chr12	52585570	52585570	C	T	ESCA16T_AF	KRT80	Silent
chr12	52710714	52710714	C	T	ESCA17T_AF	KRT83	Missense_Mutation

chr12	52911007	52911007	-	CTCCTCATA(	ESCA18T_AF	KRT5	In_Frame_Ins
chr12	53045564	53045564	G	C	ESCA17T_AF	KRT2	Silent
chr12	53457618	53457618	C	T	ESCA16T_AF	TNS2	Silent
chr12	53671005	53671005	G	A	ESCA16T_AF	ESPL1	Missense_Mutation
chr12	53685623	53685623	C	T	ESCA01T_AF	ESPL1	Silent
chr12	53875862	53875862	C	T	ESCA19T_AF	MAP3K12	Missense_Mutation
chr12	54757287	54757287	-	CTCTGCCTC.	ESCA15T_AF	GPR84	Nonsense_Mutation
chr12	54757290	54757290	G	T	ESCA15T_AF	GPR84	Missense_Mutation
chr12	54930828	54930828	A	T	ESCA19T_AF	NCKAP1L	Missense_Mutation
chr12	55863618	55863618	-	A	ESCA17T_AF	OR6C70	Frame_Shift_Ins
chr12	56091059	56091059	A	C	ESCA18T_AF	ITGA7	Missense_Mutation
chr12	56549363	56549363	T	C	ESCA10T_AF	MYL6B	Silent
chr12	56862460	56862460	-	ATAATCCTA(	ESCA15T_AF	SPRYD4	In_Frame_Ins
chr12	56865404	56865404	T	A	ESCA15T_AF	GLS2	Missense_Mutation
chr12	56865407	56865407	-	TCCCCTGGA	ESCA15T_AF	GLS2	In_Frame_Ins
chr12	57115121	57115121	A	C	ESCA16T_AF	NACA	Missense_Mutation
chr12	57178729	57178729	G	T	ESCA16T_AF	HSD17B6	Missense_Mutation
chr12	58109581	58109581	C	T	ESCA07T_AF	OS9	Silent
chr12	58158550	58158550	G	T	ESCA14T_AF	CYP27B1	Missense_Mutation
chr12	6495581	6495581	C	A	ESCA01T_AF	LTBR	Missense_Mutation
chr12	65269191	65269191	-	CTCCGAACA	ESCA16T_AF	TBC1D30	Frame_Shift_Ins
chr12	6672286	6672286	C	T	ESCA17T_AF	NOP2	Silent
chr12	6729956	6729956	G	A	ESCA16T_AF	LPAR5	Silent
chr12	69233264	69233264	A	C	ESCA19T_AF	MDM2	Silent
chr12	70304706	70304706	A	G	ESCA14T_AF	MYRFL	Missense_Mutation
chr12	7053807	7053807	A	T	ESCA18T_AF	C12orf57	Missense_Mutation
chr12	7069323	7069323	C	T	ESCA19T_AF	PTPN6	Missense_Mutation
chr12	71029675	71029675	C	T	ESCA01T_AF	PTPRB	Missense_Mutation
chr12	72027118	72027118	G	C	ESCA14T_AF	ZFC3H1	Missense_Mutation
chr12	72416194	72416194	ATCGAGTTT	-	ESCA10T_AF	TPH2	Frame_Shift_Del
chr12	7360666	7360666	-	TACT	ESCA07T_AF	PEX5	Frame_Shift_Ins
chr12	7360668	7360668	-	CCTCCGCAA	ESCA07T_AF	PEX5	Nonsense_Mutation
chr12	78562583	78562583	C	T	ESCA18T_AF	NAV3	Nonsense_Mutation
chr12	7882210	7882210	C	T	ESCA15T_AF	CLEC4C	Missense_Mutation
chr12	81101538	81101538	TACT	-	ESCA07T_AF	MYF6	Frame_Shift_Del
chr12	81647358	81647358	A	G	ESCA18T_AF	ACSS3	Missense_Mutation
chr12	81991751	81991751	T	C	ESCA15T_AF	PPFIA2	Missense_Mutation
chr12	9994467	9994467	G	T	ESCA15T_AF	KLRF1	Nonsense_Mutation
chr13	100635008	100635008	CCACCA	-	ESCA07T_AF	ZIC2	In_Frame_Del
chr13	103385687	103385687	C	A	ESCA07T_AF	CCDC168	Missense_Mutation
chr13	103703632	103703632	T	C	ESCA18T_AF	SLC10A2	Missense_Mutation
chr13	109540824	109540824	-	AAAAACTTA	ESCA10T_AF	MYO16	In_Frame_Ins
chr13	109753192	109753192	G	A	ESCA18T_AF	MYO16	Missense_Mutation
chr13	111114690	111114690	C	T	ESCA16T_AF	COL4A2	Missense_Mutation
chr13	113173319	113173319	G	A	ESCA20T_AF	TUBGCP3	Silent
chr13	113201924	113201924	C	T	ESCA18T_AF	TUBGCP3	Missense_Mutation
chr13	113914949	113914949	G	C	ESCA15T_AF	CUL4A	Missense_Mutation
chr13	114623809	114623809	G	A	ESCA20T_AF	LINC00452	Missense_Mutation
chr13	115091300	115091300	A	G	ESCA14T_AF	CHAMP1	Silent
chr13	22069445	22069445	C	T	ESCA15T_AF	MICU2	Missense_Mutation
chr13	22255189	22255189	G	T	ESCA01T_AF	FGF9	Nonsense_Mutation
chr13	25671267	25671267	C	T	ESCA07T_AF	PABPC3	Missense_Mutation
chr13	28367021	28367021	C	G	ESCA15T_AF	GSX1	Missense_Mutation
chr13	28895606	28895606	T	-	ESCA18T_AF	FLT1	Frame_Shift_Del
chr13	28964191	28964191	C	T	ESCA15T_AF	FLT1	Missense_Mutation
chr13	31037675	31037675	CT	-	ESCA10T_AF	HMGB1	Frame_Shift_Del
chr13	31722132	31722132	C	T	ESCA15T_AF	HSPH1	Missense_Mutation
chr13	32930628	32930628	G	A	ESCA16T_AF	BRCA2	Missense_Mutation

chr13	39264665	39264665	-	GCTATCAAC	ESCA10T_AF	FREM2	Frame_Shift_Ins
chr13	39264667	39264667	T	G	ESCA10T_AF	FREM2	Silent
chr13	39264690	39264690	T	C	ESCA16T_AF	FREM2	Missense_Mutation
chr13	41650347	41650347	-	CCCCTCCTT(	ESCA15T_AF	WBP4	In_Frame_Ins
chr13	42358025	42358025	G	A	ESCA14T_AF	VWA8	Missense_Mutation
chr13	42358030	42358030	-	TAGGTGAAG	ESCA14T_AF	VWA8	Frame_Shift_Ins
chr13	42742628	42742628	G	A	ESCA15T_AF	DGKH	Missense_Mutation
chr13	45582963	45582963	C	T	ESCA15T_AF	GPALPP1	Silent
chr13	49027169	49027169	G	A	ESCA07T_AF	RB1	Missense_Mutation
chr13	50042039	50042039	C	A	ESCA07T_AF	SETDB2;SETDB2	Missense_Mutation
chr13	50100581	50100581	A	-	ESCA20T_AF	PHF11;SETDB2-I	Frame_Shift_Del
chr13	51658297	51658297	G	A	ESCA16T_AF	C13orf42	Missense_Mutation
chr13	51658356	51658356	G	A	ESCA16T_AF	C13orf42	Silent
chr13	53624342	53624342	C	A	ESCA16T_AF	OLFM4	Silent
chr13	73357859	73357859	G	A	ESCA19T_AF	PIBF1	Silent
chr13	77752019	77752019	C	T	ESCA15T_AF	MYCBP2	Missense_Mutation
chr13	79190079	79190079	-	CACTTCCAT	ESCA15T_AF	OBI1	Nonsense_Mutation
chr13	96624846	96624846	T	C	ESCA18T_AF	UGGT2	Missense_Mutation
chr13	96675328	96675328	C	T	ESCA16T_AF	UGGT2	Missense_Mutation
chr14	100118593	100118593	C	T	ESCA16T_AF	HHIPL1	Silent
chr14	100126062	100126062	C	T	ESCA19T_AF	HHIPL1	Silent
chr14	100615472	100615472	-	CTCCTTCCT	ESCA07T_AF	DEGS2	In_Frame_Ins
chr14	100615473	100615473	-	GGCCTGGG(	ESCA07T_AF	DEGS2	In_Frame_Ins
chr14	104643152	104643152	A	C	ESCA07T_AF	KIF26A	Missense_Mutation
chr14	104643155	104643155	-	CCCTTCTTG(	ESCA07T_AF	KIF26A	In_Frame_Ins
chr14	104643859	104643859	A	G	ESCA16T_AF	KIF26A	Silent
chr14	105930406	105930406	G	A	ESCA16T_AF	MTA1	Missense_Mutation
chr14	105939839	105939839	-	TGTTTGT	ESCA14T_AF	CRIP2	Nonsense_Mutation
chr14	105996005	105996005	G	A	ESCA07T_AF	TMEM121	Silent
chr14	20876253	20876253	A	G	ESCA16T_AF	TEP1	Missense_Mutation
chr14	21424308	21424308	G	A	ESCA18T_AF	RNASE2	Silent
chr14	22038116	22038116	C	G	ESCA16T_AF	OR10G3	Missense_Mutation
chr14	23237353	23237353	-	CCCCACCAA	ESCA15T_AF	OXA1L	Nonsense_Mutation
chr14	23859652	23859652	G	A	ESCA19T_AF	MYH6	Missense_Mutation
chr14	24590647	24590647	C	T	ESCA20T_AF	DCAF11	Silent
chr14	24601513	24601513	A	T	ESCA10T_AF	FITM1	Silent
chr14	24728974	24728974	C	T	ESCA20T_AF	TGM1	Missense_Mutation
chr14	24901504	24901504	G	A	ESCA19T_AF	KHNYN	Missense_Mutation
chr14	29237654	29237654	-	CGGC	ESCA10T_AF	FOXG1	Frame_Shift_Ins
chr14	30135389	30135389	C	G	ESCA19T_AF	PRKD1	Missense_Mutation
chr14	31058628	31058628	G	A	ESCA15T_AF	G2E3	Missense_Mutation
chr14	31598366	31598366	G	A	ESCA16T_AF	HECTD1	Missense_Mutation
chr14	31828244	31828244	-	TGCTATCCT(	ESCA07T_AF	HEATR5A	Frame_Shift_Ins
chr14	31828246	31828246	-	AGAGCTGG(	ESCA07T_AF	HEATR5A	Frame_Shift_Ins
chr14	34145440	34145440	C	G	ESCA15T_AF	NPAS3	Silent
chr14	38679748	38679748	C	T	ESCA15T_AF	SSTR1	Missense_Mutation
chr14	45415111	45415111	-	ATGGACCAC	ESCA16T_AF	KLHL28	Nonsense_Mutation
chr14	50623770	50623770	G	A	ESCA07T_AF	SOS2	Silent
chr14	53360016	53360016	C	T	ESCA14T_AF	FERMT2	Missense_Mutation
chr14	55878404	55878404	C	T	ESCA01T_AF	ATG14	Missense_Mutation
chr14	58605857	58605857	G	A	ESCA19T_AF	ARMH4	Missense_Mutation
chr14	59113434	59113434	G	A	ESCA14T_AF	DACT1	Missense_Mutation
chr14	60004868	60004868	C	T	ESCA14T_AF	CCDC175	Missense_Mutation
chr14	60591388	60591388	C	A	ESCA01T_AF	PCNX4	Silent
chr14	62204915	62204915	C	T	ESCA15T_AF	HIF1A	Missense_Mutation
chr14	62207557	62207557	C	T	ESCA16T_AF	HIF1A	Missense_Mutation
chr14	64882362	64882362	C	G	ESCA14T_AF	MTHFD1	Missense_Mutation
chr14	66082960	66082960	G	A	ESCA17T_AF	FUT8	Silent

chr14	70512940	70512940	G	A	ESCA15T_AF	SLC8A3	Silent
chr14	70925484	70925484	G	C	ESCA07T_AF	ADAM21	Missense_Mutation
chr14	74454658	74454658	C	T	ESCA19T_AF	ENTPD5	Missense_Mutation
chr14	75325122	75325122	A	T	ESCA14T_AF	PROX2	Missense_Mutation
chr14	76620906	76620906	C	G	ESCA16T_AF	GPATCH2L	Nonsense_Mutation
chr14	77493785	77493785	C	T	ESCA16T_AF	IRF2BPL	Silent
chr14	77493831	77493831	GCGGCGGC	-	ESCA14T_AF	IRF2BPL	Frame_Shift_Del
chr14	77735624	77735624	G	A	ESCA18T_AF	NGB	Silent
chr14	80327846	80327846	G	T	ESCA07T_AF	NRXN3	Missense_Mutation
chr14	80971291	80971291	G	A	ESCA07T_AF	CEP128	Missense_Mutation
chr14	90755298	90755298	-	GAAAAGTTT	ESCA07T_AF	NRDE2	Frame_Shift_Ins
chr14	91700432	91700432	C	T	ESCA16T_AF	GPR68	Silent
chr14	92171014	92171014	C	T	ESCA01T_AF	CATSPERB	Silent
chr14	92174532	92174532	C	T	ESCA07T_AF	CATSPERB	Missense_Mutation
chr14	94953738	94953738	C	T	ESCA16T_AF	SERPINA12	Missense_Mutation
chr14	96707356	96707356	G	A	ESCA16T_AF	BDKRB2	Missense_Mutation
chr15	101592033	101592033	A	T	ESCA14T_AF	LRRK1	Missense_Mutation
chr15	23002968	23002968	C	T	ESCA19T_AF	CYFIP1	Silent
chr15	23684975	23684975	C	A	ESCA16T_AF	GOLGA6L2	Nonsense_Mutation
chr15	24921176	24921176	C	T	ESCA14T_AF	NPAP1	Silent
chr15	24922816	24922816	C	A	ESCA18T_AF	NPAP1	Missense_Mutation
chr15	28447322	28447322	C	T	ESCA19T_AF	HERC2	Silent
chr15	29397583	29397583	C	-	ESCA07T_AF	APBA2	Frame_Shift_Del
chr15	30434576	30434576	G	T	ESCA17T_AF	GOLGA8T	Missense_Mutation
chr15	33023216	33023216	A	G	ESCA17T_AF	GREM1	Missense_Mutation
chr15	33922231	33922231	A	G	ESCA07T_AF	RYR3	Missense_Mutation
chr15	35045276	35045276	G	A	ESCA16T_AF	GJD2	Silent
chr15	35273620	35273620	C	T	ESCA16T_AF	ZNF770	Silent
chr15	40573675	40573675	G	T	ESCA16T_AF	ANKRD63	Silent
chr15	40590906	40590906	-	ATCTCCTGT(	ESCA16T_AF	PLCB2	Nonsense_Mutation
chr15	41105029	41105029	-	TCCGTTCCT(	ESCA10T_AF	ZFYVE19	Frame_Shift_Ins
chr15	41803700	41803700	C	T	ESCA15T_AF	LTK	Missense_Mutation
chr15	42618542	42618542	C	A	ESCA17T_AF	GANC	Nonsense_Mutation
chr15	42643529	42643529	T	C	ESCA16T_AF	GANC	Missense_Mutation
chr15	42643538	42643538	A	G	ESCA16T_AF	GANC	Missense_Mutation
chr15	42682180	42682180	T	A	ESCA16T_AF	CAPN3	Silent
chr15	42691798	42691798	C	T	ESCA18T_AF	CAPN3	Silent
chr15	42979271	42979271	C	G	ESCA18T_AF	STARD9	Nonsense_Mutation
chr15	43028271	43028271	-	ACTCTCAGC	ESCA14T_AF	CDAN1	Frame_Shift_Ins
chr15	43501637	43501637	T	C	ESCA16T_AF	EPB42	Missense_Mutation
chr15	43701241	43701241	C	A	ESCA17T_AF	TP53BP1	Silent
chr15	43816004	43816004	-	AGCACCTTC	ESCA15T_AF	MAP1A	In_Frame_Ins
chr15	43893164	43893164	T	G	ESCA14T_AF	STRC	Missense_Mutation
chr15	48063090	48063090	C	A	ESCA07T_AF	SEMA6D	Missense_Mutation
chr15	48451974	48451974	-	TCGTAGGGC	ESCA15T_AF	MYEF2	Frame_Shift_Ins
chr15	48734036	48734036	-	TGTGAT	ESCA15T_AF	FBN1	In_Frame_Ins
chr15	48734038	48734038	-	TTTTAGATA1	ESCA15T_AF	FBN1	In_Frame_Ins
chr15	49301562	49301562	-	CCCAGTGAT	ESCA10T_AF	SECISBP2L	Frame_Shift_Ins
chr15	52075020	52075020	G	A	ESCA10T_AF	TMOD2	Missense_Mutation
chr15	52472062	52472062	C	A	ESCA07T_AF	GNB5	Silent
chr15	55912923	55912923	T	A	ESCA10T_AF	PRTG	Nonsense_Mutation
chr15	59144134	59144134	T	A	ESCA07T_AF	MINDY2	Silent
chr15	59144137	59144137	T	A	ESCA07T_AF	MINDY2	Silent
chr15	62327219	62327219	-	ATTCCTTGG/	ESCA07T_AF	VPS13C	In_Frame_Ins
chr15	65917114	65917114	C	A	ESCA15T_AF	SLC24A1	Silent
chr15	67479784	67479784	A	G	ESCA19T_AF	SMAD3	Missense_Mutation
chr15	67501880	67501880	-	TTTCAG	ESCA07T_AF	AAGAB	In_Frame_Ins
chr15	67501882	67501882	-	AGGAACCA/	ESCA07T_AF	AAGAB	Nonsense_Mutation

chr15	68121586	68121586	G	A	ESCA16T_AF	SKOR1	Missense_Mutation
chr15	72690998	72690998	C	G	ESCA18T_AF	TMEM202	Missense_Mutation
chr15	72954595	72954595	A	G	ESCA10T_AF	GOLGA6B	Missense_Mutation
chr15	74315207	74315207	-	ACT	ESCA10T_AF	PML	In_Frame_Ins
chr15	74315211	74315211	CGC	-	ESCA10T_AF	PML	In_Frame_Del
chr15	74912454	74912454	C	T	ESCA18T_AF	CLK3	Missense_Mutation
chr15	75094359	75094359	G	T	ESCA17T_AF	CSK	Missense_Mutation
chr15	75094782	75094782	C	T	ESCA17T_AF	CSK	Silent
chr15	75122663	75122663	-	GCTGATTGC	ESCA14T_AF	CPLX3	Nonsense_Mutation
chr15	75216102	75216102	C	T	ESCA16T_AF	COX5A	Missense_Mutation
chr15	75932129	75932129	G	T	ESCA16T_AF	IMP3	Silent
chr15	75968558	75968558	C	A	ESCA07T_AF	CSPG4	Missense_Mutation
chr15	77328273	77328273	G	A	ESCA15T_AF	PSTPIP1	Silent
chr15	77407043	77407043	C	G	ESCA17T_AF	PEAK1	Missense_Mutation
chr15	77425765	77425765	C	A	ESCA07T_AF	PEAK1	Missense_Mutation
chr15	78557207	78557207	C	T	ESCA15T_AF	DNAJA4	Silent
chr15	78557211	78557211	-	GTTCTTGTC(	ESCA15T_AF	DNAJA4	Nonsense_Mutation
chr15	78894133	78894133	G	A	ESCA15T_AF	CHRNA3	Missense_Mutation
chr15	78913131	78913131	G	A	ESCA16T_AF	CHRNA3	Silent
chr15	79750780	79750780	C	T	ESCA10T_AF	MINAR1	Missense_Mutation
chr15	79750784	79750784	-	TTACCTCTTT	ESCA10T_AF	MINAR1	Nonsense_Mutation
chr15	81582868	81582868	G	A	ESCA16T_AF	IL16	Silent
chr15	83335621	83335621	-	CGCGAATAT	ESCA07T_AF	AP3B2	Nonsense_Mutation
chr15	83348484	83348484	C	A	ESCA15T_AF	AP3B2	Silent
chr15	85383145	85383145	C	G	ESCA16T_AF	ALPK3	Missense_Mutation
chr15	85400052	85400052	G	A	ESCA17T_AF	ALPK3	Missense_Mutation
chr15	85431087	85431087	G	A	ESCA07T_AF	SLC28A1	Silent
chr15	89386697	89386697	G	A	ESCA16T_AF	ACAN	Nonsense_Mutation
chr15	89402239	89402239	T	C	ESCA16T_AF	ACAN	Silent
chr15	89415295	89415295	C	T	ESCA18T_AF	ACAN	Silent
chr15	89450434	89450434	-	GGTATGCCT	ESCA15T_AF	MFGE8	In_Frame_Ins
chr15	90127608	90127608	-	AAAGGCTCG	ESCA10T_AF	TICRR	Nonsense_Mutation
chr15	90760709	90760709	C	T	ESCA15T_AF	SEMA4B	Missense_Mutation
chr15	90768527	90768527	C	G	ESCA15T_AF	SEMA4B	Silent
chr15	91499986	91499986	G	T	ESCA16T_AF	RCCD1	Missense_Mutation
chr15	91545354	91545354	G	A	ESCA17T_AF	VPS33B	Missense_Mutation
chr15	93545541	93545541	AG	-	ESCA07T_AF	CHD2	Frame_Shift_Del
chr16	10524542	10524542	G	T	ESCA19T_AF	ATF7IP2	Missense_Mutation
chr16	10721468	10721468	C	T	ESCA18T_AF	TEKT5	Missense_Mutation
chr16	10783865	10783865	C	G	ESCA07T_AF	TEKT5	Silent
chr16	11000965	11000965	G	A	ESCA15T_AF	CIITA	Missense_Mutation
chr16	11217640	11217640	G	C	ESCA15T_AF	CLEC16A	Silent
chr16	1398451	1398451	-	GGCTGGC	ESCA10T_AF	BAIAP3	Frame_Shift_Ins
chr16	1398456	1398456	-	ATGGACTCA	ESCA10T_AF	BAIAP3	In_Frame_Ins
chr16	14758759	14758759	G	C	ESCA10T_AF	BFAR	Missense_Mutation
chr16	14819867	14819867	T	C	ESCA16T_AF	NPIPA2;NPIPA3	Missense_Mutation
chr16	1779153	1779153	-	GCATTGTAC	ESCA15T_AF	MAPK8IP3	Frame_Shift_Ins
chr16	1779155	1779155	G	C	ESCA15T_AF	MAPK8IP3	Missense_Mutation
chr16	20693762	20693762	-	CATCTTCATT	ESCA10T_AF	ACSM1	In_Frame_Ins
chr16	20814222	20814222	T	C	ESCA10T_AF	ERI2	Missense_Mutation
chr16	20814226	20814226	-	AGTGTTGCT(	ESCA10T_AF	ERI2	Frame_Shift_Ins
chr16	20975522	20975522	A	G	ESCA16T_AF	DNAH3	Silent
chr16	2103417	2103417	G	A	ESCA16T_AF	TSC2	Silent
chr16	22120874	22120874	G	C	ESCA18T_AF	VWA3A	Missense_Mutation
chr16	23080884	23080884	A	T	ESCA19T_AF	USP31	Missense_Mutation
chr16	2503283	2503283	-	CGCTATGGA	ESCA16T_AF	CCNF	Nonsense_Mutation
chr16	27509063	27509063	C	G	ESCA15T_AF	GTF3C1	Missense_Mutation
chr16	28374074	28374074	G	T	ESCA19T_AF	NPIPB6	Missense_Mutation

chr16	28619848	28619848	G	A	ESCA17T_AF	SULT1A1	Silent
chr16	28834612	28834612	C	T	ESCA15T_AF	ATXN2L	Missense_Mutation
chr16	28895962	28895962	A	T	ESCA16T_AF	ATP2A1	Missense_Mutation
chr16	28895965	28895965	-	AGGATGGAC	ESCA16T_AF	ATP2A1	Frame_Shift_Ins
chr16	29818418	29818418	C	T	ESCA16T_AF	MAZ	Silent
chr16	29907158	29907158	-	AGTTCTGTG	ESCA07T_AF	SEZ6L2	In_Frame_Ins
chr16	30749828	30749828	C	T	ESCA07T_AF	SRCAP	Nonsense_Mutation
chr16	30999253	30999253	C	T	ESCA17T_AF	HSD3B7	Missense_Mutation
chr16	31088723	31088723	-	GTGCA	ESCA16T_AF	ZNF646	Frame_Shift_Ins
chr16	31096433	31096433	-	CTGCTTC	ESCA10T_AF	PRSS53	Frame_Shift_Ins
chr16	31096435	31096435	-	TGGGTGAGT	ESCA10T_AF	PRSS53	Frame_Shift_Ins
chr16	336396	336396	G	A	ESCA19T_AF	PDIA2	Missense_Mutation
chr16	3544586	3544586	C	T	ESCA16T_AF	C16orf90	Missense_Mutation
chr16	3786795	3786795	C	A	ESCA10T_AF	CREBBP	Missense_Mutation
chr16	4042351	4042351	G	C	ESCA07T_AF	ADCY9	Missense_Mutation
chr16	427784	427784	G	A	ESCA16T_AF	TMEM8A	Silent
chr16	4659833	4659833	G	A	ESCA01T_AF	UBALD1	Missense_Mutation
chr16	47143400	47143400	C	A	ESCA19T_AF	NETO2	Missense_Mutation
chr16	4790403	4790403	-	TCATGGCAG	ESCA14T_AF	C16orf71	In_Frame_Ins
chr16	4812716	4812716	-	CGGGATAGC	ESCA10T_AF	ZNF500	Nonsense_Mutation
chr16	49671043	49671043	-	CA	ESCA16T_AF	ZNF423	Frame_Shift_Ins
chr16	49672531	49672531	-	CAAGCACAA	ESCA15T_AF	ZNF423	Nonsense_Mutation
chr16	50324561	50324561	C	T	ESCA16T_AF	ADCY7	Missense_Mutation
chr16	50357520	50357520	C	A	ESCA18T_AF	BRD7	Missense_Mutation
chr16	51173270	51173270	C	T	ESCA16T_AF	SALL1	Missense_Mutation
chr16	51176003	51176003	C	T	ESCA15T_AF	SALL1	Missense_Mutation
chr16	52060278	52060278	TTTTATTTT	-	ESCA16T_AF	C16orf97	Frame_Shift_Del
chr16	52060290	52060290	C	A	ESCA16T_AF	C16orf97	Missense_Mutation
chr16	53504078	53504078	G	A	ESCA17T_AF	RBL2	Silent
chr16	57060350	57060350	G	A	ESCA01T_AF	NLRC5	Missense_Mutation
chr16	58208381	58208381	G	A	ESCA14T_AF	CSNK2A2	Missense_Mutation
chr16	603471	603471	A	G	ESCA16T_AF	CAPN15	Silent
chr16	65038696	65038696	T	C	ESCA14T_AF	CDH11	Missense_Mutation
chr16	67037206	67037206	-	ATAGTGCCC	ESCA07T_AF	CES4A	In_Frame_Ins
chr16	67208309	67208309	T	G	ESCA16T_AF	NOL3	Missense_Mutation
chr16	67208312	67208312	C	A	ESCA16T_AF	NOL3	Silent
chr16	67399234	67399234	C	T	ESCA20T_AF	LRRC36	Missense_Mutation
chr16	67865747	67865747	G	T	ESCA01T_AF	CENPT	Missense_Mutation
chr16	67974092	67974092	GGGCGTGGC	-	ESCA16T_AF	LCAT	In_Frame_Del
chr16	68936398	68936398	-	GAACAGTAA	ESCA16T_AF	TANGO6	Frame_Shift_Ins
chr16	692237	692237	GGCCCCTGT	-	ESCA15T_AF	MCRIP2	Frame_Shift_Del
chr16	75256676	75256676	C	T	ESCA15T_AF	CTRB1	Silent
chr16	774952	774952	-	CTCTCGGGC	ESCA17T_AF	CCDC78	Frame_Shift_Ins
chr16	78056631	78056631	A	G	ESCA01T_AF	CLEC3A	Silent
chr16	81253917	81253917	A	G	ESCA16T_AF	PKD1L2	Missense_Mutation
chr16	836149	836149	A	G	ESCA16T_AF	RPUSD1	Missense_Mutation
chr16	83842917	83842917	T	C	ESCA14T_AF	HSBP1	Silent
chr16	84779248	84779248	T	G	ESCA16T_AF	USP10	Silent
chr16	8857990	8857990	G	A	ESCA20T_AF	ABAT	Missense_Mutation
chr16	8870335	8870335	G	C	ESCA18T_AF	ABAT	Silent
chr16	88945717	88945717	G	A	ESCA16T_AF	CBFA2T3	Silent
chr16	89167644	89167644	-	CCTGCTCTG	ESCA07T_AF	ACSF3	In_Frame_Ins
chr16	89167650	89167650	C	T	ESCA07T_AF	ACSF3	Silent
chr16	89599035	89599035	G	A	ESCA15T_AF	SPG7	Missense_Mutation
chr16	89767484	89767484	C	T	ESCA18T_AF	SPATA2L	Missense_Mutation
chr16	89851319	89851319	G	C	ESCA19T_AF	FANCA	Silent
chr16	89972653	89972653	-	TCAGAGAGC	ESCA14T_AF	TCF25	In_Frame_Ins
chr16	90025612	90025612	C	T	ESCA15T_AF	DEF8	Missense_Mutation

chr16	9857519	9857519	G	A	ESCA15T_AF	GRIN2A	Silent
chr17	10215339	10215339	C	A	ESCA20T_AF	MYH13	Missense_Mutation
chr17	10231289	10231289	C	A	ESCA18T_AF	MYH13	Missense_Mutation
chr17	10366410	10366410	-	AAGAAACCA	ESCA15T_AF	MYH4	In_Frame_Ins
chr17	10366417	10366417	TGGTTTCTT	-	ESCA15T_AF	MYH4	In_Frame_Del
chr17	12898282	12898282	C	A	ESCA19T_AF	ELAC2	Nonsense_Mutation
chr17	1531119	1531119	C	T	ESCA10T_AF	SLC43A2	Missense_Mutation
chr17	1557144	1557144	C	T	ESCA18T_AF	PRPF8	Missense_Mutation
chr17	15960939	15960939	C	A	ESCA07T_AF	NCOR1	Missense_Mutation
chr17	1675235	1675235	C	T	ESCA17T_AF	SERPINF1	Missense_Mutation
chr17	18002355	18002355	C	A	ESCA15T_AF	DRG2	Missense_Mutation
chr17	18023083	18023083	G	A	ESCA16T_AF	MYO15A	Silent
chr17	18023897	18023897	G	A	ESCA16T_AF	MYO15A	Missense_Mutation
chr17	18150489	18150489	CAG	-	ESCA16T_AF	FLII	In_Frame_Del
chr17	19287954	19287954	G	A	ESCA19T_AF	MFAP4	Nonsense_Mutation
chr17	19618447	19618447	C	T	ESCA16T_AF	SLC47A2	Silent
chr17	19643672	19643672	C	T	ESCA16T_AF	ALDH3A1	Missense_Mutation
chr17	20914617	20914617	-	CTCCACTGG	ESCA14T_AF	USP22	Frame_Shift_Ins
chr17	21101740	21101740	-	GCACCACCC	ESCA16T_AF	TMEM11	Frame_Shift_Ins
chr17	26093610	26093610	G	A	ESCA15T_AF	NOS2	Silent
chr17	26711558	26711558	C	A	ESCA19T_AF	SARM1	Silent
chr17	26882077	26882077	A	C	ESCA15T_AF	PIGS	Missense_Mutation
chr17	27308719	27308719	-	CGGTACCCA	ESCA14T_AF	SEZ6	In_Frame_Ins
chr17	27613208	27613208	-	A	ESCA17T_AF	NUFIP2	Frame_Shift_Ins
chr17	27613209	27613209	-	TTGAAGATG	ESCA17T_AF	NUFIP2	Frame_Shift_Ins
chr17	27908768	27908768	C	T	ESCA19T_AF	GIT1	Missense_Mutation
chr17	27943312	27943312	C	G	ESCA18T_AF	CORO6	Missense_Mutation
chr17	29111446	29111446	-	GGAAAAGAA	ESCA07T_AF	CRLF3	Frame_Shift_Ins
chr17	29192731	29192731	C	G	ESCA01T_AF	ATAD5	Missense_Mutation
chr17	29855661	29855661	G	A	ESCA16T_AF	RAB11FIP4	Silent
chr17	30376284	30376284	G	A	ESCA15T_AF	LRRC37B	Silent
chr17	33585712	33585712	G	A	ESCA18T_AF	SLFN5	Translation_Start_Site
chr17	33690017	33690017	C	T	ESCA15T_AF	SLFN11	Silent
chr17	34398330	34398330	C	T	ESCA18T_AF	CCL18	Missense_Mutation
chr17	34746950	34746950	G	A	ESCA10T_AF	TBC1D3F;TBC1D	Silent
chr17	35300080	35300080	C	A	ESCA19T_AF	LHX1	Silent
chr17	3567141	3567141	C	A	ESCA07T_AF	TAX1BP3	Missense_Mutation
chr17	3567145	3567145	-	CACACACGA	ESCA07T_AF	TAX1BP3	Nonsense_Mutation
chr17	36099546	36099546	G	A	ESCA19T_AF	HNF1B	Silent
chr17	36339725	36339725	C	T	ESCA17T_AF	TBC1D3;TBC1D3	Missense_Mutation
chr17	36718351	36718351	C	T	ESCA14T_AF	SRCIN1	Missense_Mutation
chr17	37453380	37453380	G	A	ESCA16T_AF	FBXL20	Missense_Mutation
chr17	37618889	37618889	G	A	ESCA14T_AF	CDK12	Missense_Mutation
chr17	37792104	37792104	G	A	ESCA15T_AF	PPP1R1B	Missense_Mutation
chr17	3788841	3788841	C	A	ESCA18T_AF	CAMKK1	Silent
chr17	38249569	38249569	C	T	ESCA19T_AF	THRA	Silent
chr17	40004205	40004205	T	G	ESCA18T_AF	KLHL10	Silent
chr17	40065265	40065265	G	T	ESCA10T_AF	ACLY	Missense_Mutation
chr17	40260072	40260072	C	A	ESCA18T_AF	DHX58	Nonsense_Mutation
chr17	40318554	40318554	-	TACTGCGTG	ESCA14T_AF	KCNH4	In_Frame_Ins
chr17	40716088	40716088	G	A	ESCA15T_AF	COASY	Silent
chr17	40831861	40831861	G	A	ESCA19T_AF	CCR10	Silent
chr17	40913287	40913287	CGCTCCGGC	-	ESCA17T_AF	RAMP2	In_Frame_Del
chr17	40913287	40913287	CGCTCCGGC	-	ESCA18T_AF	RAMP2	In_Frame_Del
chr17	40970418	40970418	G	T	ESCA16T_AF	BECN1	Silent
chr17	41228533	41228533	T	C	ESCA07T_AF	BRCA1	Missense_Mutation
chr17	42338066	42338066	G	A	ESCA01T_AF	SLC4A1	Missense_Mutation
chr17	42433371	42433371	-	TACTGGGT	ESCA16T_AF	FAM171A2	Frame_Shift_Ins

chr17	42477252	42477252	G	A	ESCA18T_AF	GPATCH8	Silent
chr17	43003787	43003787	A	C	ESCA07T_AF	KIF18B	Missense_Mutation
chr17	43333141	43333141	G	A	ESCA16T_AF	SPATA32	Silent
chr17	4693054	4693054	G	A	ESCA16T_AF	GLTPD2	Silent
chr17	47388699	47388699	G	A	ESCA14T_AF	ZNF652	Silent
chr17	48917795	48917795	G	A	ESCA19T_AF	WFIKKN2	Silent
chr17	5404055	5404055	C	T	ESCA15T_AF	LOC728392	Silent
chr17	54535327	54535327	C	A	ESCA18T_AF	ANKFN1	Missense_Mutation
chr17	54671846	54671846	C	A	ESCA18T_AF	NOG	Missense_Mutation
chr17	58134634	58134634	G	A	ESCA19T_AF	HEATR6	Silent
chr17	58740839	58740839	C	T	ESCA20T_AF	PPM1D	Missense_Mutation
chr17	59485577	59485577	C	G	ESCA17T_AF	TBX2	Missense_Mutation
chr17	60683508	60683508	G	A	ESCA19T_AF	TLK2	Missense_Mutation
chr17	61562305	61562305	-	TGTTG	ESCA10T_AF	ACE	Frame_Shift_Ins
chr17	61904017	61904017	-	TTTGCGC	ESCA14T_AF	FTSJ3	Frame_Shift_Ins
chr17	61904018	61904018	-	CTTGGTTGT	ESCA14T_AF	FTSJ3	Nonsense_Mutation
chr17	61950575	61950575	G	A	ESCA15T_AF	CSH2	Silent
chr17	61995460	61995460	-	GAAGCCTAT	ESCA15T_AF	GH1	Frame_Shift_Ins
chr17	62226423	62226423	-	GGATGATGT	ESCA16T_AF	TEX2	In_Frame_Ins
chr17	62226424	62226424	C	T	ESCA16T_AF	TEX2	Missense_Mutation
chr17	6337383	6337383	-	ATGAAATGT	ESCA10T_AF	AIPL1;AIPL1	Frame_Shift_Ins
chr17	636408	636408	G	A	ESCA17T_AF	TLCD3A	Missense_Mutation
chr17	648299	648299	G	A	ESCA15T_AF	GEMIN4	Missense_Mutation
chr17	65908191	65908191	G	C	ESCA01T_AF	BPTF	Missense_Mutation
chr17	66303842	66303842	G	A	ESCA01T_AF	ARSG	Missense_Mutation
chr17	67119462	67119462	C	A	ESCA15T_AF	ABCA6	Nonsense_Mutation
chr17	70845851	70845851	T	C	ESCA16T_AF	SLC39A11	Missense_Mutation
chr17	71398099	71398099	-	GCACCCATG	ESCA16T_AF	SDK2	Frame_Shift_Ins
chr17	72436606	72436606	C	G	ESCA16T_AF	GPRC5C	Missense_Mutation
chr17	73273512	73273512	G	A	ESCA19T_AF	SLC25A19	Silent
chr17	7329082	7329082	C	T	ESCA20T_AF	SPEM2	Silent
chr17	73654514	73654514	T	C	ESCA19T_AF	RECQL5	Missense_Mutation
chr17	73686842	73686842	A	T	ESCA14T_AF	SAP30BP	Missense_Mutation
chr17	73686844	73686844	-	CTTATCATG	ESCA14T_AF	SAP30BP	Nonsense_Mutation
chr17	73759481	73759481	G	C	ESCA18T_AF	GALK1	Nonsense_Mutation
chr17	73761120	73761120	G	A	ESCA18T_AF	GALK1	Missense_Mutation
chr17	74099457	74099457	C	T	ESCA16T_AF	EXOC7	Silent
chr17	74382394	74382394	G	A	ESCA15T_AF	SPHK1	Missense_Mutation
chr17	7466636	7466636	G	A	ESCA07T_AF	SENP3	Silent
chr17	75489140	75489140	A	T	ESCA16T_AF	SEPTIN9	Nonsense_Mutation
chr17	7574003	7574003	G	A	ESCA16T_AF	TP53	Nonsense_Mutation
chr17	7577580	7577580	T	C	ESCA07T_AF	TP53	Missense_Mutation
chr17	7578406	7578406	C	T	ESCA18T_AF	TP53	Missense_Mutation
chr17	7578441	7578441	G	T	ESCA20T_AF	TP53	Nonsense_Mutation
chr17	7578475	7578475	G	-	ESCA15T_AF	TP53	Frame_Shift_Del
chr17	7578496	7578496	A	G	ESCA17T_AF	TP53	Missense_Mutation
chr17	7578553	7578553	T	C	ESCA10T_AF	TP53	Missense_Mutation
chr17	7579315	7579315	G	T	ESCA10T_AF	TP53	Nonsense_Mutation
chr17	7579358	7579358	C	A	ESCA10T_AF	TP53	Missense_Mutation
chr17	76803180	76803180	C	T	ESCA16T_AF	USP36	Missense_Mutation
chr17	76989782	76989782	G	A	ESCA16T_AF	CANT1	Silent
chr17	77100178	77100178	-	TTTAGACGT	ESCA14T_AF	RBFOX3	Nonsense_Mutation
chr17	78092141	78092141	-	TTCCTGGCC	ESCA16T_AF	GAA	Frame_Shift_Ins
chr17	7830685	7830685	G	A	ESCA15T_AF	KCNAB3	Silent
chr17	78337566	78337566	C	G	ESCA15T_AF	RNF213	Missense_Mutation
chr17	78867516	78867516	C	T	ESCA16T_AF	RPTOR	Missense_Mutation
chr17	78921118	78921118	G	A	ESCA20T_AF	RPTOR	Missense_Mutation
chr17	7907066	7907066	G	T	ESCA19T_AF	GUCY2D	Missense_Mutation

chr17	79220094	79220094	G	A	ESCA15T_AF	SLC38A10	Silent
chr17	79513944	79513944	C	T	ESCA15T_AF	FAAP100	Missense_Mutation
chr17	80400324	80400324	-	CGTATTGCC	ESCA07T_AF	HEXD	In_Frame_Ins
chr17	8046843	8046843	G	A	ESCA16T_AF	PER1	Missense_Mutation
chr17	8383573	8383573	G	A	ESCA17T_AF	MYH10	Missense_Mutation
chr17	8700683	8700683	C	A	ESCA16T_AF	MFSD6L	Missense_Mutation
chr17	9515788	9515788	C	T	ESCA16T_AF	CFAP52	Silent
chr17	9515792	9515792	-	TCTTCTACT(	ESCA16T_AF	CFAP52	Frame_Shift_Ins
chr18	13826410	13826410	G	A	ESCA15T_AF	MC5R	Missense_Mutation
chr18	22805572	22805572	A	C	ESCA15T_AF	ZNF521	Missense_Mutation
chr18	23637600	23637600	C	G	ESCA14T_AF	SS18	Missense_Mutation
chr18	24128223	24128223	TCCTCCTCC	-	ESCA10T_AF	KCTD1	In_Frame_Del
chr18	29522644	29522644	-	GCTTTGTCG(	ESCA16T_AF	TRAPPC8	In_Frame_Ins
chr18	31318840	31318840	A	G	ESCA20T_AF	ASXL3	Missense_Mutation
chr18	32834186	32834186	T	G	ESCA16T_AF	ZSCAN30	Missense_Mutation
chr18	33795659	33795659	G	A	ESCA17T_AF	MOCOS	Missense_Mutation
chr18	43418765	43418765	G	A	ESCA07T_AF	SIGLEC15	Silent
chr18	43568717	43568717	T	C	ESCA16T_AF	PSTPIP2	Missense_Mutation
chr18	44089662	44089662	C	T	ESCA20T_AF	LOXHD1	Missense_Mutation
chr18	46889585	46889585	A	T	ESCA17T_AF	DYM	Missense_Mutation
chr18	51013185	51013185	C	T	ESCA19T_AF	DCC	Missense_Mutation
chr18	55238772	55238772	A	C	ESCA19T_AF	FECH	Missense_Mutation
chr18	56651335	56651335	C	T	ESCA17T_AF	ZNF532	Silent
chr18	56823038	56823038	G	A	ESCA16T_AF	SEC11C	Missense_Mutation
chr18	58039471	58039471	C	T	ESCA15T_AF	MC4R	Missense_Mutation
chr18	60587331	60587331	-	AGA	ESCA15T_AF	PHLPP1	In_Frame_Ins
chr18	60587335	60587335	-	AAACAAGA(	ESCA15T_AF	PHLPP1	Frame_Shift_Ins
chr18	60645764	60645764	-	TCCTCAGTG.	ESCA14T_AF	PHLPP1	Frame_Shift_Ins
chr18	64221720	64221720	G	C	ESCA17T_AF	CDH19	Missense_Mutation
chr18	645094	645094	-	CAGGCACT	ESCA16T_AF	CLUL1	Frame_Shift_Ins
chr18	645097	645097	-	CTTACCAGG	ESCA16T_AF	CLUL1	In_Frame_Ins
chr18	67344984	67344984	G	T	ESCA17T_AF	DOK6	Nonsense_Mutation
chr18	690631	690631	T	G	ESCA07T_AF	ENOSF1	Missense_Mutation
chr18	6958503	6958503	C	T	ESCA20T_AF	LAMA1	Missense_Mutation
chr18	72344045	72344045	-	GGAAGTAA(	ESCA15T_AF	ZNF407	In_Frame_Ins
chr18	72592993	72592993	-	GAGAAGTC(	ESCA16T_AF	ZNF407	Nonsense_Mutation
chr18	72998037	72998037	C	T	ESCA01T_AF	TSHZ1	Silent
chr18	77893683	77893683	A	G	ESCA16T_AF	ADNP2	Silent
chr19	10116473	10116473	-	CACAGATG(	ESCA14T_AF	COL5A3	Frame_Shift_Ins
chr19	1014250	1014250	T	C	ESCA17T_AF	TMEM259	Missense_Mutation
chr19	10334812	10334812	-	CCTCCTTCT(	ESCA15T_AF	S1PR2	Frame_Shift_Ins
chr19	10334813	10334813	-	CTATGCCT	ESCA15T_AF	S1PR2	Frame_Shift_Ins
chr19	1045202	1045202	G	C	ESCA16T_AF	ABCA7	Missense_Mutation
chr19	10472202	10472202	C	A	ESCA19T_AF	TYK2	Missense_Mutation
chr19	1049396	1049396	G	A	ESCA14T_AF	ABCA7	Missense_Mutation
chr19	10655496	10655496	C	T	ESCA15T_AF	ATG4D	Silent
chr19	11040302	11040302	-	CCTTCTTCT(	ESCA07T_AF	TIMM29	Frame_Shift_Ins
chr19	11513363	11513363	G	A	ESCA16T_AF	RGL3	Missense_Mutation
chr19	11534697	11534697	G	C	ESCA07T_AF	CCDC151	Missense_Mutation
chr19	11624857	11624857	C	T	ESCA19T_AF	ECSIT	Silent
chr19	12222912	12222912	C	T	ESCA16T_AF	ZNF788P	Nonsense_Mutation
chr19	12882099	12882099	G	A	ESCA19T_AF	HOOK2	Missense_Mutation
chr19	13365957	13365957	G	A	ESCA18T_AF	CACNA1A	Silent
chr19	13428042	13428042	C	T	ESCA19T_AF	CACNA1A	Missense_Mutation
chr19	1371110	1371110	G	A	ESCA16T_AF	PWWP3A	Silent
chr19	14034553	14034553	G	C	ESCA07T_AF	CC2D1A	Silent
chr19	14048699	14048699	G	C	ESCA14T_AF	PODNL1	Missense_Mutation
chr19	14069945	14069945	G	T	ESCA07T_AF	DCAF15	Silent

chr19	14070217	14070217	C	G	ESCA16T_AF	DCAF15	Missense_Mutation
chr19	1453139	1453139	A	T	ESCA16T_AF	APC2	Nonsense_Mutation
chr19	1453141	1453141	-	GCCCCCACC	ESCA16T_AF	APC2	In_Frame_Ins
chr19	1506192	1506192	C	T	ESCA19T_AF	ADAMTSL5	Missense_Mutation
chr19	16345055	16345055	-	CCTGGGGCC	ESCA10T_AF	AP1M1	Nonsense_Mutation
chr19	17393484	17393484	C	-	ESCA14T_AF	ANKLE1	Frame_Shift_Del
chr19	17397438	17397438	A	T	ESCA15T_AF	ANKLE1	Missense_Mutation
chr19	17397441	17397441	-	GAAACCAGA	ESCA15T_AF	ANKLE1	Nonsense_Mutation
chr19	17534419	17534419	C	G	ESCA07T_AF	MVB12A	Missense_Mutation
chr19	18092671	18092671	G	A	ESCA18T_AF	KCNN1	Missense_Mutation
chr19	18368390	18368390	-	GGGAGTGG(	ESCA07T_AF	IQCN	Frame_Shift_Ins
chr19	18794593	18794593	C	G	ESCA16T_AF	CRTC1	Silent
chr19	1881418	1881418	C	T	ESCA14T_AF	ABHD17A	Missense_Mutation
chr19	18899427	18899427	C	G	ESCA17T_AF	COMP	Missense_Mutation
chr19	19625474	19625474	T	A	ESCA17T_AF	TSSK6	Missense_Mutation
chr19	19654859	19654859	T	A	ESCA16T_AF	CILP2	Missense_Mutation
chr19	19681102	19681102	G	A	ESCA18T_AF	PBX4	Missense_Mutation
chr19	19746469	19746469	-	GATGACTTT(	ESCA16T_AF	GMIP	Frame_Shift_Ins
chr19	2235980	2235980	-	CCTGAGTGC	ESCA07T_AF	PLEKHJ1	Frame_Shift_Ins
chr19	22496518	22496518	C	G	ESCA16T_AF	ZNF729	Missense_Mutation
chr19	2279090	2279090	C	A	ESCA17T_AF	PEAK3	Silent
chr19	24009993	24009993	G	T	ESCA16T_AF	RPSAP58	Missense_Mutation
chr19	2808281	2808281	-	CCGGCTGCC	ESCA15T_AF	THOP1	Frame_Shift_Ins
chr19	30164911	30164911	C	T	ESCA17T_AF	PLEKHF1	Silent
chr19	30165570	30165570	C	T	ESCA17T_AF	PLEKHF1	Missense_Mutation
chr19	307151	307151	C	T	ESCA19T_AF	MIER2	Silent
chr19	31039604	31039604	C	A	ESCA16T_AF	ZNF536	Silent
chr19	32843816	32843816	-	CAATTTCCA	ESCA15T_AF	ZNF507	Nonsense_Mutation
chr19	33289296	33289296	G	C	ESCA07T_AF	TDRD12	Silent
chr19	33289301	33289301	T	A	ESCA07T_AF	TDRD12	Missense_Mutation
chr19	33467576	33467576	G	A	ESCA16T_AF	FAAP24	Silent
chr19	34868742	34868742	G	A	ESCA19T_AF	GPI	Missense_Mutation
chr19	35843106	35843106	C	A	ESCA10T_AF	FFAR1	Silent
chr19	36159368	36159368	T	G	ESCA16T_AF	UPK1A	Missense_Mutation
chr19	36166788	36166788	C	T	ESCA18T_AF	UPK1A	Missense_Mutation
chr19	36205852	36205852	A	G	ESCA17T_AF	ZBTB32	Silent
chr19	36352918	36352918	-	CA	ESCA07T_AF	KIRREL2	Frame_Shift_Ins
chr19	37117412	37117412	G	T	ESCA15T_AF	ZNF382	Missense_Mutation
chr19	37680582	37680582	C	A	ESCA19T_AF	ZNF585B	Missense_Mutation
chr19	38692616	38692616	C	A	ESCA19T_AF	SIPA1L3	Missense_Mutation
chr19	38976466	38976466	G	A	ESCA01T_AF	RYR1	Missense_Mutation
chr19	39025414	39025414	-	G	ESCA17T_AF	RYR1	Frame_Shift_Ins
chr19	3907815	3907815	G	C	ESCA15T_AF	ATCAY	Missense_Mutation
chr19	39361379	39361379	G	A	ESCA15T_AF	RINL	Silent
chr19	39907564	39907564	T	G	ESCA18T_AF	PLEKHG2	Missense_Mutation
chr19	39964155	39964155	C	T	ESCA14T_AF	SUPT5H	Missense_Mutation
chr19	40321375	40321375	G	A	ESCA18T_AF	DYRK1B	Missense_Mutation
chr19	40363020	40363020	G	A	ESCA16T_AF	FCGBP	Missense_Mutation
chr19	40424229	40424229	G	A	ESCA07T_AF	FCGBP	Silent
chr19	40504297	40504297	C	G	ESCA15T_AF	ZNF546	Missense_Mutation
chr19	41249853	41249853	C	T	ESCA16T_AF	C19orf54	Missense_Mutation
chr19	41627946	41627946	C	G	ESCA18T_AF	CYP2F1	Missense_Mutation
chr19	41758809	41758809	T	G	ESCA16T_AF	AXL	Silent
chr19	42383652	42383652	C	T	ESCA17T_AF	CD79A	Nonsense_Mutation
chr19	42795037	42795037	G	A	ESCA19T_AF	CIC	Missense_Mutation
chr19	42818660	42818660	G	A	ESCA19T_AF	TMEM145	Missense_Mutation
chr19	43763039	43763039	G	A	ESCA17T_AF	PSG9	Missense_Mutation
chr19	4422768	4422768	G	A	ESCA19T_AF	CHAF1A	Missense_Mutation

chr19	44570384	44570384 G	A	ESCA20T_AF	ZNF223	Missense_Mutation
chr19	44933212	44933212 G	A	ESCA19T_AF	ZNF229	Missense_Mutation
chr19	45411165	45411165 -	CTCCTCCTG(	ESCA10T_AF	APOE	Frame_Shift_Ins
chr19	45579386	45579386 G	A	ESCA15T_AF	ZNF296	Silent
chr19	45920089	45920089 G	C	ESCA16T_AF	ERCC1	Missense_Mutation
chr19	45924634	45924634 T	C	ESCA10T_AF	ERCC1	Silent
chr19	46268840	46268840 C	A	ESCA16T_AF	SIX5	Missense_Mutation
chr19	46269685	46269685 -	CTGCAGCTG	ESCA07T_AF	SIX5	Nonsense_Mutation
chr19	46347382	46347382 G	A	ESCA15T_AF	SYMPK	Silent
chr19	46394581	46394581 T	C	ESCA07T_AF	MYPOP	Missense_Mutation
chr19	46807301	46807301 G	A	ESCA14T_AF	HIF3A	Missense_Mutation
chr19	47164150	47164150 C	T	ESCA17T_AF	DACT3	Missense_Mutation
chr19	48537607	48537607 C	T	ESCA15T_AF	CABP5	Missense_Mutation
chr19	48537610	48537610 -	CAGTTTGAC.	ESCA15T_AF	CABP5	Nonsense_Mutation
chr19	49621257	49621257 -	ACAGCATCA	ESCA10T_AF	LIN7B	Nonsense_Mutation
chr19	49636584	49636584 G	A	ESCA16T_AF	PPFIA3	Missense_Mutation
chr19	49901333	49901333 G	T	ESCA17T_AF	CCDC155	Nonsense_Mutation
chr19	50098867	50098867 C	T	ESCA16T_AF	PRR12	Silent
chr19	50100751	50100751 C	G	ESCA20T_AF	PRR12	Silent
chr19	50105131	50105131 C	A	ESCA20T_AF	PRR12	Silent
chr19	50166689	50166689 G	A	ESCA20T_AF	IRF3	Missense_Mutation
chr19	50200722	50200722 -	GA	ESCA10T_AF	CPT1C	Frame_Shift_Ins
chr19	5033007	5033007 G	A	ESCA20T_AF	KDM4B	Missense_Mutation
chr19	50832220	50832220 C	T	ESCA10T_AF	KCNC3	Silent
chr19	50979859	50979859 G	A	ESCA18T_AF	EMC10	Missense_Mutation
chr19	51140553	51140553 C	T	ESCA16T_AF	SYT3	Missense_Mutation
chr19	51228322	51228322 C	A	ESCA07T_AF	CLEC11A	Silent
chr19	51411714	51411714 C	T	ESCA19T_AF	KLK4	Silent
chr19	51841403	51841403 C	T	ESCA15T_AF	VSIG10L	Missense_Mutation
chr19	52725409	52725409 C	G	ESCA18T_AF	PPP2R1A	Missense_Mutation
chr19	54304587	54304587 G	T	ESCA20T_AF	NLRP12	Missense_Mutation
chr19	54418631	54418631 C	T	ESCA07T_AF	CACNG7	Missense_Mutation
chr19	54486092	54486092 A	G	ESCA16T_AF	CACNG8	Missense_Mutation
chr19	54486095	54486095 -	CCCTACACA	ESCA16T_AF	CACNG8	Frame_Shift_Ins
chr19	54631527	54631527 C	T	ESCA17T_AF	PRPF31	Missense_Mutation
chr19	54866901	54866901 C	T	ESCA01T_AF	LAIR1	Silent
chr19	55175795	55175795 G	A	ESCA16T_AF	LILRB4	Missense_Mutation
chr19	55378162	55378162 G	T	ESCA16T_AF	KIR3DL2	Missense_Mutation
chr19	55441995	55441995 A	G	ESCA16T_AF	NLRP7	Silent
chr19	55743452	55743452 G	A	ESCA18T_AF	PPP6R1	Nonsense_Mutation
chr19	55824328	55824328 C	T	ESCA01T_AF	TMEM150B	Missense_Mutation
chr19	56090169	56090169 G	A	ESCA17T_AF	ZNF579	Silent
chr19	56104408	56104408 C	T	ESCA07T_AF	FIZ1	Missense_Mutation
chr19	56154166	56154166 G	T	ESCA20T_AF	ZNF580	Missense_Mutation
chr19	56313063	56313063 G	C	ESCA10T_AF	NLRP11	Silent
chr19	56569766	56569766 -	CCAATTATC	ESCA16T_AF	NLRP5	Nonsense_Mutation
chr19	56599405	56599405 C	G	ESCA16T_AF	ZNF787	Missense_Mutation
chr19	5664070	5664070 G	A	ESCA18T_AF	SAFB	Missense_Mutation
chr19	57641799	57641799 G	A	ESCA16T_AF	USP29	Missense_Mutation
chr19	57744876	57744876 G	C	ESCA16T_AF	AURKC	Missense_Mutation
chr19	5784637	5784637 GCTGCAGG(	-	ESCA14T_AF	PRR22	Frame_Shift_Del
chr19	58189571	58189571 G	A	ESCA15T_AF	ZSCAN4	Silent
chr19	58232214	58232214 GAACAAGT(	-	ESCA14T_AF	ZNF671	In_Frame_Del
chr19	582572	582572 TCCTGAGG(	-	ESCA10T_AF	BSG	Frame_Shift_Del
chr19	58352702	58352702 G	A	ESCA20T_AF	ZNF587B	Missense_Mutation
chr19	58578415	58578415 -	TGTGTGGGG	ESCA15T_AF	ZNF135	Frame_Shift_Ins
chr19	58601603	58601603 -	A	ESCA15T_AF	ZSCAN18	Frame_Shift_Ins
chr19	58601604	58601604 -	AAGGCGTTT	ESCA15T_AF	ZSCAN18	Frame_Shift_Ins

chr19	58861875	58861875	G	T	ESCA14T_AF	A1BG	Missense_Mutation
chr19	58904550	58904550	G	A	ESCA15T_AF	RPS5	Missense_Mutation
chr19	5923907	5923907	C	T	ESCA14T_AF	RANBP3	Missense_Mutation
chr19	6040165	6040165	C	T	ESCA15T_AF	RFX2	Silent
chr19	6430156	6430156	G	T	ESCA14T_AF	SLC25A41	Missense_Mutation
chr19	7685438	7685438	GGT	-	ESCA10T_AF	XAB2	In_Frame_Del
chr19	7685441	7685441	-	CATCT	ESCA10T_AF	XAB2	Frame_Shift_Ins
chr19	7982975	7982975	C	A	ESCA15T_AF	TGFBR3L	Nonsense_Mutation
chr19	8138069	8138069	G	A	ESCA17T_AF	FBN3	Silent
chr19	8182432	8182432	-	TGTGTCCCC.	ESCA07T_AF	FBN3	Nonsense_Mutation
chr19	8183875	8183875	G	C	ESCA16T_AF	FBN3	Silent
chr19	8186262	8186262	C	A	ESCA14T_AF	FBN3	Nonsense_Mutation
chr19	8321946	8321946	T	C	ESCA16T_AF	CERS4	Silent
chr19	8429516	8429516	-	CCCTGGGGA	ESCA10T_AF	ANGPTL4	In_Frame_Ins
chr19	8429521	8429521	-	AGGCTGTGA	ESCA10T_AF	ANGPTL4	In_Frame_Ins
chr19	8592294	8592294	G	A	ESCA20T_AF	MYO1F	Missense_Mutation
chr19	8670087	8670087	G	A	ESCA17T_AF	ADAMTS10	Missense_Mutation
chr19	8807988	8807988	C	T	ESCA07T_AF	ACTL9	Missense_Mutation
chr19	9058333	9058333	G	C	ESCA14T_AF	MUC16	Missense_Mutation
chr19	9077207	9077207	T	C	ESCA15T_AF	MUC16	Silent
chr19	9324863	9324863	G	A	ESCA16T_AF	OR7D4	Silent
chr19	984533	984533	T	C	ESCA16T_AF	WDR18	Silent
chr19	984554	984554	C	G	ESCA16T_AF	WDR18	Silent
chr2	100900795	100900795	G	T	ESCA19T_AF	LONRF2	Missense_Mutation
chr2	103095320	103095320	G	T	ESCA14T_AF	SLC9A4	Silent
chr2	103274107	103274107	T	C	ESCA07T_AF	SLC9A2	Missense_Mutation
chr2	109746039	109746039	G	A	ESCA16T_AF	SH3RF3	Missense_Mutation
chr2	110301812	110301812	C	G	ESCA14T_AF	SEPTIN10	Missense_Mutation
chr2	112620132	112620132	-	TCTTTTTCAG	ESCA10T_AF	ANAPC1	Frame_Shift_Ins
chr2	113082009	113082009	TCTT	-	ESCA19T_AF	ZC3H6	Nonsense_Mutation
chr2	120231084	120231084	G	A	ESCA14T_AF	SCTR	Missense_Mutation
chr2	121106850	121106850	C	T	ESCA16T_AF	INHBB	Silent
chr2	122516298	122516298	G	T	ESCA01T_AF	TSN	Missense_Mutation
chr2	128346071	128346071	G	A	ESCA16T_AF	MYO7B	Missense_Mutation
chr2	128747338	128747338	G	A	ESCA15T_AF	SAP130	Missense_Mutation
chr2	128757719	128757719	-	CCCTCACAG	ESCA15T_AF	SAP130	Nonsense_Mutation
chr2	131520596	131520596	G	A	ESCA19T_AF	AMER3	Silent
chr2	1320143	1320143	C	T	ESCA16T_AF	SNTG2	Missense_Mutation
chr2	133541660	133541660	C	A	ESCA01T_AF	NCKAP5	Missense_Mutation
chr2	135744739	135744739	C	T	ESCA01T_AF	MAP3K19	Missense_Mutation
chr2	136575199	136575199	G	T	ESCA16T_AF	LCT	Silent
chr2	136624260	136624260	C	-	ESCA20T_AF	MCM6	Frame_Shift_Del
chr2	138400161	138400161	C	A	ESCA16T_AF	THSD7B	Nonsense_Mutation
chr2	141083400	141083400	C	A	ESCA07T_AF	LRP1B	Nonsense_Mutation
chr2	141242964	141242964	C	T	ESCA18T_AF	LRP1B	Missense_Mutation
chr2	143676221	143676221	C	T	ESCA17T_AF	KYNU	Silent
chr2	145147321	145147321	C	T	ESCA15T_AF	ZEB2	Silent
chr2	153519683	153519683	-	TAACTATTA	ESCA10T_AF	PRPF40A	Nonsense_Mutation
chr2	15432830	15432830	C	A	ESCA16T_AF	NBAS	Missense_Mutation
chr2	160229618	160229618	C	T	ESCA07T_AF	BAZ2B	Missense_Mutation
chr2	163256769	163256769	G	A	ESCA18T_AF	KCNH7	Silent
chr2	163302704	163302704	T	A	ESCA20T_AF	KCNH7	Missense_Mutation
chr2	164467423	164467423	C	T	ESCA16T_AF	FIGN	Missense_Mutation
chr2	1652940	1652940	C	G	ESCA10T_AF	PXDN	Missense_Mutation
chr2	1664685	1664685	G	A	ESCA18T_AF	PXDN	Missense_Mutation
chr2	166532999	166532999	G	A	ESCA07T_AF	CSRNP3	Missense_Mutation
chr2	166758417	166758417	G	T	ESCA16T_AF	TTC21B	Silent
chr2	167140968	167140968	C	G	ESCA16T_AF	SCN9A	Missense_Mutation

chr2	169792742	169792742 G	A	ESCA15T_AF	ABCB11	Nonsense_Mutation
chr2	171649402	171649402 C	T	ESCA16T_AF	ERICH2	Silent
chr2	172803242	172803242 G	C	ESCA16T_AF	HAT1	Missense_Mutation
chr2	174820819	174820819 G	A	ESCA15T_AF	SP3	Missense_Mutation
chr2	175618298	175618298 G	A	ESCA16T_AF	CHRNA1	Silent
chr2	176957676	176957676 G	A	ESCA15T_AF	HOXD13	Missense_Mutation
chr2	179510754	179510754 G	T	ESCA15T_AF	TTN	Missense_Mutation
chr2	179586641	179586641 A	G	ESCA18T_AF	TTN	Silent
chr2	179600637	179600637 C	T	ESCA16T_AF	TTN	Missense_Mutation
chr2	179638970	179638970 C	A	ESCA19T_AF	TTN	Missense_Mutation
chr2	179640529	179640529 C	T	ESCA10T_AF	TTN	Missense_Mutation
chr2	179641416	179641416 C	T	ESCA07T_AF	TTN	Silent
chr2	179647291	179647291 C	G	ESCA15T_AF	TTN	Missense_Mutation
chr2	183848070	183848070 C	T	ESCA16T_AF	NCKAP1	Missense_Mutation
chr2	183943834	183943834 A	G	ESCA16T_AF	DUSP19	Missense_Mutation
chr2	185802563	185802563 C	T	ESCA19T_AF	ZNF804A	Nonsense_Mutation
chr2	186664503	186664503 C	T	ESCA19T_AF	FSIP2	Silent
chr2	186678275	186678275 G	A	ESCA10T_AF	FSIP2	Missense_Mutation
chr2	190339046	190339046 C	T	ESCA15T_AF	WDR75	Silent
chr2	196642627	196642627 G	A	ESCA15T_AF	DNAH7	Missense_Mutation
chr2	197511211	197511211 GG	-	ESCA07T_AF	CCDC150	Frame_Shift_Del
chr2	202519636	202519636 G	C	ESCA16T_AF	MPP4	Silent
chr2	202901047	202901047 C	A	ESCA10T_AF	FZD7	Silent
chr2	203980761	203980761 C	T	ESCA19T_AF	NBEAL1	Missense_Mutation
chr2	204305633	204305633 G	A	ESCA14T_AF	RAPH1	Silent
chr2	204305968	204305968 GTGGGGGA	-	ESCA10T_AF	RAPH1	In_Frame_Del
chr2	206994831	206994831 A	C	ESCA17T_AF	NDUFS1	Missense_Mutation
chr2	207509046	207509046 C	A	ESCA17T_AF	FAM237A	Missense_Mutation
chr2	208611806	208611806 C	T	ESCA19T_AF	CCNYL1	Silent
chr2	20870588	20870588 C	T	ESCA15T_AF	GDF7	Silent
chr2	20870715	20870715 C	G	ESCA15T_AF	GDF7	Missense_Mutation
chr2	208841964	208841964 A	G	ESCA16T_AF	PLEKHM3	Silent
chr2	210574906	210574906 G	A	ESCA16T_AF	MAP2	Silent
chr2	21252549	21252549 C	A	ESCA16T_AF	APOB	Missense_Mutation
chr2	21258576	21258576 C	T	ESCA17T_AF	APOB	Missense_Mutation
chr2	219894296	219894296 C	T	ESCA07T_AF	CFAP65	Silent
chr2	220166431	220166431 C	T	ESCA14T_AF	PTPRN	Silent
chr2	220423995	220423995 C	A	ESCA10T_AF	OBSL1	Missense_Mutation
chr2	223781214	223781214 C	T	ESCA19T_AF	ACSL3	Missense_Mutation
chr2	228882210	228882210 C	T	ESCA16T_AF	SPHKAP	Silent
chr2	230724181	230724181 G	A	ESCA15T_AF	TRIP12	Nonsense_Mutation
chr2	233198649	233198649 C	T	ESCA07T_AF	DIS3L2	Missense_Mutation
chr2	233681584	233681584 G	C	ESCA15T_AF	GIGYF2	Missense_Mutation
chr2	233708804	233708804 CAA	-	ESCA10T_AF	GIGYF2	In_Frame_Del
chr2	234202917	234202917 C	T	ESCA16T_AF	ATG16L1	Missense_Mutation
chr2	234713706	234713706 T	C	ESCA16T_AF	MROH2A	Silent
chr2	234858672	234858672 G	A	ESCA07T_AF	TRPM8	Missense_Mutation
chr2	234891827	234891827 C	A	ESCA10T_AF	TRPM8	Missense_Mutation
chr2	238253065	238253065 C	T	ESCA16T_AF	COL6A3	Silent
chr2	238672453	238672453 T	A	ESCA16T_AF	LRRFIP1	Silent
chr2	239039078	239039078 G	A	ESCA16T_AF	ESPNL	Missense_Mutation
chr2	24108620	24108620 G	A	ESCA17T_AF	ATAD2B	Nonsense_Mutation
chr2	241439491	241439491 C	G	ESCA19T_AF	ANKMY1	Missense_Mutation
chr2	26410190	26410190 C	T	ESCA19T_AF	GAREM2	Silent
chr2	26533613	26533613 C	G	ESCA15T_AF	ADGRF3	Missense_Mutation
chr2	26702515	26702515 -	TCTGCTCCA	ESCA14T_AF	OTOF	Nonsense_Mutation
chr2	27672588	27672588 G	A	ESCA07T_AF	IFT172	Missense_Mutation
chr2	29221101	29221101 -	TGAGGCAG	ESCA14T_AF	TOGARAM2	Frame_Shift_Ins

chr2	29543696	29543696	C	T	ESCA17T_AF	ALK	Nonsense_Mutation
chr2	32475725	32475725	G	A	ESCA20T_AF	NLRC4	Missense_Mutation
chr2	32725176	32725176	-	GTAAGATTA	ESCA14T_AF	BIRC6	Nonsense_Mutation
chr2	33359972	33359972	C	T	ESCA15T_AF	LTBP1	Silent
chr2	37456067	37456067	T	C	ESCA14T_AF	CEBPZ	Missense_Mutation
chr2	39156961	39156961	-	TTAAAGCCA	ESCA15T_AF	ARHGEF33	Nonsense_Mutation
chr2	39893150	39893150	C	T	ESCA15T_AF	TMEM178A	Silent
chr2	43452095	43452095	G	A	ESCA10T_AF	ZFP36L2	Missense_Mutation
chr2	44508662	44508662	C	A	ESCA17T_AF	SLC3A1	Silent
chr2	49216145	49216145	G	A	ESCA15T_AF	FSHR	Silent
chr2	50765424	50765424	C	T	ESCA16T_AF	NRXN1	Missense_Mutation
chr2	53995149	53995149	C	A	ESCA01T_AF	CHAC2	Silent
chr2	55126916	55126916	-	AAGGTACAT	ESCA16T_AF	EML6	Nonsense_Mutation
chr2	55186394	55186394	A	C	ESCA16T_AF	EML6	Silent
chr2	55252645	55252645	-	AGATGAAAC	ESCA10T_AF	RTN4	In_Frame_Ins
chr2	55747013	55747013	T	G	ESCA17T_AF	CFAP36	Missense_Mutation
chr2	58275986	58275986	A	T	ESCA19T_AF	VRK2	Missense_Mutation
chr2	61430349	61430349	T	C	ESCA14T_AF	USP34	Missense_Mutation
chr2	61484079	61484079	C	T	ESCA17T_AF	USP34	Missense_Mutation
chr2	61542016	61542016	T	C	ESCA14T_AF	USP34	Missense_Mutation
chr2	62115610	62115610	C	T	ESCA15T_AF	CCT4	Silent
chr2	69053238	69053238	G	A	ESCA15T_AF	ARHGAP25	Missense_Mutation
chr2	7005307	7005307	T	G	ESCA07T_AF	CMPK2	Missense_Mutation
chr2	7005311	7005311	-	GCTGTGG	ESCA07T_AF	CMPK2	Frame_Shift_Ins
chr2	70742032	70742032	G	C	ESCA14T_AF	TGFA	Missense_Mutation
chr2	70742038	70742038	-	GGTATTGTG	ESCA14T_AF	TGFA	In_Frame_Ins
chr2	72406482	72406482	G	T	ESCA14T_AF	EXOC6B	Silent
chr2	74327620	74327620	G	A	ESCA07T_AF	TET3	Silent
chr2	75100484	75100484	-	CCCCACTCA	ESCA10T_AF	HK2	In_Frame_Ins
chr2	75100488	75100488	A	G	ESCA10T_AF	HK2	Missense_Mutation
chr2	80831257	80831257	G	A	ESCA19T_AF	CTNNA2	Missense_Mutation
chr2	88998051	88998051	T	G	ESCA17T_AF	RPIA	Missense_Mutation
chr2	9347243	9347243	C	T	ESCA14T_AF	ASAP2	Nonsense_Mutation
chr2	9520865	9520865	TTTAGCAAA	-	ESCA18T_AF	ASAP2	Frame_Shift_Del
chr2	97359420	97359420	-	ACCCAGAAC	ESCA16T_AF	FER1L5	Frame_Shift_Ins
chr2	98377264	98377264	T	C	ESCA15T_AF	TMEM131	Missense_Mutation
chr2	98409408	98409408	C	T	ESCA18T_AF	TMEM131	Silent
chr2	98914394	98914394	G	A	ESCA19T_AF	VWA3B	Missense_Mutation
chr2	98986519	98986519	C	T	ESCA16T_AF	CNGA3	Silent
chr2	99013162	99013162	G	T	ESCA16T_AF	CNGA3	Missense_Mutation
chr2	99438756	99438756	C	T	ESCA07T_AF	KIAA1211L	Silent
chr20	11904033	11904033	C	T	ESCA17T_AF	BTBD3	Missense_Mutation
chr20	16362385	16362385	A	T	ESCA17T_AF	KIF16B	Missense_Mutation
chr20	17446134	17446134	G	T	ESCA18T_AF	PCSK2	Missense_Mutation
chr20	17492680	17492680	G	A	ESCA15T_AF	BFSP1	Silent
chr20	17716416	17716416	C	G	ESCA16T_AF	BANF2	Missense_Mutation
chr20	18296076	18296076	G	A	ESCA16T_AF	ZNF133	Missense_Mutation
chr20	18365673	18365673	C	T	ESCA16T_AF	DZANK1	Missense_Mutation
chr20	20033297	20033297	C	T	ESCA15T_AF	CRNKL1	Missense_Mutation
chr20	20493617	20493617	T	C	ESCA14T_AF	RALGAPA2	Missense_Mutation
chr20	30227804	30227804	G	A	ESCA19T_AF	COX4I2	Missense_Mutation
chr20	3128170	3128170	-	TA	ESCA01T_AF	FASTKD5	Frame_Shift_Ins
chr20	3204191	3204191	-	GAAG	ESCA16T_AF	ITPA	In_Frame_Ins
chr20	32664620	32664620	-	GATGGCAAC	ESCA19T_AF	RALY	In_Frame_Ins
chr20	33030038	33030038	G	A	ESCA18T_AF	ITCH	Missense_Mutation
chr20	34241031	34241031	-	GGGATTGGT	ESCA10T_AF	RBM12	In_Frame_Ins
chr20	37357084	37357084	C	T	ESCA17T_AF	SLC32A1	Silent
chr20	37630462	37630462	G	A	ESCA17T_AF	DHX35	Silent

chr20	39991664	39991664	C	T	ESCA18T_AF	EMILIN3	Missense_Mutation
chr20	42169446	42169446	C	G	ESCA15T_AF	L3MBTL1	Missense_Mutation
chr20	42835597	42835597	T	C	ESCA14T_AF	OSER1	Translation_Start_Site
chr20	43141505	43141505	A	G	ESCA15T_AF	SERINC3	Missense_Mutation
chr20	44505724	44505724	-	AGTTCTTCAT	ESCA16T_AF	ZSWIM3	Frame_Shift_Ins
chr20	44574851	44574851	G	A	ESCA18T_AF	PCIF1	Missense_Mutation
chr20	47648594	47648594	G	A	ESCA07T_AF	ARFGEF2	Missense_Mutation
chr20	48807949	48807949	G	C	ESCA14T_AF	CEBPB	Missense_Mutation
chr20	50701101	50701101	G	T	ESCA14T_AF	ZFP64	Missense_Mutation
chr20	53205033	53205033	C	G	ESCA17T_AF	DOK5	Silent
chr20	54961395	54961395	C	A	ESCA15T_AF	AURKA	Missense_Mutation
chr20	5556620	5556620	-	CTTCCTGAA	ESCA07T_AF	GPCPD1	Nonsense_Mutation
chr20	55777560	55777560	C	T	ESCA19T_AF	BMP7	Missense_Mutation
chr20	57088939	57088939	C	A	ESCA16T_AF	APCDD1L	Missense_Mutation
chr20	57485432	57485432	G	A	ESCA19T_AF	GNAS	Silent
chr20	57766235	57766235	T	A	ESCA07T_AF	ZNF831	Missense_Mutation
chr20	57767708	57767708	G	A	ESCA19T_AF	ZNF831	Missense_Mutation
chr20	60714241	60714241	-	AGCGTTATA	ESCA16T_AF	PSMA7	Frame_Shift_Ins
chr20	60887236	60887236	G	T	ESCA20T_AF	LAMA5	Missense_Mutation
chr20	61941159	61941159	G	C	ESCA15T_AF	COL20A1	Silent
chr20	61981896	61981896	C	A	ESCA17T_AF	CHRNA4	Silent
chr20	62126185	62126185	A	G	ESCA16T_AF	EEF1A2	Silent
chr20	62319016	62319016	C	T	ESCA17T_AF	RTEL1	Silent
chr20	62323172	62323172	C	T	ESCA17T_AF	RTEL1	Silent
chr20	62597666	62597666	C	T	ESCA16T_AF	ZNF512B	Missense_Mutation
chr20	62597694	62597694	T	G	ESCA16T_AF	ZNF512B	Silent
chr20	8721037	8721037	G	T	ESCA10T_AF	PLCB1	Silent
chr21	22782597	22782597	C	T	ESCA01T_AF	NCAM2	Missense_Mutation
chr21	31744019	31744019	G	T	ESCA20T_AF	KRTAP13-2	Nonsense_Mutation
chr21	40781989	40781989	C	T	ESCA17T_AF	LCA5L	Missense_Mutation
chr21	43279149	43279149	G	A	ESCA20T_AF	PRDM15	Missense_Mutation
chr21	43414003	43414003	-	TTATTCACA	ESCA14T_AF	ZBTB21	Nonsense_Mutation
chr21	47422179	47422179	A	G	ESCA18T_AF	COL6A1	Missense_Mutation
chr21	47533971	47533971	GCTACCGTC	-	ESCA10T_AF	COL6A2	Frame_Shift_Del
chr21	47546055	47546055	G	A	ESCA14T_AF	COL6A2	Missense_Mutation
chr21	47705184	47705184	G	C	ESCA01T_AF	MCM3AP	Missense_Mutation
chr21	47957193	47957193	G	A	ESCA10T_AF	DIP2A	Missense_Mutation
chr22	17447245	17447245	T	C	ESCA07T_AF	GAB4	Missense_Mutation
chr22	17447245	17447245	T	C	ESCA15T_AF	GAB4	Missense_Mutation
chr22	17447253	17447253	G	A	ESCA07T_AF	GAB4	Missense_Mutation
chr22	17447253	17447253	G	A	ESCA10T_AF	GAB4	Missense_Mutation
chr22	17447253	17447253	G	A	ESCA15T_AF	GAB4	Missense_Mutation
chr22	18905901	18905901	G	A	ESCA07T_AF	LOC102724788;I	Missense_Mutation
chr22	19373161	19373161	G	C	ESCA20T_AF	HIRA	Nonsense_Mutation
chr22	20051110	20051110	C	T	ESCA19T_AF	TANGO2	Silent
chr22	20109830	20109830	G	C	ESCA15T_AF	RANBP1	Missense_Mutation
chr22	20458697	20458697	C	T	ESCA19T_AF	RIMBP3	Missense_Mutation
chr22	24179250	24179250	C	G	ESCA16T_AF	DERL3;DERL3	Silent
chr22	24580225	24580225	-	C	ESCA15T_AF	SUSD2	Frame_Shift_Ins
chr22	24580229	24580229	-	CAGTTCGAT	ESCA15T_AF	SUSD2	Nonsense_Mutation
chr22	24584279	24584279	C	T	ESCA07T_AF	SUSD2	Missense_Mutation
chr22	24919587	24919587	C	A	ESCA16T_AF	UPB1	Missense_Mutation
chr22	25750759	25750759	C	T	ESCA20T_AF	LRP5L	Silent
chr22	26286839	26286839	C	T	ESCA19T_AF	MYO18B	Silent
chr22	26422561	26422561	C	T	ESCA16T_AF	MYO18B	Silent
chr22	26688711	26688711	C	T	ESCA19T_AF	SEZ6L	Missense_Mutation
chr22	26688897	26688897	C	T	ESCA15T_AF	SEZ6L	Missense_Mutation
chr22	27012140	27012140	G	T	ESCA10T_AF	CRYBB1	Missense_Mutation

chr22	29075628	29075628	C	T	ESCA19T_AF	TTC28	Silent
chr22	29650213	29650213	C	T	ESCA01T_AF	EMID1	Missense_Mutation
chr22	30735238	30735238	G	-	ESCA18T_AF	SF3A1	Frame_Shift_Del
chr22	30768183	30768183	C	T	ESCA20T_AF	CCDC157	Missense_Mutation
chr22	30887596	30887596	C	T	ESCA07T_AF	SEC14L4	Missense_Mutation
chr22	30891412	30891412	G	A	ESCA16T_AF	SEC14L4	Silent
chr22	30983376	30983376	C	T	ESCA16T_AF	PES1	Missense_Mutation
chr22	31487303	31487303	G	T	ESCA14T_AF	SMTN	Missense_Mutation
chr22	31621804	31621804	C	T	ESCA18T_AF	LIMK2	Missense_Mutation
chr22	33255168	33255168	T	A	ESCA17T_AF	TIMP3	Missense_Mutation
chr22	37471234	37471234	T	C	ESCA07T_AF	TMPRSS6	Missense_Mutation
chr22	37578379	37578379	-	GAGCCAGA(	ESCA10T_AF	C1QTNF6	Frame_Shift_Ins
chr22	37964602	37964602	C	T	ESCA19T_AF	CDC42EP1	Silent
chr22	38111939	38111939	-	CTCACCCTC	ESCA16T_AF	TRIOBP	Frame_Shift_Ins
chr22	38155226	38155226	G	A	ESCA18T_AF	TRIOBP	Silent
chr22	39883877	39883877	C	T	ESCA20T_AF	MGAT3	Silent
chr22	40069035	40069035	G	T	ESCA20T_AF	CACNA1I	Silent
chr22	41620226	41620226	G	A	ESCA14T_AF	L3MBTL2	Missense_Mutation
chr22	41895860	41895860	G	T	ESCA16T_AF	ACO2	Missense_Mutation
chr22	42293159	42293159	G	A	ESCA16T_AF	SREBF2	Missense_Mutation
chr22	42306445	42306445	C	T	ESCA16T_AF	SHISA8	Missense_Mutation
chr22	42606744	42606744	G	A	ESCA17T_AF	TCF20	Missense_Mutation
chr22	42910760	42910760	A	G	ESCA16T_AF	RRP7A	Silent
chr22	45182463	45182463	G	T	ESCA17T_AF	ARHGAP8;PRR5	Missense_Mutation
chr22	46773152	46773152	C	G	ESCA07T_AF	CELSR1	Missense_Mutation
chr22	46773155	46773155	-	ATAGAACGG	ESCA07T_AF	CELSR1	Frame_Shift_Ins
chr22	47064627	47064627	G	C	ESCA16T_AF	GRAMD4	Missense_Mutation
chr22	50181522	50181522	G	A	ESCA15T_AF	BRD1	Silent
chr22	50356693	50356693	T	C	ESCA16T_AF	PIM3	Missense_Mutation
chr22	50471777	50471777	G	A	ESCA18T_AF	TTLL8	Silent
chr22	50609372	50609372	-	TGCGAAGTT	ESCA14T_AF	PANX2	Frame_Shift_Ins
chr22	50615649	50615649	G	A	ESCA17T_AF	PANX2	Missense_Mutation
chr22	50695537	50695537	C	T	ESCA17T_AF	MAPK12	Missense_Mutation
chr22	50703923	50703923	-	CTTCC	ESCA10T_AF	MAPK11	Frame_Shift_Ins
chr22	50724312	50724312	ACTGG	-	ESCA07T_AF	PLXNB2	Frame_Shift_Del
chr22	50724328	50724328	-	CCAGT	ESCA07T_AF	PLXNB2	Frame_Shift_Ins
chr22	50893999	50893999	-	ATCCACAAG	ESCA14T_AF	SBF1	Frame_Shift_Ins
chr22	50944176	50944176	-	AGCACCAG(	ESCA16T_AF	LMF2	In_Frame_Ins
chr22	50969216	50969216	G	A	ESCA15T_AF	ODF3B	Silent
chr22	50987105	50987105	-	CCTCCTCCT(	ESCA15T_AF	KLHDC7B	Nonsense_Mutation
chr22	51169557	51169557	C	T	ESCA15T_AF	SHANK3	Silent
chr3	10012329	10012329	A	T	ESCA18T_AF	EMC3	Missense_Mutation
chr3	101284288	101284288	G	A	ESCA20T_AF	TRMT10C	Silent
chr3	108149754	108149754	C	T	ESCA15T_AF	MYH15	Silent
chr3	111426999	111426999	C	T	ESCA07T_AF	PLCXD2	Silent
chr3	113172645	113172645	-	CTCAGAGAT	ESCA10T_AF	SPICE1	In_Frame_Ins
chr3	113866319	113866319	C	T	ESCA18T_AF	DRD3	Missense_Mutation
chr3	115395026	115395026	C	G	ESCA01T_AF	GAP43	Missense_Mutation
chr3	118647547	118647547	C	T	ESCA15T_AF	IGSF11	Missense_Mutation
chr3	118647550	118647550	-	GTATCAGGG	ESCA15T_AF	IGSF11	In_Frame_Ins
chr3	119325773	119325773	T	C	ESCA16T_AF	PLA1A	Missense_Mutation
chr3	121634097	121634097	C	T	ESCA16T_AF	SLC15A2	Silent
chr3	122271480	122271480	C	T	ESCA19T_AF	PARP9	Missense_Mutation
chr3	123452835	123452835	C	T	ESCA16T_AF	MYLK	Silent
chr3	124951804	124951804	G	C	ESCA19T_AF	ZNF148	Nonsense_Mutation
chr3	126220774	126220774	A	G	ESCA17T_AF	UROC1	Missense_Mutation
chr3	127540626	127540626	C	T	ESCA19T_AF	MGLL	Silent
chr3	128720741	128720741	G	A	ESCA16T_AF	EFCC1	Silent

chr3	129278474	129278474	C	T	ESCA16T_AF	PLXND1	Nonsense_Mutation
chr3	129284288	129284288	G	A	ESCA19T_AF	PLXND1	Silent
chr3	129370589	129370589	-	GCTGCATCT	ESCA10T_AF	TMCC1	Frame_Shift_Ins
chr3	130122462	130122462	G	T	ESCA16T_AF	COL6A5	Missense_Mutation
chr3	136085892	136085892	C	A	ESCA15T_AF	STAG1	Nonsense_Mutation
chr3	13612020	13612020	T	C	ESCA16T_AF	FBLN2	Silent
chr3	136664719	136664719	C	T	ESCA15T_AF	NCK1	Missense_Mutation
chr3	138474748	138474748	C	G	ESCA15T_AF	PIK3CB	Missense_Mutation
chr3	1424718	1424718	G	A	ESCA16T_AF	CNTN6	Silent
chr3	142521181	142521181	C	T	ESCA16T_AF	TRPC1	Silent
chr3	146239358	146239358	G	A	ESCA17T_AF	PLSCR1	Silent
chr3	147128649	147128649	C	T	ESCA17T_AF	ZIC1	Silent
chr3	14798916	14798916	C	T	ESCA16T_AF	C3orf20	Missense_Mutation
chr3	148601516	148601516	G	T	ESCA16T_AF	CPA3	Missense_Mutation
chr3	149688584	149688584	G	A	ESCA19T_AF	PFN2	Silent
chr3	150588874	150588874	G	T	ESCA10T_AF	MINDY4B	Missense_Mutation
chr3	151105665	151105665	C	A	ESCA16T_AF	MED12L	Missense_Mutation
chr3	15115372	15115372	G	A	ESCA10T_AF	RBSN	Missense_Mutation
chr3	15507937	15507937	T	C	ESCA07T_AF	COLQ	Missense_Mutation
chr3	15507944	15507944	CCT	AGG	ESCA07T_AF	COLQ	Nonsense_Mutation
chr3	155198899	155198899	A	T	ESCA17T_AF	PLCH1	Missense_Mutation
chr3	156241622	156241622	C	T	ESCA18T_AF	KCNAB1	Nonsense_Mutation
chr3	156763198	156763198	C	G	ESCA16T_AF	LEKR1	Missense_Mutation
chr3	160395571	160395571	G	A	ESCA07T_AF	ARL14	Missense_Mutation
chr3	161214876	161214876	C	A	ESCA19T_AF	OTOL1	Missense_Mutation
chr3	164741402	164741402	G	A	ESCA15T_AF	SI	Missense_Mutation
chr3	164750424	164750424	T	C	ESCA19T_AF	SI	Silent
chr3	169514040	169514040	G	A	ESCA16T_AF	LRRC34	Silent
chr3	169866975	169866975	T	G	ESCA15T_AF	PHC3	Missense_Mutation
chr3	173993223	173993223	C	A	ESCA15T_AF	NLGN1	Silent
chr3	178936096	178936096	G	T	ESCA20T_AF	PIK3CA	Missense_Mutation
chr3	180379735	180379735	G	C	ESCA15T_AF	CCDC39	Missense_Mutation
chr3	180688110	180688110	G	A	ESCA15T_AF	FXR1	Missense_Mutation
chr3	183896901	183896901	-	ACAAAAT	ESCA15T_AF	AP2M1	Frame_Shift_Ins
chr3	183907679	183907679	G	C	ESCA16T_AF	ABCF3	Missense_Mutation
chr3	183959320	183959320	C	T	ESCA18T_AF	VWA5B2	Missense_Mutation
chr3	185769856	185769856	G	A	ESCA10T_AF	ETV5	Missense_Mutation
chr3	192126001	192126001	C	T	ESCA15T_AF	FGF12	Silent
chr3	192516848	192516848	-	GGAAGAGG	ESCA10T_AF	MB21D2	Nonsense_Mutation
chr3	192994551	192994551	A	G	ESCA18T_AF	ATP13A5	Silent
chr3	194175025	194175025	T	C	ESCA16T_AF	ATP13A3	Missense_Mutation
chr3	195974322	195974322	G	A	ESCA15T_AF	PCYT1A	Silent
chr3	196434681	196434681	C	G	ESCA14T_AF	CEP19	Missense_Mutation
chr3	197421306	197421306	G	A	ESCA15T_AF	RUBCN	Missense_Mutation
chr3	197751573	197751573	G	A	ESCA17T_AF	LMLN	Missense_Mutation
chr3	21462767	21462767	A	C	ESCA18T_AF	ZNF385D	Missense_Mutation
chr3	21467028	21467028	C	G	ESCA20T_AF	ZNF385D	Missense_Mutation
chr3	26751493	26751493	A	T	ESCA16T_AF	LRRC3B	Silent
chr3	32859647	32859647	CTC	-	ESCA07T_AF	TRIM71	In_Frame_Del
chr3	38739797	38739797	G	A	ESCA15T_AF	SCN10A	Silent
chr3	38739818	38739818	A	T	ESCA10T_AF	SCN10A	Missense_Mutation
chr3	40528664	40528664	G	T	ESCA01T_AF	ZNF619	Silent
chr3	42233088	42233088	G	A	ESCA07T_AF	TRAK1	Silent
chr3	43122459	43122459	G	A	ESCA19T_AF	POMGNT2	Silent
chr3	44905764	44905764	C	T	ESCA19T_AF	TMEM42	Missense_Mutation
chr3	45152224	45152224	G	A	ESCA16T_AF	CDCP1	Silent
chr3	46450070	46450070	T	A	ESCA16T_AF	CCRL2	Missense_Mutation
chr3	47043622	47043622	G	A	ESCA16T_AF	NBEAL2	Silent

chr3	4712413	4712413	G	A	ESCA16T_AF	ITPR1	Silent
chr3	48462281	48462281	-	TCGCCAAAA	ESCA15T_AF	PLXNB1	Frame_Shift_Ins
chr3	48624723	48624723	C	A	ESCA16T_AF	COL7A1	Missense_Mutation
chr3	48716375	48716375	G	T	ESCA15T_AF	NCKIPSD	Missense_Mutation
chr3	48732363	48732363	T	C	ESCA14T_AF	IP6K2	Missense_Mutation
chr3	49008034	49008034	T	A	ESCA16T_AF	ARIH2	Missense_Mutation
chr3	49008038	49008038	-	TGGGGCAGT	ESCA16T_AF	ARIH2	Frame_Shift_Ins
chr3	49843529	49843529	C	T	ESCA07T_AF	UBA7	Missense_Mutation
chr3	49933743	49933743	C	T	ESCA18T_AF	MST1R	Missense_Mutation
chr3	51458008	51458008	-	GAACTTGAC	ESCA15T_AF	DCAF1	Nonsense_Mutation
chr3	51743392	51743392	-	GGCAGTGG(	ESCA16T_AF	GRM2	Frame_Shift_Ins
chr3	52004199	52004199	-	CCTGAATC	ESCA07T_AF	ABHD14B	Frame_Shift_Ins
chr3	52266136	52266136	-	CGCTGGGA(	ESCA10T_AF	TWF2	Nonsense_Mutation
chr3	52407014	52407014	C	T	ESCA20T_AF	DNAH1	Silent
chr3	52474787	52474787	-	A	ESCA16T_AF	SEMA3G	Frame_Shift_Ins
chr3	52474788	52474788	G	C	ESCA16T_AF	SEMA3G	Missense_Mutation
chr3	5249908	5249908	-	AACTCTGGT	ESCA07T_AF	EDEM1	Frame_Shift_Ins
chr3	57882309	57882309	G	A	ESCA19T_AF	SLMAP	Missense_Mutation
chr3	58302295	58302295	-	TACCT	ESCA16T_AF	RPP14	Frame_Shift_Ins
chr3	58302297	58302297	-	TACATATTC	ESCA16T_AF	RPP14	Nonsense_Mutation
chr3	62204525	62204525	C	T	ESCA16T_AF	PTPRG	Missense_Mutation
chr3	62739250	62739250	G	A	ESCA07T_AF	CADPS	Missense_Mutation
chr3	63813381	63813381	C	T	ESCA15T_AF	C3orf49	Missense_Mutation
chr3	64601746	64601746	T	C	ESCA16T_AF	ADAMTS9	Missense_Mutation
chr3	64672435	64672435	A	G	ESCA17T_AF	ADAMTS9	Missense_Mutation
chr3	93615487	93615487	G	T	ESCA19T_AF	PROS1	Missense_Mutation
chr3	9419531	9419531	A	G	ESCA07T_AF	THUMPD3	Missense_Mutation
chr3	97806890	97806890	-	CTC	ESCA15T_AF	OR5AC2	In_Frame_Ins
chr3	97806891	97806891	-	AGCTGTAAA	ESCA15T_AF	OR5AC2	Nonsense_Mutation
chr3	9988377	9988377	C	G	ESCA17T_AF	PRRT3	Missense_Mutation
chr4	103557077	103557077	G	A	ESCA16T_AF	MANBA	Missense_Mutation
chr4	103645028	103645028	G	T	ESCA15T_AF	MANBA	Missense_Mutation
chr4	106763234	106763234	C	T	ESCA15T_AF	GSTCD	Silent
chr4	111539826	111539826	G	A	ESCA16T_AF	PITX2	Missense_Mutation
chr4	113359688	113359688	C	T	ESCA20T_AF	ALPK1	Silent
chr4	114277976	114277976	AGAAGAAA	-	ESCA07T_AF	ANK2	Frame_Shift_Del
chr4	114277987	114277987	-	TCTTTTTCTT	ESCA07T_AF	ANK2	Frame_Shift_Ins
chr4	119952989	119952989	A	G	ESCA15T_AF	SYNPO2	Missense_Mutation
chr4	121957452	121957452	C	G	ESCA18T_AF	NDNF	Missense_Mutation
chr4	123377579	123377579	-	T	ESCA14T_AF	IL2	Frame_Shift_Ins
chr4	123377580	123377580	-	CAACTCCTG	ESCA14T_AF	IL2	Frame_Shift_Ins
chr4	128807219	128807219	T	A	ESCA16T_AF	PLK4	Missense_Mutation
chr4	128949765	128949765	G	A	ESCA19T_AF	ABHD18	Missense_Mutation
chr4	129767674	129767674	C	T	ESCA15T_AF	JADE1	Nonsense_Mutation
chr4	134072199	134072199	G	A	ESCA16T_AF	PCDH10	Missense_Mutation
chr4	141600119	141600119	AGG	-	ESCA16T_AF	TBC1D9	In_Frame_Del
chr4	144619220	144619220	C	A	ESCA16T_AF	FREM3	Missense_Mutation
chr4	148545061	148545061	T	C	ESCA14T_AF	TMEM184C	Missense_Mutation
chr4	152640628	152640628	T	G	ESCA07T_AF	GATB	Silent
chr4	154644537	154644537	T	C	ESCA16T_AF	RNF175	Missense_Mutation
chr4	155487145	155487145	-	CCAGGTCTG	ESCA15T_AF	FGB	In_Frame_Ins
chr4	155665928	155665928	C	A	ESCA16T_AF	LRAT	Silent
chr4	156831228	156831228	A	G	ESCA15T_AF	TDO2	Silent
chr4	159092355	159092355	G	A	ESCA16T_AF	GASK1B	Missense_Mutation
chr4	164246779	164246779	G	C	ESCA17T_AF	NPY1R	Silent
chr4	16504480	16504480	C	G	ESCA16T_AF	LDB2	Missense_Mutation
chr4	166141094	166141094	A	C	ESCA01T_AF	KLHL2	Missense_Mutation
chr4	170077789	170077789	A	G	ESCA16T_AF	SH3RF1	Silent

chr4	173873242	173873242	C	A	ESCA18T_AF	GALNTL6	Missense_Mutation
chr4	175749940	175749940	C	T	ESCA16T_AF	GLRA3	Missense_Mutation
chr4	177113836	177113836	C	T	ESCA16T_AF	SPATA4	Silent
chr4	183268057	183268057	C	T	ESCA07T_AF	TENM3	Silent
chr4	183601807	183601807	-	CC	ESCA07T_AF	TENM3	Frame_Shift_Ins
chr4	183601814	183601814	G	C	ESCA07T_AF	TENM3	Missense_Mutation
chr4	183676180	183676180	-	TTG	ESCA15T_AF	TENM3	In_Frame_Ins
chr4	183676181	183676181	-	TGTCTGTCA(	ESCA15T_AF	TENM3	In_Frame_Ins
chr4	185687857	185687857	-	GACTTCGCC	ESCA14T_AF	ACSL1	Frame_Shift_Ins
chr4	185687862	185687862	C	A	ESCA14T_AF	ACSL1	Missense_Mutation
chr4	187122493	187122493	-	AATGATATC	ESCA15T_AF	CYP4V2	Nonsense_Mutation
chr4	187455446	187455446	G	A	ESCA15T_AF	MTNR1A	Silent
chr4	187518306	187518306	C	T	ESCA07T_AF	FAT1	Missense_Mutation
chr4	187630981	187630981	T	C	ESCA07T_AF	FAT1	Translation_Start_Site
chr4	188924024	188924024	C	T	ESCA16T_AF	ZFP42	Silent
chr4	190876263	190876263	A	T	ESCA17T_AF	FRG1	Missense_Mutation
chr4	24531298	24531298	T	A	ESCA07T_AF	DHX15	Missense_Mutation
chr4	24801683	24801683	C	A	ESCA14T_AF	SOD3	Silent
chr4	30724975	30724975	-	TCACATAAA	ESCA10T_AF	PCDH7	In_Frame_Ins
chr4	38016395	38016395	T	G	ESCA16T_AF	TBC1D1	Missense_Mutation
chr4	38798515	38798515	G	A	ESCA16T_AF	TLR1	Silent
chr4	39474796	39474796	C	T	ESCA01T_AF	LIAS	Missense_Mutation
chr4	40103929	40103929	C	G	ESCA15T_AF	N4BP2	Missense_Mutation
chr4	41016038	41016038	C	G	ESCA14T_AF	APBB2	Missense_Mutation
chr4	4190683	4190683	G	A	ESCA17T_AF	OTOP1	Silent
chr4	4198905	4198905	GAGGAACA	-	ESCA10T_AF	OTOP1	Frame_Shift_Del
chr4	4198918	4198918	-	CCTTCTTGT	ESCA10T_AF	OTOP1	Frame_Shift_Ins
chr4	4249855	4249855	G	A	ESCA16T_AF	TMEM128	Silent
chr4	4249895	4249895	-	GCCCGGCA(	ESCA15T_AF	TMEM128	Frame_Shift_Ins
chr4	42583747	42583747	G	A	ESCA20T_AF	ATP8A1	Missense_Mutation
chr4	42627695	42627695	C	T	ESCA15T_AF	ATP8A1	Missense_Mutation
chr4	42658874	42658874	C	T	ESCA18T_AF	ATP8A1	Missense_Mutation
chr4	4322570	4322570	G	A	ESCA16T_AF	ZBTB49	Missense_Mutation
chr4	44450366	44450366	G	C	ESCA19T_AF	KCTD8	Missense_Mutation
chr4	47643972	47643972	T	C	ESCA14T_AF	CORIN	Missense_Mutation
chr4	52928443	52928443	C	A	ESCA01T_AF	SPATA18	Missense_Mutation
chr4	5812738	5812738	A	T	ESCA16T_AF	EVC	Missense_Mutation
chr4	5812741	5812741	-	TTCTTCTCTT	ESCA16T_AF	EVC	In_Frame_Ins
chr4	6082016	6082016	C	T	ESCA18T_AF	JAKMIP1	Silent
chr4	6107406	6107406	-	GAGGAGGC(	ESCA07T_AF	JAKMIP1	Frame_Shift_Ins
chr4	62383064	62383064	T	C	ESCA18T_AF	ADGRL3	Silent
chr4	65180456	65180456	G	A	ESCA15T_AF	TECRL	Missense_Mutation
chr4	677103	677103	G	A	ESCA16T_AF	SLC49A3	Silent
chr4	68380091	68380091	T	C	ESCA18T_AF	CENPC	Missense_Mutation
chr4	6925449	6925449	G	A	ESCA16T_AF	TBC1D14	Silent
chr4	72897839	72897839	G	A	ESCA15T_AF	NPFFR2	Missense_Mutation
chr4	73931016	73931016	G	A	ESCA10T_AF	COX18	Silent
chr4	755186	755186	-	TGTTA	ESCA16T_AF	PCGF3	Frame_Shift_Ins
chr4	755187	755187	-	CTCGTTAAA	ESCA16T_AF	PCGF3	Nonsense_Mutation
chr4	75937971	75937971	C	T	ESCA16T_AF	PARM1	Missense_Mutation
chr4	75938179	75938179	G	A	ESCA16T_AF	PARM1	Silent
chr4	75938236	75938236	A	G	ESCA16T_AF	PARM1	Silent
chr4	77230885	77230885	T	C	ESCA17T_AF	STBD1	Missense_Mutation
chr4	77317509	77317509	G	A	ESCA15T_AF	CCDC158	Silent
chr4	77631425	77631425	T	A	ESCA16T_AF	SHROOM3	Missense_Mutation
chr4	78527039	78527039	C	A	ESCA19T_AF	CXCL13	Missense_Mutation
chr4	81865980	81865980	G	A	ESCA07T_AF	CFAP299	Missense_Mutation
chr4	83740264	83740264	-	CAG	ESCA15T_AF	SEC31A	In_Frame_Ins

chr4	83840042	83840042	-	A	ESCA20T_AF	THAP9	Frame_Shift_Ins
chr4	83860848	83860848	A	G	ESCA20T_AF	LIN54	Silent
chr4	84015937	84015937	-	CAGGGATCT	ESCA16T_AF	PLAC8	Nonsense_Mutation
chr4	84230072	84230072	-	GCCTGGCAA	ESCA14T_AF	HPSE	Frame_Shift_Ins
chr4	85658345	85658345	T	C	ESCA07T_AF	WDFY3	Missense_Mutation
chr4	85661486	85661486	-	ACCAGTTCT	ESCA15T_AF	WDFY3	Frame_Shift_Ins
chr4	85661488	85661488	-	ACACAAAA	ESCA15T_AF	WDFY3	Frame_Shift_Ins
chr4	8621111	8621111	C	A	ESCA10T_AF	CPZ	Missense_Mutation
chr4	87671683	87671683	G	T	ESCA17T_AF	PTPN13	Missense_Mutation
chr4	88035720	88035720	C	T	ESCA17T_AF	AFF1	Missense_Mutation
chr4	94693362	94693362	C	T	ESCA16T_AF	GRID2	Nonsense_Mutation
chr4	94750410	94750410	C	T	ESCA01T_AF	ATOH1	Silent
chr5	102360961	102360961	C	A	ESCA16T_AF	PAM	Missense_Mutation
chr5	107716432	107716432	TG	-	ESCA14T_AF	FBXL17	Frame_Shift_Del
chr5	1081702	1081702	A	G	ESCA16T_AF	SLC12A7	Silent
chr5	108680407	108680407	A	G	ESCA17T_AF	PJA2	Silent
chr5	110409316	110409316	-	TGG	ESCA10T_AF	TSLP	In_Frame_Ins
chr5	11732286	11732286	C	A	ESCA01T_AF	CTNND2	Nonsense_Mutation
chr5	118969698	118969698	C	T	ESCA07T_AF	FAM170A	Silent
chr5	121187726	121187726	G	A	ESCA07T_AF	FTMT	Missense_Mutation
chr5	122930735	122930735	G	A	ESCA18T_AF	CSNK1G3	Silent
chr5	127520183	127520183	A	G	ESCA07T_AF	SLC12A2	Missense_Mutation
chr5	128302311	128302311	-	GTAGAGTAT	ESCA10T_AF	SLC27A6	Nonsense_Mutation
chr5	128321013	128321013	T	C	ESCA19T_AF	SLC27A6	Silent
chr5	134724718	134724718	GAT	-	ESCA14T_AF	H2AFY	In_Frame_Del
chr5	137290040	137290040	-	TTTTAAGTCT	ESCA15T_AF	FAM13B	Nonsense_Mutation
chr5	137495276	137495276	T	C	ESCA01T_AF	BRD8	Missense_Mutation
chr5	13900500	13900500	G	A	ESCA16T_AF	DNAH5	Missense_Mutation
chr5	139862184	139862184	G	T	ESCA19T_AF	ANKHD1;ANKHI	Missense_Mutation
chr5	139938281	139938281	G	A	ESCA15T_AF	APBB3	Silent
chr5	139940997	139940997	G	C	ESCA07T_AF	APBB3	Missense_Mutation
chr5	140250027	140250027	G	A	ESCA19T_AF	PCDHA11	Missense_Mutation
chr5	140261956	140261956	G	A	ESCA16T_AF	PCDHA13	Missense_Mutation
chr5	140308093	140308093	T	A	ESCA20T_AF	PCDHAC1	Missense_Mutation
chr5	140557909	140557909	T	G	ESCA15T_AF	PCDHB8	Silent
chr5	140563579	140563579	C	T	ESCA16T_AF	PCDHB16	Missense_Mutation
chr5	140574309	140574309	G	A	ESCA10T_AF	PCDHB10	Silent
chr5	140580569	140580569	C	A	ESCA18T_AF	PCDHB11	Missense_Mutation
chr5	140588603	140588603	G	A	ESCA16T_AF	PCDHB12	Missense_Mutation
chr5	140595495	140595495	C	T	ESCA20T_AF	PCDHB13	Silent
chr5	140730536	140730536	-	CCGTGTTTA	ESCA16T_AF	PCDHGB1	Frame_Shift_Ins
chr5	140743962	140743962	C	T	ESCA17T_AF	PCDHGA5	Missense_Mutation
chr5	141694435	141694435	C	T	ESCA15T_AF	SPRY4	Missense_Mutation
chr5	14387956	14387956	-	CTGGCTTAC	ESCA10T_AF	TRIO	Frame_Shift_Ins
chr5	146798035	146798035	G	A	ESCA16T_AF	DPYSL3	Silent
chr5	148406803	148406803	CT	-	ESCA17T_AF	SH3TC2	Frame_Shift_Del
chr5	148411213	148411213	A	G	ESCA19T_AF	SH3TC2	Missense_Mutation
chr5	149216357	149216357	T	A	ESCA15T_AF	PPARGC1B	Missense_Mutation
chr5	149374932	149374932	T	C	ESCA16T_AF	TIGD6	Missense_Mutation
chr5	150892125	150892125	-	TT	ESCA07T_AF	FAT2	Frame_Shift_Ins
chr5	150892126	150892126	A	T	ESCA07T_AF	FAT2	Silent
chr5	150908876	150908876	-	CTTCCGGCT	ESCA07T_AF	FAT2	In_Frame_Ins
chr5	1523933	1523933	C	T	ESCA01T_AF	LPCAT1	Silent
chr5	156479619	156479619	C	A	ESCA17T_AF	HAVCR1	Silent
chr5	156908913	156908913	C	T	ESCA07T_AF	ADAM19	Silent
chr5	157078786	157078786	ACAGCCGC	-	ESCA20T_AF	SOX30	In_Frame_Del
chr5	158204507	158204507	C	A	ESCA15T_AF	EBF1	Missense_Mutation
chr5	158204509	158204509	-	CCCCTCCTC	ESCA15T_AF	EBF1	Frame_Shift_Ins

chr5	160721398	160721398	T	C	ESCA18T_AF	GABRB2	Missense_Mutation
chr5	16711327	16711327	C	T	ESCA18T_AF	MYO10	Missense_Mutation
chr5	169122890	169122890	T	G	ESCA14T_AF	DOCK2	Missense_Mutation
chr5	169435571	169435571	C	G	ESCA17T_AF	DOCK2	Nonsense_Mutation
chr5	172517808	172517808	C	G	ESCA15T_AF	CREBRF	Nonsense_Mutation
chr5	176022542	176022542	C	T	ESCA19T_AF	CDHR2	Missense_Mutation
chr5	176522434	176522434	C	T	ESCA19T_AF	FGFR4	Silent
chr5	176815124	176815124	T	C	ESCA16T_AF	SLC34A1	Silent
chr5	177612739	177612739	G	T	ESCA16T_AF	GMCL2	Missense_Mutation
chr5	177652419	177652419	-	AGGTCAGAA	ESCA07T_AF	PHYKPL	Frame_Shift_Ins
chr5	178030883	178030883	-	TAGATTGGG	ESCA10T_AF	CLK4	In_Frame_Ins
chr5	178608108	178608108	C	T	ESCA01T_AF	ADAMTS2	Missense_Mutation
chr5	178699939	178699939	G	C	ESCA14T_AF	ADAMTS2	Missense_Mutation
chr5	179023584	179023584	G	A	ESCA07T_AF	RUFY1	Silent
chr5	179106057	179106057	-	ATCACATCT	ESCA16T_AF	CBY3	In_Frame_Ins
chr5	26881314	26881314	G	A	ESCA15T_AF	CDH9	Silent
chr5	32400256	32400256	G	A	ESCA17T_AF	ZFR	Silent
chr5	32774858	32774858	C	A	ESCA19T_AF	NPR3	Nonsense_Mutation
chr5	37036499	37036499	G	T	ESCA15T_AF	NIPBL	Missense_Mutation
chr5	37330229	37330229	-	TATTCC	ESCA07T_AF	NUP155	In_Frame_Ins
chr5	37330233	37330233	-	TTTACACAG	ESCA07T_AF	NUP155	Frame_Shift_Ins
chr5	37443448	37443448	A	G	ESCA14T_AF	WDR70	Silent
chr5	40681118	40681118	C	T	ESCA16T_AF	PTGER4	Missense_Mutation
chr5	41049412	41049412	A	G	ESCA07T_AF	MROH2B	Missense_Mutation
chr5	41057272	41057272	TCTGCAGA	-	ESCA14T_AF	MROH2B	Frame_Shift_Del
chr5	41065481	41065481	G	A	ESCA15T_AF	MROH2B	Missense_Mutation
chr5	52856504	52856504	G	C	ESCA16T_AF	NDUFS4	Silent
chr5	5464093	5464093	C	G	ESCA18T_AF	ICE1	Missense_Mutation
chr5	5466544	5466544	A	T	ESCA17T_AF	ICE1	Missense_Mutation
chr5	56155654	56155654	G	A	ESCA19T_AF	MAP3K1	Missense_Mutation
chr5	65084142	65084142	-	TTGAGGTGT	ESCA07T_AF	NLN	Frame_Shift_Ins
chr5	66459776	66459776	A	G	ESCA16T_AF	MAST4	Missense_Mutation
chr5	71490022	71490022	C	A	ESCA15T_AF	MAP1B	Missense_Mutation
chr5	72378647	72378647	-	AGGACACAC	ESCA10T_AF	FCHO2	In_Frame_Ins
chr5	72378649	72378649	-	ATTTTTCT	ESCA10T_AF	FCHO2	Frame_Shift_Ins
chr5	73190223	73190223	-	CATAGAAGA	ESCA07T_AF	ARHGEF28	Nonsense_Mutation
chr5	74400427	74400427	C	T	ESCA01T_AF	ANKRD31	Missense_Mutation
chr5	74892955	74892955	G	C	ESCA15T_AF	POLK	Missense_Mutation
chr5	78746939	78746939	-	TTTTAGGCA	ESCA10T_AF	HOMER1	Nonsense_Mutation
chr5	80508251	80508251	G	A	ESCA15T_AF	RASGRF2	Missense_Mutation
chr5	80762848	80762848	C	T	ESCA17T_AF	SSBP2	Missense_Mutation
chr5	89757037	89757037	G	A	ESCA01T_AF	MBLAC2	Nonsense_Mutation
chr5	89913628	89913628	G	C	ESCA15T_AF	ADGRV1	Missense_Mutation
chr5	89985853	89985853	C	A	ESCA19T_AF	ADGRV1	Silent
chr5	93077199	93077199	G	A	ESCA19T_AF	POU5F2	Missense_Mutation
chr5	94230358	94230358	C	T	ESCA16T_AF	MCTP1	Missense_Mutation
chr5	96121524	96121524	C	T	ESCA16T_AF	ERAP1	Silent
chr5	96126308	96126308	A	G	ESCA16T_AF	ERAP1	Silent
chr5	96130836	96130836	T	C	ESCA16T_AF	ERAP1	Missense_Mutation
chr5	98224831	98224831	G	A	ESCA15T_AF	CHD1	Silent
chr6	10529942	10529942	C	T	ESCA19T_AF	GCNT2	Silent
chr6	106764003	106764003	T	C	ESCA17T_AF	ATG5	Missense_Mutation
chr6	107390204	107390204	G	T	ESCA19T_AF	BEND3	Missense_Mutation
chr6	10756712	10756712	C	T	ESCA15T_AF	TMEM14B	Silent
chr6	10756712	10756712	C	T	ESCA16T_AF	TMEM14B	Silent
chr6	10756728	10756728	C	T	ESCA15T_AF	TMEM14B	Missense_Mutation
chr6	10912922	10912922	A	G	ESCA18T_AF	SYCP2L	Missense_Mutation
chr6	109816599	109816599	G	A	ESCA16T_AF	AK9	Missense_Mutation

chr6	109816606	109816606	-	CCACACAAC	ESCA16T_AF	AK9	Frame_Shift_Ins
chr6	111901517	111901517	G	C	ESCA18T_AF	TRAF3IP2	Missense_Mutation
chr6	117589622	117589622	-	CAAAGCACC	ESCA15T_AF	VGLL2	Frame_Shift_Ins
chr6	117677792	117677792	C	T	ESCA17T_AF	ROS1	Missense_Mutation
chr6	12123699	12123699	C	G	ESCA15T_AF	HIVEP1	Nonsense_Mutation
chr6	121605051	121605051	C	T	ESCA16T_AF	TBC1D32	Missense_Mutation
chr6	12292620	12292620	G	A	ESCA15T_AF	EDN1	Silent
chr6	123101437	123101437	C	T	ESCA16T_AF	FABP7	Silent
chr6	125284090	125284090	G	A	ESCA17T_AF	RNF217	Missense_Mutation
chr6	126210123	126210123	G	C	ESCA18T_AF	NCOA7	Missense_Mutation
chr6	128316664	128316664	-	ATCACTCTG	ESCA16T_AF	PTPRK	Nonsense_Mutation
chr6	129824416	129824416	-	GGGACTATT	ESCA14T_AF	LAMA2	Frame_Shift_Ins
chr6	130761798	130761798	T	C	ESCA16T_AF	TMEM200A	Silent
chr6	131148649	131148649	G	A	ESCA18T_AF	SMLR1	Silent
chr6	131540921	131540921	-	ATCACTGTG	ESCA16T_AF	AKAP7	Nonsense_Mutation
chr6	132938701	132938701	A	G	ESCA19T_AF	TAAR2	Missense_Mutation
chr6	135511466	135511466	G	A	ESCA20T_AF	MYB	Missense_Mutation
chr6	136599668	136599668	T	C	ESCA17T_AF	BCLAF1	Silent
chr6	137187866	137187866	-	CTATACCTC	ESCA07T_AF	PEX7	In_Frame_Ins
chr6	138531075	138531075	C	T	ESCA15T_AF	ARFGEF3	Missense_Mutation
chr6	13977897	13977897	C	T	ESCA16T_AF	RNF182	Missense_Mutation
chr6	142397175	142397175	C	T	ESCA15T_AF	NMBR	Silent
chr6	144858847	144858847	-	ATAAACAAC	ESCA07T_AF	UTRN	In_Frame_Ins
chr6	144858851	144858851	-	TTCTT	ESCA07T_AF	UTRN	Frame_Shift_Ins
chr6	144878376	144878376	G	A	ESCA18T_AF	UTRN	Silent
chr6	146266706	146266706	G	A	ESCA15T_AF	SHPRH	Missense_Mutation
chr6	146678819	146678819	G	A	ESCA17T_AF	GRM1	Missense_Mutation
chr6	147684539	147684539	C	T	ESCA15T_AF	STXBP5	Missense_Mutation
chr6	150240915	150240915	A	G	ESCA01T_AF	RAET1G	Silent
chr6	151766703	151766703	G	A	ESCA20T_AF	RMND1	Missense_Mutation
chr6	151859434	151859434	A	G	ESCA07T_AF	CCDC170	Silent
chr6	151859434	151859434	A	G	ESCA16T_AF	CCDC170	Silent
chr6	152542613	152542613	G	T	ESCA17T_AF	SYNE1	Missense_Mutation
chr6	152746626	152746626	C	A	ESCA16T_AF	SYNE1	Missense_Mutation
chr6	152763311	152763311	C	A	ESCA17T_AF	SYNE1	Nonsense_Mutation
chr6	154360621	154360621	G	A	ESCA15T_AF	OPRM1	Missense_Mutation
chr6	15496886	15496886	-	TTCCTCTCC	ESCA16T_AF	JARID2	In_Frame_Ins
chr6	158922754	158922754	G	A	ESCA18T_AF	TULP4	Missense_Mutation
chr6	160858230	160858230	G	A	ESCA16T_AF	SLC22A3	Silent
chr6	161139389	161139389	G	A	ESCA17T_AF	PLG	Missense_Mutation
chr6	166914396	166914396	C	T	ESCA17T_AF	RPS6KA2	Missense_Mutation
chr6	167343202	167343202	C	G	ESCA18T_AF	RNASET2	Missense_Mutation
chr6	168314955	168314955	-	AAAACATCT	ESCA15T_AF	AFDN	Nonsense_Mutation
chr6	168711960	168711960	C	T	ESCA20T_AF	DACT2	Missense_Mutation
chr6	169633053	169633053	A	G	ESCA15T_AF	THBS2	Missense_Mutation
chr6	17637538	17637538	C	G	ESCA17T_AF	NUP153	Missense_Mutation
chr6	20212391	20212391	C	T	ESCA16T_AF	MBOAT1	Silent
chr6	24145875	24145875	G	A	ESCA10T_AF	NRSN1	Missense_Mutation
chr6	24437435	24437435	C	T	ESCA01T_AF	GPLD1	Silent
chr6	25773861	25773861	C	G	ESCA16T_AF	SLC17A4	Missense_Mutation
chr6	25819966	25819966	C	T	ESCA07T_AF	SLC17A1	Missense_Mutation
chr6	25916979	25916979	C	T	ESCA16T_AF	SLC17A2	Silent
chr6	26205083	26205083	GTGACTTAC	-	ESCA01T_AF	HIST1H4E	In_Frame_Del
chr6	26373156	26373156	C	T	ESCA19T_AF	BTN3A2	Silent
chr6	26468478	26468478	G	C	ESCA10T_AF	BTN2A1	Missense_Mutation
chr6	26501858	26501858	C	T	ESCA19T_AF	BTN1A1	Silent
chr6	26505428	26505428	-	GCAGCATTG	ESCA07T_AF	BTN1A1	In_Frame_Ins
chr6	27858350	27858350	T	C	ESCA07T_AF	HIST1H3J	Missense_Mutation

chr6	28402838	28402838	A	C	ESCA01T_AF	ZSCAN23	Missense_Mutation
chr6	2890547	2890547	G	T	ESCA19T_AF	SERPINB9	Silent
chr6	32605271	32605271	C	T	ESCA17T_AF	HLA-DQA1	Silent
chr6	32605274	32605274	T	C	ESCA17T_AF	HLA-DQA1	Silent
chr6	34004249	34004249	-	GTGCTGGCA	ESCA16T_AF	GRM4	Frame_Shift_Ins
chr6	34029800	34029800	C	T	ESCA18T_AF	GRM4	Missense_Mutation
chr6	35086282	35086282	T	C	ESCA15T_AF	TCP11	Missense_Mutation
chr6	35287311	35287311	C	G	ESCA15T_AF	DEF6	Missense_Mutation
chr6	35287315	35287315	GCAGGCTG/	-	ESCA15T_AF	DEF6	In_Frame_Del
chr6	35427213	35427213	C	T	ESCA17T_AF	FANCE	Missense_Mutation
chr6	36177686	36177686	-	CCTCACTCA	ESCA15T_AF	BRPF3	In_Frame_Ins
chr6	36177688	36177688	-	TTGAC	ESCA15T_AF	BRPF3	Nonsense_Mutation
chr6	36672219	36672219	G	A	ESCA15T_AF	RAB44	Silent
chr6	36922608	36922608	C	T	ESCA19T_AF	PI16	Silent
chr6	38029453	38029453	G	T	ESCA16T_AF	ZFAND3	Missense_Mutation
chr6	38854727	38854727	-	AAACACAGT	ESCA16T_AF	DNAH8	Frame_Shift_Ins
chr6	39284053	39284053	C	A	ESCA16T_AF	KCNK16	Missense_Mutation
chr6	39513390	39513390	C	T	ESCA17T_AF	KIF6	Missense_Mutation
chr6	4069955	4069955	T	A	ESCA07T_AF	FAM217A	Missense_Mutation
chr6	41000822	41000822	G	A	ESCA18T_AF	UNC5CL	Silent
chr6	42689755	42689755	A	G	ESCA16T_AF	PRPH2	Silent
chr6	43011274	43011274	G	A	ESCA15T_AF	CUL7	Silent
chr6	43492578	43492578	G	A	ESCA16T_AF	XPO5	Silent
chr6	44102798	44102798	C	A	ESCA16T_AF	TMEM63B	Missense_Mutation
chr6	44137083	44137083	-	TTGTCGTGC	ESCA16T_AF	CAPN11	In_Frame_Ins
chr6	46107942	46107942	A	-	ESCA18T_AF	ENPP4	Nonsense_Mutation
chr6	46107944	46107944	A	C	ESCA18T_AF	ENPP4	Missense_Mutation
chr6	46620315	46620315	T	C	ESCA16T_AF	CYP39A1	Missense_Mutation
chr6	47254056	47254056	-	ATGATTGAG	ESCA16T_AF	TNFRSF21	Nonsense_Mutation
chr6	4735035	4735035	T	C	ESCA16T_AF	CDYL	Missense_Mutation
chr6	4735071	4735071	C	G	ESCA16T_AF	CDYL	Missense_Mutation
chr6	52380805	52380805	G	A	ESCA07T_AF	TRAM2	Missense_Mutation
chr6	55304301	55304301	T	C	ESCA16T_AF	HMGCLL1	Silent
chr6	56366026	56366026	-	CTTTTTCT	ESCA16T_AF	DST	Frame_Shift_Ins
chr6	56366028	56366028	-	AGCATAAAC	ESCA16T_AF	DST	Frame_Shift_Ins
chr6	56376192	56376192	G	A	ESCA18T_AF	DST	Missense_Mutation
chr6	56420516	56420516	C	T	ESCA17T_AF	DST	Silent
chr6	56882197	56882197	GG	-	ESCA10T_AF	BEND6;BEND6	Frame_Shift_Del
chr6	56966553	56966553	AGAGT	-	ESCA07T_AF	ZNF451	Frame_Shift_Del
chr6	64395457	64395457	C	T	ESCA20T_AF	PHF3	Missense_Mutation
chr6	64431217	64431217	C	T	ESCA18T_AF	EYS	Missense_Mutation
chr6	65149236	65149236	C	G	ESCA19T_AF	EYS	Missense_Mutation
chr6	70071070	70071070	G	A	ESCA15T_AF	ADGRB3	Missense_Mutation
chr6	70610154	70610154	T	C	ESCA19T_AF	COL19A1	Missense_Mutation
chr6	70639378	70639378	G	A	ESCA19T_AF	COL19A1	Missense_Mutation
chr6	7572252	7572252	C	A	ESCA15T_AF	DSP	Missense_Mutation
chr6	7572257	7572257	-	CCTGGTCTG	ESCA15T_AF	DSP	Frame_Shift_Ins
chr6	7580958	7580958	A	G	ESCA16T_AF	DSP	Missense_Mutation
chr6	78172260	78172260	C	G	ESCA16T_AF	HTR1B	Silent
chr6	79679854	79679854	C	A	ESCA07T_AF	PHIP	Nonsense_Mutation
chr6	83845523	83845523	A	G	ESCA16T_AF	DOP1A	Missense_Mutation
chr6	84563460	84563460	G	A	ESCA15T_AF	RIPPLY2	Missense_Mutation
chr6	87966173	87966173	T	C	ESCA16T_AF	ZNF292	Silent
chr6	89888744	89888744	C	T	ESCA16T_AF	GABRR1	Silent
chr6	90499954	90499954	T	C	ESCA18T_AF	MDN1	Missense_Mutation
chr6	93967986	93967986	G	C	ESCA14T_AF	EPHA7	Silent
chr6	96974211	96974211	G	C	ESCA14T_AF	UFL1	Missense_Mutation
chr6	97246843	97246843	-	CTTATGTGA	ESCA10T_AF	GPR63	Nonsense_Mutation

chr7	100014027	100014027	C	T	ESCA16T_AF	ZCWPW1	Missense_Mutation
chr7	100609556	100609556	C	A	ESCA16T_AF	MUC3A	Missense_Mutation
chr7	100731847	100731847	C	A	ESCA19T_AF	TRIM56	Silent
chr7	102212929	102212929	C	T	ESCA16T_AF	POLR2J3	Missense_Mutation
chr7	103126769	103126769	A	G	ESCA17T_AF	RELN	Silent
chr7	104752572	104752572	C	T	ESCA15T_AF	KMT2E	Missense_Mutation
chr7	105183093	105183093	T	A	ESCA15T_AF	RINT1	Missense_Mutation
chr7	105183096	105183096	-	AGGCCCATT	ESCA15T_AF	RINT1	Frame_Shift_Ins
chr7	106545750	106545750	G	C	ESCA15T_AF	PIK3CG	Missense_Mutation
chr7	106545770	106545770	C	T	ESCA01T_AF	PIK3CG	Nonsense_Mutation
chr7	107704222	107704222	G	C	ESCA01T_AF	LAMB4	Silent
chr7	11446665	11446665	G	A	ESCA10T_AF	THSD7A	Nonsense_Mutation
chr7	11501690	11501690	G	A	ESCA19T_AF	THSD7A	Missense_Mutation
chr7	116528240	116528240	C	T	ESCA16T_AF	CAPZA2	Silent
chr7	11675855	11675855	C	T	ESCA16T_AF	THSD7A	Silent
chr7	121960329	121960329	C	T	ESCA19T_AF	CADPS2	Missense_Mutation
chr7	122130330	122130330	G	T	ESCA01T_AF	CADPS2	Missense_Mutation
chr7	123302007	123302007	G	T	ESCA15T_AF	LMOD2	Nonsense_Mutation
chr7	126544657	126544657	G	T	ESCA16T_AF	GRM8	Missense_Mutation
chr7	12666278	12666278	G	C	ESCA16T_AF	SCIN	Missense_Mutation
chr7	127013709	127013709	T	C	ESCA14T_AF	ZNF800	Missense_Mutation
chr7	127240449	127240449	T	G	ESCA10T_AF	FSCN3	Missense_Mutation
chr7	128587950	128587950	G	T	ESCA15T_AF	IRF5	Nonsense_Mutation
chr7	132659958	132659958	C	T	ESCA20T_AF	CHCHD3	Missense_Mutation
chr7	133160122	133160122	C	T	ESCA07T_AF	EXOC4	Missense_Mutation
chr7	134894374	134894374	T	A	ESCA14T_AF	WDR91	Missense_Mutation
chr7	134931364	134931364	C	A	ESCA10T_AF	STRA8	Nonsense_Mutation
chr7	137531456	137531456	G	C	ESCA17T_AF	DGKI	Silent
chr7	138313015	138313015	C	T	ESCA14T_AF	SVOPL	Silent
chr7	140107482	140107482	G	T	ESCA20T_AF	RAB19	Missense_Mutation
chr7	141362632	141362632	-	TTTCCTAGA/	ESCA14T_AF	DENND11	Nonsense_Mutation
chr7	141864673	141864673	G	T	ESCA15T_AF	MGAM2	Missense_Mutation
chr7	142566369	142566369	G	A	ESCA07T_AF	EPHB6	Missense_Mutation
chr7	143095951	143095951	-	CCCCCGTCC	ESCA10T_AF	EPHA1	Frame_Shift_Ins
chr7	148851261	148851261	G	A	ESCA15T_AF	ZNF398	Silent
chr7	149172218	149172218	C	T	ESCA19T_AF	ZNF746	Missense_Mutation
chr7	150325001	150325001	C	A	ESCA17T_AF	GIMAP6	Missense_Mutation
chr7	150811867	150811867	GGCTGGCC(	-	ESCA15T_AF	AGAP3	In_Frame_Del
chr7	150811912	150811912	TGCCGCCG(	-	ESCA15T_AF	AGAP3	Frame_Shift_Del
chr7	150878260	150878260	C	G	ESCA16T_AF	ASB10	Silent
chr7	150932286	150932286	G	T	ESCA15T_AF	CHPF2	Missense_Mutation
chr7	155299769	155299769	-	TGAGGATAT	ESCA14T_AF	CNPY1	In_Frame_Ins
chr7	155596205	155596205	G	A	ESCA20T_AF	SHH	Missense_Mutation
chr7	158672619	158672619	A	G	ESCA16T_AF	WDR60	Missense_Mutation
chr7	20824785	20824785	C	T	ESCA19T_AF	SP8	Silent
chr7	22985334	22985334	C	G	ESCA07T_AF	FAM126A	Missense_Mutation
chr7	27135314	27135314	CGA	-	ESCA07T_AF	HOXA1	In_Frame_Del
chr7	27135319	27135319	G	A	ESCA07T_AF	HOXA1	Silent
chr7	27196154	27196154	G	A	ESCA14T_AF	HOXA7	Missense_Mutation
chr7	27196156	27196156	-	TCACAGGTC	ESCA14T_AF	HOXA7	Nonsense_Mutation
chr7	27239545	27239545	GCAGCCGC(	-	ESCA17T_AF	HOXA13	In_Frame_Del
chr7	29923733	29923733	-	A	ESCA15T_AF	WIPF3	Frame_Shift_Ins
chr7	30490952	30490952	G	T	ESCA18T_AF	NOD1	Missense_Mutation
chr7	31594510	31594510	G	A	ESCA17T_AF	ITPRID1	Missense_Mutation
chr7	31912919	31912919	T	A	ESCA16T_AF	PDE1C	Missense_Mutation
chr7	32909182	32909182	G	A	ESCA16T_AF	KBTBD2	Silent
chr7	33427718	33427718	C	T	ESCA16T_AF	BBS9	Nonsense_Mutation
chr7	36917693	36917693	-	GTCGGCTTT(	ESCA10T_AF	ELMO1	In_Frame_Ins

chr7	36934491	36934491	C	T	ESCA17T_AF	ELMO1	Missense_Mutation
chr7	37947206	37947206	C	T	ESCA10T_AF	SFRP4	Missense_Mutation
chr7	44797091	44797091	C	T	ESCA16T_AF	ZMIZ2	Silent
chr7	45960625	45960625	G	T	ESCA18T_AF	IGFBP3	Missense_Mutation
chr7	47968902	47968902	A	T	ESCA19T_AF	PKD1L1	Missense_Mutation
chr7	48081112	48081112	-	AGGGGGCT(	ESCA07T_AF	C7orf57	Frame_Shift_Ins
chr7	4901292	4901292	G	A	ESCA15T_AF	PAPOLB	Silent
chr7	50070859	50070859	C	T	ESCA10T_AF	ZPBP	Missense_Mutation
chr7	50514455	50514455	C	A	ESCA14T_AF	FIGNL1	Silent
chr7	552046	552046	A	G	ESCA16T_AF	PDGFA	Silent
chr7	55902153	55902153	C	A	ESCA15T_AF	SEPTIN14	Nonsense_Mutation
chr7	55902231	55902231	C	G	ESCA15T_AF	SEPTIN14	Missense_Mutation
chr7	5984721	5984721	C	T	ESCA07T_AF	RSPH10B;RSPH1	Silent
chr7	6189323	6189323	C	T	ESCA16T_AF	USP42	Missense_Mutation
chr7	71252870	71252870	T	C	ESCA07T_AF	CALN1	Missense_Mutation
chr7	71252878	71252878	-	CCCACCACC	ESCA07T_AF	CALN1	Frame_Shift_Ins
chr7	71571175	71571175	C	T	ESCA17T_AF	CALN1	Missense_Mutation
chr7	75912144	75912144	-	TGGGGCTGT	ESCA15T_AF	SRRM3	In_Frame_Ins
chr7	77755056	77755056	C	T	ESCA17T_AF	MAGI2	Silent
chr7	794242	794242	G	A	ESCA07T_AF	DNAAF5	Silent
chr7	81637072	81637072	C	T	ESCA15T_AF	CACNA2D1	Missense_Mutation
chr7	82584865	82584865	T	A	ESCA18T_AF	PCLO	Missense_Mutation
chr7	83037760	83037760	-	CTGCTCCCG	ESCA16T_AF	SEMA3E	Frame_Shift_Ins
chr7	86469103	86469103	C	T	ESCA17T_AF	GRM3	Missense_Mutation
chr7	87323225	87323225	G	T	ESCA19T_AF	RUNDC3B	Missense_Mutation
chr7	91700320	91700320	-	TAAACTCCT	ESCA10T_AF	AKAP9	Nonsense_Mutation
chr7	94045779	94045779	A	G	ESCA10T_AF	COL1A2	Silent
chr7	94045783	94045783	-	CAGAAGGA(	ESCA10T_AF	COL1A2	Frame_Shift_Ins
chr7	94230079	94230079	C	T	ESCA19T_AF	SGCE	Missense_Mutation
chr7	94293611	94293611	G	A	ESCA17T_AF	PEG10	Missense_Mutation
chr7	98467406	98467406	C	T	ESCA18T_AF	TMEM130	Missense_Mutation
chr7	99247824	99247824	A	G	ESCA18T_AF	CYP3A5	Missense_Mutation
chr7	99774662	99774662	-	CCCGGCAG(	ESCA10T_AF	GPC2	Frame_Shift_Ins
chr8	101052308	101052308	G	A	ESCA07T_AF	RGS22	Missense_Mutation
chr8	101153725	101153725	-	G	ESCA07T_AF	FBXO43	Frame_Shift_Ins
chr8	101153726	101153726	-	ATATCTCCA	ESCA07T_AF	FBXO43	Nonsense_Mutation
chr8	103852046	103852046	CCC	-	ESCA10T_AF	AZIN1	In_Frame_Del
chr8	103852051	103852051	TCTATTA	-	ESCA10T_AF	AZIN1	Frame_Shift_Del
chr8	108509634	108509634	A	G	ESCA07T_AF	ANGPT1	Silent
chr8	109215235	109215235	G	C	ESCA14T_AF	EIF3E	Missense_Mutation
chr8	110441668	110441668	C	T	ESCA16T_AF	PKHD1L1	Missense_Mutation
chr8	110492351	110492351	-	CCTGCAGTG	ESCA10T_AF	PKHD1L1	Nonsense_Mutation
chr8	11163889	11163889	-	GACTTATGA	ESCA14T_AF	MTMR9	Frame_Shift_Ins
chr8	113267652	113267652	C	T	ESCA20T_AF	CSMD3	Silent
chr8	113363422	113363422	G	T	ESCA01T_AF	CSMD3	Missense_Mutation
chr8	113662505	113662505	G	A	ESCA17T_AF	CSMD3	Silent
chr8	11566350	11566350	G	C	ESCA15T_AF	GATA4	Missense_Mutation
chr8	120768351	120768351	-	TC	ESCA15T_AF	TAF2	Frame_Shift_Ins
chr8	120768352	120768352	-	GGCATAAGA	ESCA15T_AF	TAF2	In_Frame_Ins
chr8	120801879	120801879	A	C	ESCA19T_AF	TAF2	Missense_Mutation
chr8	125103704	125103704	G	A	ESCA19T_AF	FER1L6	Missense_Mutation
chr8	126448541	126448541	G	A	ESCA19T_AF	TRIB1	Missense_Mutation
chr8	12878820	12878820	G	A	ESCA19T_AF	TRMT9B	Missense_Mutation
chr8	12879198	12879198	A	T	ESCA16T_AF	TRMT9B	Missense_Mutation
chr8	131073206	131073206	T	A	ESCA17T_AF	ASAP1	Silent
chr8	131859727	131859727	C	T	ESCA15T_AF	ADCY8	Silent
chr8	133669080	133669080	C	T	ESCA19T_AF	LRRC6	Silent
chr8	142264273	142264273	G	A	ESCA19T_AF	SLC45A4	Missense_Mutation

chr8	144808475	144808475	C	T	ESCA15T_AF	FAM83H	Silent
chr8	144809492	144809492	C	G	ESCA15T_AF	FAM83H	Missense_Mutation
chr8	144887488	144887488	C	T	ESCA07T_AF	SCRIB	Missense_Mutation
chr8	144893084	144893084	G	A	ESCA16T_AF	SCRIB	Missense_Mutation
chr8	145006968	145006968	C	T	ESCA16T_AF	PLEC	Missense_Mutation
chr8	145007110	145007110	C	A	ESCA15T_AF	PLEC	Missense_Mutation
chr8	145150791	145150791	C	T	ESCA15T_AF	CYC1	Missense_Mutation
chr8	145154331	145154331	CACCTGTG(	-	ESCA19T_AF	SHARPIN	In_Frame_Del
chr8	145154521	145154521	CCTGTGCCA	-	ESCA19T_AF	SHARPIN	Frame_Shift_Del
chr8	145158650	145158650	GC	-	ESCA19T_AF	SHARPIN	Frame_Shift_Del
chr8	145158654	145158654	-	TCATTTTTTT	ESCA19T_AF	SHARPIN	Translation_Start_Site
chr8	145168560	145168560	CC	-	ESCA16T_AF	WDR97	Frame_Shift_Del
chr8	145620363	145620363	G	A	ESCA07T_AF	CPSF1	Silent
chr8	145675839	145675839	C	T	ESCA15T_AF	CYHR1	Missense_Mutation
chr8	145749658	145749658	A	T	ESCA16T_AF	LRRC24	Missense_Mutation
chr8	145749661	145749661	-	CTTACTTCA(	ESCA16T_AF	LRRC24	Nonsense_Mutation
chr8	1616718	1616718	A	G	ESCA16T_AF	DLGAP2	Silent
chr8	17417968	17417968	G	A	ESCA07T_AF	SLC7A2	Missense_Mutation
chr8	19251028	19251028	-	TGATATTCC/	ESCA07T_AF	SH2D4A	Nonsense_Mutation
chr8	19818457	19818457	A	G	ESCA17T_AF	LPL	Silent
chr8	21983002	21983002	C	T	ESCA16T_AF	HR	Silent
chr8	22052380	22052380	C	T	ESCA20T_AF	BMP1	Silent
chr8	22471824	22471824	G	A	ESCA16T_AF	CCAR2	Silent
chr8	23147774	23147774	C	T	ESCA16T_AF	R3HCC1	Missense_Mutation
chr8	27099963	27099963	G	A	ESCA07T_AF	STMN4	Silent
chr8	27737257	27737257	G	A	ESCA17T_AF	SCARA5	Missense_Mutation
chr8	29989708	29989708	-	CAGG	ESCA14T_AF	MBOAT4	Frame_Shift_Ins
chr8	29989710	29989710	-	AGGTGTTTG	ESCA14T_AF	MBOAT4	Frame_Shift_Ins
chr8	30700413	30700413	A	G	ESCA01T_AF	TEX15	Silent
chr8	3076805	3076805	G	T	ESCA19T_AF	CSMD1	Silent
chr8	3165342	3165342	C	T	ESCA17T_AF	CSMD1	Silent
chr8	37720608	37720608	-	ACAGCCCCT	ESCA14T_AF	RAB11FIP1	Frame_Shift_Ins
chr8	37720612	37720612	-	CT	ESCA14T_AF	RAB11FIP1	Frame_Shift_Ins
chr8	3855619	3855619	G	A	ESCA17T_AF	CSMD1	Silent
chr8	39606884	39606884	C	T	ESCA16T_AF	ADAM2	Missense_Mutation
chr8	42220211	42220211	-	ACTTACCAT(	ESCA07T_AF	POLB	Nonsense_Mutation
chr8	48650063	48650063	T	C	ESCA14T_AF	CEBPD	Missense_Mutation
chr8	55540097	55540097	A	T	ESCA18T_AF	RP1	Missense_Mutation
chr8	55541617	55541617	A	G	ESCA16T_AF	RP1	Silent
chr8	56015063	56015063	A	T	ESCA01T_AF	XKR4	Silent
chr8	59347062	59347062	G	T	ESCA19T_AF	UBXN2B	Nonsense_Mutation
chr8	62588824	62588824	T	A	ESCA19T_AF	ASPH	Missense_Mutation
chr8	67492598	67492598	G	A	ESCA19T_AF	MYBL1	Missense_Mutation
chr8	71069055	71069055	G	T	ESCA18T_AF	NCOA2	Silent
chr8	73849406	73849406	-	GGTGAAGCT	ESCA16T_AF	KCNB2	Nonsense_Mutation
chr8	73993342	73993342	G	A	ESCA19T_AF	SBSPON	Silent
chr8	8235174	8235174	C	T	ESCA17T_AF	PRAG1	Missense_Mutation
chr8	87679290	87679290	G	A	ESCA14T_AF	CNGB3	Missense_Mutation
chr8	88365988	88365988	C	A	ESCA17T_AF	CNBD1	Missense_Mutation
chr8	91072925	91072925	C	T	ESCA17T_AF	CALB1	Missense_Mutation
chr8	92378855	92378855	T	A	ESCA17T_AF	SLC26A7	Silent
chr8	95522081	95522081	A	G	ESCA16T_AF	VIRMA	Silent
chr8	95524271	95524271	-	AGTTGGCCT	ESCA07T_AF	VIRMA	Nonsense_Mutation
chr8	95863796	95863796	A	T	ESCA18T_AF	INTS8	Missense_Mutation
chr8	97256275	97256275	C	T	ESCA15T_AF	MTERF3	Missense_Mutation
chr8	97620644	97620644	G	T	ESCA20T_AF	SDC2	Missense_Mutation
chr8	98155399	98155399	G	T	ESCA16T_AF	CPQ	Silent
chr9	100076935	100076935	G	A	ESCA19T_AF	CCDC180	Silent

chr9	101558681	101558681	C	T	ESCA16T_AF	ANKS6	Silent
chr9	101767315	101767315	G	A	ESCA16T_AF	COL15A1	Missense_Mutation
chr9	102677586	102677586	A	C	ESCA20T_AF	STX17	Missense_Mutation
chr9	104314921	104314921	-	TCTCTCTCTT	ESCA15T_AF	RNF20	In_Frame_Ins
chr9	104314922	104314922	-	ACTCTTTTAC	ESCA15T_AF	RNF20	Frame_Shift_Ins
chr9	1053766	1053766	-	CTGGGGGCT	ESCA16T_AF	DMRT2	Frame_Shift_Ins
chr9	107266943	107266943	G	A	ESCA16T_AF	OR13F1	Missense_Mutation
chr9	107332386	107332386	G	A	ESCA15T_AF	OR13C8	Missense_Mutation
chr9	107457274	107457274	A	G	ESCA19T_AF	OR13D1	Missense_Mutation
chr9	112899720	112899720	G	A	ESCA16T_AF	PALM2-AKAP2	Silent
chr9	114411945	114411945	G	T	ESCA16T_AF	DNAJC25	Silent
chr9	116357924	116357924	A	C	ESCA07T_AF	RGS3	Missense_Mutation
chr9	117138846	117138846	G	T	ESCA16T_AF	AKNA	Missense_Mutation
chr9	117835955	117835955	-	TGTCCTGAG	ESCA10T_AF	TNC	In_Frame_Ins
chr9	119625888	119625888	G	A	ESCA10T_AF	ASTN2	Missense_Mutation
chr9	120475152	120475152	G	T	ESCA18T_AF	TLR4	Missense_Mutation
chr9	123171539	123171539	-	AAGACCGTC	ESCA16T_AF	CDK5RAP2	In_Frame_Ins
chr9	124074630	124074630	G	A	ESCA17T_AF	GSN	Nonsense_Mutation
chr9	124929055	124929055	G	A	ESCA16T_AF	MORN5	Missense_Mutation
chr9	125289230	125289230	C	A	ESCA16T_AF	OR1N1	Missense_Mutation
chr9	125391369	125391369	A	G	ESCA16T_AF	OR1B1	Missense_Mutation
chr9	127684159	127684159	C	A	ESCA18T_AF	GOLGA1	Nonsense_Mutation
chr9	128001747	128001747	G	A	ESCA15T_AF	HSPA5	Silent
chr9	129143435	129143435	A	G	ESCA16T_AF	MVB12B	Silent
chr9	130270479	130270479	T	C	ESCA14T_AF	NIBAN2	Missense_Mutation
chr9	130476161	130476161	G	T	ESCA01T_AF	CFAP157	Nonsense_Mutation
chr9	130828860	130828860	GGTCACTCA	-	ESCA16T_AF	NAIF1	Frame_Shift_Del
chr9	131261301	131261301	G	A	ESCA01T_AF	ODF2	Missense_Mutation
chr9	131467681	131467681	C	T	ESCA17T_AF	PKN3	Missense_Mutation
chr9	131505016	131505016	C	T	ESCA14T_AF	ZER1	Silent
chr9	131671042	131671042	-	AGCCCGTCG	ESCA07T_AF	LRRC8A	In_Frame_Ins
chr9	131671044	131671044	-	GCGGTTGTT	ESCA07T_AF	LRRC8A	Nonsense_Mutation
chr9	131698735	131698735	G	T	ESCA07T_AF	PHYHD1	Missense_Mutation
chr9	131768567	131768567	C	A	ESCA18T_AF	NUP188	Missense_Mutation
chr9	131939209	131939209	A	C	ESCA17T_AF	IER5L	Missense_Mutation
chr9	132662799	132662799	G	A	ESCA14T_AF	FNBP1	Missense_Mutation
chr9	133805293	133805293	C	A	ESCA19T_AF	FIBCD1	Silent
chr9	133924501	133924501	G	A	ESCA16T_AF	LAMC3	Silent
chr9	135205474	135205474	G	A	ESCA18T_AF	SETX	Missense_Mutation
chr9	135985753	135985753	C	T	ESCA16T_AF	RALGDS	Missense_Mutation
chr9	138441779	138441779	C	T	ESCA18T_AF	OBP2A	Missense_Mutation
chr9	138516347	138516347	A	-	ESCA16T_AF	GLT6D1	Frame_Shift_Del
chr9	138664786	138664786	-	CAAGGCAGC	ESCA16T_AF	KCNT1	Frame_Shift_Ins
chr9	139272274	139272274	G	A	ESCA15T_AF	SNAPC4	Silent
chr9	139369154	139369154	C	T	ESCA18T_AF	SEC16A	Missense_Mutation
chr9	139390677	139390677	G	A	ESCA19T_AF	NOTCH1	Missense_Mutation
chr9	139407953	139407953	GGTCCCACT	-	ESCA10T_AF	NOTCH1	Frame_Shift_Del
chr9	139410022	139410022	C	A	ESCA10T_AF	NOTCH1	Nonsense_Mutation
chr9	139925164	139925164	-	GGCCTGAGA	ESCA15T_AF	FUT7	Nonstop_Mutation
chr9	140904471	140904471	T	A	ESCA20T_AF	CACNA1B	Missense_Mutation
chr9	19516325	19516325	G	A	ESCA16T_AF	SLC24A2	Silent
chr9	20346502	20346502	G	A	ESCA15T_AF	MLLT3	Missense_Mutation
chr9	21971036	21971036	C	T	ESCA17T_AF	CDKN2A	Missense_Mutation
chr9	21971180	21971180	C	G	ESCA17T_AF	CDKN2A	Missense_Mutation
chr9	21974739	21974739	CCCGCAC	-	ESCA17T_AF	CDKN2A	Frame_Shift_Del
chr9	21974748	21974748	CC	-	ESCA17T_AF	CDKN2A	Frame_Shift_Del
chr9	27017045	27017045	-	TATGTTTGTT	ESCA10T_AF	IFT74	Nonsense_Mutation
chr9	312154	312154	-	CC	ESCA07T_AF	DOCK8	Frame_Shift_Ins

chr9	312156	312156	-	GTCCACTGA	ESCA07T_AF	DOCK8	Nonsense_Mutation
chr9	32457407	32457407	A	T	ESCA14T_AF	DDX58	Missense_Mutation
chr9	32541752	32541752	-	AAGTAAAGG	ESCA15T_AF	TOPORS	Frame_Shift_Ins
chr9	32541755	32541755	TCCTCCTTTA	-	ESCA15T_AF	TOPORS	Frame_Shift_Del
chr9	33338535	33338535	G	A	ESCA17T_AF	NFX1	Missense_Mutation
chr9	33442311	33442311	G	C	ESCA15T_AF	AQP3	Missense_Mutation
chr9	35044399	35044399	A	G	ESCA15T_AF	C9orf131	Missense_Mutation
chr9	35607615	35607615	C	A	ESCA19T_AF	TESK1	Silent
chr9	38424106	38424106	AG	-	ESCA07T_AF	IGFBPL1	Frame_Shift_Del
chr9	39118108	39118108	C	T	ESCA16T_AF	CNTNAP3	Silent
chr9	6814670	6814670	G	A	ESCA15T_AF	KDM4C	Silent
chr9	7011746	7011746	C	G	ESCA07T_AF	KDM4C	Missense_Mutation
chr9	711643	711643	G	-	ESCA15T_AF	KANK1	Frame_Shift_Del
chr9	72755078	72755078	G	A	ESCA19T_AF	MAMDC2	Missense_Mutation
chr9	74360209	74360209	G	C	ESCA16T_AF	CEMIP2	Silent
chr9	77390803	77390803	T	C	ESCA07T_AF	TRPM6	Silent
chr9	77411799	77411799	C	G	ESCA19T_AF	TRPM6	Missense_Mutation
chr9	77684010	77684010	-	CAAGGGTCT	ESCA14T_AF	NMRK1	Frame_Shift_Ins
chr9	78710958	78710958	A	T	ESCA07T_AF	PCSK5	Missense_Mutation
chr9	78710960	78710960	-	ACACTCTTCC	ESCA07T_AF	PCSK5	In_Frame_Ins
chr9	78854055	78854055	-	TCT	ESCA14T_AF	PCSK5	In_Frame_Ins
chr9	78854056	78854056	-	ACTTCTGGC.	ESCA14T_AF	PCSK5	In_Frame_Ins
chr9	79321262	79321262	G	A	ESCA15T_AF	PRUNE2	Silent
chr9	8436683	8436683	G	T	ESCA16T_AF	PTPRD	Missense_Mutation
chr9	84607763	84607763	C	T	ESCA16T_AF	SPATA31D1	Missense_Mutation
chr9	8521425	8521425	C	A	ESCA20T_AF	PTPRD	Missense_Mutation
chr9	85640700	85640700	C	T	ESCA16T_AF	RASEF	Missense_Mutation
chr9	86280040	86280040	T	C	ESCA19T_AF	UBQLN1	Silent
chr9	88923441	88923441	C	T	ESCA20T_AF	TUT7	Silent
chr9	90501528	90501528	G	A	ESCA19T_AF	SPATA31E1	Missense_Mutation
chr9	95077460	95077460	G	A	ESCA01T_AF	NOL8	Missense_Mutation
chr9	95477736	95477736	-	CCACAGGTG	ESCA10T_AF	BICD2	Frame_Shift_Ins
chr9	97062852	97062852	A	T	ESCA16T_AF	ZNF169	Missense_Mutation
chr9	97062981	97062981	C	T	ESCA16T_AF	ZNF169	Missense_Mutation
chr9	97333813	97333813	C	T	ESCA14T_AF	FBP2	Silent
chr9	977413	977413	G	T	ESCA17T_AF	DMRT3	Nonsense_Mutation
chr9	98678075	98678075	-	TGTTGTTCTC	ESCA15T_AF	ERCC6L2	Frame_Shift_Ins
chrX	101092511	101092511	C	A	ESCA17T_AF	NXF5	Missense_Mutation
chrX	101395887	101395887	C	T	ESCA15T_AF	TCEAL6	Missense_Mutation
chrX	101910156	101910156	T	-	ESCA07T_AF	GPRASP1	Frame_Shift_Del
chrX	101910357	101910357	G	A	ESCA15T_AF	GPRASP1	Missense_Mutation
chrX	102841838	102841838	GAGGGAGA	-	ESCA07T_AF	TCEAL4	In_Frame_Del
chrX	102842081	102842081	G	A	ESCA15T_AF	TCEAL4	Missense_Mutation
chrX	103267865	103267865	C	T	ESCA16T_AF	H2BFWT	Missense_Mutation
chrX	103268214	103268214	G	C	ESCA16T_AF	H2BFWT	Missense_Mutation
chrX	10437779	10437779	C	T	ESCA15T_AF	MID1	Missense_Mutation
chrX	104464950	104464950	C	A	ESCA14T_AF	TEX13A	Missense_Mutation
chrX	104464953	104464953	-	TGGGAGAAC	ESCA14T_AF	TEX13A	Frame_Shift_Ins
chrX	105277491	105277491	-	GGAAGCG	ESCA15T_AF	SERPINA7	Nonstop_Mutation
chrX	105450441	105450441	ATTT	-	ESCA16T_AF	PWWP3B	Frame_Shift_Del
chrX	105855896	105855896	G	A	ESCA14T_AF	RADX	Missense_Mutation
chrX	108625398	108625398	T	C	ESCA20T_AF	GUCY2F	Silent
chrX	110653588	110653588	C	G	ESCA15T_AF	DCX	Missense_Mutation
chrX	11139112	11139112	-	TGAAACAAC	ESCA07T_AF	HCCS	Frame_Shift_Ins
chrX	11139765	11139765	C	T	ESCA15T_AF	HCCS	Silent
chrX	111698613	111698613	T	C	ESCA16T_AF	RTL4	Silent
chrX	117528056	117528056	G	A	ESCA16T_AF	WDR44	Missense_Mutation
chrX	118724706	118724706	C	T	ESCA19T_AF	NKRF	Missense_Mutation

chrX	118892888	118892888 G	C	ESCA16T_AF	SOWAHD	Silent
chrX	118893302	118893302 -	GCAGGTAGT	ESCA14T_AF	SOWAHD	Frame_Shift_Ins
chrX	119389042	119389042 C	G	ESCA18T_AF	ZBTB33	Missense_Mutation
chrX	128615109	128615109 G	T	ESCA15T_AF	SMARCA1	Nonsense_Mutation
chrX	128878004	128878004 A	G	ESCA16T_AF	XPNPEP2	Missense_Mutation
chrX	129148216	129148216 C	T	ESCA15T_AF	BCORL1	Missense_Mutation
chrX	130409189	130409189 G	A	ESCA15T_AF	IGSF1	Nonsense_Mutation
chrX	132092543	132092543 G	A	ESCA15T_AF	HS6ST2	Missense_Mutation
chrX	133700674	133700674 G	C	ESCA14T_AF	PLAC1	Silent
chrX	133981485	133981485 -	GATGGAAGA	ESCA19T_AF	FAM122C	Nonsense_Mutation
chrX	135428486	135428486 C	G	ESCA16T_AF	ADGRG4	Nonsense_Mutation
chrX	13645366	13645366 -	TCCTTGATA(	ESCA10T_AF	EGFL6	Frame_Shift_Ins
chrX	13681115	13681115 C	T	ESCA16T_AF	TCEANC	Missense_Mutation
chrX	139038443	139038443 G	A	ESCA16T_AF	CXorf66	Missense_Mutation
chrX	140967165	140967165 T	C	ESCA16T_AF	MAGEC3	Missense_Mutation
chrX	140967195	140967195 T	A	ESCA16T_AF	MAGEC3	Missense_Mutation
chrX	140984845	140984845 G	T	ESCA16T_AF	MAGEC3	Missense_Mutation
chrX	142967640	142967640 G	T	ESCA17T_AF	UBE2NL	Missense_Mutation
chrX	148564462	148564462 A	G	ESCA15T_AF	IDS	Missense_Mutation
chrX	151092653	151092653 G	A	ESCA16T_AF	MAGEA4	Missense_Mutation
chrX	151424291	151424291 C	A	ESCA19T_AF	GABRA3	Silent
chrX	151869410	151869410 G	A	ESCA18T_AF	MAGEA6	Missense_Mutation
chrX	152018959	152018959 -	TGGTCCATTA	ESCA14T_AF	NSDHL	Frame_Shift_Ins
chrX	152086660	152086660 C	T	ESCA14T_AF	ZNF185	Silent
chrX	152613037	152613037 C	T	ESCA07T_AF	ZNF275	Silent
chrX	152814301	152814301 -	GACAGAGTA	ESCA14T_AF	ATP2B3	Nonsense_Mutation
chrX	152823728	152823728 G	C	ESCA16T_AF	ATP2B3	Silent
chrX	153006076	153006076 C	T	ESCA14T_AF	ABCD1	Silent
chrX	153037731	153037731 -	GACCTGGTA	ESCA16T_AF	PLXNB3	Nonsense_Mutation
chrX	153052270	153052270 C	T	ESCA10T_AF	IDH3G	Missense_Mutation
chrX	153134316	153134316 C	T	ESCA07T_AF	L1CAM	Silent
chrX	153581802	153581802 -	ACGACTCCA	ESCA15T_AF	FLNA	Frame_Shift_Ins
chrX	153590620	153590620 G	A	ESCA16T_AF	FLNA	Silent
chrX	15415583	15415583 C	T	ESCA16T_AF	PIR	Silent
chrX	154448561	154448561 C	G	ESCA15T_AF	VBP1	Silent
chrX	16168677	16168677 T	C	ESCA16T_AF	GRPR	Silent
chrX	16887719	16887719 -	TGATGGGGA	ESCA14T_AF	RBBP7	In_Frame_Ins
chrX	18230715	18230715 A	C	ESCA16T_AF	BEND2	Missense_Mutation
chrX	18264700	18264700 C	T	ESCA15T_AF	SCML2	Missense_Mutation
chrX	18919682	18919682 G	C	ESCA19T_AF	PHKA2	Missense_Mutation
chrX	21581435	21581435 C	A	ESCA15T_AF	CNKSR2	Missense_Mutation
chrX	21670620	21670620 -	CATTTAGAC.	ESCA16T_AF	CNKSR2	Nonsense_Mutation
chrX	22291732	22291732 C	T	ESCA16T_AF	CBLL2	Silent
chrX	24381387	24381387 G	C	ESCA16T_AF	SUPT20HL1	Silent
chrX	25031656	25031656 G	A	ESCA15T_AF	ARX	Silent
chrX	25031671	25031671 T	C	ESCA15T_AF	ARX	Silent
chrX	27766045	27766045 A	G	ESCA16T_AF	DCAF8L2	Missense_Mutation
chrX	27766776	27766776 G	A	ESCA16T_AF	DCAF8L2	Silent
chrX	27997735	27997735 C	G	ESCA15T_AF	DCAF8L1	Missense_Mutation
chrX	2825403	2825403 A	G	ESCA16T_AF	ARSD	Missense_Mutation
chrX	2861237	2861237 C	T	ESCA16T_AF	ARSE	Missense_Mutation
chrX	2861239	2861239 -	TCTTTGCAG(	ESCA16T_AF	ARSE	Frame_Shift_Ins
chrX	2947386	2947386 C	T	ESCA19T_AF	ARSH	Missense_Mutation
chrX	30237145	30237145 C	A	ESCA15T_AF	MAGEB2	Missense_Mutation
chrX	30254522	30254522 G	A	ESCA18T_AF	MAGEB3	Missense_Mutation
chrX	3239979	3239979 G	A	ESCA16T_AF	MXRA5	Silent
chrX	3592725	3592725 G	A	ESCA16T_AF	PRKX	Silent
chrX	35938045	35938045 C	T	ESCA16T_AF	CFAP47	Silent

chrX	36379467	36379467	C	A	ESCA07T_AF	CFAP47	Missense_Mutation
chrX	37518861	37518861	G	A	ESCA17T_AF	LANCL3	Missense_Mutation
chrX	40522351	40522351	-	TCCACCTCG	ESCA10T_AF	MED14	In_Frame_Ins
chrX	40522353	40522353	-	GCTCTTGGG	ESCA10T_AF	MED14	In_Frame_Ins
chrX	41333166	41333166	C	T	ESCA01T_AF	NYX	Missense_Mutation
chrX	41333769	41333769	G	T	ESCA18T_AF	NYX	Nonsense_Mutation
chrX	41555571	41555571	C	T	ESCA14T_AF	GPR34	Silent
chrX	47073960	47073960	A	C	ESCA14T_AF	UBA1	Missense_Mutation
chrX	48337063	48337063	-	GTGTGGTAC	ESCA10T_AF	FTSJ1	Nonsense_Mutation
chrX	48463374	48463374	-	ACGA	ESCA14T_AF	WDR13	Frame_Shift_Ins
chrX	48544134	48544134	G	A	ESCA16T_AF	WAS	Silent
chrX	49104751	49104751	-	CCAAC	ESCA07T_AF	CCDC22	Frame_Shift_Ins
chrX	49104763	49104763	A	C	ESCA16T_AF	CCDC22	Missense_Mutation
chrX	50052963	50052963	G	A	ESCA19T_AF	CCNB3	Missense_Mutation
chrX	53589884	53589884	-	AGTGAGCAC	ESCA07T_AF	HUWE1	Nonsense_Mutation
chrX	54975508	54975508	C	T	ESCA16T_AF	PFKFB1	Silent
chrX	64721937	64721937	T	A	ESCA07T_AF	ZC3H12B	Silent
chrX	65242195	65242195	G	A	ESCA16T_AF	VSIG4	Silent
chrX	69615635	69615635	-	A	ESCA16T_AF	KIF4A	Frame_Shift_Ins
chrX	6975752	6975752	G	T	ESCA16T_AF	PUDP	Missense_Mutation
chrX	70146475	70146475	G	C	ESCA16T_AF	SLC7A3	Missense_Mutation
chrX	70354683	70354683	G	A	ESCA15T_AF	MED12	Silent
chrX	70836337	70836337	T	C	ESCA07T_AF	CXCR3	Missense_Mutation
chrX	70836342	70836342	-	GCTCTATGC	ESCA07T_AF	CXCR3	Nonsense_Mutation
chrX	72432966	72432966	C	T	ESCA16T_AF	NAP1L2	Missense_Mutation
chrX	73963188	73963188	C	T	ESCA15T_AF	NEXMIF	Missense_Mutation
chrX	74494462	74494462	A	C	ESCA10T_AF	UPRT	Missense_Mutation
chrX	74494464	74494464	-	CACTTCCCT	ESCA10T_AF	UPRT	Nonsense_Mutation
chrX	78618183	78618183	C	G	ESCA16T_AF	ITM2A	Missense_Mutation
chrX	85404095	85404095	-	TGGCATTGG	ESCA14T_AF	DACH2	Nonsense_Mutation
chrX	85404097	85404097	-	ACAAAGTTT	ESCA14T_AF	DACH2	Frame_Shift_Ins
chrX	86069824	86069824	G	T	ESCA15T_AF	DACH2	Silent
chrX	9905342	9905342	A	G	ESCA16T_AF	SHROOM2	Silent
chrX	9914947	9914947	G	C	ESCA16T_AF	SHROOM2	Missense_Mutation
chrX	99658661	99658661	-	CCTTTGCAG	ESCA19T_AF	PCDH19	Nonsense_Mutation
