# Supplementary material for: Genomic and transcriptional characterization of early esophageal squamous cell carcinoma
Source: BMC Med Genomics. 2023 Jul 1;16:153. doi: 10.1186/s12920-023-01588-7 (PMC10315050; doi:10.1186/s12920-023-01588-7)
Supplement: Supplementary file 3 — Additional file 3: Table S2. The DEGs between tumor and normal. [file 12920_2023_1588_MOESM3_ESM.pdf]

Table S2: The DEGs between tumor (T) and normal (N)

Gene_id	Symbol	Mean_N	Mean_T	FC_TvsN	P_TTEST	type
ENSG0000(	DZIP1L	0.2813	1.665	4.628901	5.49E-10	T
ENSG0000(	SLC35C1	15.6502	4.49	0.291425	1.36E-09	N
ENSG0000(	PHF5A	11.9371	32.3653	2.697103	2.5E-09	T
ENSG0000(	CIPC	10.2151	4.5308	0.448934	2.83E-09	N
ENSG0000(	GCFC2	1.7925	4.3686	2.361215	3.02E-09	T
ENSG0000(	SGO2	0.7583	3.464	4.152394	4.12E-09	T
ENSG0000(	ILKAP	2.1282	4.9243	2.254869	7.29E-09	T
ENSG0000(	SNAPC1	0.9574	4.3795	4.236334	7.66E-09	T
ENSG0000(	UFD1	2.9628	6.9737	2.309553	1.7E-08	T
ENSG0000(	CEACAM5	260.2346	96.6047	0.371463	1.94E-08	N
ENSG0000(	ACAP2-IT	0.6804	4.7961	6.273834	2.01E-08	T
ENSG0000(	UBE2E3	8.0063	19.2129	2.382456	2.1E-08	T
ENSG0000(	BLNK	20.3066	6.2057	0.309003	2.22E-08	N
ENSG0000(	TRA2B	8.191	21.7078	2.630298	2.25E-08	T
ENSG0000(	ZGRF1	0.5453	2.113	3.429413	2.72E-08	T
ENSG0000(	TBPL1	1.3968	3.3614	2.312533	3.17E-08	T
ENSG0000(	PM20D2	2.6126	6.2842	2.353535	3.62E-08	T
ENSG0000(	CCDC18	0.7332	2.3388	2.927028	4.41E-08	T
ENSG0000(	RAB5B	52.4613	23.2709	0.444641	4.81E-08	N
ENSG0000(	CYSRT1	246.3219	27.8841	0.113562	6.79E-08	N
ENSG0000(	FAM3D	140.8725	46.0519	0.327382	7.09E-08	N
ENSG0000(	SH3PXD2A	15.3001	3.5986	0.240167	7.54E-08	N
ENSG0000(	GALE	13.4416	4.8006	0.361892	7.83E-08	N
ENSG0000(	SNRPG	7.7583	22.3025	2.850807	8.03E-08	T
ENSG0000(	PAX9	71.1835	23.5683	0.332031	8.86E-08	N
ENSG0000(	IL17RC	6.6105	3.0649	0.471634	9.52E-08	N
ENSG0000(	TIA1	3.6587	12.0963	3.244819	9.66E-08	T
ENSG0000(	TASP1	2.0653	6.6671	3.125248	9.66E-08	T
ENSG0000(	CHEK2	0.8895	3.0907	3.224558	1.01E-07	T
ENSG0000(	THADA	1.521	4.3711	2.758236	1.09E-07	T
ENSG0000(	LSM2	5.2461	15.2676	2.874544	1.16E-07	T
ENSG0000(	GPX3	110.443	20.2272	0.183885	1.24E-07	N
ENSG0000(	PDLIM2	6.6871	1.9924	0.308291	1.37E-07	N
ENSG0000(	KRT8P12	0.7108	2.7496	3.514554	1.42E-07	T
ENSG0000(	GMCL1	4.1163	9.4489	2.264758	1.48E-07	T
ENSG0000(	CCDC138	0.9447	2.7387	2.717239	1.66E-07	T
ENSG0000(	RMND5B	12.57	4.3965	0.354893	1.68E-07	N
ENSG0000(	TNFAIP8L	12.7462	2.0127	0.164461	1.75E-07	N
ENSG0000(	WDR91	3.0438	6.7853	2.19012	1.76E-07	T
ENSG0000(	MCM9	1.0574	2.337	2.105581	1.9E-07	T
ENSG0000(	EHF	188.3683	74.958	0.398253	1.9E-07	N
ENSG0000(	ZRANB3	0.3014	0.8466	2.358246	1.95E-07	T
ENSG0000(	EVPL	103.3721	27.4069	0.265839	2.13E-07	N
ENSG0000(	POT1	1.5058	4.5684	2.907211	2.16E-07	T
ENSG0000(	MED30	2.8868	8.5773	2.905216	2.25E-07	T
ENSG0000(	NMRK1	10.185	3.3073	0.331288	2.34E-07	N
ENSG0000(	ZNRF3	1.5754	3.6576	2.242808	2.55E-07	T
ENSG0000(	TPRN	4.9551	2.1123	0.437637	2.55E-07	N
ENSG0000(	KRT4	10612.11	1420.203	0.133837	2.69E-07	N
ENSG0000(	SIRT5	1.3762	2.862	2.006503	2.83E-07	T
ENSG0000(	EPB41L3	7.9	1.8753	0.246913	2.93E-07	N
ENSG0000(	POLE2	1.0716	4.69	4.088426	3.01E-07	T
ENSG0000(	RNF208	12.6347	3.3539	0.27122	3.17E-07	N
ENSG0000(	SHCBP1	0.6534	3.816	5.19777	3.2E-07	T
ENSG0000(	C1orf116	129.7068	27.8892	0.215622	3.34E-07	N
ENSG0000(	GGH	4.3915	15.0727	3.378092	3.44E-07	T

ENSG0000(	IFT80	1.0376	2.7752	2.527426	3.64E-07	T
ENSG0000(	FARS2	1.3126	3.1352	2.290245	3.75E-07	T
ENSG0000(	RPL23AP3	0.3903	1.68	3.63043	3.89E-07	T
ENSG0000(	NSL1	1.6658	4.5772	2.648771	4.25E-07	T
ENSG0000(	PEA15	97.9882	46.6426	0.476536	4.3E-07	N
ENSG0000(	DEPDC1B	0.4251	3.109	6.111217	4.56E-07	T
ENSG0000(	ORC3	2.5905	7.2538	2.733247	4.56E-07	T
ENSG0000(	PBK	1.7137	7.2689	4.06291	4.62E-07	T
ENSG0000(	ESCO2	0.4779	2.0804	3.772971	4.65E-07	T
ENSG0000(	ZNF131	2.5321	6.8354	2.63493	4.77E-07	T
ENSG0000(	ANXA1	2422.834	655.1118	0.270421	4.81E-07	N
ENSG0000(	SRSF11	6.5367	16.6031	2.516778	5.04E-07	T
ENSG0000(	UBL7-AS1	0.6331	1.7317	2.498568	5.15E-07	T
ENSG0000(	LYRM4	0.6805	1.6483	2.239974	5.16E-07	T
ENSG0000(	GMNN	1.7727	7.9665	4.307417	5.19E-07	T
ENSG0000(	INTS3	3.1	7.2627	2.300844	5.36E-07	T
ENSG0000(	ATP6V1E2	0.2816	0.8841	2.578878	5.39E-07	T
ENSG0000(	SLC23A2	2.0228	5.9399	2.845252	5.46E-07	T
ENSG0000(	CCDC57	0.5168	1.8498	3.161154	5.57E-07	T
ENSG0000(	BBOX1-AS	0.4022	5.7137	11.57646	5.71E-07	T
ENSG0000(	C15orf62	21.0167	2.1197	0.105116	5.86E-07	N
ENSG0000(	DNAJC19	3.1228	9.035	2.834492	6.19E-07	T
ENSG0000(	CLIC3	168.5923	27.3242	0.162569	6.19E-07	N
ENSG0000(	TCP1P1	0.079	0.3242	2.369832	6.57E-07	T
ENSG0000(	MORC4	2.1062	4.7555	2.200843	6.91E-07	T
ENSG0000(	ERBB3	25.1168	10.7275	0.429376	7.42E-07	N
ENSG0000(	S100A14	1171.611	302.7253	0.258447	7.55E-07	N
ENSG0000(	SF3B1	14.0592	35.039	2.481708	7.66E-07	T
ENSG0000(	SRBD1	2.0046	4.5157	2.193148	7.87E-07	T
ENSG0000(	TMEM267	1.58	4.7489	2.88625	7.94E-07	T
ENSG0000(	AL359183.	0.0611	0.3134	2.566108	7.95E-07	T
ENSG0000(	NCK1-DT	0.4438	2.3937	4.585693	7.99E-07	T
ENSG0000(	CDK6	4.0969	13.6996	3.288046	8.11E-07	T
ENSG0000(	THOC5	1.4804	4.2779	2.770121	8.54E-07	T
ENSG0000(	NUP35	2.0165	5.657	2.720057	8.69E-07	T
ENSG0000(	SELENOP	21.461	48.5504	2.256407	8.84E-07	T
ENSG0000(	PRKAA1	6.4449	15.1336	2.327553	9.1E-07	T
ENSG0000(	CAPN5	24.1489	4.1591	0.175641	9.3E-07	N
ENSG0000(	PHIP	4.4566	11.7618	2.603213	9.52E-07	T
ENSG0000(	PLEKHA6	10.0145	2.6215	0.269069	9.53E-07	N
ENSG0000(	RIOK1	3.8813	13.2654	3.357044	1.02E-06	T
ENSG0000(	WDR75	3.876	11.116	2.820926	1.03E-06	T
ENSG0000(	VAT1	110.6339	22.8864	0.207582	1.04E-06	N
ENSG0000(	BNIPL	41.0687	9.528	0.233867	1.05E-06	N
ENSG0000(	CKAP2	4.1212	10.6	2.534824	1.05E-06	T
ENSG0000(	TANGO2	1.1485	2.7843	2.310212	1.09E-06	T
ENSG0000(	CEP131	0.6816	1.9982	2.684493	1.1E-06	T
ENSG0000(	RANBP1	5.5829	19.223	3.400201	1.11E-06	T
ENSG0000(	PRSS3	45.6751	9.2225	0.203659	1.13E-06	N
ENSG0000(	RCN2	2.4562	7.0781	2.808114	1.13E-06	T
ENSG0000(	R3HDM1	2.9271	7.6956	2.57527	1.14E-06	T
ENSG0000(	GPATCH2	1.31	3.3642	2.456879	1.16E-06	T
ENSG0000(	PRPF4B	7.0668	14.3089	2.010507	1.21E-06	T
ENSG0000(	RNF2	3.3207	8.2498	2.440962	1.21E-06	T
ENSG0000(	LINC0256(	52.0536	9.2004	0.178327	1.21E-06	N
ENSG0000(	SNORA72	5.2645	32.6409	6.103253	1.22E-06	T
ENSG0000(	NUP62CL	0.2725	1.7613	4.996779	1.26E-06	T
ENSG0000(	SRSF3	16.7654	40.1816	2.388417	1.33E-06	T

ENSG0000(	NCAPH2	3.2538	9.5588	2.879957	1.35E-06	T
ENSG0000(	NMD3	7.8169	19.8525	2.520242	1.35E-06	T
ENSG0000(	A2ML1	415.9638	77.1375	0.185639	1.39E-06	N
ENSG0000(	MRPS28	1.5872	4.652	2.816501	1.4E-06	T
ENSG0000(	GMDS	13.1937	4.1182	0.317308	1.42E-06	N
ENSG0000(	NCCRP1	615.2685	86.4276	0.140611	1.44E-06	N
ENSG0000(	GMPS	4.7145	15.7131	3.284474	1.47E-06	T
ENSG0000(	NSUN6	1.3135	3.0771	2.247683	1.55E-06	T
ENSG0000(	SHROOM3	16.3498	2.3313	0.147801	1.55E-06	N
ENSG0000(	SLC25A23	27.4254	11.3102	0.414533	1.55E-06	N
ENSG0000(	STK3	1.394	4.3736	2.994378	1.55E-06	T
ENSG0000(	SKA3	1.8594	5.1639	2.686486	1.59E-06	T
ENSG0000(	EPS8L2	74.5554	20.3421	0.273819	1.59E-06	N
ENSG0000(	ODR4	3.394	7.2404	2.100859	1.6E-06	T
ENSG0000(	GSN	48.0096	16.7707	0.350672	1.61E-06	N
ENSG0000(	KIAA1841	0.865	1.8706	2.042073	1.63E-06	T
ENSG0000(	CFAP20	4.1082	9.4955	2.280191	1.69E-06	T
ENSG0000(	SORT1	52.0564	14.8722	0.287064	1.7E-06	N
ENSG0000(	NPEPPS	25.5194	12.5041	0.491975	1.7E-06	N
ENSG0000(	AFMID	1.7336	3.7964	2.125	1.73E-06	T
ENSG0000(	ZNF823	6.5511	2.983	0.463532	1.79E-06	N
ENSG0000(	KIAA0232	15.7726	6.8871	0.440199	1.79E-06	N
ENSG0000(	PHF20L1	2.4448	5.8001	2.318493	1.8E-06	T
ENSG0000(	SAMM50	2.2356	5.726	2.494434	1.89E-06	T
ENSG0000(	KNL1	0.9018	5.2018	5.292274	1.9E-06	T
ENSG0000(	DROSHA	4.2214	8.9448	2.093025	1.93E-06	T
ENSG0000(	SERPINB6	7.9178	3.1089	0.400222	1.95E-06	N
ENSG0000(	WDR12	1.5372	4.0339	2.524982	1.96E-06	T
ENSG0000(	SNX5	4.5572	13.1809	2.851692	1.96E-06	T
ENSG0000(	LCLAT1	2.8656	8.5064	2.902077	1.96E-06	T
ENSG0000(	EZH2	3.2152	8.6329	2.6342	1.96E-06	T
ENSG0000(	FXR1	3.6826	12.3771	3.298551	1.97E-06	T
ENSG0000(	TIPIN	0.8389	2.5489	2.82128	2E-06	T
ENSG0000(	RBL1	1.7635	7.6281	4.147089	2.02E-06	T
ENSG0000(	BMS1P9	0.8688	0.1751	0.28396	2.04E-06	N
ENSG0000(	SRSF6	15.3596	42.01	2.723874	2.04E-06	T
ENSG0000(	CEP95	1.6189	3.9562	2.359765	2.05E-06	T
ENSG0000(	ITGAV	7.212	28.4315	3.90201	2.07E-06	T
ENSG0000(	PSMC3IP	0.3937	1.6295	3.50314	2.07E-06	T
ENSG0000(	AC027373	0.4372	3.6034	6.893894	2.07E-06	T
ENSG0000(	MYO5B	17.9136	6.3152	0.356131	2.14E-06	N
ENSG0000(	ARL6IP6	2.4964	6.5022	2.542829	2.17E-06	T
ENSG0000(	SOX4	3.7302	23.197	6.08245	2.18E-06	T
ENSG0000(	CDKAL1	3.2197	6.957	2.125794	2.2E-06	T
ENSG0000(	HIST1H4D	122.4589	380.5709	3.106024	2.21E-06	T
ENSG0000(	C16orf91	2.7985	6.2035	2.174746	2.24E-06	T
ENSG0000(	XRN2	9.26	30.1223	3.228878	2.24E-06	T
ENSG0000(	DRG1	10.9361	22.3549	2.034677	2.27E-06	T
ENSG0000(	TFB2M	3.0959	9.0295	2.856629	2.3E-06	T
ENSG0000(	PES1	4.2915	12.4348	2.854332	2.31E-06	T
ENSG0000(	KIF15	0.8866	3.3354	3.48206	2.38E-06	T
ENSG0000(	DPM3	11.6855	23.6127	2.012023	2.39E-06	T
ENSG0000(	MAP3K2	4.9668	11.6843	2.325787	2.39E-06	T
ENSG0000(	MTFR2	0.3244	2.3357	5.739161	2.4E-06	T
ENSG0000(	CKS2	8.7942	27.3643	3.087889	2.4E-06	T
ENSG0000(	WDPCP	0.3089	1.4175	3.711176	2.42E-06	T
ENSG0000(	POC1A	0.7642	2.7561	3.304906	2.44E-06	T
ENSG0000(	MAL	3023.075	143.7026	0.047567	2.45E-06	N

ENSG0000(	DBNDD1	7.3724	2.6432	0.367111	2.48E-06	N
ENSG0000(	MEX3D	6.2748	13.8331	2.185653	2.5E-06	T
ENSG0000(	B3GLCT	1.3479	3.2934	2.34367	2.5E-06	T
ENSG0000(	SLC25A17	1.7404	4.8416	2.685068	2.51E-06	T
ENSG0000(	THSD1	0.6876	2.4806	3.276536	2.53E-06	T
ENSG0000(	ZNF185	238.5244	69.0551	0.289807	2.62E-06	N
ENSG0000(	CSTB	3146.405	547.5405	0.174047	2.63E-06	N
ENSG0000(	WDR70	1.5296	3.8152	2.402553	2.66E-06	T
ENSG0000(	PON2	4.0328	10.6622	2.604094	2.71E-06	T
ENSG0000(	STMN1	5.5877	26.9534	4.756474	2.84E-06	T
ENSG0000(	PTCD3	2.4868	6.913	2.711072	2.85E-06	T
ENSG0000(	LIMK2	9.0312	20.858	2.295208	2.86E-06	T
ENSG0000(	FLG-AS1	2.1284	0.537	0.285855	2.87E-06	N
ENSG0000(	TMEM230	11.8996	29.7423	2.486941	2.88E-06	T
ENSG0000(	IRX2	1.0697	3.0832	2.721382	2.88E-06	T
ENSG0000(	DXO	1.453	3.7642	2.488216	2.89E-06	T
ENSG0000(	GNB5	0.5434	1.4324	2.381722	2.89E-06	T
ENSG0000(	CNTROB	1.5838	4.1875	2.546324	2.92E-06	T
ENSG0000(	HIST1H4C	78.3838	236.9648	3.020557	2.93E-06	T
ENSG0000(	GPRIN1	0.1538	1.3408	5.676911	2.94E-06	T
ENSG0000(	EMP1	1383.923	189.3526	0.136885	2.94E-06	N
ENSG0000(	POLG2	0.7757	2.2376	2.669407	2.98E-06	T
ENSG0000(	UNC13B	5.6436	2.5934	0.468939	2.98E-06	N
ENSG0000(	HSPE1	5.1186	16.9604	3.269153	3E-06	T
ENSG0000(	SELENOT	14.9612	36.8476	2.453164	3E-06	T
ENSG0000(	SIM2	11.0216	2.4444	0.22878	3.02E-06	N
ENSG0000(	SLC45A4	1.7389	5.2411	2.904508	3.03E-06	T
ENSG0000(	TTC5	0.8394	2.1225	2.365872	3.05E-06	T
ENSG0000(	ASPG	6.3954	1.7276	0.281368	3.13E-06	N
ENSG0000(	ZDHHC13	18.6052	8.1288	0.43992	3.14E-06	N
ENSG0000(	HJURP	0.9841	4.8779	4.591735	3.16E-06	T
ENSG0000(	AUP1	21.921	48.843	2.22256	3.2E-06	T
ENSG0000(	NRM	2.5282	7.7843	2.999886	3.23E-06	T
ENSG0000(	SLC46A2	1.4098	0.1546	0.168632	3.28E-06	N
ENSG0000(	IRF2BP2	26.4348	61.1682	2.308975	3.36E-06	T
ENSG0000(	PAPOLG	2.0691	4.6868	2.206814	3.38E-06	T
ENSG0000(	PPL	630.4936	94.9268	0.150694	3.44E-06	N
ENSG0000(	KIF18B	0.8841	4.2955	4.466518	3.46E-06	T
ENSG0000(	ZBTB40-IT	0.3179	1.3811	3.544149	3.48E-06	T
ENSG0000(	LINC0137(	0.1172	0.9922	5.028545	3.52E-06	T
ENSG0000(	CDCA2	1.0132	4.3279	3.977632	3.55E-06	T
ENSG0000(	TGM3	2812.482	383.8259	0.136503	3.6E-06	N
ENSG0000(	FUCA2	3.7526	13.0003	3.400379	3.66E-06	T
ENSG0000(	CENPM	0.9602	4.9915	4.802396	3.67E-06	T
ENSG0000(	MDC1	1.2894	4.2638	3.14078	3.67E-06	T
ENSG0000(	COBL	2.7936	0.7617	0.297795	3.68E-06	N
ENSG0000(	TTC21B	1.2202	3.1934	2.494622	3.69E-06	T
ENSG0000(	FBXO4	1.349	3.2513	2.312836	3.73E-06	T
ENSG0000(	TRMT11	1.2615	3.6924	2.785457	3.75E-06	T
ENSG0000(	STK39	30.523	13.0834	0.430506	3.77E-06	N
ENSG0000(	ARMC2	0.3121	0.8323	2.262315	3.83E-06	T
ENSG0000(	C1orf112	0.6738	2.6998	3.618248	3.96E-06	T
ENSG0000(	TAF2	4.9076	12.9598	2.607996	4.03E-06	T
ENSG0000(	BLM	0.2906	2.1439	5.744752	4.08E-06	T
ENSG0000(	TUSC3	2.3741	9.3873	3.834647	4.1E-06	T
ENSG0000(	TARBP1	2.296	7.2734	3.077379	4.15E-06	T
ENSG0000(	DHX36	3.187	12.9907	3.982568	4.19E-06	T
ENSG0000(	HIST1H1B	131.8609	494.5134	3.748181	4.21E-06	T

ENSG0000(	TTC22	41.1817	13.464	0.328572	4.29E-06	N
ENSG0000(	MORC2	2.4341	7.8631	3.142378	4.33E-06	T
ENSG0000(	LINC0203(	1.2014	0.1409	0.185108	4.34E-06	N
ENSG0000(	CIAO2A	5.13	14.4948	2.790593	4.39E-06	T
ENSG0000(	KAT2B	30.1727	7.5459	0.252567	4.44E-06	N
ENSG0000(	SRSF10	3.8612	9.8766	2.51858	4.47E-06	T
ENSG0000(	OCLN	9.5572	3.296	0.351655	4.47E-06	N
ENSG0000(	ATP2C1	6.5944	20.6486	3.099397	4.48E-06	T
ENSG0000(	ZNF639	1.9405	6.4343	3.202303	4.49E-06	T
ENSG0000(	WNT7B	3.4484	16.6296	4.714688	4.5E-06	T
ENSG0000(	ZNF81	0.8945	2.1963	2.308999	4.52E-06	T
ENSG0000(	TMEM67	0.5016	2.6731	4.609541	4.53E-06	T
ENSG0000(	AGFG2	25.7515	5.0339	0.198592	4.65E-06	N
ENSG0000(	PTMA	129.3768	277.7588	2.146012	4.68E-06	T
ENSG0000(	RBM39	9.6115	23.3717	2.416897	4.68E-06	T
ENSG0000(	B3GAT3	1.6814	4.9959	2.860615	4.7E-06	T
ENSG0000(	PCDH1	33.2054	10.3196	0.31285	4.7E-06	N
ENSG0000(	NUF2	0.8263	6.0524	6.641909	4.71E-06	T
ENSG0000(	GTF3C3	2.947	7.8761	2.61769	4.76E-06	T
ENSG0000(	TP53I3	22.8875	6.4209	0.283672	4.78E-06	N
ENSG0000(	TP63	26.1726	105.9642	4.037065	4.81E-06	T
ENSG0000(	USP37	2.0151	4.1815	2.024254	4.86E-06	T
ENSG0000(	POP5	2.6463	6.8279	2.52263	4.91E-06	T
ENSG0000(	VPS8	3.3266	8.012	2.367361	4.92E-06	T
ENSG0000(	SMARCB1	1.6122	3.4688	2.084336	4.97E-06	T
ENSG0000(	IMMT	8.4573	19.6729	2.310647	5.04E-06	T
ENSG0000(	TMEM9	2.1627	6.6828	2.997658	5.11E-06	T
ENSG0000(	ATR	1.7674	7.0568	3.832494	5.17E-06	T
ENSG0000(	HSCB	1.5446	3.868	2.412745	5.21E-06	T
ENSG0000(	FAM89A	0.6432	4.8047	6.599435	5.22E-06	T
ENSG0000(	AC134407	0.1004	0.4	2.49501	5.23E-06	T
ENSG0000(	IGF2R	5.7072	12.0438	2.091163	5.34E-06	T
ENSG0000(	GDAP1	1.099	4.9251	4.191076	5.34E-06	T
ENSG0000(	PLK4	1.1099	4.2019	3.555583	5.35E-06	T
ENSG0000(	LIN9	1.2964	3.4408	2.535663	5.35E-06	T
ENSG0000(	AMMECR	3.4295	6.9855	2.007508	5.35E-06	T
ENSG0000(	MCTP2	1.5366	3.8349	2.404314	5.44E-06	T
ENSG0000(	MYO5A	1.6285	6.6621	3.91212	5.5E-06	T
ENSG0000(	AC117382	0.7134	3.4923	4.4164	5.57E-06	T
ENSG0000(	DHX57	1.2034	3.6206	2.854534	5.63E-06	T
ENSG0000(	CENPO	0.8978	3.7554	3.863901	5.65E-06	T
ENSG0000(	NEO1	3.8325	9.2272	2.371825	5.76E-06	T
ENSG0000(	PRSS27	79.6653	9.9186	0.125601	5.87E-06	N
ENSG0000(	SLC39A10	1.4903	3.6306	2.345847	5.95E-06	T
ENSG0000(	BLOC1S6	3.6154	8.3966	2.28686	5.96E-06	T
ENSG0000(	C17orf53	0.4353	2.8314	5.476182	6.07E-06	T
ENSG0000(	PAK1IP1	7.5801	16.2404	2.127629	6.08E-06	T
ENSG0000(	HSP90B2P	0.2052	0.961	3.476409	6.14E-06	T
ENSG0000(	NVL	1.5827	5.25	3.179414	6.16E-06	T
ENSG0000(	IFT52	3.2479	7.0857	2.146331	6.17E-06	T
ENSG0000(	ARV1	4.6148	13.5316	2.891236	6.17E-06	T
ENSG0000(	ASPM	2.0415	10.1082	4.766846	6.18E-06	T
ENSG0000(	CCNT2	3.7435	8.6062	2.265175	6.21E-06	T
ENSG0000(	GDPD3	19.3917	4.4121	0.231488	6.37E-06	N
ENSG0000(	SNORD94	9.9538	26.5699	2.652718	6.44E-06	T
ENSG0000(	AC079922	3.51	8.5931	2.408061	6.46E-06	T
ENSG0000(	PRPF40A	7.3439	16.6899	2.255525	6.48E-06	T
ENSG0000(	FLOT1	11.1301	25.6265	2.290852	6.52E-06	T

ENSG0000(	SMAGP	35.5561	15.5413	0.438671	6.53E-06	N
ENSG0000(	FAM129B	436.4122	78.6046	0.180303	6.6E-06	N
ENSG0000(	NUP85	2.2904	6.1768	2.625837	6.64E-06	T
ENSG0000(	MAP3K21	0.6664	2.2078	3.011221	6.66E-06	T
ENSG0000(	CNFN	3757.899	468.8674	0.124792	6.66E-06	N
ENSG0000(	SMIM13	2.1486	5.0445	2.287868	6.67E-06	T
ENSG0000(	TENT4A	2.7584	6.8361	2.426567	6.72E-06	T
ENSG0000(	LRRC40	4.1571	10.2398	2.428837	6.73E-06	T
ENSG0000(	VPS37B	30.3199	11.9944	0.397582	6.77E-06	N
ENSG0000(	RRM2	2.3649	14.0515	5.741207	6.84E-06	T
ENSG0000(	ITPRID2	8.9229	29.006	3.225792	6.84E-06	T
ENSG0000(	INTS7	3.1516	7.7056	2.400541	6.89E-06	T
ENSG0000(	PSMG3	3.78	9.6358	2.509227	6.96E-06	T
ENSG0000(	MIR548A(	1.366	6.0995	4.228854	7.02E-06	T
ENSG0000(	GALK2	1.3803	3.2728	2.278457	7.04E-06	T
ENSG0000(	MFHAS1	1.8642	9.7286	5.003869	7.1E-06	T
ENSG0000(	TCEA1P2	0.6236	2.3406	3.372858	7.13E-06	T
ENSG0000(	LRP10	75.2491	34.3842	0.457659	7.17E-06	N
ENSG0000(	RPS10P7	0.2392	0.7143	2.400649	7.17E-06	T
ENSG0000(	LSM5	2.4637	7.7472	3.060889	7.19E-06	T
ENSG0000(	RPP40	2.3529	5.2376	2.176037	7.19E-06	T
ENSG0000(	TMEM138	1.5147	4.7796	3.021986	7.21E-06	T
ENSG0000(	N4BP3	7.6029	2.4217	0.32737	7.24E-06	N
ENSG0000(	ST3GAL4	21.45	3.0703	0.147114	7.33E-06	N
ENSG0000(	VBP1	9.9221	22.4349	2.248521	7.35E-06	T
ENSG0000(	HIST1H2A	28.0035	95.3338	3.395798	7.48E-06	T
ENSG0000(	FNDC4	24.0715	2.0777	0.090094	7.48E-06	N
ENSG0000(	CDC5L	4.7363	9.6316	2.012199	7.5E-06	T
ENSG0000(	PTK7	2.6904	8.0361	2.915747	7.56E-06	T
ENSG0000(	MICAL3	0.8717	2.3991	2.571884	7.59E-06	T
ENSG0000(	DCAF13	2.327	6.8475	2.862588	7.71E-06	T
ENSG0000(	AC130324	0.1049	1.3645	7.147389	7.78E-06	T
ENSG0000(	NUP153	13.0229	26.6864	2.041195	7.78E-06	T
ENSG0000(	VPS4B	61.257	28.6815	0.469083	7.81E-06	N
ENSG0000(	CENPP	0.4179	1.371	2.840317	7.81E-06	T
ENSG0000(	CENPF	1.8514	10.2768	5.317618	7.83E-06	T
ENSG0000(	KIF1C	46.4121	20.3597	0.439879	7.86E-06	N
ENSG0000(	DYNC2LI1	1.3145	3.0536	2.22948	7.88E-06	T
ENSG0000(	ZNF107	1.185	3.4845	2.789494	7.9E-06	T
ENSG0000(	BMP7	6.8223	22.047	3.19937	7.92E-06	T
ENSG0000(	OXSR1	24.242	11.451	0.47453	7.96E-06	N
ENSG0000(	TMEM237	1.9083	4.9751	2.527063	8.03E-06	T
ENSG0000(	MUC21	724.1174	77.4315	0.107056	8.04E-06	N
ENSG0000(	MBNL1	14.62	39.4248	2.685109	8.04E-06	T
ENSG0000(	SP6	4.1533	1.3156	0.332824	8.07E-06	N
ENSG0000(	DLGAP5	1.1281	7.0301	5.805798	8.1E-06	T
ENSG0000(	DEK	13.1822	42.7226	3.224059	8.12E-06	T
ENSG0000(	RNF225	20.8727	3.3171	0.162931	8.14E-06	N
ENSG0000(	BX470102	2.0659	0.8916	0.457824	8.33E-06	N
ENSG0000(	ZNF415P1	0.2327	1.2724	4.125038	8.38E-06	T
ENSG0000(	STIM2	1.3479	4.5407	3.205125	8.43E-06	T
ENSG0000(	SLC25A12	3.0074	7.4047	2.415106	8.47E-06	T
ENSG0000(	MAB21L4	80.2097	17.8174	0.223104	8.5E-06	N
ENSG0000(	MASTL	3.2832	8.9071	2.662302	8.5E-06	T
ENSG0000(	SMIM5	4.9683	1.4219	0.300278	8.56E-06	N
ENSG0000(	XPNPEP3	1.8126	4.3978	2.351668	8.67E-06	T
ENSG0000(	C2orf69	3.6408	7.7614	2.101529	8.71E-06	T
ENSG0000(	TAF1A	0.7257	2.8245	3.541843	8.73E-06	T

ENSG0000(	ZBED2	17.8303	2.8956	0.167069	8.74E-06	N
ENSG0000(	CENPL	0.6083	2.078	3.074968	8.76E-06	T
ENSG0000(	AC104041	0.0515	3.9564	26.77492	8.78E-06	T
ENSG0000(	TPR	5.9836	13.0749	2.165642	8.85E-06	T
ENSG0000(	CARMIL1	3.3802	9.0951	2.642118	8.9E-06	T
ENSG0000(	POLR2H	2.9061	10.6118	3.563355	8.96E-06	T
ENSG0000(	KRIT1	0.8674	2.0295	2.201261	8.99E-06	T
ENSG0000(	TPX2	3.6385	24.6437	6.618617	9E-06	T
ENSG0000(	AC007879	0.0462	0.2295	2.253762	9.02E-06	T
ENSG0000(	ANKRD39	0.2865	0.9929	2.827684	9.11E-06	T
ENSG0000(	CRTC3-AS	0.0686	0.406	3.001186	9.12E-06	T
ENSG0000(	DUT	4.7637	11.2845	2.340708	9.12E-06	T
ENSG0000(	MAPK3	51.8775	15.3606	0.297448	9.15E-06	N
ENSG0000(	RAB11A	73.0089	35.6443	0.488919	9.25E-06	N
ENSG0000(	TOMM22	18.3006	36.9159	2.011668	9.33E-06	T
ENSG0000(	CTDSPL2	2.92	7.6787	2.575728	9.35E-06	T
ENSG0000(	MC1R	0.0666	0.3332	2.60024	9.36E-06	T
ENSG0000(	FANCL	3.0634	9.3845	2.998198	9.41E-06	T
ENSG0000(	B3GNT6	8.4924	2.2662	0.275383	9.48E-06	N
ENSG0000(	CEP57L1	0.6444	1.4987	2.147636	9.61E-06	T
ENSG0000(	CCT4	21.3946	50.3031	2.344919	9.62E-06	T
ENSG0000(	C9orf3	3.7812	7.8558	2.04983	9.68E-06	T
ENSG0000(	CHRNA6	0.053	0.4634	3.682353	9.74E-06	T
ENSG0000(	DNMT3B	0.1938	2.3609	8.376106	9.78E-06	T
ENSG0000(	CWH43	18.1724	3.0586	0.172862	9.78E-06	N
ENSG0000(	C4orf3	174.0632	38.2814	0.220376	9.8E-06	N
ENSG0000(	SEC61A2	0.5888	1.465	2.272067	9.81E-06	T
ENSG0000(	SENP5	5.5757	15.5104	2.750392	9.85E-06	T
ENSG0000(	PYCR2	1.8167	7.004	3.70637	9.88E-06	T
ENSG0000(	WASHC5	7.9534	17.2612	2.15576	9.9E-06	T
ENSG0000(	PSMD14	4.966	10.3354	2.059889	9.91E-06	T
ENSG0000(	PPFIBP2	5.2134	2.4685	0.4834	9.94E-06	N
ENSG0000(	RCCD1	0.8302	2.1349	2.402602	1E-05	T
ENSG0000(	AL391005.	0.5912	0.0259	0.182147	1E-05	N
ENSG0000(	TRMT2B	2.845	5.9723	2.061902	1E-05	T
ENSG0000(	NCBP2	4.3084	13.4525	3.074245	1.01E-05	T
ENSG0000(	CCT5	12.686	36.0638	2.82839	1.01E-05	T
ENSG0000(	TMEM209	2.5652	8.6822	3.295137	1.01E-05	T
ENSG0000(	CASP2	3.6987	7.7752	2.07313	1.01E-05	T
ENSG0000(	SPRED1	2.5058	8.0332	3.121191	1.01E-05	T
ENSG0000(	GTSE1	0.6546	4.1328	5.609329	1.02E-05	T
ENSG0000(	HIST2H2A	134.7802	286.0328	2.121385	1.02E-05	T
ENSG0000(	PMS1	1.4874	3.2984	2.140859	1.04E-05	T
ENSG0000(	DNAAF2	1.6176	4.7629	2.831218	1.04E-05	T
ENSG0000(	CCNE2	1.0572	4.6478	4.102834	1.05E-05	T
ENSG0000(	AGTRAP	4.6926	14.835	3.116263	1.06E-05	T
ENSG0000(	MORF4L1	13.9024	28.8602	2.068231	1.06E-05	T
ENSG0000(	KIFC1	1.7485	7.6472	4.191074	1.07E-05	T
ENSG0000(	EEF1E1	1.7254	4.1799	2.344637	1.08E-05	T
ENSG0000(	XPO1	9.0751	24.456	2.676374	1.08E-05	T
ENSG0000(	FOXI2	1.1767	0.0591	0.124618	1.1E-05	N
ENSG0000(	DOHH	1.0466	2.4089	2.188121	1.11E-05	T
ENSG0000(	DESI2	5.6063	14.0309	2.476368	1.11E-05	T
ENSG0000(	TBC1D32	0.7194	3.0996	3.904808	1.11E-05	T
ENSG0000(	ANP32E	7.7635	20.1728	2.578089	1.12E-05	T
ENSG0000(	CENPQ	2.5606	8.3889	3.190596	1.12E-05	T
ENSG0000(	MTIF2	4.8218	10.7766	2.209883	1.12E-05	T
ENSG0000(	PAK2	13.485	29.498	2.178727	1.12E-05	T

ENSG0000(	BTG1	12.1849	36.5953	2.987025	1.13E-05	T
ENSG0000(	DYRK3	0.4676	2.9132	5.308668	1.13E-05	T
ENSG0000(	RNF224	1.9124	0.3823	0.239664	1.15E-05	N
ENSG0000(	MMS22L	0.4577	1.7447	3.307692	1.15E-05	T
ENSG0000(	MKRN2OS	1.8471	0.4319	0.273175	1.15E-05	N
ENSG0000(	SDHAF3	2.3409	6.2415	2.598017	1.16E-05	T
ENSG0000(	RNF13	10.2209	30.3205	2.947466	1.16E-05	T
ENSG0000(	ECT2	2.1779	23.7999	10.49208	1.16E-05	T
ENSG0000(	CCT3	20.3865	53.428	2.612843	1.16E-05	T
ENSG0000(	NIF3L1	4.6403	11.2842	2.401578	1.17E-05	T
ENSG0000(	CKMT2-A	0.3748	0.9531	2.217987	1.18E-05	T
ENSG0000(	NXT2	4.0166	11.0624	2.711558	1.18E-05	T
ENSG0000(	SMPD4	3.2271	8.2391	2.506417	1.18E-05	T
ENSG0000(	C17orf75	0.7677	1.8618	2.26092	1.19E-05	T
ENSG0000(	GPR180	1.1048	2.5161	2.171398	1.19E-05	T
ENSG0000(	C16orf95	0.0824	0.3556	2.497807	1.19E-05	T
ENSG0000(	EFHC1	0.2352	0.7262	2.464797	1.2E-05	T
ENSG0000(	C6orf132	127.7179	29.1459	0.228809	1.2E-05	N
ENSG0000(	TBX3	0.6234	2.55	3.663257	1.21E-05	T
ENSG0000(	SPATA33	0.2469	0.7381	2.41597	1.21E-05	T
ENSG0000(	HSPD1	20.3177	68.4822	3.358958	1.21E-05	T
ENSG0000(	LINC0242	0	2.8614	29.614	1.22E-05	T
ENSG0000(	TTK	1.2094	6.996	5.419276	1.23E-05	T
ENSG0000(	GNG4	3.8527	0.4927	0.149948	1.23E-05	N
ENSG0000(	IQCH	0.1031	0.3364	2.148695	1.25E-05	T
ENSG0000(	ATAD5	0.8377	4.0344	4.409086	1.25E-05	T
ENSG0000(	SEC62	7.9506	17.9685	2.244367	1.26E-05	T
ENSG0000(	SUV39H2	1.992	5.4345	2.645554	1.29E-05	T
ENSG0000(	SSB	4.2549	11.3932	2.639142	1.29E-05	T
ENSG0000(	PRPS2	6.8563	18.7484	2.709544	1.3E-05	T
ENSG0000(	FLRT3	1.1213	4.785	3.999836	1.31E-05	T
ENSG0000(	MTRR	1.9484	5.244	2.608865	1.31E-05	T
ENSG0000(	DCTPP1	3.8979	13.7796	3.471723	1.33E-05	T
ENSG0000(	OSGEP	1.375	3.5127	2.449288	1.33E-05	T
ENSG0000(	AL022100.	0.0183	1.4094	12.75909	1.35E-05	T
ENSG0000(	VSIG10L	96.4206	25.9823	0.270225	1.35E-05	N
ENSG0000(	SNRPB2	5.8898	13.1536	2.212695	1.36E-05	T
ENSG0000(	NUP155	3.0358	10.2475	3.299796	1.36E-05	T
ENSG0000(	FANCI	1.354	7.7298	5.385007	1.37E-05	T
ENSG0000(	PDCD5	6.2082	13.28	2.121049	1.39E-05	T
ENSG0000(	NEK11	0.4597	1.441	2.753261	1.39E-05	T
ENSG0000(	POLR1A	2.1156	5.2821	2.429184	1.4E-05	T
ENSG0000(	GUSBP1	0.6272	1.56	2.282728	1.41E-05	T
ENSG0000(	AC079336	0.0739	0.2494	2.009201	1.41E-05	T
ENSG0000(	BUB1B	1.1239	5.7956	4.81706	1.41E-05	T
ENSG0000(	CHML	1.3951	7.4488	5.049027	1.41E-05	T
ENSG0000(	TCEA1	12.5932	26.1245	2.066027	1.44E-05	T
ENSG0000(	RF00019	0.239	1.6918	5.285546	1.44E-05	T
ENSG0000(	CSTF3	2.1341	5.018	2.290855	1.44E-05	T
ENSG0000(	ZNF697	0.4071	1.3449	2.849339	1.46E-05	T
ENSG0000(	NSMCE2	3.274	7.3469	2.207143	1.46E-05	T
ENSG0000(	CPSF3	4.8436	11.8545	2.418177	1.46E-05	T
ENSG0000(	RNF217	2.6721	7.1753	2.624472	1.46E-05	T
ENSG0000(	AC025171	0.5611	2.0497	3.251702	1.47E-05	T
ENSG0000(	TYRO3	5.3744	2.1883	0.418	1.47E-05	N
ENSG0000(	TTC26	0.3622	1.54	3.548248	1.48E-05	T
ENSG0000(	DDX18	3.9661	10.8923	2.703401	1.48E-05	T
ENSG0000(	GCKR	0.7309	0.1114	0.254423	1.49E-05	N

ENSG0000(	ATP6V1D	34.3711	15.0549	0.439641	1.49E-05	N
ENSG0000(	MRPS9	4.4426	9.2569	2.059812	1.5E-05	T
ENSG0000(	DDX27	3.064	8.8713	2.83543	1.5E-05	T
ENSG0000(	LMAN2L	2.5936	7.2156	2.715919	1.5E-05	T
ENSG0000(	LINC0046'	0.4241	1.3326	2.733448	1.51E-05	T
ENSG0000(	HMGN2P5	6.6189	17.2396	2.58072	1.52E-05	T
ENSG0000(	SPINK5	1368.186	159.726	0.116807	1.53E-05	N
ENSG0000(	WDR76	1.2871	5.4136	3.974912	1.55E-05	T
ENSG0000(	SELENOF	23.7323	51.981	2.185312	1.55E-05	T
ENSG0000(	MTF2	1.8409	4.8117	2.53063	1.56E-05	T
ENSG0000(	USP1	6.4712	16.7631	2.566213	1.57E-05	T
ENSG0000(	HSF2	1.5908	4.5859	2.77141	1.58E-05	T
ENSG0000(	LIPH	15.268	7.0901	0.467862	1.58E-05	N
ENSG0000(	CCNL1	5.9507	27.0424	4.485828	1.58E-05	T
ENSG0000(	DNA2	0.7626	3.3853	4.040459	1.59E-05	T
ENSG0000(	MPZL3	43.5234	14.9745	0.34556	1.59E-05	N
ENSG0000(	MAP2K5	1.843	3.8566	2.036336	1.6E-05	T
ENSG0000(	MND1	0.6939	2.2444	2.953017	1.6E-05	T
ENSG0000(	PTPN1	11.4171	27.5092	2.397235	1.61E-05	T
ENSG0000(	CAST	76.5359	32.5366	0.425866	1.61E-05	N
ENSG0000(	BX255925	22.9756	5.5359	0.244236	1.61E-05	N
ENSG0000(	CENPI	0.5216	3.9503	6.515927	1.62E-05	T
ENSG0000(	PDSS1	2.2908	5.3277	2.270244	1.63E-05	T
ENSG0000(	LEXM	14.9411	1.8994	0.132929	1.63E-05	N
ENSG0000(	SYMPK	3.2983	7.339	2.189036	1.63E-05	T
ENSG0000(	CYB561A3	1.0917	3.1968	2.766468	1.63E-05	T
ENSG0000(	TRAF3IP1	2.2101	5.2833	2.330332	1.64E-05	T
ENSG0000(	ARFGAP3	5.0792	11.4949	2.238743	1.64E-05	T
ENSG0000(	SNRPE	7.9638	23.6117	2.940512	1.64E-05	T
ENSG0000(	SPRTN	1.6749	3.6904	2.135557	1.66E-05	T
ENSG0000(	TOP2A	4.2586	28.2494	6.504244	1.66E-05	T
ENSG0000(	DPY19L2F	0.0676	0.3303	2.567422	1.68E-05	T
ENSG0000(	EWSR1	5.9441	15.0942	2.51389	1.69E-05	T
ENSG0000(	LRRC45	1.0196	2.5351	2.353608	1.69E-05	T
ENSG0000(	AL669831.	0.4512	1.0502	2.08672	1.69E-05	T
ENSG0000(	KIF3B	3.5136	10.206	2.852004	1.7E-05	T
ENSG0000(	TMEM182	0.2181	1.0943	3.75448	1.71E-05	T
ENSG0000(	AGER	0.5107	1.5164	2.646799	1.71E-05	T
ENSG0000(	B9D2	0.8123	2.4331	2.776609	1.72E-05	T
ENSG0000(	AMZ2	5.7782	12.4022	2.126876	1.73E-05	T
ENSG0000(	METTL13	2.6653	6.0973	2.241095	1.73E-05	T
ENSG0000(	CRNN	4701.273	489.2719	0.104091	1.73E-05	N
ENSG0000(	CENPA	0.7591	5.4784	6.493307	1.74E-05	T
ENSG0000(	CPNE8	1.4173	8.5608	5.708034	1.74E-05	T
ENSG0000(	NXT1	4.3918	12.945	2.904181	1.75E-05	T
ENSG0000(	PCNA	19.6281	83.3219	4.228583	1.75E-05	T
ENSG0000(	FETUB	6.3684	1.3928	0.230784	1.75E-05	N
ENSG0000(	CNN3	88.8781	36.2317	0.408322	1.75E-05	N
ENSG0000(	S100A10	466.5732	183.7839	0.394031	1.76E-05	N
ENSG0000(	DHFR	2.73	8.6791	3.102155	1.76E-05	T
ENSG0000(	IPO9	2.4237	6.9153	2.779768	1.76E-05	T
ENSG0000(	SLC25A36	2.3753	8.0434	3.289864	1.77E-05	T
ENSG0000(	CHP1	113.7867	51.2175	0.450601	1.77E-05	N
ENSG0000(	TAGLN2	66.585	171.6277	2.575207	1.78E-05	T
ENSG0000(	DLEU2	0.9802	3.089	2.952231	1.78E-05	T
ENSG0000(	HIST1H3C	9.1996	40.0192	4.314078	1.78E-05	T
ENSG0000(	CENPK	0.6553	2.6618	3.65656	1.79E-05	T
ENSG0000(	TPRG1	19.4713	5.7298	0.297875	1.79E-05	N

ENSG0000(	SPPL2A	5.1166	10.4334	2.019208	1.79E-05	T
ENSG0000(	LYRM2	1.9844	4.0768	2.003838	1.8E-05	T
ENSG0000(	WDR77	3.2913	7.8187	2.335004	1.81E-05	T
ENSG0000(	MCM4	5.6846	24.4002	4.235418	1.82E-05	T
ENSG0000(	METAP1D	0.4126	1.3376	2.804526	1.82E-05	T
ENSG0000(	FASTKD1	3.6294	7.4643	2.028289	1.82E-05	T
ENSG0000(	CCNB2	1.9052	9.7547	4.914572	1.83E-05	T
ENSG0000(	PUS7	2.3192	6.9108	2.897983	1.83E-05	T
ENSG0000(	HELLS	1.0369	5.2191	4.6786	1.83E-05	T
ENSG0000(	FAM72B	0.2365	0.9707	3.181872	1.84E-05	T
ENSG0000(	DVL3	10.909	28.1217	2.563512	1.84E-05	T
ENSG0000(	NSD2	1.9577	5.463	2.703504	1.84E-05	T
ENSG0000(	SLC25A14	0.8159	2.3613	2.687302	1.84E-05	T
ENSG0000(	TMEM39A	2.4251	8.1299	3.259237	1.84E-05	T
ENSG0000(	POLR1C	1.7269	4.1149	2.307132	1.85E-05	T
ENSG0000(	ZNF695	0.0483	0.4697	3.841537	1.86E-05	T
ENSG0000(	ORC2	2.518	5.3443	2.079565	1.86E-05	T
ENSG0000(	PSMA4	7.6956	17.4423	2.250282	1.87E-05	T
ENSG0000(	POGLUT1	1.2972	3.8674	2.839536	1.87E-05	T
ENSG0000(	CTIF	4.9985	2.4291	0.496048	1.89E-05	N
ENSG0000(	SNUPN	0.9413	2.2087	2.217132	1.9E-05	T
ENSG0000(	TYW1	2.4994	5.246	2.056628	1.9E-05	T
ENSG0000(	HSPA14	2.253	5.7	2.464938	1.91E-05	T
ENSG0000(	HPS3	1.5304	6.3098	3.931428	1.91E-05	T
ENSG0000(	SLC2A9	0.7404	2.6964	3.327463	1.92E-05	T
ENSG0000(	XPO5	3.3078	7.535	2.240448	1.92E-05	T
ENSG0000(	QTRT2	1.4944	5.8397	3.725351	1.93E-05	T
ENSG0000(	PPP1R14C	4.2592	17.2512	3.980363	1.93E-05	T
ENSG0000(	ZNF662	1.9987	0.3667	0.222376	1.94E-05	N
ENSG0000(	MIR6747	0.0518	1.1319	8.115283	1.94E-05	T
ENSG0000(	ACTL6A	4.9636	19.9926	3.968046	1.95E-05	T
ENSG0000(	NEK8	0.288	0.8677	2.494072	1.95E-05	T
ENSG0000(	TUBB6	64.845	21.56	0.333513	1.96E-05	N
ENSG0000(	LRRC58	4.8813	16.946	3.421998	1.96E-05	T
ENSG0000(	GINS1	1.439	7.4093	4.879337	1.96E-05	T
ENSG0000(	RF00019	2.2135	19.9842	8.681305	1.97E-05	T
ENSG0000(	ATAD2	3.3178	14.783	4.354556	1.98E-05	T
ENSG0000(	TMEM39B	1.8006	4.5116	2.426392	1.98E-05	T
ENSG0000(	NOCT	1.4868	5.1512	3.309302	1.99E-05	T
ENSG0000(	EXOSC5	3.1547	9.3647	2.90801	2E-05	T
ENSG0000(	TUBG2	1.7359	4.2933	2.392995	2E-05	T
ENSG0000(	FANCB	0.667	2.2879	3.113299	2E-05	T
ENSG0000(	ZNF146	7.2867	19.8033	2.694478	2.01E-05	T
ENSG0000(	AFDN	41.8816	16.5645	0.396948	2.04E-05	N
ENSG0000(	PTK2	4.684	12.71	2.677676	2.05E-05	T
ENSG0000(	AC010735	0.0509	0.8182	6.084824	2.06E-05	T
ENSG0000(	ACOT13	1.0075	3.2136	2.991964	2.07E-05	T
ENSG0000(	CPSF7	9.2865	19.3149	2.068385	2.07E-05	T
ENSG0000(	PRPF3	3.0274	6.8289	2.215546	2.08E-05	T
ENSG0000(	HTRA2	2.3812	5.4183	2.224045	2.08E-05	T
ENSG0000(	C1D	3.1998	7.1801	2.206225	2.11E-05	T
ENSG0000(	YWHAH	17.3998	41.6355	2.384913	2.11E-05	T
ENSG0000(	ANO8	6.1234	2.5725	0.429428	2.12E-05	N
ENSG0000(	C3orf67	5.0888	1.1853	0.247707	2.12E-05	N
ENSG0000(	SERPINB1	466.3749	141.2085	0.302928	2.13E-05	N
ENSG0000(	GNPDA1	2.4311	5.9816	2.40275	2.14E-05	T
ENSG0000(	ISG20L2	4.2052	9.6029	2.253763	2.15E-05	T
ENSG0000(	LINC0051	0.3495	2.4183	5.602447	2.17E-05	T

ENSG0000(	ALS2	1.2165	2.5582	2.019142	2.19E-05	T
ENSG0000(	CDC27	6.1936	12.8468	2.057137	2.2E-05	T
ENSG0000(	NDUFAF6	0.7545	2.4087	2.935869	2.2E-05	T
ENSG0000(	TIMM8A	0.9261	2.4172	2.453172	2.21E-05	T
ENSG0000(	LRCH3	2.2279	4.799	2.104472	2.21E-05	T
ENSG0000(	HNRNPH1	10.5644	28.6869	2.699345	2.25E-05	T
ENSG0000(	STN1	50.1401	12.2211	0.245244	2.25E-05	N
ENSG0000(	COX11	1.7914	4.1008	2.221	2.25E-05	T
ENSG0000(	WDR72	0.1512	11.339	45.53742	2.27E-05	T
ENSG0000(	AHI1	1.0367	2.7943	2.54623	2.27E-05	T
ENSG0000(	TMX1	8.9349	24.2042	2.690035	2.27E-05	T
ENSG0000(	ASF1A	5.842	13.7512	2.331067	2.28E-05	T
ENSG0000(	JPH1	0.6622	3.3372	4.509578	2.29E-05	T
ENSG0000(	CYP2J2	7.8104	1.5241	0.205312	2.29E-05	N
ENSG0000(	SH3TC2	0.9621	0.224	0.305056	2.3E-05	N
ENSG0000(	POLD1	1.1255	4.2897	3.581967	2.31E-05	T
ENSG0000(	NAV1	1.2649	4.6185	3.45703	2.31E-05	T
ENSG0000(	TSEN15	1.0538	3.25	2.903449	2.31E-05	T
ENSG0000(	EML6	0.1804	0.5593	2.351284	2.33E-05	T
ENSG0000(	ZC3HC1	1.2901	3.3395	2.474282	2.33E-05	T
ENSG0000(	NDC1	3.7765	12.1821	3.168348	2.36E-05	T
ENSG0000(	SUPT7L	3.2072	6.7902	2.083394	2.37E-05	T
ENSG0000(	TUBB	62.1099	184.0161	2.959595	2.37E-05	T
ENSG0000(	IFT27	0.4871	1.5626	2.831886	2.39E-05	T
ENSG0000(	AC012360	0.1644	0.5548	2.476551	2.4E-05	T
ENSG0000(	RSRC1	2.9275	10.8246	3.608456	2.4E-05	T
ENSG0000(	SLC35G1	0.2665	1.3006	3.821555	2.41E-05	T
ENSG0000(	DTL	0.8928	5.7477	5.890109	2.43E-05	T
ENSG0000(	NCBP2-AS	5.6588	19.8057	3.456571	2.45E-05	T
ENSG0000(	PTPN14	3.4733	7.5778	2.148658	2.46E-05	T
ENSG0000(	FBXL16	3.8548	1.3583	0.368742	2.46E-05	N
ENSG0000(	CWF19L1	1.9573	5.6198	2.780246	2.47E-05	T
ENSG0000(	AC097376	0.4535	1.0515	2.080397	2.47E-05	T
ENSG0000(	HOPX	156.2259	22.9027	0.147146	2.48E-05	N
ENSG0000(	UBL3	72.1366	18.26	0.254165	2.48E-05	N
ENSG0000(	LYPD2	423.8524	41.5978	0.098355	2.49E-05	N
ENSG0000(	AC012498	0.0826	0.906	5.50931	2.49E-05	T
ENSG0000(	ECHDC1	3.6468	10.0708	2.71453	2.5E-05	T
ENSG0000(	GTDC1	0.6778	1.5193	2.081898	2.5E-05	T
ENSG0000(	NOP58	6.8333	23.7461	3.439358	2.51E-05	T
ENSG0000(	RIF1	2.577	8.0354	3.038999	2.54E-05	T
ENSG0000(	CDCA3	0.4206	1.9231	3.886093	2.54E-05	T
ENSG0000(	AC004951	9.904	3.2913	0.338994	2.54E-05	N
ENSG0000(	AL035461.	0.1685	1.0642	4.33594	2.54E-05	T
ENSG0000(	ELK4	6.764	14.7634	2.165414	2.54E-05	T
ENSG0000(	LSG1	3.8533	16.1578	4.112463	2.55E-05	T
ENSG0000(	NFXL1	2.1432	4.8899	2.224456	2.55E-05	T
ENSG0000(	CCAR1	6.0705	13.3103	2.173292	2.56E-05	T
ENSG0000(	EML4	5.3163	17.2601	3.205159	2.57E-05	T
ENSG0000(	GAS5	11.2976	22.7292	2.002983	2.57E-05	T
ENSG0000(	KIF22	3.4232	11.1875	3.203764	2.58E-05	T
ENSG0000(	KPNA2	8.2439	37.2414	4.475293	2.58E-05	T
ENSG0000(	PSMB1	20.3928	44.9791	2.199753	2.59E-05	T
ENSG0000(	CAB39L	6.785	2.4332	0.36793	2.59E-05	N
ENSG0000(	TMEM154	35.3884	14.0059	0.397479	2.6E-05	N
ENSG0000(	HOXC10	0.0402	2.7222	20.12981	2.61E-05	T
ENSG0000(	PDIA6	24.2079	56.3591	2.322665	2.61E-05	T
ENSG0000(	HIST1H4F	15.4795	36.5383	2.351699	2.61E-05	T

ENSG0000(	BUB1	1.0754	5.7487	4.975923	2.63E-05	T
ENSG0000(	U2SURP	4.1594	14.7482	3.485984	2.63E-05	T
ENSG0000(	C1QTNF6	0.2853	0.8697	2.51674	2.65E-05	T
ENSG0000(	PLEKHA7	8.4569	3.8284	0.459091	2.65E-05	N
ENSG0000(	CNOT9	6.2905	13.5026	2.128566	2.67E-05	T
ENSG0000(	MED10	6.805	13.8785	2.024403	2.68E-05	T
ENSG0000(	RPRM	0.0287	0.607	5.493395	2.69E-05	T
ENSG0000(	CCDC9B	4.7634	1.295	0.286836	2.69E-05	N
ENSG0000(	STRN3	5.983	14.4752	2.396055	2.69E-05	T
ENSG0000(	FAM91A1	7.9833	19.0966	2.374847	2.7E-05	T
ENSG0000(	BRCA2	0.6675	2.9069	3.917785	2.7E-05	T
ENSG0000(	SRM	9.3549	21.6015	2.295265	2.71E-05	T
ENSG0000(	AL031577.	0.141	1.0113	4.611203	2.72E-05	T
ENSG0000(	CLCN2	0.2169	0.9704	3.377722	2.73E-05	T
ENSG0000(	FIGNL1	1.6147	5.2706	3.132093	2.74E-05	T
ENSG0000(	KIF3A	0.8025	2.0824	2.418172	2.75E-05	T
ENSG0000(	ANAPC5	4.1464	8.6607	2.063089	2.75E-05	T
ENSG0000(	SCNN1B	36.0483	11.8278	0.329968	2.76E-05	N
ENSG0000(	MAD2L1	0.8279	3.5495	3.933075	2.77E-05	T
ENSG0000(	ILF3	7.2068	17.749	2.442793	2.77E-05	T
ENSG0000(	SNORA49	19.0776	54.2698	2.835068	2.77E-05	T
ENSG0000(	TMEM161	1.3387	3.5031	2.504414	2.78E-05	T
ENSG0000(	USP39	5.8678	13.3201	2.248752	2.78E-05	T
ENSG0000(	AC138696	0.6937	2.5957	3.396371	2.79E-05	T
ENSG0000(	SP140L	1.1999	4.9995	3.922994	2.79E-05	T
ENSG0000(	LRPPRC	8.1536	24.2058	2.944873	2.79E-05	T
ENSG0000(	FAM98B	2.5411	6.6043	2.53845	2.82E-05	T
ENSG0000(	AC027279	0.256	0.966	2.994382	2.82E-05	T
ENSG0000(	AC104791	0.0436	0.2996	2.78273	2.83E-05	T
ENSG0000(	CASK	1.7035	4.8693	2.755365	2.83E-05	T
ENSG0000(	ABLIM1	166.0224	57.9349	0.34935	2.84E-05	N
ENSG0000(	FAM3B	10.1646	4.716	0.469185	2.85E-05	N
ENSG0000(	SPRR3	14262.95	2073.615	0.145391	2.85E-05	N
ENSG0000(	COG2	0.9826	2.5845	2.479679	2.85E-05	T
ENSG0000(	KNSTRN	1.0113	4.2156	3.88338	2.86E-05	T
ENSG0000(	AC113404	0.9721	3.5422	3.397258	2.87E-05	T
ENSG0000(	PRIM1	1.098	3.5286	3.028881	2.88E-05	T
ENSG0000(	DTNBP1	1.0479	2.7178	2.454743	2.88E-05	T
ENSG0000(	ABLIM2	4.492	1.8124	0.416463	2.88E-05	N
ENSG0000(	NUCB2	26.321	8.4546	0.32378	2.9E-05	N
ENSG0000(	S100A16	635.7381	252.0335	0.396537	2.91E-05	N
ENSG0000(	DONSON	1.5524	3.8767	2.406621	2.91E-05	T
ENSG0000(	SH3GL1	67.4483	23.012	0.342155	2.91E-05	N
ENSG0000(	RANGAP1	9.8551	19.9175	2.010778	2.92E-05	T
ENSG0000(	RBP7	19.2893	3.5829	0.189945	2.93E-05	N
ENSG0000(	PSMC2	12.6542	27.7014	2.179784	2.94E-05	T
ENSG0000(	FBXO22	0.9753	2.559	2.472798	2.94E-05	T
ENSG0000(	SRSF7	10.0551	27.5237	2.72018	2.95E-05	T
ENSG0000(	PRC1	0.0728	0.332	2.5	2.96E-05	T
ENSG0000(	FBXO5	1.0348	3.9549	3.573229	2.96E-05	T
ENSG0000(	BIRC5	1.5537	9.3486	5.713612	2.97E-05	T
ENSG0000(	NEU1	3.0989	7.8509	2.485511	2.97E-05	T
ENSG0000(	E2F3	1.6077	5.3153	3.171107	2.97E-05	T
ENSG0000(	ATAT1	1.1032	2.8832	2.479388	2.97E-05	T
ENSG0000(	HMGB2	9.028	23.578	2.593996	2.98E-05	T
ENSG0000(	CTNNBL1	3.9033	8.2521	2.086304	2.99E-05	T
ENSG0000(	SNAI2	3.3467	14.5013	4.236313	2.99E-05	T
ENSG0000(	BX322234	0.2606	1.1027	3.335275	3E-05	T

ENSG0000(	MCM8	0.7097	4.3498	5.495616	3.01E-05	T
ENSG0000(	NDUFAF5	0.7425	1.7619	2.20997	3.01E-05	T
ENSG0000(	TUBE1	0.7919	1.9511	2.299697	3.03E-05	T
ENSG0000(	CEP97	0.7588	2.882	3.472287	3.04E-05	T
ENSG0000(	MTBP	0.2538	1.6903	5.060204	3.04E-05	T
ENSG0000(	HSPA13	3.4801	8.7774	2.479651	3.06E-05	T
ENSG0000(	CHD1L	4.0464	8.6081	2.100159	3.07E-05	T
ENSG0000(	LINC0224!	0.0056	0.3379	4.14678	3.08E-05	T
ENSG0000(	UBA5	2.6517	6.7434	2.486972	3.09E-05	T
ENSG0000(	ATF2	4.3875	10.3994	2.339699	3.1E-05	T
ENSG0000(	TRMT6	2.3585	8.4209	3.465894	3.1E-05	T
ENSG0000(	FTSJ3	2.9696	6.592	2.180089	3.11E-05	T
ENSG0000(	TGS1	3.7612	11.9626	3.124055	3.11E-05	T
ENSG0000(	CLDN12	2.654	6.0503	2.233224	3.11E-05	T
ENSG0000(	FUS	4.9408	17.0358	3.399421	3.12E-05	T
ENSG0000(	SKP2	4.5019	13.56	2.968339	3.13E-05	T
ENSG0000(	NOMO1	5.1098	13.6396	2.637261	3.13E-05	T
ENSG0000(	KRT13	14436.83	3935.136	0.272581	3.14E-05	N
ENSG0000(	BAALC-A	0.0939	0.4622	2.899433	3.15E-05	T
ENSG0000(	RRP15	1.14	2.9233	2.438145	3.15E-05	T
ENSG0000(	IL1RAP	4.464	11.9959	2.650285	3.16E-05	T
ENSG0000(	RNF168	6.131	25.1627	4.054357	3.17E-05	T
ENSG0000(	RBM12	6.765	14.7169	2.158325	3.17E-05	T
ENSG0000(	DOCK3	1.5579	0.2611	0.217806	3.17E-05	N
ENSG0000(	STAG1	5.0451	14.2464	2.788362	3.18E-05	T
ENSG0000(	LSM8	1.6255	3.9882	2.369284	3.19E-05	T
ENSG0000(	AC021078	2.7058	7.049	2.547936	3.19E-05	T
ENSG0000(	LIG1	1.4491	5.2621	3.461429	3.19E-05	T
ENSG0000(	TRMT12	1.5233	4.5076	2.838416	3.21E-05	T
ENSG0000(	POC5	0.6384	2.0545	2.917795	3.21E-05	T
ENSG0000(	WSB1	4.1307	9.7985	2.339684	3.22E-05	T
ENSG0000(	ZRANB2	7.1127	15.1959	2.12069	3.23E-05	T
ENSG0000(	ASTE1	0.9447	3.4318	3.380683	3.25E-05	T
ENSG0000(	CEP41	0.2482	0.8572	2.748995	3.26E-05	T
ENSG0000(	CCDC162I	0.031	1.1668	9.670229	3.27E-05	T
ENSG0000(	SNHG19	9.7337	26.923	2.747999	3.27E-05	T
ENSG0000(	CLN6	0.8932	2.5761	2.694422	3.28E-05	T
ENSG0000(	TTI1	2.6106	7.9989	2.987862	3.28E-05	T
ENSG0000(	C18orf54	0.3426	1.8275	4.354948	3.29E-05	T
ENSG0000(	ZWILCH	1.8579	6.3519	3.295316	3.3E-05	T
ENSG0000(	POLR1B	2.8495	7.4262	2.551687	3.32E-05	T
ENSG0000(	ZNF844	2.4403	0.4592	0.220131	3.32E-05	N
ENSG0000(	ANO7L1	15.0948	3.872	0.261405	3.32E-05	N
ENSG0000(	RFC3	1.6833	6.536	3.721191	3.35E-05	T
ENSG0000(	ADSS	8.6386	22.0756	2.53766	3.38E-05	T
ENSG0000(	LINC0113'	1.4437	3.8188	2.538576	3.39E-05	T
ENSG0000(	ICA1	1.9841	0.7535	0.409529	3.4E-05	N
ENSG0000(	LINC0063(	0.8712	2.0634	2.227554	3.41E-05	T
ENSG0000(	BSPRY	14.9974	4.7182	0.319141	3.43E-05	N
ENSG0000(	EHD3	48.1265	9.3051	0.195019	3.43E-05	N
ENSG0000(	NAXE	11.4663	25.9873	2.255458	3.44E-05	T
ENSG0000(	ACTR3B	0.4166	1.0793	2.282811	3.44E-05	T
ENSG0000(	SLC26A11	0.6902	1.5202	2.050367	3.46E-05	T
ENSG0000(	ASAP1	3.2311	11.3593	3.440095	3.47E-05	T
ENSG0000(	AL512274.	54.8827	9.2657	0.170339	3.47E-05	N
ENSG0000(	AC026304	0.3329	1.026	2.601063	3.47E-05	T
ENSG0000(	LINC0254!	1.0623	2.6537	2.369182	3.48E-05	T
ENSG0000(	FAM111B	1.4119	8.3739	5.604802	3.49E-05	T

ENSG0000(	SHISAL2E	0.4603	1.9834	3.718365	3.49E-05	T
ENSG0000(	COL4A5	0.6631	3.1565	4.267462	3.52E-05	T
ENSG0000(	ZNF451	2.6833	5.7492	2.101534	3.52E-05	T
ENSG0000(	NR2C2AP	2.5004	5.9624	2.331334	3.52E-05	T
ENSG0000(	GGT7	1.2473	4.7733	3.617086	3.52E-05	T
ENSG0000(	SRRT	7.287	16.4969	2.246771	3.52E-05	T
ENSG0000(	UBE2C	4.6025	30.1031	6.422775	3.54E-05	T
ENSG0000(	SSR3	19.3121	45.0546	2.326106	3.54E-05	T
ENSG0000(	MIR4451	0.4302	3.6221	7.020181	3.57E-05	T
ENSG0000(	NPHP4	0.3674	1.0579	2.477321	3.58E-05	T
ENSG0000(	KAT2A	2.655	7.6644	2.818294	3.59E-05	T
ENSG0000(	TARBP2	1.2974	3.0788	2.274796	3.59E-05	T
ENSG0000(	LRWD1	0.706	2.0597	2.679529	3.62E-05	T
ENSG0000(	MAD2L2	0.9368	3.9236	3.880787	3.65E-05	T
ENSG0000(	NEDD4	1.448	4.121	2.726744	3.65E-05	T
ENSG0000(	PHC3	4.1073	9.4283	2.264707	3.65E-05	T
ENSG0000(	HSPBAP1	0.76	2.6916	3.246047	3.68E-05	T
ENSG0000(	AGK	1.0808	2.9278	2.564194	3.7E-05	T
ENSG0000(	CSE1L	16.0889	47.4765	2.938835	3.7E-05	T
ENSG0000(	SH3GLB2	13.5356	6.623	0.493048	3.7E-05	N
ENSG0000(	WDR74	1.9541	6.4962	3.211236	3.72E-05	T
ENSG0000(	TSNAX	1.9841	4.8784	2.388753	3.72E-05	T
ENSG0000(	RRAGD	13.2702	4.8612	0.371064	3.73E-05	N
ENSG0000(	AL136984.	0.1077	0.5087	2.930669	3.75E-05	T
ENSG0000(	ITGB1BP1	1.7518	4.2209	2.333351	3.77E-05	T
ENSG0000(	PCLAF	0.4391	2.5503	4.916157	3.79E-05	T
ENSG0000(	ZFR	8.7986	17.7347	2.004214	3.81E-05	T
ENSG0000(	MCM3	6.2911	22.5392	3.542301	3.82E-05	T
ENSG0000(	CIT	0.8847	3.5736	3.730679	3.84E-05	T
ENSG0000(	DPY19L4	2.7268	8.7867	3.143731	3.84E-05	T
ENSG0000(	SH3BGRL	164.604	17.6342	0.107673	3.84E-05	N
ENSG0000(	PRPS1	2.7856	9.5373	3.339791	3.84E-05	T
ENSG0000(	ERCC3	2.0703	4.6428	2.18532	3.85E-05	T
ENSG0000(	ALDH3B1	4.2448	0.7681	0.199802	3.85E-05	N
ENSG0000(	ASPH	5.0606	18.49	3.602294	3.86E-05	T
ENSG0000(	ZBTB5	13.2649	6.3659	0.483797	3.89E-05	N
ENSG0000(	RMDN2	0.3302	0.9974	2.550907	3.9E-05	T
ENSG0000(	BRMS1	3.3313	9.9773	2.936875	3.91E-05	T
ENSG0000(	MFAP2	0.1959	2.5336	8.900304	3.91E-05	T
ENSG0000(	IGSF9	1.3936	4.5659	3.123929	3.92E-05	T
ENSG0000(	TEAD2	1.6991	7.3226	4.12573	3.92E-05	T
ENSG0000(	MAP4K3	4.3477	10.2021	2.316276	3.92E-05	T
ENSG0000(	UST	1.3585	5.6349	3.932053	3.93E-05	T
ENSG0000(	RAD54B	0.2518	0.8831	2.794486	3.94E-05	T
ENSG0000(	MGME1	4.4423	15.4845	3.430971	3.95E-05	T
ENSG0000(	GEMIN7	2.2755	5.0906	2.185056	3.97E-05	T
ENSG0000(	TOP1MT	1.3541	3.8754	2.733925	3.98E-05	T
ENSG0000(	RHCG	1911.414	508.6393	0.266145	3.98E-05	N
ENSG0000(	RNF115	1.3801	3.0845	2.151544	3.99E-05	T
ENSG0000(	ULK3	26.6366	11.5653	0.436305	3.99E-05	N
ENSG0000(	HAUS8	0.2858	1.0662	3.02281	4E-05	T
ENSG0000(	AC103563	6.7699	0.5426	0.093538	4.01E-05	N
ENSG0000(	COA1	1.3249	3.0906	2.239175	4.02E-05	T
ENSG0000(	CENPE	0.9538	4.5627	4.424654	4.03E-05	T
ENSG0000(	TMEM260	1.268	3.271	2.464181	4.05E-05	T
ENSG0000(	SCNN1A	45.6402	22.0068	0.483312	4.07E-05	N
ENSG0000(	ADAMTSI	11.7853	4.2696	0.367647	4.09E-05	N
ENSG0000(	AC073585	0.1107	0.5659	3.160418	4.1E-05	T

ENSG0000(	ERCC8	0.9709	2.124	2.076758	4.12E-05	T
ENSG0000(	FLJ42393	0.2406	1.2853	4.067234	4.12E-05	T
ENSG0000(	RAD18	1.0454	2.4032	2.185437	4.13E-05	T
ENSG0000(	AL392172.	1.0048	3.0101	2.81508	4.14E-05	T
ENSG0000(	NPHP1	0.0844	0.2977	2.156725	4.14E-05	T
ENSG0000(	TMA16	2.3896	5.1761	2.119256	4.15E-05	T
ENSG0000(	HEATR6	1.261	3.2093	2.431521	4.17E-05	T
ENSG0000(	TMEM198	1.3488	3.3739	2.397777	4.18E-05	T
ENSG0000(	UBIAD1	1.4912	3.12	2.02363	4.19E-05	T
ENSG0000(	PGAP1	2.1804	6.3687	2.836651	4.2E-05	T
ENSG0000(	AC110619	1.7789	0.1939	0.156421	4.2E-05	N
ENSG0000(	SNORA14.	106.195	273.6042	2.574949	4.22E-05	T
ENSG0000(	MIS18A	1.956	5.7722	2.856128	4.22E-05	T
ENSG0000(	RBM28	1.0336	2.9887	2.724682	4.23E-05	T
ENSG0000(	WDR26	43.5585	16.5626	0.381658	4.25E-05	N
ENSG0000(	TXNRD2	0.5544	1.4284	2.335575	4.25E-05	T
ENSG0000(	PLBD1-AS	14.4554	5.3226	0.372549	4.27E-05	N
ENSG0000(	BRMS1L	1.4784	3.9997	2.597377	4.28E-05	T
ENSG0000(	MYBL1	0.1972	0.8851	3.314603	4.3E-05	T
ENSG0000(	CDC45	0.5591	4.4697	6.933242	4.32E-05	T
ENSG0000(	EGFL6	1.3346	4.1954	2.994145	4.32E-05	T
ENSG0000(	GALNT12	12.6233	4.7701	0.38277	4.32E-05	N
ENSG0000(	GTF2E1	2.241	5.7317	2.491115	4.36E-05	T
ENSG0000(	SNORA33	15.4943	48.8601	3.139615	4.38E-05	T
ENSG0000(	NELFCD	4.1737	11.172	2.637527	4.39E-05	T
ENSG0000(	POU5F2	0.1909	0.6307	2.51186	4.41E-05	T
ENSG0000(	GSS	3.906	8.7078	2.198652	4.42E-05	T
ENSG0000(	FLYWCH1	1.4246	4.6419	3.110258	4.45E-05	T
ENSG0000(	AC009404	0.0991	0.4909	2.967855	4.46E-05	T
ENSG0000(	HIST1H2E	28.0863	103.8003	3.686199	4.46E-05	T
ENSG0000(	RBM14	5.4192	13.3572	2.438252	4.47E-05	T
ENSG0000(	MCM6	3.3046	12.5872	3.726488	4.47E-05	T
ENSG0000(	FLVCR1	0.9028	3.2962	3.386717	4.48E-05	T
ENSG0000(	DHX16	4.0623	8.7095	2.116498	4.48E-05	T
ENSG0000(	AC092611	0.0649	0.5907	4.188599	4.48E-05	T
ENSG0000(	ARHGAP3	0.3192	1.0757	2.804628	4.5E-05	T
ENSG0000(	RPF2	3.3505	7.4543	2.189335	4.52E-05	T
ENSG0000(	GINS3	1.2728	3.0857	2.320586	4.55E-05	T
ENSG0000(	HNRNPU	8.3703	22.1501	2.626837	4.55E-05	T
ENSG0000(	SYNPR-AS	0	0.6052	7.052	4.56E-05	T
ENSG0000(	MAIP1	3.5176	8.1184	2.271782	4.57E-05	T
ENSG0000(	NOL7	7.5609	16.2611	2.135663	4.59E-05	T
ENSG0000(	FASTKD3	1.1457	3.0179	2.50293	4.6E-05	T
ENSG0000(	MCC	5.2949	11.1618	2.08749	4.6E-05	T
ENSG0000(	CHST11	1.079	4.3488	3.773367	4.62E-05	T
ENSG0000(	XRCC4	1.5077	3.8429	2.45251	4.63E-05	T
ENSG0000(	NUDCD1	1.8829	7.0665	3.614151	4.64E-05	T
ENSG0000(	UBA2	12.7795	29.5075	2.298808	4.65E-05	T
ENSG0000(	CIP2A	0.7271	5.2995	6.528231	4.66E-05	T
ENSG0000(	WDHD1	0.9349	4.4852	4.430573	4.67E-05	T
ENSG0000(	IWS1	4.6291	9.5129	2.032712	4.7E-05	T
ENSG0000(	TAF5	0.9194	2.4177	2.469786	4.73E-05	T
ENSG0000(	AL358334.	0.0128	0.2619	3.208333	4.76E-05	T
ENSG0000(	MTDH	14.3085	34.3603	2.391665	4.77E-05	T
ENSG0000(	BYSL	2.3146	6.6069	2.777644	4.77E-05	T
ENSG0000(	NOC3L	2.7931	6.5253	2.290035	4.78E-05	T
ENSG0000(	FAM49B	4.2939	13.2315	3.034093	4.79E-05	T
ENSG0000(	MMP11	0.0899	0.6256	3.820958	4.82E-05	T

ENSG0000(	COX7A2L	5.4372	13.2478	2.410569	4.82E-05	T
ENSG0000(	SLC47A1	0.3919	1.5717	3.398455	4.83E-05	T
ENSG0000(	RNF141	34.1472	16.2109	0.47627	4.83E-05	N
ENSG0000(	INTS8	3.0692	7.4147	2.371166	4.85E-05	T
ENSG0000(	AC104046	2.6777	11.2146	4.07337	4.87E-05	T
ENSG0000(	IQGAP3	1.1193	5.9569	4.967522	4.88E-05	T
ENSG0000(	LMF2	7.5458	19.165	2.519684	4.88E-05	T
ENSG0000(	WDYHV1	1.0366	2.4555	2.248372	4.88E-05	T
ENSG0000(	SMC3	7.4297	15.7966	2.111186	4.88E-05	T
ENSG0000(	PRRC2C	14.3423	29.1252	2.023584	4.89E-05	T
ENSG0000(	RBM45	0.7866	1.7638	2.102188	4.9E-05	T
ENSG0000(	AC079160	0.0168	0.9115	8.660103	4.94E-05	T
ENSG0000(	NEK2	0.7014	4.3847	5.596082	4.95E-05	T
ENSG0000(	MRPL55	2.867	7.9291	2.706134	4.95E-05	T
ENSG0000(	HEATR1	3.0547	11.9957	3.834184	4.98E-05	T
ENSG0000(	H1FX-AS1	0.3372	0.9252	2.344922	4.99E-05	T
ENSG0000(	DDOST	21.5977	44.7077	2.06509	4.99E-05	T
ENSG0000(	DPM1	19.3978	39.8193	2.047375	5E-05	T
ENSG0000(	RN7SKP29	0.6873	3.6609	4.776959	5E-05	T
ENSG0000(	RANGRF	1.2576	3.5695	2.702932	5.02E-05	T
ENSG0000(	AC022031	0	2.7453	28.453	5.03E-05	T
ENSG0000(	CEP162	1.3241	2.9807	2.163261	5.07E-05	T
ENSG0000(	HNRNPR	6.4156	15.6338	2.414789	5.07E-05	T
ENSG0000(	LTV1	3.3558	7.8695	2.306123	5.08E-05	T
ENSG0000(	RRAGB	1.4924	3.411	2.204848	5.08E-05	T
ENSG0000(	MTERF3	2.2095	8.2093	3.597878	5.11E-05	T
ENSG0000(	MR1	2.4601	5.1449	2.048709	5.12E-05	T
ENSG0000(	IFT57	3.0163	8.1556	2.649167	5.13E-05	T
ENSG0000(	UBE2T	1.1687	6.8517	5.479388	5.14E-05	T
ENSG0000(	SPAG5	0.7721	4.3543	5.107556	5.16E-05	T
ENSG0000(	HOXC11	0.0023	0.4643	5.516129	5.16E-05	T
ENSG0000(	GEN1	0.9575	4.18	4.047281	5.18E-05	T
ENSG0000(	KHDC4	3.1154	7.3899	2.329384	5.19E-05	T
ENSG0000(	TTC13	1.7349	5.0141	2.787127	5.2E-05	T
ENSG0000(	SLC25A32	0.499	1.9447	3.413523	5.23E-05	T
ENSG0000(	PLPP2	1.8525	7.9245	4.109859	5.23E-05	T
ENSG0000(	RBMX	9.6815	25.9427	2.662444	5.23E-05	T
ENSG0000(	PSMA7	31.775	72.7873	2.28666	5.25E-05	T
ENSG0000(	TMEM14A	12.9885	42.8409	3.280811	5.26E-05	T
ENSG0000(	AP2M1	26.3073	62.5958	2.374184	5.26E-05	T
ENSG0000(	AC106053	0.2464	1.4205	4.389434	5.27E-05	T
ENSG0000(	RAB30-AS	1.3887	4.5531	3.125613	5.28E-05	T
ENSG0000(	CASD1	1.5537	4.1951	2.597267	5.3E-05	T
ENSG0000(	CLSPN	0.5217	2.7316	4.554608	5.32E-05	T
ENSG0000(	PSMB4	19.2543	48.5224	2.512227	5.33E-05	T
ENSG0000(	EEF1DP2	0.6813	1.5359	2.093818	5.33E-05	T
ENSG0000(	WDR43	9.1837	26.8606	2.904079	5.34E-05	T
ENSG0000(	ZNF433	1.3821	0.4696	0.38432	5.34E-05	N
ENSG0000(	ING5	0.9114	2.1907	2.26488	5.36E-05	T
ENSG0000(	TSC22D4	19.3969	8.5538	0.443855	5.36E-05	N
ENSG0000(	EXO1	0.6273	5.1073	7.159769	5.39E-05	T
ENSG0000(	RHOT1P2	43.5457	6.7687	0.157374	5.39E-05	N
ENSG0000(	ELP1	3.0653	6.7428	2.161817	5.4E-05	T
ENSG0000(	KIF11	3.09	13.1388	4.150094	5.4E-05	T
ENSG0000(	MIEF1	2.7624	5.948	2.112912	5.41E-05	T
ENSG0000(	GAB2	9.4636	3.6626	0.393429	5.41E-05	N
ENSG0000(	AC015813	0.3111	1.5725	4.068353	5.41E-05	T
ENSG0000(	LRIF1	3.0851	6.8292	2.175505	5.42E-05	T

ENSG0000(	HAT1	2.7376	8.7838	3.130744	5.42E-05	T
ENSG0000(	CHEK1	0.7957	3.4006	3.908228	5.43E-05	T
ENSG0000(	AGPS	3.7452	8.3494	2.197389	5.45E-05	T
ENSG0000(	SNX24	14.5858	5.0598	0.351346	5.47E-05	N
ENSG0000(	RPL5P17	0.3508	1.0291	2.504658	5.47E-05	T
ENSG0000(	GJA3	0.4642	1.5711	2.961893	5.48E-05	T
ENSG0000(	CDC20	4.0977	19.9345	4.772733	5.48E-05	T
ENSG0000(	GMPSP1	0.5535	2.3344	3.725172	5.49E-05	T
ENSG0000(	HIST1H2E	42.5363	108.2485	2.541227	5.49E-05	T
ENSG0000(	GPR85	0.0369	0.1826	2.06428	5.49E-05	T
ENSG0000(	NPM1P6	0.4624	1.3786	2.62909	5.5E-05	T
ENSG0000(	ERICH6-A	0.2556	1.0758	3.306524	5.5E-05	T
ENSG0000(	TTC27	3.2883	7.5412	2.255172	5.51E-05	T
ENSG0000(	NCLN	4.476	11.9045	2.623361	5.51E-05	T
ENSG0000(	GNPAT	6.986	14.0723	2.000042	5.52E-05	T
ENSG0000(	FAM208B	4.3351	12.4824	2.837005	5.52E-05	T
ENSG0000(	MAPT	0.732	0.1129	0.255889	5.55E-05	N
ENSG0000(	PRR11	1.5026	6.6268	4.197429	5.57E-05	T
ENSG0000(	KATNB1	2.2143	5.0868	2.241196	5.6E-05	T
ENSG0000(	BRCA1	0.8104	3.6461	4.114785	5.61E-05	T
ENSG0000(	CAMSAP3	15.0121	6.2802	0.422191	5.61E-05	N
ENSG0000(	YDJC	2.8248	9.476	3.27407	5.61E-05	T
ENSG0000(	KIF23	1.1131	6.5145	5.45256	5.61E-05	T
ENSG0000(	DSN1	2.255	7.1201	3.06586	5.62E-05	T
ENSG0000(	AC122718	1.3054	8.6593	6.232603	5.63E-05	T
ENSG0000(	THOC2	3.6893	10.7495	2.863194	5.64E-05	T
ENSG0000(	KBTBD6	0.8597	2.1404	2.33448	5.67E-05	T
ENSG0000(	ETHE1	40.9311	14.8623	0.364658	5.67E-05	N
ENSG0000(	PRIM2	1.0703	4.4211	3.863197	5.68E-05	T
ENSG0000(	MRPL11	6.5696	20.7529	3.126559	5.68E-05	T
ENSG0000(	NCAPG	1.3455	6.9321	4.864822	5.68E-05	T
ENSG0000(	NOL11	4.9084	12.0933	2.43457	5.75E-05	T
ENSG0000(	PTK6	61.6621	17.3881	0.283153	5.78E-05	N
ENSG0000(	MRGBP	2.1646	6.8984	3.090347	5.79E-05	T
ENSG0000(	SPTSSA	8.0294	19.0241	2.352461	5.8E-05	T
ENSG0000(	MOGS	4.756	12.5972	2.614745	5.81E-05	T
ENSG0000(	TRIB2	2.2572	10.7499	4.602876	5.82E-05	T
ENSG0000(	AL033530.	0.0046	1.6529	16.75813	5.82E-05	T
ENSG0000(	MCRIP2	0.7458	2.6629	3.266611	5.83E-05	T
ENSG0000(	METTL5	3.3063	8.0068	2.379943	5.83E-05	T
ENSG0000(	SFTA2	20.4976	2.8393	0.142701	5.83E-05	N
ENSG0000(	TROAP	0.8427	5.2133	5.636258	5.85E-05	T
ENSG0000(	TFAP2A	5.9328	13.3344	2.226893	5.85E-05	T
ENSG0000(	CENPH	1.6721	6.6062	3.784324	5.87E-05	T
ENSG0000(	MCM5	2.2522	10.9659	4.704489	5.88E-05	T
ENSG0000(	HIGD1A	57.7846	28.5266	0.494546	5.88E-05	N
ENSG0000(	PRR15L	3.5891	0.8229	0.250169	5.89E-05	N
ENSG0000(	SLC30A6	4.9046	10.5979	2.137613	5.89E-05	T
ENSG0000(	EIF2A	17.2822	39.895	2.300917	5.89E-05	T
ENSG0000(	IFT122	0.6737	2.1864	2.955151	5.92E-05	T
ENSG0000(	SNRPGP1!	1.2134	3.7987	2.968403	5.98E-05	T
ENSG0000(	MIA3	3.3622	7.8178	2.286927	5.98E-05	T
ENSG0000(	EMC1	2.8135	7.0904	2.467959	6E-05	T
ENSG0000(	PNPT1	2.6186	10.5624	3.922019	6.01E-05	T
ENSG0000(	LRRC39	0.0731	0.4972	3.450029	6.01E-05	T
ENSG0000(	SRPK2	5.334	11.4267	2.121218	6.02E-05	T
ENSG0000(	AC010680	0.6971	1.9789	2.608079	6.02E-05	T
ENSG0000(	CCNA2	3.4182	12.9467	3.708345	6.02E-05	T

ENSG0000(	PHKG2	0.5318	1.5708	2.644508	6.03E-05	T
ENSG0000(	GTF2I	5.2829	11.002	2.062457	6.06E-05	T
ENSG0000(	TMPO-AS	0.3341	1.4637	3.602165	6.07E-05	T
ENSG0000(	UNC13D	7.2	3.4088	0.480658	6.07E-05	N
ENSG0000(	AC083855	0.4662	1.3563	2.572059	6.08E-05	T
ENSG0000(	NUDT11	0.3749	1.6402	3.66435	6.09E-05	T
ENSG0000(	HIST1H3B	110.3609	355.9815	3.223598	6.11E-05	T
ENSG0000(	BRD9	1.3467	4.1627	2.946499	6.12E-05	T
ENSG0000(	COL27A1	0.6572	2.4079	3.312071	6.12E-05	T
ENSG0000(	ACBD6	1.2852	4.1218	3.047791	6.13E-05	T
ENSG0000(	HIST1H3J	25.0964	96.1796	3.821165	6.13E-05	T
ENSG0000(	TRMT2A	2.6657	6.1926	2.275229	6.15E-05	T
ENSG0000(	HSPB8	92.9794	25.5935	0.276039	6.15E-05	N
ENSG0000(	C1orf174	2.2061	4.7312	2.094966	6.19E-05	T
ENSG0000(	SNRNP40	3.4044	9.1659	2.644076	6.19E-05	T
ENSG0000(	CDC25C	0.185	1.3082	4.941053	6.21E-05	T
ENSG0000(	PIGU	5.0207	14.0775	2.768664	6.22E-05	T
ENSG0000(	ANGEL1	1.2035	3.2441	2.565478	6.24E-05	T
ENSG0000(	AC005670	0.0084	0.3117	3.79797	6.26E-05	T
ENSG0000(	EIF2B5	2.6086	8.3126	3.105885	6.27E-05	T
ENSG0000(	AC009486	0.0133	0.2723	3.285966	6.28E-05	T
ENSG0000(	EDRF1	1.3684	2.9893	2.103855	6.29E-05	T
ENSG0000(	CCDC14	1.1809	5.1414	4.091967	6.3E-05	T
ENSG0000(	POLR2K	11.3228	27.0109	2.373402	6.33E-05	T
ENSG0000(	MKI67	4.0901	16.088	3.863392	6.33E-05	T
ENSG0000(	IDH3B	8.4474	19.4861	2.291469	6.33E-05	T
ENSG0000(	FZD6	7.8221	31.0078	3.926711	6.38E-05	T
ENSG0000(	ADA	0.9992	4.9896	4.630277	6.41E-05	T
ENSG0000(	ALG6	1.7144	3.7934	2.145833	6.43E-05	T
ENSG0000(	SUZ12P1	1.5288	3.9362	2.478021	6.44E-05	T
ENSG0000(	PPP1R10	10.7936	22.3129	2.057437	6.46E-05	T
ENSG0000(	CACYBP	7.3025	16.7011	2.269652	6.46E-05	T
ENSG0000(	AC010280	0.0766	0.6007	3.967724	6.48E-05	T
ENSG0000(	NUP50-DT	1.8844	4.7265	2.432221	6.51E-05	T
ENSG0000(	MIS12	2.7282	6.8864	2.470264	6.54E-05	T
ENSG0000(	CHD6	2.9804	6.4912	2.139722	6.55E-05	T
ENSG0000(	AC012640	0.4827	1.3852	2.548824	6.57E-05	T
ENSG0000(	FOXRED2	1.5827	7.6859	4.627028	6.58E-05	T
ENSG0000(	PRKDC	8.1319	35.0779	4.273363	6.58E-05	T
ENSG0000(	RNF215	0.1704	0.5259	2.314719	6.58E-05	T
ENSG0000(	CLPTM1L	6.098	14.6782	2.38435	6.6E-05	T
ENSG0000(	AL353625.	0.3832	1.259	2.8125	6.62E-05	T
ENSG0000(	AC073569	0.4498	2.3723	4.496726	6.63E-05	T
ENSG0000(	RPL7P57	0.0215	0.2382	2.783539	6.64E-05	T
ENSG0000(	CASP8AP2	2.5713	5.4283	2.069517	6.66E-05	T
ENSG0000(	FANCD2	1.0025	3.292	3.076644	6.67E-05	T
ENSG0000(	POLE	1.0155	3.2115	2.968624	6.67E-05	T
ENSG0000(	ETAA1	1.4695	4.4556	2.90258	6.68E-05	T
ENSG0000(	PRPSAP2	2.7347	5.8385	2.094931	6.68E-05	T
ENSG0000(	INTS4	1.5856	3.6171	2.205209	6.71E-05	T
ENSG0000(	ZNF335	2.0508	4.6435	2.205458	6.71E-05	T
ENSG0000(	MRPL9	8.305	17.6437	2.111089	6.73E-05	T
ENSG0000(	CAPN14	85.8425	11.3448	0.133168	6.77E-05	N
ENSG0000(	AC016949	0.4683	2.5341	4.635052	6.77E-05	T
ENSG0000(	NIFK	8.0997	19.1211	2.344122	6.79E-05	T
ENSG0000(	ADAM23	0.7539	4.0546	4.865441	6.79E-05	T
ENSG0000(	HOXB7	0.3291	4.8729	11.58914	6.79E-05	T
ENSG0000(	TANC2	1.366	3.8933	2.723943	6.8E-05	T

ENSG0000(	ICE2	1.5794	3.5904	2.197451	6.81E-05	T
ENSG0000(	TMEM217	1.4767	0.4216	0.330818	6.81E-05	N
ENSG0000(	IRS1	1.6882	7.3661	4.175204	6.82E-05	T
ENSG0000(	RMND1	1.1089	2.5015	2.151956	6.84E-05	T
ENSG0000(	ANKRD20	5.1286	0.3808	0.091956	6.84E-05	N
ENSG0000(	TRIP10	103.6281	24.0283	0.232611	6.85E-05	N
ENSG0000(	L3MBTL3	0.5943	1.5476	2.373038	6.86E-05	T
ENSG0000(	SNHG17	1.5312	5.2699	3.291994	6.87E-05	T
ENSG0000(	PPP1R1C	0.0673	0.4642	3.372385	6.89E-05	T
ENSG0000(	TMEM184	3.8328	11.0035	2.823307	6.89E-05	T
ENSG0000(	AC019205	0.2839	0.9155	2.64522	6.89E-05	T
ENSG0000(	PIGT	1.3733	4.1705	2.898595	6.9E-05	T
ENSG0000(	MRTO4	4.4666	11.3581	2.50911	6.92E-05	T
ENSG0000(	AC080188	1.0952	5.8015	4.937667	6.93E-05	T
ENSG0000(	AC092718	1.3282	5.7607	4.103557	6.94E-05	T
ENSG0000(	CDK5RAP	1.2053	3.6271	2.855359	6.97E-05	T
ENSG0000(	PAXIP1	1.1295	2.5755	2.176088	6.97E-05	T
ENSG0000(	DERL1	5.7328	14.7008	2.537512	6.98E-05	T
ENSG0000(	UACA	15.2444	5.5125	0.365769	6.98E-05	N
ENSG0000(	SNCAIP	0.1568	1.3157	5.51285	6.99E-05	T
ENSG0000(	EPRS	11.6363	26.2994	2.24938	7.04E-05	T
ENSG0000(	EDARADI	0.8503	3.5719	3.863938	7.05E-05	T
ENSG0000(	DHX9	10.4118	32.5635	3.107317	7.05E-05	T
ENSG0000(	NPIPA5	0.0415	0.2139	2.218375	7.07E-05	T
ENSG0000(	MIIP	0.9165	2.4326	2.49149	7.07E-05	T
ENSG0000(	ORC6	0.4071	2.2139	4.563005	7.08E-05	T
ENSG0000(	CEP170	1.2837	3.4656	2.576859	7.1E-05	T
ENSG0000(	NAPEPLD	0.5419	2.0154	3.295529	7.13E-05	T
ENSG0000(	RBBP4	5.3994	14.0312	2.569589	7.16E-05	T
ENSG0000(	NIPSNAP1	3.9959	11.6609	2.871384	7.2E-05	T
ENSG0000(	COLGALT	4.9878	15.9648	3.157514	7.23E-05	T
ENSG0000(	PDIA3P1	0.6805	1.8873	2.546188	7.24E-05	T
ENSG0000(	PKP3	103.5942	46.1823	0.446335	7.24E-05	N
ENSG0000(	HOXA7	2.0786	5.4113	2.529744	7.25E-05	T
ENSG0000(	DHRS1	11.4729	4.6147	0.407391	7.25E-05	N
ENSG0000(	RHBDD3	1.095	3.4306	2.954477	7.26E-05	T
ENSG0000(	LINC0245'	0.0121	0.4455	4.866191	7.26E-05	T
ENSG0000(	PHF6	3.1212	6.7552	2.128151	7.27E-05	T
ENSG0000(	FMNL2	1.6292	8.5192	4.984502	7.27E-05	T
ENSG0000(	MFGE8	2.2143	4.5713	2.018451	7.27E-05	T
ENSG0000(	ACO2	13.8017	28.2491	2.039254	7.29E-05	T
ENSG0000(	BOD1	3.6533	8.382	2.259878	7.3E-05	T
ENSG0000(	CEP68	2.113	5.1696	2.381202	7.31E-05	T
ENSG0000(	SLC4A4	0.7725	0.0913	0.219255	7.31E-05	N
ENSG0000(	CAD	2.1438	8.1038	3.656208	7.32E-05	T
ENSG0000(	BTBD19	0.8974	2.3652	2.471626	7.33E-05	T
ENSG0000(	PRELID2P	0.3821	0.128	0.472931	7.36E-05	N
ENSG0000(	CRLS1	2.503	6.1607	2.405186	7.36E-05	T
ENSG0000(	E2F6	0.9587	3.4237	3.328327	7.38E-05	T
ENSG0000(	ARHGAP1	2.1566	7.8951	3.542985	7.4E-05	T
ENSG0000(	DPY19L3	1.1948	2.629	2.107661	7.4E-05	T
ENSG0000(	PRPF39	2.4779	5.835	2.302262	7.43E-05	T
ENSG0000(	TOMM40	6.3385	20.4413	3.190386	7.43E-05	T
ENSG0000(	NFS1	1.2127	3.131	2.461339	7.45E-05	T
ENSG0000(	EME1	0.1633	0.9938	4.154197	7.46E-05	T
ENSG0000(	ALG1	1.3488	4.4659	3.151505	7.46E-05	T
ENSG0000(	SFPQ	13.3938	40.346	2.997377	7.49E-05	T
ENSG0000(	PTPDC1	0.4035	1.0152	2.214896	7.5E-05	T

ENSG0000(	PNISR	3.3185	7.1524	2.121515	7.53E-05	T
ENSG0000(	TATDN1	1.297	3.9132	2.872727	7.58E-05	T
ENSG0000(	U62317.2	1.0467	3.5068	3.145374	7.62E-05	T
ENSG0000(	IQCG	0.6984	1.9135	2.521919	7.63E-05	T
ENSG0000(	GTPBP3	1.0057	2.2048	2.084471	7.65E-05	T
ENSG0000(	CENPW	4.9838	29.8187	5.885106	7.68E-05	T
ENSG0000(	PRMT7	0.5371	1.3939	2.344844	7.72E-05	T
ENSG0000(	GPN3	2.5928	6.5824	2.481581	7.74E-05	T
ENSG0000(	GABPB1	1.9958	4.5034	2.196488	7.76E-05	T
ENSG0000(	MYNN	3.0808	8.7493	2.782099	7.76E-05	T
ENSG0000(	XPR1	2.7722	10.4876	3.686234	7.77E-05	T
ENSG0000(	CEP85	1.7726	4.4695	2.44019	7.78E-05	T
ENSG0000(	AC104986	0.6725	1.9133	2.606214	7.81E-05	T
ENSG0000(	UTP6	2.3857	5.4711	2.24126	7.82E-05	T
ENSG0000(	B9D1	0.789	1.922	2.274466	7.82E-05	T
ENSG0000(	PIH1D2	1.0576	0.398	0.4302	7.83E-05	N
ENSG0000(	KLC4	0.7192	1.7813	2.296509	7.84E-05	T
ENSG0000(	RPSAP13	0.0206	0.2912	3.243781	7.85E-05	T
ENSG0000(	RTTN	0.4733	1.6863	3.115821	7.87E-05	T
ENSG0000(	PLEKHN1	15.5436	5.8513	0.38043	7.88E-05	N
ENSG0000(	COQ8B	1.1641	2.8259	2.314611	7.88E-05	T
ENSG0000(	GDF7	0.8676	0.155	0.263539	7.89E-05	N
ENSG0000(	DTYMK	2.1427	9.0258	4.069113	7.89E-05	T
ENSG0000(	ZNF670	0.5582	1.7778	2.852932	7.89E-05	T
ENSG0000(	ENY2	2.0058	4.9778	2.41134	7.92E-05	T
ENSG0000(	DDIAS	0.6	3.7287	5.469571	7.94E-05	T
ENSG0000(	TACC3	1.1855	4.8641	3.86161	7.94E-05	T
ENSG0000(	FAM111A	2.2188	7.7296	3.376574	7.94E-05	T
ENSG0000(	FAM72A	0.1246	0.6766	3.457703	7.95E-05	T
ENSG0000(	CEP192	3.9251	9.6718	2.427716	7.95E-05	T
ENSG0000(	RPA3	1.825	5.0872	2.694649	7.96E-05	T
ENSG0000(	LINC0064(	0.3978	1.4438	3.101245	7.97E-05	T
ENSG0000(	METTL26	4.3946	11.3099	2.53858	7.98E-05	T
ENSG0000(	WRAP73	0.7245	1.7132	2.199151	8.01E-05	T
ENSG0000(	ATP5MGL	0.1246	0.5744	3.002671	8.01E-05	T
ENSG0000(	CLCN7	2.3268	6.0214	2.522416	8.02E-05	T
ENSG0000(	APEX1	15.3167	39.9309	2.596593	8.02E-05	T
ENSG0000(	PASK	0.4468	1.2832	2.529627	8.04E-05	T
ENSG0000(	LONP1	3.1312	8.2686	2.589936	8.1E-05	T
ENSG0000(	HIST1H1C	165.3496	365.7787	2.211421	8.11E-05	T
ENSG0000(	DNAJC19I	3.9009	11.9039	3.0003	8.15E-05	T
ENSG0000(	COMMD4	0.8768	2.298	2.454955	8.17E-05	T
ENSG0000(	PUS10	0.7004	1.5603	2.074338	8.17E-05	T
ENSG0000(	SSR1	12.4209	27.4165	2.197646	8.18E-05	T
ENSG0000(	KIAA0930	3.9906	9.0486	2.236493	8.18E-05	T
ENSG0000(	PARP6	1.8458	4.6053	2.418183	8.2E-05	T
ENSG0000(	AP000240.	1.1125	3.1896	2.713072	8.23E-05	T
ENSG0000(	C6orf48	2.3157	5.8037	2.443888	8.27E-05	T
ENSG0000(	CCNB1	5.6103	25.8034	4.536259	8.27E-05	T
ENSG0000(	TDP1	1.7191	4.0898	2.303227	8.28E-05	T
ENSG0000(	ZC3H12A	12.7206	41.3468	3.232828	8.3E-05	T
ENSG0000(	ANXA4	2.6496	5.6411	2.087976	8.3E-05	T
ENSG0000(	PFDN6	1.8197	3.7775	2.019847	8.32E-05	T
ENSG0000(	OR7E14P	5.5329	2.0537	0.382343	8.33E-05	N
ENSG0000(	PSME4	6.373	24.3646	3.779484	8.35E-05	T
ENSG0000(	MSH2	0.9369	3.52	3.491176	8.35E-05	T
ENSG0000(	ASCC3	4.3503	9.3701	2.127969	8.37E-05	T
ENSG0000(	DHX34	1.8429	6.0934	3.187709	8.4E-05	T

ENSG0000(	CDC14A	0.5906	1.7281	2.647118	8.41E-05	T
ENSG0000(	SLC27A5	0.1667	0.5868	2.575178	8.41E-05	T
ENSG0000(	TNFAIP8L	0.7494	2.1415	2.638922	8.42E-05	T
ENSG0000(	ATRAID	14.5501	31.3377	2.145903	8.44E-05	T
ENSG0000(	STK11IP	1.2982	2.9323	2.168717	8.45E-05	T
ENSG0000(	AC019186	0.2513	0.9978	3.124964	8.45E-05	T
ENSG0000(	NLRP1	1.532	3.9239	2.465625	8.48E-05	T
ENSG0000(	TBCCD1	2.1627	6.4229	2.882795	8.52E-05	T
ENSG0000(	DBR1	2.1347	6.4964	2.951806	8.53E-05	T
ENSG0000(	MSN	16.3806	65.1382	3.958485	8.53E-05	T
ENSG0000(	ANKHD1	0.2028	0.5675	2.204425	8.56E-05	T
ENSG0000(	CCDC6	50.974	24.8008	0.487544	8.58E-05	N
ENSG0000(	PON3	0.1262	1.03	4.995579	8.58E-05	T
ENSG0000(	CLCA4	471.5572	149.7003	0.317604	8.59E-05	N
ENSG0000(	RAB25	148.369	67.8456	0.457642	8.6E-05	N
ENSG0000(	SNRNP48	1.3351	3.6871	2.63891	8.6E-05	T
ENSG0000(	PLEKHG6	16.779	4.7491	0.287286	8.61E-05	N
ENSG0000(	EDEM2	4.9216	12.7883	2.566572	8.64E-05	T
ENSG0000(	CD40	1.4912	5.4118	3.463927	8.66E-05	T
ENSG0000(	CPSF1	3.2411	8.0484	2.438838	8.67E-05	T
ENSG0000(	HSPB11	2.2385	5.0943	2.22121	8.71E-05	T
ENSG0000(	UHRF1BP	1.9119	4.8742	2.472389	8.72E-05	T
ENSG0000(	HOXD11	0.002	2.2412	22.95294	8.73E-05	T
ENSG0000(	HNRNPM	13.7179	29.6469	2.15278	8.76E-05	T
ENSG0000(	TRUB1	3.694	8.8151	2.349789	8.83E-05	T
ENSG0000(	C19orf48	2.8182	8.8228	3.057638	8.83E-05	T
ENSG0000(	TOMM40I	2.1741	4.7237	2.121147	8.85E-05	T
ENSG0000(	SMC6	2.566	8.1197	3.083158	8.89E-05	T
ENSG0000(	DUSP12	1.8182	4.6653	2.484256	8.91E-05	T
ENSG0000(	METTL23	2.0151	4.5788	2.212094	8.91E-05	T
ENSG0000(	TBC1D31	1.0362	2.7007	2.464971	8.93E-05	T
ENSG0000(	MKS1	1.0249	2.3019	2.135212	8.94E-05	T
ENSG0000(	KDM1A	5.4681	13.4061	2.425621	8.95E-05	T
ENSG0000(	RRM1	9.3903	21.3787	2.263227	8.96E-05	T
ENSG0000(	MID1	2.6093	5.9755	2.242461	8.97E-05	T
ENSG0000(	HIST1H2B	5.7269	21.1893	3.653624	8.99E-05	T
ENSG0000(	AC022075	0.3798	2.2645	4.928095	9E-05	T
ENSG0000(	PHC1	4.4741	1.9428	0.446602	9E-05	N
ENSG0000(	LINC0169'	1.6318	0.59	0.398429	9.01E-05	N
ENSG0000(	CEP55	1.4906	11.3487	7.197724	9.01E-05	T
ENSG0000(	AC009264	0.0033	2.0448	20.76283	9.02E-05	T
ENSG0000(	NUTF2	6.7908	14.6139	2.135296	9.02E-05	T
ENSG0000(	BCL11B	2.7662	7.341	2.59612	9.05E-05	T
ENSG0000(	KNTC1	1.2407	6.1151	4.635713	9.05E-05	T
ENSG0000(	POLA1	2.0421	5.6666	2.692031	9.06E-05	T
ENSG0000(	SNRPGP2	2.4339	6.6728	2.672876	9.06E-05	T
ENSG0000(	TCF19	3.4958	7.9035	2.225791	9.1E-05	T
ENSG0000(	AL663070.	0.7438	2.5471	3.137118	9.1E-05	T
ENSG0000(	MTO1	0.9913	2.2492	2.152662	9.12E-05	T
ENSG0000(	ZXDC	2.3826	6.0787	2.488802	9.12E-05	T
ENSG0000(	UCN	0.053	0.28	2.48366	9.17E-05	T
ENSG0000(	RACGAP1	4.1603	12.8426	3.037955	9.18E-05	T
ENSG0000(	ERH	21.9947	51.8749	2.35237	9.18E-05	T
ENSG0000(	CAMSAP2	9.3183	18.8649	2.013622	9.19E-05	T
ENSG0000(	MCM10	0.4858	2.7269	4.825708	9.19E-05	T
ENSG0000(	BEAN1	0.3977	0.1108	0.423548	9.2E-05	N
ENSG0000(	AL021707.	0.1699	0.7182	3.031493	9.21E-05	T
ENSG0000(	RNPS1P1	0.2798	1.7229	4.799631	9.21E-05	T

ENSG0000(	CMSS1	0.806	4.0057	4.531678	9.21E-05	T
ENSG0000(	PGRMC1	20.2658	43.4797	2.139847	9.22E-05	T
ENSG0000(	FLAD1	2.7176	7.1826	2.584682	9.23E-05	T
ENSG0000(	AHCTF1	4.3522	12.8234	2.9027	9.28E-05	T
ENSG0000(	WDR62	0.2939	1.3944	3.793856	9.28E-05	T
ENSG0000(	DIO2	48.4292	6.7167	0.140466	9.28E-05	N
ENSG0000(	FLJ45513	0.2696	0.8899	2.678301	9.29E-05	T
ENSG0000(	SRPX2	7.1468	2.7484	0.393056	9.3E-05	N
ENSG0000(	PCYT1A	7.4863	16.8891	2.239445	9.32E-05	T
ENSG0000(	AC114763	0.1523	0.5828	2.706302	9.36E-05	T
ENSG0000(	ZCWPW1	0.3653	0.848	2.037395	9.38E-05	T
ENSG0000(	CKAP2L	0.9167	5.0649	5.080063	9.39E-05	T
ENSG0000(	ARMC10	4.5548	9.5937	2.082517	9.41E-05	T
ENSG0000(	PRNP	24.9697	83.4131	3.331237	9.44E-05	T
ENSG0000(	CLSTN1	22.1687	60.8485	2.736958	9.45E-05	T
ENSG0000(	FAM149A	1.2191	0.2082	0.233644	9.45E-05	N
ENSG0000(	NPM1P25	2.2814	0.9109	0.424498	9.5E-05	N
ENSG0000(	OTUD6B	2.707	7.6966	2.777556	9.51E-05	T
ENSG0000(	IQCC	0.347	0.9366	2.319016	9.54E-05	T
ENSG0000(	BOP1	2.6233	9.556	3.545698	9.55E-05	T
ENSG0000(	MYH9	35.3436	106.2483	3.000494	9.55E-05	T
ENSG0000(	TMEM104	1.7592	4.23	2.328959	9.56E-05	T
ENSG0000(	SLC52A2	2.6588	8.3284	3.055096	9.63E-05	T
ENSG0000(	ABCF3	4.3388	10.9786	2.495855	9.63E-05	T
ENSG0000(	ZNF200	0.5102	1.561	2.722058	9.64E-05	T
ENSG0000(	GTPBP4	3.4471	8.6287	2.460799	9.66E-05	T
ENSG0000(	RN7SKP1(	0	0.4971	5.971	9.66E-05	T
ENSG0000(	Z95115.1	0.1933	0.6939	2.706785	9.68E-05	T
ENSG0000(	PDCD6IP	30.2806	14.4415	0.478644	9.69E-05	N
ENSG0000(	TEDC2	0.1866	0.9768	3.757153	9.7E-05	T
ENSG0000(	AC012213	0.1832	1.3998	5.295904	9.71E-05	T
ENSG0000(	CMTR1	3.2211	8.3871	2.555509	9.71E-05	T
ENSG0000(	UBN1	2.8449	5.8872	2.033074	9.71E-05	T
ENSG0000(	LUC7L2	2.6082	6.1457	2.306218	9.73E-05	T
ENSG0000(	HNRNPA2	16.0593	44.3701	2.751982	9.75E-05	T
ENSG0000(	ANLN	3.5769	19.0651	5.212298	9.77E-05	T
ENSG0000(	SGO1	0.5948	2.4929	3.731865	9.82E-05	T
ENSG0000(	CYREN	2.1471	5.5988	2.536069	9.84E-05	T
ENSG0000(	ZNF480	2.2554	5.2913	2.288911	9.85E-05	T
ENSG0000(	CEBPZ	8.4396	18.7003	2.201543	9.86E-05	T
ENSG0000(	MYC	29.8708	88.3929	2.952637	9.89E-05	T
ENSG0000(	DDX50P1	0.2041	0.7958	2.945742	9.89E-05	T
ENSG0000(	CEP70	3.9695	9.4925	2.357169	9.93E-05	T
ENSG0000(	FAM3C	9.8546	24.3775	2.458913	9.96E-05	T
ENSG0000(	CPNE8-AS	0.0517	0.6428	4.896506	9.97E-05	T
ENSG0000(	AC092903	0.2222	1.2512	4.193669	0.0001	T
ENSG0000(	NRDE2	0.5456	1.3841	2.298792	0.0001	T
ENSG0000(	AC005532	10.5751	1.6554	0.164439	0.000101	N
ENSG0000(	P3H1	0.4911	1.9047	3.391474	0.000101	T
ENSG0000(	NUP88	3.8447	8.9571	2.296017	0.000101	T
ENSG0000(	DNAAF5	1.6251	3.5724	2.128804	0.000101	T
ENSG0000(	HDGF	34.9687	76.8847	2.195254	0.000102	T
ENSG0000(	RNU6-4P	12.4663	36.8043	2.936767	0.000102	T
ENSG0000(	WDR3	1.5129	4.0316	2.561597	0.000102	T
ENSG0000(	ARL13B	1.2359	4.0867	3.133992	0.000102	T
ENSG0000(	PUF60	5.6362	15.5956	2.736237	0.000102	T
ENSG0000(	HAUS2	3.1987	7.009	2.155091	0.000102	T
ENSG0000(	RRP7A	4.1042	10.2988	2.473431	0.000102	T

ENSG0000(	RBM12B	1.9703	6.3018	3.092209	0.000102	T
ENSG0000(	TEX261	6.4328	13.0174	2.007929	0.000102	T
ENSG0000(	MAGOH	5.415	12.8749	2.352656	0.000103	T
ENSG0000(	CENPN	1.8286	7.8305	4.11205	0.000103	T
ENSG0000(	DOP1B	12.6449	5.9727	0.476481	0.000103	N
ENSG0000(	HNRNPA3	0.1478	0.5531	2.635593	0.000103	T
ENSG0000(	TFRC	9.2532	51.6051	5.528065	0.000103	T
ENSG0000(	DOCK7	0.7576	1.6982	2.096782	0.000103	T
ENSG0000(	CBFB	9.2959	19.317	2.06654	0.000103	T
ENSG0000(	CKS1B	2.3408	10.9772	4.538348	0.000104	T
ENSG0000(	FAM204B	0.1789	1.3687	5.266045	0.000104	T
ENSG0000(	DDX20	1.2201	3.07	2.401333	0.000104	T
ENSG0000(	AC092803	0.1236	0.6835	3.504025	0.000104	T
ENSG0000(	RFC2	4.947	15.3686	3.06491	0.000105	T
ENSG0000(	MSL2	5.7918	14.3115	2.446027	0.000105	T
ENSG0000(	LRRC8B	2.6841	8.7885	3.192594	0.000106	T
ENSG0000(	ITGB3BP	1.2805	3.3934	2.530532	0.000106	T
ENSG0000(	HOXA10	0.0937	1.5776	8.660816	0.000106	T
ENSG0000(	POLR3K	1.3974	4.453	3.040604	0.000106	T
ENSG0000(	CTPS1	1.4298	5.1638	3.440842	0.000106	T
ENSG0000(	HIST1H3C	75.2067	292.5156	3.885652	0.000107	T
ENSG0000(	HIST1H2A	17.8435	60.6317	3.384607	0.000107	T
ENSG0000(	ACAA1	9.6115	4.1272	0.435278	0.000107	N
ENSG0000(	LINC0187(	0.4783	3.365	5.9917	0.000107	T
ENSG0000(	SMYD5	2.1142	6.3379	2.907551	0.000107	T
ENSG0000(	ADAM17	3.5588	10.2726	2.834973	0.000107	T
ENSG0000(	ST7	1.4505	3.2516	2.161625	0.000107	T
ENSG0000(	ZFAS1	6.0498	12.6959	2.080702	0.000107	T
ENSG0000(	TERF1	1.5479	4.0199	2.500091	0.000107	T
ENSG0000(	COMMD2	3.7339	10.5323	2.773234	0.000107	T
ENSG0000(	RN7SL454	0.0714	0.6422	4.330222	0.000108	T
ENSG0000(	BRIP1	0.733	3.2831	4.061345	0.000108	T
ENSG0000(	ALDH9A1	44.3524	21.6333	0.488912	0.000108	N
ENSG0000(	GPR137B	3.9177	10.8418	2.723399	0.000108	T
ENSG0000(	SNRPA1	3.0622	8.6135	2.755518	0.000108	T
ENSG0000(	NUBPL	0.671	1.5595	2.152399	0.000108	T
ENSG0000(	CD109	6.8204	16.7132	2.429513	0.000109	T
ENSG0000(	C2orf74	0.0425	0.2177	2.229474	0.000109	T
ENSG0000(	SLC25A5	140.1135	295.6338	2.109168	0.000109	T
ENSG0000(	PPIL1	6.7474	15.9238	2.340129	0.00011	T
ENSG0000(	KIAA1324	0.4884	2.2939	4.068491	0.00011	T
ENSG0000(	CHRAC1	6.4272	14.8719	2.293771	0.00011	T
ENSG0000(	DMTF1	2.4408	6.2179	2.486579	0.000111	T
ENSG0000(	FANCM	0.7246	2.2793	2.885399	0.000111	T
ENSG0000(	XYLT1	4.7881	2.3015	0.491295	0.000111	N
ENSG0000(	RN7SKP9(	83.8203	236.5384	2.819799	0.000111	T
ENSG0000(	ZC3H7A	2.8422	6.3471	2.191251	0.000111	T
ENSG0000(	TAF10	0.3609	0.8349	2.028423	0.000111	T
ENSG0000(	C20orf27	2.3415	7.6892	3.190334	0.000111	T
ENSG0000(	RBM15	3.3245	7.9053	2.337655	0.000112	T
ENSG0000(	BICDL2	18.7033	7.9956	0.430541	0.000112	N
ENSG0000(	FUT3	43.0703	15.2999	0.356724	0.000112	N
ENSG0000(	CCDC88A	0.5485	1.869	3.036237	0.000112	T
ENSG0000(	NUS1P1	0.2326	0.7937	2.687011	0.000112	T
ENSG0000(	PRPF38B	3.906	8.2417	2.082302	0.000113	T
ENSG0000(	POLR2D	2.6726	5.5739	2.046419	0.000114	T
ENSG0000(	NSMAF	3.7791	9.3966	2.448145	0.000114	T
ENSG0000(	CDA	61.5257	17.432	0.284492	0.000114	N

ENSG0000(	ACBD3-A!	4.4956	1.4228	0.33136	0.000115	N
ENSG0000(	NIPBL-DT	0.8067	2.0765	2.400463	0.000115	T
ENSG0000(	MKNK1	1.756	3.8202	2.112177	0.000116	T
ENSG0000(	RNU5A-8I	0.7088	8.712	10.89515	0.000116	T
ENSG0000(	GNAI1	1.0585	2.5149	2.257143	0.000116	T
ENSG0000(	SGMS1	4.3418	11.3736	2.583097	0.000116	T
ENSG0000(	PIK3R4	3.8734	12.5801	3.191247	0.000117	T
ENSG0000(	ACADM	22.5311	7.7464	0.346709	0.000117	N
ENSG0000(	AIF1L	90.9581	4.4887	0.050393	0.000117	N
ENSG0000(	BAX	4.7985	10.9482	2.255425	0.000117	T
ENSG0000(	NIPAL1	18.1346	6.6476	0.370044	0.000117	N
ENSG0000(	RIC8B	0.5735	1.505	2.383073	0.000117	T
ENSG0000(	KIF4A	0.8205	5.2234	5.783161	0.000118	T
ENSG0000(	DIAPH3	0.612	2.6743	3.896489	0.000118	T
ENSG0000(	MIS18BP1	2.8455	6.6195	2.281277	0.000118	T
ENSG0000(	TIMM44	2.2015	5.5326	2.44736	0.000119	T
ENSG0000(	ALG3	4.8181	19.6066	4.006954	0.000119	T
ENSG0000(	RPUSD1	2.2555	5.9172	2.554532	0.000119	T
ENSG0000(	ACOX1	24.5323	10.9631	0.44913	0.000119	N
ENSG0000(	HOXD8	0.2135	1.2431	4.284211	0.00012	T
ENSG0000(	POLQ	0.8862	3.9477	4.10434	0.00012	T
ENSG0000(	AC005332	0.2758	1.0087	2.950239	0.00012	T
ENSG0000(	DGCR8	2.1604	4.5724	2.067068	0.00012	T
ENSG0000(	CEP63	1.0339	2.459	2.256813	0.00012	T
ENSG0000(	CUTA	9.6305	20.0451	2.070305	0.00012	T
ENSG0000(	AC013652	0.1245	0.8168	4.083742	0.00012	T
ENSG0000(	NOL10	4.3062	12.5619	2.873655	0.00012	T
ENSG0000(	HAUS5	1.2067	3.6413	2.863167	0.000121	T
ENSG0000(	CDK1	2.2145	13.1141	5.709268	0.000121	T
ENSG0000(	ATP6V1C1	3.3782	12.37	3.585188	0.000121	T
ENSG0000(	PGP	1.3092	3.8215	2.782785	0.000122	T
ENSG0000(	TP53BP1	1.5609	3.6773	2.274249	0.000122	T
ENSG0000(	TMEM8A	17.5244	6.8699	0.395469	0.000122	N
ENSG0000(	NUP50	8.3789	17.8535	2.117433	0.000122	T
ENSG0000(	POU6F2	0.0037	1.0661	11.24494	0.000122	T
ENSG0000(	CAP2P1	0.3292	1.1827	2.988583	0.000122	T
ENSG0000(	LPGAT1	4.3834	9.4953	2.140184	0.000122	T
ENSG0000(	CEP72	0.8641	3.185	3.407323	0.000122	T
ENSG0000(	GART	4.3987	10.7532	2.412519	0.000122	T
ENSG0000(	NMNAT3	0.2332	1.3496	4.35054	0.000122	T
ENSG0000(	BMS1P12	1.3607	0.0874	0.128295	0.000123	N
ENSG0000(	MAZ	13.8797	34.9254	2.505447	0.000123	T
ENSG0000(	THUMPD2	1.4472	4.1986	2.778309	0.000123	T
ENSG0000(	RBM15-A!	0.0265	0.1905	2.296443	0.000123	T
ENSG0000(	PTDSS1	5.1515	15.3853	2.948738	0.000123	T
ENSG0000(	EXOSC10	4.1728	8.6814	2.055186	0.000123	T
ENSG0000(	SAC3D1	0.8608	2.6794	2.892798	0.000123	T
ENSG0000(	DARS2	1.4329	4.4864	2.991976	0.000124	T
ENSG0000(	IFT172	0.884	2.2864	2.425203	0.000124	T
ENSG0000(	RPS29P16	2.1631	5.09	2.293314	0.000124	T
ENSG0000(	HIST1H2E	1.8277	7.1553	3.763708	0.000124	T
ENSG0000(	TATDN3	1.0303	2.1866	2.023003	0.000124	T
ENSG0000(	USP13	1.056	2.2653	2.046107	0.000124	T
ENSG0000(	ITPR3	6.4832	15.8947	2.429624	0.000125	T
ENSG0000(	LRRC49	0.1472	0.5745	2.72856	0.000125	T
ENSG0000(	HADH	1.4549	3.271	2.167985	0.000125	T
ENSG0000(	SLC35C2	2.4604	5.4914	2.183799	0.000125	T
ENSG0000(	ERCC2	0.8769	2.3095	2.466476	0.000126	T

ENSG0000(	AC096677	0.3254	1.0189	2.63023	0.000126	T
ENSG0000(	IQCB1	2.4955	9.5932	3.734618	0.000126	T
ENSG0000(	ASPSCR1	0.5562	1.4538	2.367876	0.000126	T
ENSG0000(	DBF4	1.6128	6.2868	3.728865	0.000126	T
ENSG0000(	AAR2	3.6204	7.7118	2.09972	0.000126	T
ENSG0000(	BACH1-IT	0.4931	2.0697	3.658236	0.000126	T
ENSG0000(	ARHGAP2	21.6952	7.5112	0.349215	0.000126	N
ENSG0000(	BRAT1	1.1113	3.1548	2.68703	0.000126	T
ENSG0000(	POU5F1	0.0405	0.326	3.032028	0.000126	T
ENSG0000(	FASTKD2	3.6831	8.0067	2.142872	0.000126	T
ENSG0000(	KIF14	0.5475	3.9232	6.213436	0.000127	T
ENSG0000(	MRPL24	6.2454	17.0202	2.698049	0.000127	T
ENSG0000(	RNF139	10.0993	20.6666	2.036081	0.000127	T
ENSG0000(	KRT16P4	0.3946	2.961	6.188839	0.000128	T
ENSG0000(	SHLD1	1.1178	2.3557	2.016505	0.000128	T
ENSG0000(	AL136040.	0.2726	0.678	2.08803	0.000128	T
ENSG0000(	TENT5B	43.2753	15.0063	0.34827	0.000129	N
ENSG0000(	CBX3	8.8764	27.9996	3.130386	0.000129	T
ENSG0000(	SLC35B4	1.3642	3.1157	2.196216	0.000129	T
ENSG0000(	LUC7L3	4.916	10.1861	2.050658	0.000129	T
ENSG0000(	SLC35A5	3.4101	7.1001	2.051252	0.000129	T
ENSG0000(	UBR1	2.2273	4.988	2.186224	0.00013	T
ENSG0000(	EIF5A2	0.3819	1.6327	3.595559	0.00013	T
ENSG0000(	NUTM2A-	1.2469	2.9743	2.282501	0.000131	T
ENSG0000(	EBAG9	3.289	8.1332	2.429389	0.000131	T
ENSG0000(	POMT2	0.5212	1.2719	2.208467	0.000131	T
ENSG0000(	BTG3	5.1849	16.4446	3.130542	0.000131	T
ENSG0000(	YTHDF1	7.9836	17.846	2.22005	0.000132	T
ENSG0000(	GPR89A	0.6402	1.8863	2.683464	0.000132	T
ENSG0000(	AC099343	0.5611	1.2431	2.031614	0.000132	T
ENSG0000(	TRAIP	0.3619	1.258	2.94003	0.000132	T
ENSG0000(	ALG8	3.4695	8.5626	2.426838	0.000132	T
ENSG0000(	TMEM106	5.8224	15.568	2.645549	0.000132	T
ENSG0000(	SLC39A7	10.6067	22.5162	2.112341	0.000133	T
ENSG0000(	ALKBH2	1.4853	4.2325	2.732921	0.000133	T
ENSG0000(	RNF19A	7.9451	16.0308	2.005047	0.000133	T
ENSG0000(	ZNF251	0.9545	2.2903	2.266761	0.000133	T
ENSG0000(	ANKRD20	20.6834	4.3761	0.215369	0.000133	N
ENSG0000(	UCN2	0.2322	1.2262	3.992173	0.000134	T
ENSG0000(	SFXN5	0.3508	1.0255	2.496673	0.000134	T
ENSG0000(	PCNX2	0.9613	2.0804	2.054462	0.000134	T
ENSG0000(	MAPKAPI	1.2929	3.2378	2.396295	0.000134	T
ENSG0000(	NCAPG2	1.3235	6.034	4.309097	0.000134	T
ENSG0000(	HIST1H2A	65.2038	172.9918	2.650562	0.000135	T
ENSG0000(	CDS1	25.94	12.6229	0.488591	0.000135	N
ENSG0000(	TEAD1	22.3318	10.1393	0.456464	0.000136	N
ENSG0000(	ZNF263	1.3457	3.2698	2.330912	0.000137	T
ENSG0000(	LINC0217	3.2936	0.7853	0.260873	0.000137	N
ENSG0000(	FCF1	4.6403	10.0145	2.133726	0.000137	T
ENSG0000(	NPM3	5.9593	17.3391	2.878072	0.000138	T
ENSG0000(	DHX33	1.8275	5.8699	3.097224	0.000138	T
ENSG0000(	SNORA74	1.8538	6.3423	3.297318	0.000138	T
ENSG0000(	PAXBP1	2.2057	5.3795	2.376502	0.000138	T
ENSG0000(	SNRNP20(	8.0558	26.2103	3.225962	0.000138	T
ENSG0000(	ZSWIM4	11.2137	5.1197	0.461361	0.000139	N
ENSG0000(	PDIA4	16.3788	49.22	2.992936	0.000139	T
ENSG0000(	FUT6	28.4663	8.2116	0.290958	0.000139	N
ENSG0000(	NTPCR	0.6988	1.6589	2.201928	0.000139	T

ENSG0000(	TTC14	1.8158	6.1316	3.25274	0.00014	T
ENSG0000(	XRCC1	2.0957	4.7586	2.21278	0.00014	T
ENSG0000(	HIST1H2A	12.1091	53.6835	4.405198	0.00014	T
ENSG0000(	USF1	5.9511	12.2403	2.039348	0.00014	T
ENSG0000(	METTL1	1.4609	4.1382	2.715228	0.000141	T
ENSG0000(	RPL7P13	0.7638	1.6438	2.018754	0.000141	T
ENSG0000(	MMEL1	1.3157	0.2592	0.253726	0.000141	N
ENSG0000(	S100PBP	1.0288	2.4663	2.273476	0.000141	T
ENSG0000(	CACHD1	1.5212	4.5761	2.884345	0.000141	T
ENSG0000(	ZDBF2	0.8704	2.8299	3.01927	0.000141	T
ENSG0000(	MUC1	51.6006	9.0114	0.176234	0.000141	N
ENSG0000(	PHF14	1.3968	4.0194	2.752138	0.000142	T
ENSG0000(	AKR1B10	38.4651	170.0622	4.412337	0.000142	T
ENSG0000(	SDHAP3	0.6692	1.6113	2.224779	0.000142	T
ENSG0000(	EIF3EP1	0.7508	1.8806	2.327927	0.000142	T
ENSG0000(	COG7	2.352	4.8284	2.009951	0.000142	T
ENSG0000(	RYK	7.0779	16.8227	2.357612	0.000142	T
ENSG0000(	METTL17	2.4445	5.8221	2.327412	0.000142	T
ENSG0000(	PARP1	4.6494	15.0877	3.197814	0.000143	T
ENSG0000(	PLBD2	4.6179	12.4657	2.66341	0.000143	T
ENSG0000(	IFNGR1	13.4957	62.755	4.623153	0.000143	T
ENSG0000(	FAAH	1.4737	3.7814	2.466417	0.000143	T
ENSG0000(	ATAD3B	0.9524	2.6561	2.618871	0.000143	T
ENSG0000(	MPV17L2	1.4786	3.3055	2.157291	0.000144	T
ENSG0000(	IL12A	2.8301	0.424	0.178833	0.000144	N
ENSG0000(	HIST1H2E	35.4247	140.6561	3.962204	0.000144	T
ENSG0000(	HMG20B	6.881	15.8255	2.281263	0.000144	T
ENSG0000(	AF127577.	0.0046	0.5342	6.063098	0.000144	T
ENSG0000(	GALNT18	0.7097	3.8473	4.875015	0.000144	T
ENSG0000(	AC015799	0.5479	1.9089	3.100633	0.000145	T
ENSG0000(	RAD54L	0.5999	4.4211	6.459637	0.000145	T
ENSG0000(	RNU6-583	0.0878	1.0857	6.313632	0.000145	T
ENSG0000(	CCNQ	2.9159	7.458	2.506051	0.000146	T
ENSG0000(	NBN	5.6237	13.4607	2.369219	0.000146	T
ENSG0000(	ST7-OT4	0.1231	0.5909	3.096818	0.000147	T
ENSG0000(	KIF18A	0.5483	3.6408	5.770168	0.000147	T
ENSG0000(	DPH2	2.5541	6.8429	2.615915	0.000147	T
ENSG0000(	PTPN2	2.1433	5.2517	2.385637	0.000147	T
ENSG0000(	GGT6	18.6765	7.5463	0.407227	0.000148	N
ENSG0000(	MYO1B	5.5523	24.5808	4.366506	0.000148	T
ENSG0000(	MAN2C1	1.6983	3.8219	2.180893	0.000148	T
ENSG0000(	XCL1	0.16	1.6262	6.639231	0.000148	T
ENSG0000(	SLC25A33	0.9269	3.2582	3.270231	0.000149	T
ENSG0000(	CSPP1	1.7117	5.073	2.855329	0.000149	T
ENSG0000(	ATG3	5.9624	14.0621	2.336055	0.000149	T
ENSG0000(	MYOF	11.4206	27.0138	2.353506	0.000149	T
ENSG0000(	NOP56P1	0.4876	1.9795	3.538972	0.000149	T
ENSG0000(	SAFB2	2.2207	5.3683	2.356315	0.00015	T
ENSG0000(	AC006017	0.2879	1.341	3.714875	0.00015	T
ENSG0000(	LAS1L	2.3602	5.5774	2.307699	0.00015	T
ENSG0000(	SUPV3L1	2.5178	5.8565	2.275384	0.00015	T
ENSG0000(	U2AF2	15.1725	31.2905	2.055361	0.00015	T
ENSG0000(	GPR89B	0.5346	1.2863	2.184526	0.000151	T
ENSG0000(	PABPN1	4.451	9.6682	2.146385	0.000151	T
ENSG0000(	TRNP1	72.6766	13.0572	0.180789	0.000151	N
ENSG0000(	NUSAP1	0.3823	2.0166	4.388555	0.000152	T
ENSG0000(	ZBTB11	3.9954	8.1363	2.01111	0.000152	T
ENSG0000(	STXBP4	0.3977	1.032	2.274463	0.000152	T

ENSG0000(	SERP1	21.3964	44.1106	2.056651	0.000153	T
ENSG0000(	AC068870	0.0409	0.2362	2.386089	0.000153	T
ENSG0000(	AL133243.	0.486	2.6627	4.714505	0.000153	T
ENSG0000(	CEP120	1.518	3.6565	2.321693	0.000153	T
ENSG0000(	PABPC1L	0.6016	2.8617	4.221351	0.000154	T
ENSG0000(	PLCD1	10.4409	4.0207	0.390925	0.000154	N
ENSG0000(	SLC16A10	0.0482	0.6619	5.141026	0.000154	T
ENSG0000(	HIST1H3F	38.1042	141.5596	3.707959	0.000155	T
ENSG0000(	SPC24	0.8316	3.6314	4.005367	0.000155	T
ENSG0000(	CYP4F29P	54.5816	4.5629	0.085274	0.000155	N
ENSG0000(	GNL3L	4.0781	8.8531	2.142864	0.000155	T
ENSG0000(	TPI1P2	0.2821	0.7345	2.183983	0.000155	T
ENSG0000(	NONO	23.854	49.797	2.083034	0.000155	T
ENSG0000(	STIL	0.9222	5.4973	5.475739	0.000155	T
ENSG0000(	DBF4B	0.2361	0.7802	2.618863	0.000156	T
ENSG0000(	RNU6-942	2.7751	9.6321	3.384961	0.000157	T
ENSG0000(	ICE1	3.6088	9.2361	2.517283	0.000157	T
ENSG0000(	HIST1H1E	68.0501	199.3599	2.926773	0.000157	T
ENSG0000(	AC005042	0.3287	1.298	3.261022	0.000157	T
ENSG0000(	PPDPF	530.1993	174.4769	0.329204	0.000157	N
ENSG0000(	SLC4A3	0.4763	1.6871	3.100989	0.000157	T
ENSG0000(	HMMR	1.1372	4.3954	3.633527	0.000157	T
ENSG0000(	EEFSEC	3.8855	11.1192	2.815004	0.000157	T
ENSG0000(	COL7A1	3.3543	12.0667	3.52219	0.000157	T
ENSG0000(	PSMA3-AS	2.0136	4.7791	2.308431	0.000158	T
ENSG0000(	ZNF280C	1.1167	3.9993	3.369195	0.000158	T
ENSG0000(	DENND4B	1.7804	4.6703	2.536854	0.000158	T
ENSG0000(	PTMAP5	1.8967	5.5831	2.846246	0.000158	T
ENSG0000(	TTPAL	2.3686	5.2375	2.162157	0.000159	T
ENSG0000(	SMIM8	0.3607	0.9278	2.230953	0.000159	T
ENSG0000(	CHIC1	0.7065	2.8683	3.680471	0.000159	T
ENSG0000(	SLK	64.9849	30.6717	0.472793	0.000159	N
ENSG0000(	TIAM2	0.218	0.6309	2.298428	0.000159	T
ENSG0000(	FOXP4-AS	0.0234	0.3781	3.874392	0.00016	T
ENSG0000(	DPP3	3.7292	9.8077	2.587407	0.000161	T
ENSG0000(	NIT2	1.6343	4.4764	2.638759	0.000161	T
ENSG0000(	ASXL1	1.6249	4.5413	2.690765	0.000161	T
ENSG0000(	MAP3K13	2.1952	6.2132	2.75061	0.000161	T
ENSG0000(	RBM4B	1.588	3.3081	2.019017	0.000161	T
ENSG0000(	NDUFB5	8.6978	18.8204	2.150583	0.000162	T
ENSG0000(	PPP5C	2.5902	5.4867	2.076686	0.000162	T
ENSG0000(	ARAP3	0.5221	1.636	2.790548	0.000162	T
ENSG0000(	CCDC191	0.5631	1.3739	2.222742	0.000162	T
ENSG0000(	CEP152	0.5106	1.7788	3.076973	0.000162	T
ENSG0000(	ZNF318	1.9328	4.4339	2.230372	0.000162	T
ENSG0000(	HSPA8P1	0.6017	1.4756	2.245404	0.000163	T
ENSG0000(	FUBP1	5.9989	17.0522	2.812343	0.000163	T
ENSG0000(	RTKN	2.1407	4.7541	2.166332	0.000163	T
ENSG0000(	RNPS1	6.2788	14.3666	2.267919	0.000163	T
ENSG0000(	LRP1B	0.0427	0.2594	2.51857	0.000163	T
ENSG0000(	TCEAL9	6.9884	15.5397	2.206379	0.000163	T
ENSG0000(	NEDD1	2.8296	7.5557	2.613224	0.000163	T
ENSG0000(	BRF1	0.6792	1.5234	2.083419	0.000163	T
ENSG0000(	SNRPB	21.2192	50.8494	2.389836	0.000163	T
ENSG0000(	WDR73	0.748	1.7057	2.129363	0.000164	T
ENSG0000(	HMBS	0.832	1.9597	2.209979	0.000164	T
ENSG0000(	AC130895	0.5962	2.7283	4.062482	0.000164	T
ENSG0000(	MIR1244-4	1.8622	6.5823	3.405514	0.000164	T

ENSG0000(	USP49	0.3554	0.8385	2.060826	0.000164	T
ENSG0000(	OBSL1	0.7841	2.4927	2.932587	0.000164	T
ENSG0000(	LIPA	6.886	16.9958	2.447151	0.000164	T
ENSG0000(	HIST1H4A	18.427	59.4293	3.213111	0.000165	T
ENSG0000(	DEPDC1	1.0258	5.0424	4.567774	0.000165	T
ENSG0000(	GPATCH4	1.6586	4.3408	2.52519	0.000165	T
ENSG0000(	SLC25A24	3.5711	8.5823	2.36504	0.000165	T
ENSG0000(	AC026412	0.6792	2.2417	3.005262	0.000166	T
ENSG0000(	CCDC134	3.9013	7.9395	2.009222	0.000167	T
ENSG0000(	PNKP	2.1421	5.6246	2.553231	0.000167	T
ENSG0000(	VNN1	0.1172	10.1002	46.96225	0.000167	T
ENSG0000(	CDCA5	0.907	6.3038	6.359285	0.000167	T
ENSG0000(	DNAJC11	4.0032	8.1789	2.017669	0.000167	T
ENSG0000(	CABLES2	1.1201	3.3755	2.848537	0.000168	T
ENSG0000(	LINC0024(	0.0433	0.3948	3.452896	0.000168	T
ENSG0000(	CD58	6.6505	17.6145	2.624176	0.000168	T
ENSG0000(	CUL7	1.8649	4.1737	2.175022	0.000168	T
ENSG0000(	ALMS1-IT	0.1272	0.5478	2.851232	0.000168	T
ENSG0000(	SMC2	2.7731	7.8118	2.75375	0.000168	T
ENSG0000(	MGLL	26.7629	8.7082	0.327895	0.000168	N
ENSG0000(	GAS2L1	3.6717	8.1495	2.18721	0.000168	T
ENSG0000(	GIPC1	50.3864	23.3171	0.46383	0.000169	N
ENSG0000(	PDE8A	1.9996	4.2078	2.051724	0.000169	T
ENSG0000(	TRMT112	15.4035	39.2744	2.53971	0.000169	T
ENSG0000(	KIF20B	1.8001	6.0137	3.217567	0.00017	T
ENSG0000(	ALDH16A	2.2075	6.3107	2.778202	0.00017	T
ENSG0000(	RN7SL434	0.2855	0.9418	2.702464	0.00017	T
ENSG0000(	AC096992	0.2618	1.1486	3.451078	0.000171	T
ENSG0000(	AL359915.	0.4325	1.2378	2.5123	0.000171	T
ENSG0000(	CERNA2	1.3491	9.3544	6.524325	0.000171	T
ENSG0000(	SNRPC	12.4412	34.1394	2.730153	0.000171	T
ENSG0000(	PLIN3	65.9066	22.6441	0.344573	0.000171	N
ENSG0000(	WDCP	1.1569	2.915	2.398759	0.000172	T
ENSG0000(	CCNG2	54.0123	8.9072	0.166454	0.000172	N
ENSG0000(	MRPL13	5.1092	11.511	2.228941	0.000172	T
ENSG0000(	IQCK	0.4146	1.0475	2.229887	0.000172	T
ENSG0000(	HSF1	7.1205	16.126	2.247213	0.000172	T
ENSG0000(	MZT1	1.9966	7.4241	3.588715	0.000172	T
ENSG0000(	NCDN	1.2372	3.6362	2.794047	0.000173	T
ENSG0000(	ZNF148	5.1997	11.1492	2.122611	0.000173	T
ENSG0000(	NUP205	5.2426	14.7739	2.784019	0.000173	T
ENSG0000(	OPA1	7.9186	19.9233	2.497107	0.000173	T
ENSG0000(	CRNDE	0.9896	2.7633	2.627845	0.000173	T
ENSG0000(	PTRH2	1.2492	3.2188	2.459828	0.000174	T
ENSG0000(	STIP1	11.3967	28.9079	2.52315	0.000174	T
ENSG0000(	HMGXB3	2.5978	5.7841	2.181073	0.000174	T
ENSG0000(	UBE3D	0.4019	0.9746	2.141064	0.000174	T
ENSG0000(	AP000577.	0.1853	0.7932	3.13074	0.000174	T
ENSG0000(	EEF1AKM	0.5268	3.0612	5.043395	0.000174	T
ENSG0000(	KAZN	7.4122	3.3868	0.464152	0.000175	N
ENSG0000(	LMNB1	3.6374	15.0544	4.054797	0.000175	T
ENSG0000(	ARMC1	6.4545	16.3648	2.511984	0.000175	T
ENSG0000(	KRT32	2.0197	0.4579	0.263198	0.000175	N
ENSG0000(	GOLGA7E	0.3582	0.9913	2.381711	0.000175	T
ENSG0000(	RSL24D1	13.0271	26.5712	2.031766	0.000176	T
ENSG0000(	AC108463	0.0469	0.8252	6.298162	0.000176	T
ENSG0000(	VRK1	4.4351	15.6294	3.468369	0.000176	T
ENSG0000(	PLK1	1.1283	6.9844	5.767646	0.000178	T

ENSG0000(	LMCD1-A	0.0794	0.2956	2.205128	0.000178	T
ENSG0000(	DRG2	1.6843	3.7902	2.180239	0.000178	T
ENSG0000(	TSN	6.6523	15.1054	2.251885	0.000179	T
ENSG0000(	ZNF558	0.8396	1.9598	2.192209	0.000179	T
ENSG0000(	DISC1	0.1561	0.6671	2.995314	0.000179	T
ENSG0000(	DNTTIP2	4.4472	9.5551	2.123307	0.000179	T
ENSG0000(	NDE1	3.3359	9.4697	2.785209	0.00018	T
ENSG0000(	UBR5	8.2255	17.3508	2.096066	0.00018	T
ENSG0000(	NUP160	2.9678	8.0898	2.6696	0.00018	T
ENSG0000(	STIP1P3	0.0901	0.4016	2.638611	0.00018	T
ENSG0000(	TTC32	0.9686	3.2453	3.130545	0.000181	T
ENSG0000(	CHAF1A	2.3738	6.9161	2.836163	0.000181	T
ENSG0000(	AL117329.	0.0213	3.1227	26.56801	0.000181	T
ENSG0000(	HNRNPLL	3.144	7.117	2.224723	0.000181	T
ENSG0000(	AHSA1	9.3306	19.6148	2.090514	0.000182	T
ENSG0000(	AC004980	0.9803	4.381	4.147922	0.000182	T
ENSG0000(	NLRX1	17.5477	7.6908	0.441463	0.000183	N
ENSG0000(	PAQR8	17.2244	3.2579	0.193825	0.000183	N
ENSG0000(	HIST2H2A	28.9098	108.8699	3.756313	0.000183	T
ENSG0000(	SEC22A	1.8229	4.1052	2.186905	0.000183	T
ENSG0000(	GFM1	2.6421	12.3346	4.5347	0.000183	T
ENSG0000(	ARMC10P	0.3398	0.9963	2.492724	0.000183	T
ENSG0000(	LY6E-DT	0.1363	0.7257	3.494287	0.000184	T
ENSG0000(	MIR1244-2	29.8178	99.6096	3.332785	0.000184	T
ENSG0000(	EXOSC8	2.4763	5.2123	2.061988	0.000184	T
ENSG0000(	CCHCR1	1.0415	2.4089	2.197898	0.000184	T
ENSG0000(	IL1RN	933.5492	258.9545	0.277464	0.000184	N
ENSG0000(	EIF2AK3	4.6293	9.8687	2.10786	0.000185	T
ENSG0000(	FP325332.	0.0622	0.2659	2.255857	0.000185	T
ENSG0000(	AC134407	0.0277	0.1784	2.18011	0.000185	T
ENSG0000(	RNF222	19.2512	3.9774	0.210705	0.000186	N
ENSG0000(	SIRT6	1.7013	3.5482	2.025315	0.000186	T
ENSG0000(	SNORA22	5.6783	17.568	3.057647	0.000186	T
ENSG0000(	GABPB1-A	0.2532	1.0317	3.204134	0.000186	T
ENSG0000(	UGT1A7	7.9214	2.6399	0.341574	0.000186	N
ENSG0000(	HACD3	3.7933	12.3695	3.20281	0.000187	T
ENSG0000(	SCIN	4.1644	0.5081	0.142599	0.000187	N
ENSG0000(	RAB26	0.2347	1.086	3.543472	0.000187	T
ENSG0000(	NOL9	2.7687	6.0203	2.133475	0.000188	T
ENSG0000(	AC138150	0.0639	0.2733	2.277608	0.000188	T
ENSG0000(	ZHX2	4.7729	11.4923	2.378932	0.000188	T
ENSG0000(	EAF2	0.5652	3.4587	5.34982	0.000188	T
ENSG0000(	PFN2	4.832	32.3857	6.586719	0.000189	T
ENSG0000(	MPC2	5.6454	12.2279	2.145699	0.000189	T
ENSG0000(	KIAA1614	0.523	1.6515	2.811396	0.000189	T
ENSG0000(	CDKN2AI	4.0183	9.1614	2.248841	0.000189	T
ENSG0000(	RPS3AP23	0	0.8764	9.764	0.00019	T
ENSG0000(	COA6	4.4384	16.057	3.560065	0.000191	T
ENSG0000(	RORC	1.1273	0.2639	0.296505	0.000191	N
ENSG0000(	KIF24	0.5044	1.2389	2.215255	0.000192	T
ENSG0000(	TMEM201	1.2011	3.624	2.862194	0.000192	T
ENSG0000(	LINC0157	0.3104	1.5156	3.936647	0.000192	T
ENSG0000(	PTPRS	5.6705	17.3068	3.016515	0.000192	T
ENSG0000(	SF3A3	5.4547	12.6335	2.292383	0.000193	T
ENSG0000(	PANK4	1.2429	2.9701	2.286172	0.000194	T
ENSG0000(	PAQR3	0.9535	2.4645	2.434267	0.000194	T
ENSG0000(	RAD51AP	0.8706	5.8792	6.160313	0.000194	T
ENSG0000(	ATP1B3	33.9654	170.6265	5.011727	0.000194	T

ENSG0000(	CCDC59	2.5749	5.6243	2.140005	0.000194	T
ENSG0000(	CRISPLD1	0.4626	1.145	2.21294	0.000195	T
ENSG0000(	RNU6-104	1.1097	8.5093	7.116888	0.000195	T
ENSG0000(	RPGRIP1L	0.2348	0.7757	2.615591	0.000196	T
ENSG0000(	NSUN5	1.3362	4.1953	2.990739	0.000196	T
ENSG0000(	LINC0127(	0.0047	0.356	4.355301	0.000196	T
ENSG0000(	PTBP1	19.782	40.6862	2.051413	0.000197	T
ENSG0000(	GNA15	64.5921	30.0383	0.465873	0.000197	N
ENSG0000(	TTC9	88.7044	15.1934	0.172214	0.000197	N
ENSG0000(	GNPNAT1	4.4679	9.1347	2.021651	0.000198	T
ENSG0000(	G2E3	1.772	4.3851	2.395887	0.000198	T
ENSG0000(	SPATA5	1.0838	3.2205	2.80495	0.000199	T
ENSG0000(	SNAP47	1.2337	3.6224	2.791032	0.000199	T
ENSG0000(	DSCC1	0.5577	3.4001	5.321727	0.000199	T
ENSG0000(	RN7SL481	0.2731	1.5407	4.397481	0.000199	T
ENSG0000(	CENPX	7.7889	18.2285	2.323328	0.0002	T
ENSG0000(	SH3BP4	1.8803	8.0326	4.106752	0.0002	T
ENSG0000(	PARL	1.1991	3.9689	3.132091	0.0002	T
ENSG0000(	SLC34A3	0.4698	0.0974	0.346437	0.0002	N
ENSG0000(	VMA21	7.8005	18.5309	2.358193	0.0002	T
ENSG0000(	TMEM68	1.4156	3.3478	2.274875	0.000201	T
ENSG0000(	THBD	11.8843	31.158	2.608246	0.000201	T
ENSG0000(	CHAF1B	0.6965	2.6243	3.420339	0.000201	T
ENSG0000(	AC090617	0.4031	1.537	3.253826	0.000202	T
ENSG0000(	RAD1	1.7273	4.6841	2.618125	0.000202	T
ENSG0000(	STAG3L5I	0.3671	1.3231	3.046671	0.000202	T
ENSG0000(	ACSS1	1.3585	3.1758	2.246006	0.000202	T
ENSG0000(	RNU6-6P	4.9707	15.8281	3.141203	0.000202	T
ENSG0000(	B4GALT2	4.6798	10.3945	2.195594	0.000203	T
ENSG0000(	PIGX	2.9642	15.0606	4.947654	0.000203	T
ENSG0000(	SEC61A1	27.3731	68.6775	2.503449	0.000203	T
ENSG0000(	C1orf52	2.6163	5.626	2.108015	0.000203	T
ENSG0000(	NDUFAF7	1.2507	3.5512	2.703191	0.000204	T
ENSG0000(	DNAJC13	5.7497	13.5529	2.333949	0.000204	T
ENSG0000(	MUCL3	13.4921	1.8133	0.140766	0.000204	N
ENSG0000(	KLF13	6.9378	15.3718	2.198386	0.000205	T
ENSG0000(	EFCAB7	0.594	1.7194	2.621614	0.000206	T
ENSG0000(	BAMBI	0.1576	2.724	10.96273	0.000206	T
ENSG0000(	DKC1	5.5287	18.0666	3.227495	0.000206	T
ENSG0000(	CRYBG1	92.562	35.1733	0.380666	0.000207	N
ENSG0000(	RAN	24.7904	54.337	2.187068	0.000207	T
ENSG0000(	ATP1A1	30.2676	93.9528	3.097143	0.000207	T
ENSG0000(	TMEM223	1.746	4.1531	2.303954	0.000207	T
ENSG0000(	TKFC	1.1617	3.0146	2.468574	0.000208	T
ENSG0000(	TRIM27	1.9647	5.0902	2.513779	0.000208	T
ENSG0000(	SPR	2.7751	9.4621	3.325832	0.000208	T
ENSG0000(	GORAB	1.0458	3.2076	2.886717	0.000208	T
ENSG0000(	PRPF40B	0.6438	1.5195	2.177333	0.000208	T
ENSG0000(	TJP3	18.7444	4.6641	0.252813	0.000209	N
ENSG0000(	DDX39A	2.6608	9.9801	3.651152	0.000209	T
ENSG0000(	NUPL2	1.8124	4.3736	2.33926	0.000209	T
ENSG0000(	DNAJC10	1.5772	3.9206	2.39721	0.000209	T
ENSG0000(	MRPL47	12.675	40.7071	3.194294	0.000209	T
ENSG0000(	MFN1	7.7613	17.5393	2.243815	0.00021	T
ENSG0000(	PCDH7	3.043	6.612	2.135539	0.00021	T
ENSG0000(	HOXC13-A	0.0414	0.9479	7.410891	0.000211	T
ENSG0000(	AC026401	2.7965	7.7697	2.716969	0.000211	T
ENSG0000(	NOM1	1.7505	4.4992	2.485382	0.000212	T

ENSG0000(	CAMK2N1	30.6184	2.0286	0.069294	0.000212	N
ENSG0000(	RN7SKP71	228.9175	639.9858	2.794921	0.000212	T
ENSG0000(	PKMYT1	0.5505	4.2579	6.699308	0.000212	T
ENSG0000(	GYS2	0.9714	0.0801	0.168098	0.000212	N
ENSG0000(	AP000866.	3.1643	0.1721	0.083356	0.000213	N
ENSG0000(	UCK2	2.5257	5.1739	2.008569	0.000213	T
ENSG0000(	RAD51B	0.6154	1.7555	2.593654	0.000213	T
ENSG0000(	UPRT	4.48	2.0326	0.465633	0.000213	N
ENSG0000(	TMEM70	2.5249	7.5473	2.913368	0.000213	T
ENSG0000(	RAB40AL	0.0606	0.2991	2.485056	0.000213	T
ENSG0000(	LSM10	3.0389	7.1722	2.316799	0.000213	T
ENSG0000(	ZFP69B	0.2864	0.7001	2.070652	0.000213	T
ENSG0000(	CHL1	1.3001	4.7131	3.437683	0.000214	T
ENSG0000(	HSP90AB1	122.9667	296.0173	2.406153	0.000215	T
ENSG0000(	TMEM179	8.2445	16.9774	2.046546	0.000215	T
ENSG0000(	TOR3A	2.2703	6.7639	2.895794	0.000215	T
ENSG0000(	SNORA77	0.1934	1.1967	4.419564	0.000216	T
ENSG0000(	PRPF31	5.2379	11.3323	2.141722	0.000216	T
ENSG0000(	SLC25A48	0.0575	0.3681	2.972063	0.000216	T
ENSG0000(	EXOSC9	1.6571	3.7789	2.207558	0.000217	T
ENSG0000(	AP005212.	2.2931	0.1777	0.116042	0.000218	N
ENSG0000(	YARS2	2.4981	5.674	2.222393	0.000218	T
ENSG0000(	YARS	4.5651	10.3785	2.246147	0.000218	T
ENSG0000(	AC012370	0.3803	1.8371	4.033104	0.000218	T
ENSG0000(	THOC6	3.1425	10.2841	3.202498	0.000219	T
ENSG0000(	MAGOHB	1.0509	2.8193	2.536537	0.000219	T
ENSG0000(	SLC12A9	1.08	3.0325	2.654661	0.00022	T
ENSG0000(	BTG3-AS1	0.1611	0.4941	2.275373	0.00022	T
ENSG0000(	TMEM198	0.1493	0.5833	2.740874	0.00022	T
ENSG0000(	TTF2	1.2573	4.0297	3.042585	0.00022	T
ENSG0000(	TPPP	2.7021	7.7146	2.788837	0.000221	T
ENSG0000(	WDR36	4.485	10.7757	2.372017	0.000221	T
ENSG0000(	PPP4R1L	0.4193	1.3894	2.868092	0.000221	T
ENSG0000(	DOCK9	26.9705	12.7541	0.474838	0.000221	N
ENSG0000(	RAD51D	0.3056	0.8081	2.238905	0.000221	T
ENSG0000(	LGALS8	4.5079	9.6622	2.118579	0.000222	T
ENSG0000(	ENOX2	2.078	5.1272	2.4	0.000222	T
ENSG0000(	PICK1	1.9215	3.9499	2.003413	0.000222	T
ENSG0000(	FAM72D	0.1639	0.8563	3.623721	0.000222	T
ENSG0000(	TMEM132	2.2147	9.662	4.217393	0.000223	T
ENSG0000(	TCOF1	1.3396	4.2072	2.991942	0.000223	T
ENSG0000(	EBNA1BP	4.5107	12.0379	2.63255	0.000224	T
ENSG0000(	SEM1	2.1938	4.9401	2.197271	0.000224	T
ENSG0000(	C5orf15	9.1769	22.7902	2.467441	0.000224	T
ENSG0000(	UHMK1	9.2428	20.9432	2.252344	0.000224	T
ENSG0000(	VEGFA	4.2881	11.0001	2.529591	0.000224	T
ENSG0000(	TRMT10C	6.6959	18.8779	2.792551	0.000224	T
ENSG0000(	SLC37A3	0.9322	2.5023	2.52112	0.000225	T
ENSG0000(	MBD4	3.4397	11.6393	3.316467	0.000225	T
ENSG0000(	WNK4	4.7992	0.7687	0.177315	0.000225	N
ENSG0000(	SDF4	7.4283	18.3822	2.45503	0.000226	T
ENSG0000(	PRMT3	1.7319	4.8998	2.729297	0.000226	T
ENSG0000(	HEXA	0.7301	2.3118	2.905433	0.000226	T
ENSG0000(	MPHOSPH	1.0594	3.258	2.896326	0.000227	T
ENSG0000(	AL356801.	0.4169	1.4494	2.997485	0.000227	T
ENSG0000(	RNF130	1.1663	2.852	2.331201	0.000228	T
ENSG0000(	AL031123.	0.0708	0.6462	4.368852	0.000228	T
ENSG0000(	CDKN3	1.5021	8.7471	5.52219	0.000228	T

ENSG0000(	E2F7	0.6341	2.8791	4.058166	0.000228	T
ENSG0000(	KLHL2	13.9652	6.925	0.49946	0.000228	N
ENSG0000(	RBM4	1.4488	4.9609	3.267627	0.000228	T
ENSG0000(	NSUN2	5.2055	14.0561	2.668193	0.000228	T
ENSG0000(	PARN	4.4699	10.8202	2.389593	0.000228	T
ENSG0000(	AL133338.	0.2692	0.6922	2.14572	0.000229	T
ENSG0000(	AC073529	0.0853	0.4258	2.837561	0.000229	T
ENSG0000(	ATRN	4.7084	11.1296	2.335413	0.000229	T
ENSG0000(	BHLHB9	0.6291	1.483	2.17117	0.00023	T
ENSG0000(	AL139095.	0.0082	0.8883	9.134011	0.00023	T
ENSG0000(	UTP25	1.1563	3.1557	2.591499	0.00023	T
ENSG0000(	FABP5P2	7.1242	25.4588	3.537942	0.000231	T
ENSG0000(	SSRP1	6.6605	20.2396	3.008594	0.000233	T
ENSG0000(	FBXO43	0.0558	0.2325	2.134146	0.000233	T
ENSG0000(	ETV5	0.7221	2.3354	2.962413	0.000234	T
ENSG0000(	RUVBL2	6.7661	15.5469	2.278863	0.000234	T
ENSG0000(	RAB29	1.7099	4.1152	2.328968	0.000235	T
ENSG0000(	HOMER3	0.649	2.8504	3.939119	0.000235	T
ENSG0000(	FAAP20	0.8246	2.038	2.312351	0.000236	T
ENSG0000(	CCDC142	0.5269	1.422	2.427819	0.000236	T
ENSG0000(	LMBR1	2.7095	5.8052	2.101869	0.000236	T
ENSG0000(	FOXM1	1.8948	12.8926	6.513234	0.000237	T
ENSG0000(	RN7SKP23	99.016	276.408	2.789741	0.000237	T
ENSG0000(	MYBBP1A	3.3949	9.6951	2.802684	0.000237	T
ENSG0000(	AL160408.	0.1577	0.644	2.887078	0.000237	T
ENSG0000(	RFC5	1.9776	6.5509	3.201242	0.000237	T
ENSG0000(	CALB2	6.7846	0.8443	0.137161	0.000237	N
ENSG0000(	TRIM45	0.232	0.7425	2.537651	0.000237	T
ENSG0000(	IL34	9.259	2.4421	0.271621	0.000237	N
ENSG0000(	TMPRSS1	428.9527	102.6826	0.239557	0.000238	N
ENSG0000(	RAVER1	1.4772	3.7121	2.417005	0.000238	T
ENSG0000(	AC106799	0	2.1109	22.109	0.000238	T
ENSG0000(	PPT1	5.6794	21.3351	3.70888	0.000238	T
ENSG0000(	SLC30A7	3.3218	8.8441	2.613858	0.000239	T
ENSG0000(	ZWINT	2.077	8.3468	3.880018	0.000239	T
ENSG0000(	AC005840	0.2173	1.167	3.993066	0.00024	T
ENSG0000(	MRPS26	6.0979	15.4025	2.50125	0.00024	T
ENSG0000(	MAT2A	23.5483	58.4608	2.476322	0.00024	T
ENSG0000(	SKIV2L	2.7078	6.0621	2.194636	0.00024	T
ENSG0000(	CCS	2.0766	4.6702	2.191583	0.00024	T
ENSG0000(	GALNT10	0.8011	2.1181	2.461547	0.00024	T
ENSG0000(	AC073195	0.2417	1.0379	3.330114	0.000241	T
ENSG0000(	GEMIN2	0.7883	3.1726	3.684116	0.000242	T
ENSG0000(	RNF227	1.1327	2.9913	2.507747	0.000242	T
ENSG0000(	PPP1R3C	82.6981	8.999	0.109894	0.000243	N
ENSG0000(	ACTR5	1.4938	3.932	2.529803	0.000243	T
ENSG0000(	MAFB	17.6597	35.4302	2.000608	0.000243	T
ENSG0000(	NUP62	3.0186	8.2268	2.670044	0.000244	T
ENSG0000(	EIPR1-IT1	0.2254	0.9858	3.336816	0.000244	T
ENSG0000(	CLCN6	0.8663	2.3314	2.516196	0.000244	T
ENSG0000(	AC068491	0.1218	0.709	3.64743	0.000244	T
ENSG0000(	TMPO	5.7343	18.8006	3.239566	0.000245	T
ENSG0000(	SLFNL1-A	0.1895	0.9179	3.516062	0.000245	T
ENSG0000(	GAPLINC	0.0453	0.3652	3.201652	0.000245	T
ENSG0000(	SMARCA5	6.1218	13.1982	2.137356	0.000245	T
ENSG0000(	AL031281.	0.2973	0.8489	2.388372	0.000245	T
ENSG0000(	SENP3	1.3776	3.593	2.499323	0.000246	T
ENSG0000(	EEF1A1P3	0.2918	0.8353	2.387187	0.000246	T

ENSG0000(	PTGFRN	10.6957	31.0411	2.884584	0.000246	T
ENSG0000(	LSM6	1.0396	2.2677	2.077659	0.000246	T
ENSG0000(	CEBPD	12.8483	52.6971	4.077531	0.000247	T
ENSG0000(	CERK	6.2993	12.7912	2.01447	0.000247	T
ENSG0000(	SLC26A6	0.3475	1.2404	2.995307	0.000247	T
ENSG0000(	TTC30A	0.5771	1.6952	2.651307	0.000248	T
ENSG0000(	RPL39L	0.9618	10.3339	9.826615	0.000248	T
ENSG0000(	NCL	24.3544	58.9039	2.412813	0.000248	T
ENSG0000(	ACOT7	2.5689	6.81	2.589082	0.000248	T
ENSG0000(	AC092902	0.2866	1.3444	3.736161	0.000249	T
ENSG0000(	TBC1D7	0.7571	1.7466	2.154474	0.000249	T
ENSG0000(	RN7SL144	0.0605	0.653	4.691589	0.000249	T
ENSG0000(	ENDOU	57.7484	13.2942	0.23154	0.00025	N
ENSG0000(	ZNF415	0.7657	0.1746	0.3172	0.00025	N
ENSG0000(	PRMT1	6.0259	16.2349	2.666531	0.00025	T
ENSG0000(	BEND3	0.4627	1.6712	3.147681	0.00025	T
ENSG0000(	C10orf91	0.1786	0.6685	2.758435	0.000251	T
ENSG0000(	POLR3H	2.1361	4.427	2.024507	0.000251	T
ENSG0000(	SULT2B1	86.0045	28.9625	0.337526	0.000252	N
ENSG0000(	ANAPC10	0.4721	1.1066	2.109072	0.000252	T
ENSG0000(	RN7SKP2(	241.727	697.1878	2.883416	0.000252	T
ENSG0000(	PAN2	1.6127	4.051	2.423659	0.000253	T
ENSG0000(	RFC4	1.0235	7.8858	7.107966	0.000253	T
ENSG0000(	HIST1H2B	11.0175	40.1395	3.619474	0.000253	T
ENSG0000(	FAM161A	0.3662	1.005	2.370227	0.000254	T
ENSG0000(	LINC0248'	42.128	2.6418	0.064928	0.000254	N
ENSG0000(	AC093895	0	2.538	26.38	0.000254	T
ENSG0000(	LINC0141(	0.1685	0.7802	3.278212	0.000255	T
ENSG0000(	WDR35	0.6541	1.9184	2.676568	0.000255	T
ENSG0000(	AL355802.	0.9386	2.2271	2.240612	0.000255	T
ENSG0000(	ADTRP	0.2863	1.1027	3.113383	0.000255	T
ENSG0000(	PFDN4	3.4973	9.5689	2.687821	0.000256	T
ENSG0000(	LRR1	1.6176	4.4217	2.632569	0.000257	T
ENSG0000(	ZFYVE1	9.9196	3.5839	0.367669	0.000257	N
ENSG0000(	DHRS13	1.2688	4.3305	3.236777	0.000257	T
ENSG0000(	GON7	4.4139	11.4962	2.568998	0.000257	T
ENSG0000(	TRIP13	0.9291	7.8101	7.686425	0.000258	T
ENSG0000(	CRYBG2	31.5713	10.3748	0.330735	0.000258	N
ENSG0000(	AL162390.	0.1939	0.7631	2.936713	0.000259	T
ENSG0000(	SPRED2	5.6601	12.0719	2.11314	0.000259	T
ENSG0000(	DNAJC2	1.6226	5.6399	3.332114	0.000259	T
ENSG0000(	POLR2G	6.6292	16.8567	2.519869	0.000259	T
ENSG0000(	CRYL1	6.1992	2.8527	0.468742	0.00026	N
ENSG0000(	SMC1A	4.3526	8.8159	2.002403	0.00026	T
ENSG0000(	DNASE2	4.8656	11.647	2.365676	0.000262	T
ENSG0000(	GEMIN4	2.2379	5.5478	2.415758	0.000262	T
ENSG0000(	FAM151B	0.4358	0.9791	2.013998	0.000262	T
ENSG0000(	PTPRK	3.5208	14.5531	4.046923	0.000263	T
ENSG0000(	ABCC5	2.7313	26.4317	9.370854	0.000264	T
ENSG0000(	ATP2A1	0.0438	0.2656	2.54242	0.000265	T
ENSG0000(	AC009948	0.2083	0.8034	2.930263	0.000266	T
ENSG0000(	NPM1P24	0.2194	0.9969	3.434252	0.000266	T
ENSG0000(	SRSF2	22.7628	51.7593	2.268283	0.000267	T
ENSG0000(	AC122718	0.6402	4.4378	6.130505	0.000267	T
ENSG0000(	GTPBP8	2.1239	5.1331	2.353118	0.000267	T
ENSG0000(	NEU3	0.5621	1.6192	2.596587	0.000268	T
ENSG0000(	CALU	10.591	26.2431	2.464045	0.000268	T
ENSG0000(	TIMMDC1	12.5695	26.6177	2.10882	0.000269	T

ENSG0000	PMS2P1	2.5651	7.264	2.763123	0.000269	T
ENSG0000	PARP8	1.6582	3.898	2.273917	0.000269	T
ENSG0000	ZNF18	1.2435	2.6252	2.028433	0.00027	T
ENSG0000	LRRC8D	1.7941	10.9591	5.83871	0.00027	T
ENSG0000	TFCP2L1	3.6614	9.0258	2.426171	0.000271	T
ENSG0000	ZNF473	0.7199	2.1887	2.791438	0.000271	T
ENSG0000	AC132008	3.9791	8.7642	2.173077	0.000272	T
ENSG0000	MAPK10	0.1337	0.99	4.664099	0.000273	T
ENSG0000	RNF32	0.0927	0.3177	2.167618	0.000273	T
ENSG0000	SNRNP70	8.3619	20.6866	2.456493	0.000273	T
ENSG0000	PLEKHA1	2.1804	4.5126	2.022715	0.000273	T
ENSG0000	FAM86JP	0.3556	1.0613	2.548946	0.000274	T
ENSG0000	SDR39U1	2.3231	4.904	2.065123	0.000274	T
ENSG0000	RF00019	1.6816	6.4602	3.682196	0.000274	T
ENSG0000	DDX56	4.4521	9.2885	2.062455	0.000274	T
ENSG0000	OSTM1	3.255	6.6888	2.023487	0.000274	T
ENSG0000	AC124248	0.2385	1.0036	3.260266	0.000275	T
ENSG0000	KIF20A	1.0904	7.6874	6.541835	0.000275	T
ENSG0000	L3MBTL2	1.8009	3.9112	2.110158	0.000275	T
ENSG0000	ARMC8	2.8477	6.3833	2.199444	0.000276	T
ENSG0000	ANAPC4	1.8473	3.9755	2.092898	0.000276	T
ENSG0000	DNAJC9	2.973	7.0109	2.313993	0.000277	T
ENSG0000	LSM7	8.2976	18.5271	2.218146	0.000277	T
ENSG0000	ZNF354A	1.0767	2.7548	2.426107	0.000277	T
ENSG0000	C1orf74	1.5806	4.4406	2.701773	0.000278	T
ENSG0000	SENP1	2.8461	6.3561	2.191406	0.000278	T
ENSG0000	AC139792	0.1383	0.5612	2.774654	0.000278	T
ENSG0000	NDUFB9	7.1138	19.9458	2.778813	0.000278	T
ENSG0000	UPK3B	24.824	3.6312	0.149703	0.000279	N
ENSG0000	PFKM	1.9552	4.4674	2.222363	0.000279	T
ENSG0000	E2F1	1.0583	5.4409	4.783648	0.000279	T
ENSG0000	SENP2	3.5334	8.6011	2.394754	0.00028	T
ENSG0000	SLC5A6	1.752	5.5163	3.032559	0.00028	T
ENSG0000	B3GNT9	1.7114	4.2933	2.425362	0.00028	T
ENSG0000	GPT2	24.6267	5.6704	0.233367	0.00028	N
ENSG0000	MTND5P2	0.1224	0.7465	3.806205	0.00028	T
ENSG0000	GCNA	0.2083	0.7154	2.644826	0.000281	T
ENSG0000	SVIP	4.2788	1.9086	0.45871	0.000281	N
ENSG0000	ZNF132	0.6243	0.1308	0.318652	0.000281	N
ENSG0000	MAP3K10	0.8637	1.955	2.132406	0.000281	T
ENSG0000	NOP56	5.0097	16.8766	3.322426	0.000282	T
ENSG0000	SBK1	0.2755	0.862	2.561917	0.000282	T
ENSG0000	CHTF18	0.5243	2.9255	4.846228	0.000283	T
ENSG0000	TM2D3	0.923	2.1256	2.175562	0.000283	T
ENSG0000	RRP7BP	0.6323	2.2975	3.273931	0.000284	T
ENSG0000	SLC1A5	13.966	61.4577	4.376347	0.000284	T
ENSG0000	KRT14	77.9617	914.4085	11.7152	0.000284	T
ENSG0000	TUBAP2	0.2002	0.8313	3.102265	0.000285	T
ENSG0000	RTL6	2.6714	5.9572	2.18561	0.000285	T
ENSG0000	IMP4	4.488	10.3139	2.269813	0.000285	T
ENSG0000	PSMD2	24.2072	55.6705	2.294402	0.000286	T
ENSG0000	MAN2A2	1.3849	2.9981	2.086403	0.000286	T
ENSG0000	GLB1L	0.2476	0.9996	3.163406	0.000286	T
ENSG0000	AC011498	0.096	0.4859	2.989286	0.000286	T
ENSG0000	TSPAN6	32.2542	13.4429	0.418582	0.000286	N
ENSG0000	ENTPD7	4.2694	9.4961	2.196205	0.000286	T
ENSG0000	ARL2	3.2717	7.5309	2.26322	0.000287	T
ENSG0000	RPL35P3	0.1307	0.8744	4.223667	0.000287	T

ENSG0000(	PABPC4	7.6548	16.3872	2.126064	0.000288	T
ENSG0000(	ZNF281	1.7476	5.082	2.80472	0.000289	T
ENSG0000(	BBS4	0.8916	2.049	2.167205	0.000289	T
ENSG0000(	SEPHS2	8.0899	18.8853	2.318136	0.000289	T
ENSG0000(	AC099066	0.0027	0.3148	4.038948	0.00029	T
ENSG0000(	PLOD1	2.4369	8.4098	3.354409	0.00029	T
ENSG0000(	PSCA	124.5343	7.946	0.064557	0.00029	N
ENSG0000(	PNO1	3.125	11.3583	3.552961	0.000291	T
ENSG0000(	TCAF2	2.2594	0.9201	0.432356	0.000291	N
ENSG0000(	ACRBP	0.1049	0.3776	2.330893	0.000292	T
ENSG0000(	CDKN2AI	22.1342	7.2433	0.33027	0.000293	N
ENSG0000(	HCN2	3.5882	0.494	0.161054	0.000293	N
ENSG0000(	C12orf73	0.6533	1.6371	2.305987	0.000294	T
ENSG0000(	CCP110	1.1697	2.9829	2.428054	0.000294	T
ENSG0000(	TCP1	18.8876	41.2628	2.178411	0.000294	T
ENSG0000(	MPV17	2.704	5.9923	2.172718	0.000294	T
ENSG0000(	CBLB	1.2255	4.4241	3.413127	0.000294	T
ENSG0000(	FGFR1OP	0.2358	0.6089	2.111078	0.000295	T
ENSG0000(	GNB1L	0.1574	0.4973	2.320513	0.000295	T
ENSG0000(	MIR1244-3	1.7832	8.5179	4.5762	0.000297	T
ENSG0000(	AC074117	0.7235	1.7168	2.206193	0.000298	T
ENSG0000(	SLCO1A2	0	0.7555	8.555	0.000298	T
ENSG0000(	PALB2	2.9574	6.0827	2.022208	0.000301	T
ENSG0000(	DDB1	6.4299	15.8624	2.444509	0.000301	T
ENSG0000(	L3HYPDH	0.8021	3.0601	3.503048	0.000301	T
ENSG0000(	LINC0253:	0.0222	0.1978	2.436989	0.000301	T
ENSG0000(	HMGB1P1	0	0.2045	3.045	0.000302	T
ENSG0000(	TRMT112I	0.1737	0.9077	3.681768	0.000305	T
ENSG0000(	AIP	7.219	16.4016	2.254625	0.000305	T
ENSG0000(	PSMG1	4.5801	10.2445	2.210316	0.000305	T
ENSG0000(	SETD1A	2.3956	5.6207	2.292314	0.000306	T
ENSG0000(	ZNF717	1.1395	0.4908	0.476644	0.000307	N
ENSG0000(	RNY1P9	0.0908	1.0703	6.133648	0.000308	T
ENSG0000(	LMNB2	4.8315	15.6576	3.195296	0.000308	T
ENSG0000(	ACVR1	3.1269	10.2755	3.215315	0.000309	T
ENSG0000(	ARHGEF1	9.5619	4.4241	0.468241	0.000309	N
ENSG0000(	NAP1L2	0.6682	1.8578	2.548555	0.000309	T
ENSG0000(	PYCR3	0.8782	3.8057	3.992742	0.00031	T
ENSG0000(	PDIA5	0.7092	4.4155	5.580203	0.000311	T
ENSG0000(	GANAB	19.6198	55.411	2.814988	0.000311	T
ENSG0000(	ILF2	28.1742	67.9835	2.407973	0.000312	T
ENSG0000(	ESF1	3.6608	9.6953	2.604579	0.000312	T
ENSG0000(	CEP83	0.7523	2.8542	3.46615	0.000312	T
ENSG0000(	SNN	3.7157	8.5926	2.278114	0.000312	T
ENSG0000(	ATP13A3	6.5457	26.7651	4.042479	0.000312	T
ENSG0000(	BSG	30.6528	71.4315	2.326016	0.000313	T
ENSG0000(	C11orf24	2.1131	6.4504	2.95983	0.000314	T
ENSG0000(	SGK1	7.6496	55.9684	7.235006	0.000314	T
ENSG0000(	CHCHD10	12.0117	29.136	2.413864	0.000315	T
ENSG0000(	HOXC13	0.0717	1.5785	9.775772	0.000315	T
ENSG0000(	CDCA7	1.5942	6.1719	3.701983	0.000315	T
ENSG0000(	PLA2G6	0.9054	2.3144	2.401432	0.000315	T
ENSG0000(	IKBKG	0.6111	1.4444	2.171846	0.000316	T
ENSG0000(	AGRN	3.9322	13.2757	3.317221	0.000316	T
ENSG0000(	HAGLR	0.1095	0.8939	4.744153	0.000316	T
ENSG0000(	GALNS	0.9499	3.0969	3.044957	0.000317	T
ENSG0000(	FAIM	0.8207	2.1587	2.453242	0.000317	T
ENSG0000(	RRN3P1	0.343	1.3561	3.286907	0.000317	T

ENSG0000(	AC009118	0.4596	2.5553	4.744996	0.000318	T
ENSG0000(	PDE7A	1.1847	3.5841	2.867673	0.000318	T
ENSG0000(	HIST1H4J	5.8687	19.5173	3.286696	0.000318	T
ENSG0000(	C19orf54	1.3465	3.3768	2.403595	0.000319	T
ENSG0000(	ZC3HAV1	2.002	4.8976	2.377545	0.000319	T
ENSG0000(	TAF5L	2.2729	4.8151	2.071347	0.00032	T
ENSG0000(	CDT1	1.326	6.0358	4.302805	0.00032	T
ENSG0000(	C1QBP	20.2785	48.8974	2.404367	0.00032	T
ENSG0000(	BCCIP	3.6954	8.6895	2.31583	0.00032	T
ENSG0000(	DLX2	0.0015	0.3765	4.694581	0.00032	T
ENSG0000(	AIFM1	3.1415	7.8839	2.463026	0.000321	T
ENSG0000(	SLC7A6	1.4546	4.9342	3.238261	0.000321	T
ENSG0000(	LRRC26	0.236	0.0413	0.420536	0.000321	N
ENSG0000(	RNU6-128	0.5479	6.8527	10.73113	0.000322	T
ENSG0000(	NCAPD2	4.3979	14.2017	3.179639	0.000322	T
ENSG0000(	BMS1P11	0.6782	0.1731	0.350938	0.000323	N
ENSG0000(	AC007878	0.9261	3.4651	3.474418	0.000323	T
ENSG0000(	RAPGEF6	0.4634	1.1354	2.192758	0.000323	T
ENSG0000(	CASC4	5.9842	12.4666	2.065448	0.000324	T
ENSG0000(	CENPJ	0.8659	2.9338	3.140905	0.000324	T
ENSG0000(	KLF7	1.792	6.2952	3.380127	0.000324	T
ENSG0000(	POLR3F	1.9146	5.2891	2.675022	0.000324	T
ENSG0000(	YEATS2	3.1036	7.6214	2.410226	0.000325	T
ENSG0000(	AC011978	0.2835	0.9367	2.703259	0.000326	T
ENSG0000(	BCS1L	2.6526	5.7372	2.120613	0.000327	T
ENSG0000(	HIST1H4L	63.0142	179.389	2.843877	0.000327	T
ENSG0000(	CDC7	1.0442	3.8046	3.412515	0.000328	T
ENSG0000(	LINC0101(	0.0261	0.436	4.250595	0.000328	T
ENSG0000(	SH3PXD2I	2.4638	7.1062	2.81075	0.000329	T
ENSG0000(	UBE2O	2.3098	4.8538	2.055689	0.000329	T
ENSG0000(	PTGER4	2.4576	10.4863	4.139154	0.000329	T
ENSG0000(	FSCN1	13.9736	57.1253	4.066145	0.000329	T
ENSG0000(	ABCF1	6.7951	14.7453	2.153022	0.000329	T
ENSG0000(	CDC42EP4	5.5403	29.6604	5.276386	0.000329	T
ENSG0000(	MIR548O2	0.7958	5.0173	5.712547	0.00033	T
ENSG0000(	AL355312.	16.0487	3.92	0.248936	0.00033	N
ENSG0000(	OSGEPL1	0.6538	1.6852	2.368267	0.00033	T
ENSG0000(	TRIM56	7.8859	3.6977	0.475551	0.00033	N
ENSG0000(	FOSB	10.1727	63.9165	6.231711	0.000331	T
ENSG0000(	TRMT1	2.3794	5.5165	2.265266	0.000331	T
ENSG0000(	KRI1	1.9534	5.4427	2.699279	0.000332	T
ENSG0000(	LINC0088!	0.5567	2.0343	3.250038	0.000332	T
ENSG0000(	RHBG	1.0057	0.2081	0.278647	0.000333	N
ENSG0000(	C5orf22	4.2316	9.342	2.179795	0.000333	T
ENSG0000(	LAGE3	3.2478	10.309	3.109206	0.000334	T
ENSG0000(	UTP14A	2.5995	7.0347	2.642971	0.000334	T
ENSG0000(	SPIDR	5.5239	12.1943	2.186081	0.000335	T
ENSG0000(	SNRPA	9.2487	19.7027	2.11823	0.000335	T
ENSG0000(	USP6NL	17.0054	6.572	0.390052	0.000335	N
ENSG0000(	CDCA8	2.0972	7.686	3.543601	0.000335	T
ENSG0000(	CXorf56	3.0769	6.8219	2.178822	0.000336	T
ENSG0000(	ADORA2I	2.5372	10.7527	4.115236	0.000336	T
ENSG0000(	ALMS1	2.2613	5.3535	2.309533	0.000336	T
ENSG0000(	POMGNT1	1.9204	4.9621	2.505494	0.000336	T
ENSG0000(	MRPL15	17.8258	38.6385	2.161047	0.000337	T
ENSG0000(	GRK3	0.8819	2.4324	2.579081	0.000338	T
ENSG0000(	TANGO6	1.1804	2.5239	2.049281	0.000338	T
ENSG0000(	PADI1	72.5442	8.7619	0.12199	0.000338	N

ENSG0000(	PRPF19	16.3224	41.3452	2.523699	0.000338	T
ENSG0000(	HIST1H2A	41.5686	146.4871	3.517927	0.000339	T
ENSG0000(	HIST1H1D	45.9748	152.6145	3.314491	0.00034	T
ENSG0000(	SHTN1	2.3208	5.5974	2.353519	0.00034	T
ENSG0000(	TMEM173	2.8328	8.5701	2.956253	0.00034	T
ENSG0000(	AC100861	0.6605	1.7593	2.444839	0.000341	T
ENSG0000(	SPRR1A	9834.883	3774.307	0.383774	0.000341	N
ENSG0000(	AC112206	0.1006	3.0591	15.74826	0.000342	T
ENSG0000(	NT5C3B	4.4076	10.2633	2.299073	0.000343	T
ENSG0000(	MTND2P2	0.1593	1.0604	4.475125	0.000343	T
ENSG0000(	B4GALT1-	2.6139	0.8249	0.340801	0.000344	N
ENSG0000(	ERVMER3	0.6385	2.8467	3.990115	0.000344	T
ENSG0000(	HMGN1P8	0.9083	3.8732	3.940494	0.000344	T
ENSG0000(	GRB10	0.8218	2.6353	2.967346	0.000344	T
ENSG0000(	JMJD4	1.4663	3.5315	2.318521	0.000345	T
ENSG0000(	AC009533	0.5396	2.4076	3.920575	0.000345	T
ENSG0000(	DIABLO	0.179	0.5545	2.345878	0.000346	T
ENSG0000(	C15orf61	0.6169	1.4282	2.131678	0.000346	T
ENSG0000(	AC020916	1.1622	6.5397	5.260418	0.000346	T
ENSG0000(	PYCR1	1.7136	10.4321	5.807289	0.000347	T
ENSG0000(	NCAPD3	1.6867	4.2314	2.424246	0.000347	T
ENSG0000(	TPRG1-AS	0.1175	2.3605	11.31264	0.000348	T
ENSG0000(	PPP6R3	13.0519	31.5094	2.403409	0.00035	T
ENSG0000(	AP001160.	0.3081	1.8562	4.793433	0.00035	T
ENSG0000(	BEX3	16.6565	51.0162	3.05053	0.00035	T
ENSG0000(	NPRL3	1.6237	4.0319	2.397111	0.00035	T
ENSG0000(	TNRC6C-A	0.3054	0.8175	2.263197	0.00035	T
ENSG0000(	AC019197	0.0681	0.3018	2.390244	0.00035	T
ENSG0000(	KCTD13	0.8979	2.1447	2.249424	0.00035	T
ENSG0000(	AC009948	0.5297	1.8013	3.019374	0.00035	T
ENSG0000(	TSEN54	1.893	4.3416	2.2286	0.000352	T
ENSG0000(	RAB34	3.2004	9.324	2.855411	0.000353	T
ENSG0000(	RN7SL200	0.4194	1.394	2.876396	0.000353	T
ENSG0000(	TBILA	0.2852	2.6343	7.09839	0.000353	T
ENSG0000(	AC007560	0.1029	0.5114	3.013307	0.000353	T
ENSG0000(	NAGPA	0.6274	1.5818	2.31207	0.000354	T
ENSG0000(	UTP20	1.6661	4.758	2.750694	0.000354	T
ENSG0000(	MUTYH	0.7779	2.0129	2.406766	0.000354	T
ENSG0000(	TMEM40	49.3733	12.4765	0.254208	0.000354	N
ENSG0000(	RBM26	3.5201	7.5762	2.120439	0.000354	T
ENSG0000(	TYRO3P	0.1774	0.5505	2.344989	0.000354	T
ENSG0000(	ACVR1C	0.0669	0.8268	5.553026	0.000354	T
ENSG0000(	MAGED1	5.5392	13.4412	2.401263	0.000355	T
ENSG0000(	XRCC2	0.3704	2.9705	6.527423	0.000356	T
ENSG0000(	HNRNPA1	0.3148	0.9065	2.426471	0.000356	T
ENSG0000(	NDC80	0.8969	4.1466	4.259805	0.000357	T
ENSG0000(	NOMO2	2.3268	6.1286	2.56659	0.000358	T
ENSG0000(	HLTF	1.6135	10.8472	6.388795	0.000359	T
ENSG0000(	MINPP1	1.0249	4.0872	3.722286	0.000359	T
ENSG0000(	ZNF487	0.2052	0.6443	2.438729	0.00036	T
ENSG0000(	FUT1	1.0494	2.4167	2.189577	0.00036	T
ENSG0000(	HSP90AB3	0.7266	2.8555	3.57549	0.000361	T
ENSG0000(	DDRGK1	6.8238	14.1547	2.058797	0.000362	T
ENSG0000(	USB1	1.3335	4.7007	3.348936	0.000362	T
ENSG0000(	ZNF777	1.5563	3.9184	2.426131	0.000363	T
ENSG0000(	RNASET2	0.9931	2.5982	2.468393	0.000363	T
ENSG0000(	CDC6	0.619	4.7232	6.708206	0.000363	T
ENSG0000(	SLC12A2	1.5199	5.1054	3.213408	0.000363	T

ENSG0000(	SKOR1	0.0277	0.1774	2.172279	0.000365	T
ENSG0000(	MIR4435-2	0.3957	1.743	3.717975	0.000367	T
ENSG0000(	CTSH	3.7788	9.9759	2.597685	0.000367	T
ENSG0000(	CPSF6	5.2554	13.6858	2.574187	0.000368	T
ENSG0000(	AC020661	0.0818	0.5041	3.322882	0.000369	T
ENSG0000(	ATAD3A	2.63	6.9438	2.580147	0.000369	T
ENSG0000(	CDAN1	1.09	2.6076	2.275294	0.00037	T
ENSG0000(	FBXO21	3.1081	6.4889	2.053832	0.00037	T
ENSG0000(	DNAJA3	3.0842	6.9917	2.227153	0.000371	T
ENSG0000(	RIBC2	0.347	1.3212	3.179418	0.000371	T
ENSG0000(	AGO3	1.2241	2.7521	2.153991	0.000372	T
ENSG0000(	SPINT1	131.8052	54.5	0.413934	0.000372	N
ENSG0000(	MARK1	2.396	9.011	3.65024	0.000372	T
ENSG0000(	H2AFZ	18.9698	65.8651	3.45914	0.000372	T
ENSG0000(	TAGLN2P	0.6568	2.798	3.829281	0.000373	T
ENSG0000(	AURKA	1.4342	9.3049	6.130166	0.000374	T
ENSG0000(	PCGF6	1.0892	2.6926	2.348301	0.000374	T
ENSG0000(	LEKR1	0.087	0.325	2.272727	0.000375	T
ENSG0000(	FAM102A	41.0586	17.7894	0.434645	0.000375	N
ENSG0000(	WHRN	0.7866	1.6839	2.012069	0.000375	T
ENSG0000(	TBRG4	2.8295	7.3373	2.538761	0.000375	T
ENSG0000(	MRPL3	15.807	51.2637	3.229	0.000375	T
ENSG0000(	NPHP3	0.2193	0.6757	2.429377	0.000375	T
ENSG0000(	KHSRP	17.7512	37.552	2.109214	0.000376	T
ENSG0000(	AP001347.	1.2099	0.29	0.297733	0.000377	N
ENSG0000(	AC087481	0.4955	1.7143	3.046683	0.000377	T
ENSG0000(	RPS17P2	0.3032	0.7993	2.230407	0.000378	T
ENSG0000(	FAM173B	2.4086	5.3367	2.167225	0.000378	T
ENSG0000(	RAI1	2.8484	8.9388	3.065663	0.000378	T
ENSG0000(	NCAPH	1.2987	8.7506	6.327733	0.000379	T
ENSG0000(	KIF2C	1.0735	6.8649	5.935151	0.000379	T
ENSG0000(	TIMM9	5.3133	12.4527	2.318863	0.00038	T
ENSG0000(	NPIPB11	0.1107	0.3392	2.08448	0.00038	T
ENSG0000(	MTFR1	4.192	10.3094	2.425303	0.00038	T
ENSG0000(	GPS2	0.1635	0.5926	2.628463	0.000383	T
ENSG0000(	AK4	10.6981	5.1265	0.48402	0.000384	N
ENSG0000(	CBWD2	1.0032	2.689	2.5281	0.000384	T
ENSG0000(	NAA50	25.1382	61.6752	2.447686	0.000384	T
ENSG0000(	CNGA1	3.1991	0.5354	0.192598	0.000385	N
ENSG0000(	TRMU	0.7928	2.1648	2.536738	0.000386	T
ENSG0000(	TELO2	1.7869	4.8108	2.602576	0.000386	T
ENSG0000(	ZNF273	0.9807	2.0791	2.016378	0.000386	T
ENSG0000(	COPS8	3.4844	8.0961	2.286603	0.000386	T
ENSG0000(	MCRIP1	1.0166	2.382	2.222819	0.000386	T
ENSG0000(	NAB1	12.5776	38.7206	3.062141	0.000387	T
ENSG0000(	ZFAND1	4.943	11.019	2.204838	0.000387	T
ENSG0000(	PIGK	1.9997	4.7405	2.305329	0.000387	T
ENSG0000(	IFI16	13.5513	55.5518	4.076667	0.000388	T
ENSG0000(	RBM25	5.1767	11.3118	2.162677	0.000389	T
ENSG0000(	TTC31	1.4554	3.1883	2.114119	0.000389	T
ENSG0000(	TAS2R14	0.1732	0.8621	3.521596	0.00039	T
ENSG0000(	SUMO3	12.9841	27.2335	2.089062	0.00039	T
ENSG0000(	STARD7	15.1597	44.7015	2.935936	0.000391	T
ENSG0000(	METTL22	0.8093	1.8778	2.17508	0.000391	T
ENSG0000(	CMC2	0.5629	1.4633	2.358274	0.000392	T
ENSG0000(	ZNF787	2.8422	6.3731	2.200088	0.000392	T
ENSG0000(	NASP	2.0175	5.956	2.859976	0.000394	T
ENSG0000(	IRF3	2.8301	7.267	2.514249	0.000394	T

ENSG0000(	SPERT	0.0135	0.2531	3.111013	0.000395	T
ENSG0000(	RNU4ATA	11.6498	62.6542	5.340874	0.000395	T
ENSG0000(	TCTN2	0.7788	1.8097	2.173077	0.000396	T
ENSG0000(	ZNF165	1.348	3.3049	2.35145	0.000397	T
ENSG0000(	NDUFV1	7.5307	17.9629	2.367135	0.000398	T
ENSG0000(	PNKD	1.178	3.3463	2.696635	0.000398	T
ENSG0000(	IL12A-AS1	2.2827	0.0622	0.068074	0.000399	N
ENSG0000(	SLC41A1	1.8396	5.4222	2.847082	0.0004	T
ENSG0000(	NUP37	2.3202	5.9891	2.515949	0.000401	T
ENSG0000(	THAP5	2.9525	6.1561	2.0495	0.000401	T
ENSG0000(	KCNMB2-	0.2044	4.9194	16.48949	0.000401	T
ENSG0000(	HIST2H2E	9.3424	35.0392	3.721427	0.000401	T
ENSG0000(	KPNA1	6.009	14.3141	2.359486	0.000401	T
ENSG0000(	AL353807.	0.1098	0.3979	2.373213	0.000402	T
ENSG0000(	AC068768	0.6241	1.3656	2.02403	0.000402	T
ENSG0000(	TAS2R19	0.2685	1.2072	3.547354	0.000403	T
ENSG0000(	DLEU1	0.1718	1.0361	4.179912	0.000403	T
ENSG0000(	PSMD6-AS	0.5029	1.2954	2.31448	0.000404	T
ENSG0000(	DDX28	2.1725	5.5875	2.50275	0.000405	T
ENSG0000(	DHX37	1.2948	3.6607	2.696229	0.000405	T
ENSG0000(	SNORA71	4.2023	13.1173	3.072147	0.000405	T
ENSG0000(	PRR5	0.8544	2.1194	2.32544	0.000405	T
ENSG0000(	SLC52A3	0.9757	4.1117	3.915311	0.000406	T
ENSG0000(	AC006460	0.0803	0.6404	4.106489	0.000406	T
ENSG0000(	TCERG1	3.7789	9.1469	2.383897	0.000408	T
ENSG0000(	GOPC	3.2988	7.3213	2.183506	0.000408	T
ENSG0000(	HSPA1A	18.2905	63.4616	3.456219	0.000408	T
ENSG0000(	SFI1	0.5529	1.3299	2.190075	0.000409	T
ENSG0000(	AC006978	0.1611	0.6896	3.024129	0.000409	T
ENSG0000(	FAM92A	0.3188	1.347	3.45511	0.000409	T
ENSG0000(	BCL2L12	1.4626	5.6606	3.686548	0.00041	T
ENSG0000(	TONSL	0.38	2.3623	5.129792	0.00041	T
ENSG0000(	NOP16	3.6528	7.5154	2.029258	0.000411	T
ENSG0000(	HIST1H2A	22.2113	85.2787	3.826702	0.000412	T
ENSG0000(	NUS1	7.9027	19.5372	2.453822	0.000412	T
ENSG0000(	PGBD5	0.2746	1.0756	3.138281	0.000413	T
ENSG0000(	ARSJ	1.181	3.364	2.704137	0.000413	T
ENSG0000(	SMG9	0.9975	2.5875	2.448747	0.000414	T
ENSG0000(	ELOA-AS1	0.4879	1.4379	2.615921	0.000414	T
ENSG0000(	SYCE3	0.0472	0.3375	2.972147	0.000416	T
ENSG0000(	AP005264.	0.0933	0.3066	2.103466	0.000416	T
ENSG0000(	ABHD5	13.5119	4.7503	0.356328	0.000417	N
ENSG0000(	C1orf131	0.854	2.5149	2.740985	0.000419	T
ENSG0000(	TJAP1	0.9964	2.6023	2.464703	0.00042	T
ENSG0000(	AC069439	0.1684	0.8984	3.719821	0.00042	T
ENSG0000(	POFUT2	0.7542	1.7642	2.182393	0.000421	T
ENSG0000(	U2AF1L4	0.4175	1.1125	2.342995	0.000421	T
ENSG0000(	PSMA1	3.451	7.3959	2.110926	0.000421	T
ENSG0000(	DUS3L	1.5835	4.0971	2.49308	0.000422	T
ENSG0000(	AC090181	0.2517	2.2771	6.758885	0.000423	T
ENSG0000(	ABHD10	2.3253	6.8042	2.846741	0.000423	T
ENSG0000(	RAD21	21.6652	44.0534	2.028624	0.000424	T
ENSG0000(	ZNF626	0.9065	0.3192	0.416493	0.000424	N
ENSG0000(	TMEM87E	7.4757	17.1211	2.273202	0.000424	T
ENSG0000(	MFSD13A	0.5475	1.8872	3.069035	0.000425	T
ENSG0000(	CDHR3	0.1085	0.3449	2.133813	0.000425	T
ENSG0000(	ATP6V1G	0.0625	0.6924	4.876308	0.000425	T
ENSG0000(	AC119150	0	0.6333	7.333	0.000426	T

ENSG0000(	LINC0204:	0.0534	1.2292	8.664928	0.000426	T
ENSG0000(	ANAPC1	1.9673	4.2854	2.121318	0.000426	T
ENSG0000(	POU6F2-A	0	3.0024	31.024	0.000427	T
ENSG0000(	SLC2A1	34.2012	94.4184	2.755542	0.000427	T
ENSG0000(	HYPK	0.1336	0.4229	2.238442	0.000428	T
ENSG0000(	PSMA3	9.4044	23.6119	2.494834	0.000428	T
ENSG0000(	CCSAP	1.3307	3.5562	2.555532	0.000429	T
ENSG0000(	CDH24	1.0675	3.918	3.441542	0.000429	T
ENSG0000(	CCDC87	0.0422	0.2185	2.239803	0.00043	T
ENSG0000(	KCNQ1	0.3829	1.4118	3.130669	0.00043	T
ENSG0000(	SLF1	0.8187	2.1464	2.445194	0.00043	T
ENSG0000(	PVT1	1.1367	4.4321	3.664672	0.00043	T
ENSG0000(	GLRX5	5.6685	12.3216	2.15335	0.000431	T
ENSG0000(	PSPC1	2.8295	6.4252	2.227411	0.000431	T
ENSG0000(	FOXD2	0.0124	0.2051	2.714413	0.000431	T
ENSG0000(	CEP89	0.8097	1.8006	2.08926	0.000432	T
ENSG0000(	URB2	1.3758	4.6161	3.195623	0.000432	T
ENSG0000(	LMNA	23.1358	49.9853	2.155523	0.000432	T
ENSG0000(	MOK	0.2695	1.148	3.377537	0.000432	T
ENSG0000(	GAPDHP6	0.0797	0.4736	3.191987	0.000434	T
ENSG0000(	TNS4	13.6199	58.5543	4.275126	0.000435	T
ENSG0000(	RN7SKP79	4.6799	13.3241	2.808448	0.000435	T
ENSG0000(	P3H4	0.5068	2.1481	3.704845	0.000436	T
ENSG0000(	KLHDC4	0.6992	1.8618	2.454705	0.000436	T
ENSG0000(	RN7SKP25	223.7009	611.3016	2.7319	0.000437	T
ENSG0000(	AL157827.	0.1345	0.5218	2.651599	0.000437	T
ENSG0000(	UBXN11	0.8716	2.3442	2.515644	0.000439	T
ENSG0000(	HSPH1	7.6	19.8434	2.590052	0.00044	T
ENSG0000(	ORC1	0.709	3.4325	4.366502	0.000441	T
ENSG0000(	ZNF593	7.765	18.7745	2.399809	0.000441	T
ENSG0000(	SORBS2	4.0592	0.3667	0.112209	0.000442	N
ENSG0000(	CCDC15	0.5715	1.5199	2.41236	0.000443	T
ENSG0000(	LANCL2	1.9159	6.6722	3.359393	0.000443	T
ENSG0000(	AL096870.	0.0468	0.2157	2.150545	0.000444	T
ENSG0000(	NPEPL1	0.5346	1.1756	2.010085	0.000445	T
ENSG0000(	APBB3	0.7571	2.0882	2.553028	0.000445	T
ENSG0000(	SAAL1	0.9753	2.7078	2.611178	0.000445	T
ENSG0000(	BANF1	14.0676	38.5977	2.731422	0.000445	T
ENSG0000(	ARHGAP1	11.5068	2.2587	0.203217	0.000445	N
ENSG0000(	EPB41L4B	0.3131	1.4009	3.633261	0.000447	T
ENSG0000(	C1orf109	1.434	3.2852	2.20678	0.000447	T
ENSG0000(	GGTA1P	2.9329	0.8905	0.326585	0.000448	N
ENSG0000(	LPCAT3	6.1195	13.2181	2.141346	0.00045	T
ENSG0000(	NADK	3.9019	8.3088	2.101202	0.00045	T
ENSG0000(	AL031775.	1.2828	3.0591	2.284568	0.000451	T
ENSG0000(	MNAT1	1.7664	4.6686	2.554972	0.000452	T
ENSG0000(	PHYHIP	2.7219	0.4174	0.183352	0.000452	N
ENSG0000(	CDK18	0.6548	2.2732	3.144144	0.000453	T
ENSG0000(	MRPL16	5.1366	10.5235	2.028702	0.000453	T
ENSG0000(	MAPK15	0.0929	0.4775	2.993779	0.000453	T
ENSG0000(	CACTIN	1.5152	3.1798	2.030584	0.000453	T
ENSG0000(	PRKAA2	3.0873	0.5985	0.219151	0.000454	N
ENSG0000(	SENP7	2.5592	5.7401	2.196187	0.000454	T
ENSG0000(	IRX3	5.2049	13.1989	2.506909	0.000455	T
ENSG0000(	NRCAM	0.0725	0.9088	5.848116	0.000455	T
ENSG0000(	MRPL2	3.7751	9.1151	2.378029	0.000455	T
ENSG0000(	CST6	46.675	8.8866	0.192124	0.000456	N
ENSG0000(	POLR3E	1.9831	4.2282	2.077769	0.000459	T

ENSG0000(	SLC3A2	9.1949	31.9547	3.448633	0.00046	T
ENSG0000(	KLK13	840.7754	180.096	0.214296	0.00046	N
ENSG0000(	BNIP3	13.9994	3.9615	0.288062	0.000461	N
ENSG0000(	SUPT3H	0.8863	2.2288	2.361148	0.000461	T
ENSG0000(	HSD11B2	0.8714	2.8647	3.051987	0.000461	T
ENSG0000(	UMAD1	1.2421	2.7051	2.090083	0.000461	T
ENSG0000(	TENM2	1.7916	7.3562	3.941742	0.000461	T
ENSG0000(	CHPF	5.6185	14.6353	2.576777	0.000463	T
ENSG0000(	CIB2	0.6547	2.2296	3.086789	0.000463	T
ENSG0000(	RUVBL1	1.3255	6.1867	4.410172	0.000464	T
ENSG0000(	APP	19.4016	62.4306	3.206434	0.000465	T
ENSG0000(	TMC6	1.93	4.2556	2.145616	0.000466	T
ENSG0000(	TMEM107	1.3755	4.0349	2.802372	0.000467	T
ENSG0000(	TBC1D8B	0.7148	1.7726	2.298233	0.000467	T
ENSG0000(	FEN1	2.4074	12.3975	4.984247	0.000467	T
ENSG0000(	CREB3L4	1.0976	2.6055	2.259102	0.000468	T
ENSG0000(	AC087632	0.0457	0.1938	2.016472	0.000468	T
ENSG0000(	GDPD2	0.1146	1.2285	6.190587	0.000468	T
ENSG0000(	SNRPD2	23.9262	55.4405	2.311664	0.000468	T
ENSG0000(	CLDND2	0.2536	0.6127	2.015554	0.000468	T
ENSG0000(	AC068831	0.0946	0.4466	2.808839	0.000468	T
ENSG0000(	RRP1B	3.3875	7.1319	2.073663	0.000469	T
ENSG0000(	CAMK2N2	0.0473	0.5584	4.46979	0.00047	T
ENSG0000(	HBP1	30.7277	13.4308	0.438917	0.00047	N
ENSG0000(	FSCN1P1	0.0258	0.1835	2.253577	0.00047	T
ENSG0000(	DHODH	0.3906	1.0532	2.350591	0.00047	T
ENSG0000(	RNU6-2	9.8005	33.9293	3.437129	0.00047	T
ENSG0000(	LY96	1.5823	17.7404	10.60477	0.000471	T
ENSG0000(	SASS6	0.9447	3.6931	3.630803	0.000472	T
ENSG0000(	ADAM12	0.1021	0.6404	3.663533	0.000472	T
ENSG0000(	AL359220.	0.1915	0.7883	3.047341	0.000473	T
ENSG0000(	CLBA1	0.6054	1.409	2.139212	0.000473	T
ENSG0000(	CEP295	1.4866	3.5511	2.30121	0.000473	T
ENSG0000(	NLN	0.9216	2.334	2.382537	0.000473	T
ENSG0000(	COQ3	2.0122	6.022	2.8984	0.000473	T
ENSG0000(	RBPMS	6.2224	2.5079	0.412486	0.000474	N
ENSG0000(	C4orf46	0.886	2.4226	2.558418	0.000474	T
ENSG0000(	VWA8	4.0117	8.2516	2.031179	0.000476	T
ENSG0000(	LINC0068(	0.502	1.4171	2.5201	0.000476	T
ENSG0000(	ERCC6L	0.3505	2.2947	5.315649	0.000477	T
ENSG0000(	TCF24	0	0.2684	3.684	0.000477	T
ENSG0000(	USP10	8.5526	18.8501	2.190105	0.000479	T
ENSG0000(	TAF1	3.1722	6.5046	2.018397	0.00048	T
ENSG0000(	SNORA80.	0.9896	4.0673	3.824615	0.00048	T
ENSG0000(	LRRC69	0.1846	0.8289	3.263879	0.00048	T
ENSG0000(	COA7	2.3218	5.7725	2.424849	0.00048	T
ENSG0000(	RNF7	5.7951	14.4798	2.473207	0.000481	T
ENSG0000(	LRP5L	0.3107	0.9974	2.672023	0.000481	T
ENSG0000(	EPPK1	1.9378	11.1773	5.534056	0.000481	T
ENSG0000(	TFAP4	0.6279	1.7359	2.522187	0.000481	T
ENSG0000(	DDX21	14.0491	36.2962	2.572333	0.000481	T
ENSG0000(	AC007738	0	0.657	7.57	0.000481	T
ENSG0000(	ZDHHC1	0.7161	2.3926	3.054283	0.000482	T
ENSG0000(	MANBA	0.8219	1.8865	2.154789	0.000482	T
ENSG0000(	STAG3L3	0.7639	1.9534	2.376895	0.000483	T
ENSG0000(	LGI3	5.3065	1.6499	0.323666	0.000484	N
ENSG0000(	THOC1	1.3637	3.3656	2.367698	0.000484	T
ENSG0000(	C1orf53	1.482	3.3919	2.207269	0.000486	T

ENSG0000(	JOSD1	5.4333	14.8867	2.708456	0.000486	T
ENSG0000(	AAAS	3.1656	6.5488	2.036012	0.000486	T
ENSG0000(	FAM118A	1.2349	2.8859	2.236797	0.000487	T
ENSG0000(	AL669818.	0.2185	0.7259	2.593093	0.00049	T
ENSG0000(	RUNX1	1.7201	7.3494	4.092852	0.00049	T
ENSG0000(	CYC1	21.3825	53.1108	2.476937	0.00049	T
ENSG0000(	ECM1	919.063	101.298	0.110316	0.000491	N
ENSG0000(	AC010148	0.0426	0.4794	4.063114	0.000492	T
ENSG0000(	ERO1B	0.7671	2.7963	3.340215	0.000493	T
ENSG0000(	CATSPER	0.2002	0.6386	2.46036	0.000494	T
ENSG0000(	COPS7B	1.6271	3.5491	2.112848	0.000494	T
ENSG0000(	SNORA12	37.9597	102.6523	2.699766	0.000495	T
ENSG0000(	GLI3	1.5125	3.2257	2.06245	0.000495	T
ENSG0000(	ELP5	2.5616	7.0601	2.690149	0.000495	T
ENSG0000(	WRAP53	0.4352	1.4618	2.918161	0.000496	T
ENSG0000(	HIST1H2B	16.5457	36.0422	2.171263	0.000497	T
ENSG0000(	RAD51-AS	0.8658	2.149	2.328639	0.000497	T
ENSG0000(	ARFGAP1	2.2766	5.6465	2.41795	0.000497	T
ENSG0000(	AC103703	0.1523	0.714	3.226318	0.000498	T
ENSG0000(	HNRNPRP	0.0456	0.3249	2.918269	0.000499	T
ENSG0000(	GCNT1P3	0.39	1.7986	3.874694	0.000499	T
ENSG0000(	FBXO34	40.2318	16.2373	0.405072	0.0005	N
ENSG0000(	SLIRP	6.1112	12.7118	2.062693	0.000501	T
ENSG0000(	KIRREL1	1.8445	4.2022	2.212497	0.000502	T
ENSG0000(	AC068491	0.2293	1.1353	3.751291	0.000503	T
ENSG0000(	AC018442	0.0431	0.2951	2.761006	0.000504	T
ENSG0000(	HIST1H3H	14.7541	45.2917	3.055836	0.000504	T
ENSG0000(	CKAP5	4.271	10.5915	2.446008	0.000505	T
ENSG0000(	DHX15	9.2472	20.4498	2.198498	0.000505	T
ENSG0000(	STK26	5.9231	12.8424	2.148794	0.000506	T
ENSG0000(	DNTTIP1	3.7389	10.3419	2.720024	0.000506	T
ENSG0000(	ANKEF1	1.6301	4.9352	2.910352	0.000508	T
ENSG0000(	PISD	3.4014	7.8606	2.273548	0.00051	T
ENSG0000(	SLC4A2	2.5869	5.2872	2.004987	0.000511	T
ENSG0000(	UTP23	2.0378	5.0507	2.409346	0.000511	T
ENSG0000(	NSUN4	1.326	2.9164	2.115288	0.000512	T
ENSG0000(	PTPRH	0.1638	3.694	14.38211	0.000512	T
ENSG0000(	HEXB	4.2498	11.2872	2.617867	0.000512	T
ENSG0000(	DNMT1	2.1912	7.7705	3.4351	0.000512	T
ENSG0000(	TIMM10	8.8975	21.9166	2.446969	0.000512	T
ENSG0000(	IKBIP	0.807	3.3145	3.764609	0.000513	T
ENSG0000(	AC022483	0.1051	0.4402	2.633837	0.000514	T
ENSG0000(	HMGA2	0.0226	0.9534	8.59217	0.000514	T
ENSG0000(	HIST1H3I	67.5397	209.598	3.100221	0.000514	T
ENSG0000(	HFE	0.5969	1.7061	2.59162	0.000515	T
ENSG0000(	SAFB	4.4155	9.6677	2.163149	0.000516	T
ENSG0000(	FAN1	1.7923	4.4319	2.394916	0.000517	T
ENSG0000(	OVCH1-A	0.3782	0.952	2.199916	0.000517	T
ENSG0000(	SPPL2B	1.1178	2.6399	2.249877	0.000517	T
ENSG0000(	TCF3	3.0913	8.5777	2.719174	0.00052	T
ENSG0000(	IL31RA	0.012	0.1891	2.58125	0.00052	T
ENSG0000(	MAP3K9	14.8551	6.5737	0.446249	0.000521	N
ENSG0000(	KRT8P36	0.0182	0.3052	3.428088	0.000522	T
ENSG0000(	AF117829.	0.2807	1.0034	2.898345	0.000522	T
ENSG0000(	TNFRSF18	0.5994	4.7672	6.959108	0.000522	T
ENSG0000(	AC125257	2.788	5.7274	2.017798	0.000523	T
ENSG0000(	SCARNA2	2.1357	7.4779	3.389498	0.000523	T
ENSG0000(	VSIG10	8.4709	2.1677	0.264581	0.000523	N

ENSG0000(	DDX55	1.2299	3.2401	2.511542	0.000524	T
ENSG0000(	MINDY1	13.3288	6.2447	0.47247	0.000524	N
ENSG0000(	RN7SL760	0.536	1.9273	3.187579	0.000524	T
ENSG0000(	SCRN1	5.9307	12.8079	2.140365	0.000524	T
ENSG0000(	OTX1	0.3502	1.2545	3.008663	0.000525	T
ENSG0000(	HCFC1	7.5953	17.0243	2.225293	0.000528	T
ENSG0000(	SSX2IP	1.2473	3.3248	2.541973	0.000528	T
ENSG0000(	AC026271	2.1622	4.6043	2.079524	0.000528	T
ENSG0000(	AL355472.	1.1835	4.6702	3.716556	0.000529	T
ENSG0000(	MFSD4B	1.2576	3.1859	2.420374	0.000529	T
ENSG0000(	ATRIP	0.7164	1.5554	2.027683	0.00053	T
ENSG0000(	SRP54-AS	0.3458	0.9977	2.462315	0.00053	T
ENSG0000(	APOO	1.4769	3.7243	2.425201	0.000531	T
ENSG0000(	RPA1	5.0748	14.8459	2.888208	0.000531	T
ENSG0000(	RNU7-45P	2.7568	11.6483	4.112398	0.000531	T
ENSG0000(	ENAH	2.6495	10.3048	3.784252	0.000532	T
ENSG0000(	FANCA	0.4358	2.3787	4.626166	0.000533	T
ENSG0000(	VAC14	1.962	4.7508	2.352473	0.000533	T
ENSG0000(	RRN3	3.877	9.8017	2.489741	0.000534	T
ENSG0000(	TMEM216	1.7195	4.5469	2.553943	0.000534	T
ENSG0000(	TMEM241	1.1119	2.5265	2.167258	0.000535	T
ENSG0000(	FIBP	5.094	11.1039	2.157085	0.000536	T
ENSG0000(	NGDN	1.5864	3.9015	2.372806	0.000537	T
ENSG0000(	KLK11	92.2552	41.1351	0.446484	0.000537	N
ENSG0000(	SLC25A19	0.8619	2.5038	2.706934	0.000538	T
ENSG0000(	GFOD2	11.3805	4.122	0.367754	0.000539	N
ENSG0000(	AC108136	4.0536	1.4274	0.367729	0.00054	N
ENSG0000(	CHRNA5	0.4792	1.729	3.157804	0.000542	T
ENSG0000(	CERS2	5.0899	16.0649	3.114684	0.000543	T
ENSG0000(	RGS19	1.9693	4.6744	2.307254	0.000543	T
ENSG0000(	ALG5	3.2866	7.4205	2.220664	0.000544	T
ENSG0000(	BACH1-IT	1.484	3.3889	2.202588	0.000544	T
ENSG0000(	ZNF367	0.8332	3.6432	4.011144	0.000544	T
ENSG0000(	FANCE	1.8349	4.2864	2.266991	0.000544	T
ENSG0000(	PIGO	1.6088	3.7632	2.260768	0.000545	T
ENSG0000(	ATIC	3.9561	12.3215	3.062424	0.000546	T
ENSG0000(	PTPRZ1	2.8097	8.8173	3.06468	0.000546	T
ENSG0000(	SLC38A1	11.1481	23.7692	2.122065	0.000547	T
ENSG0000(	PPP1R14B	2.0505	4.3322	2.061009	0.000548	T
ENSG0000(	Z93241.1	0.3986	2.836	5.888488	0.000548	T
ENSG0000(	TRPM7	3.7202	8.3551	2.213261	0.000548	T
ENSG0000(	SLURP1	675.8151	134.8883	0.199712	0.000549	N
ENSG0000(	AC015802	0.2064	0.6095	2.315601	0.000549	T
ENSG0000(	UHRF1	1.1791	7.359	5.831444	0.00055	T
ENSG0000(	IGSF1	0.0409	0.8454	6.709723	0.000551	T
ENSG0000(	C15orf40	0.4741	1.0814	2.05783	0.000553	T
ENSG0000(	TFAM	3.4104	7.7626	2.239802	0.000553	T
ENSG0000(	TMX2	12.7403	29.1374	2.277003	0.000553	T
ENSG0000(	MSTO1	0.8393	1.9138	2.143937	0.000553	T
ENSG0000(	NAA38	10.042	23.4996	2.326918	0.000554	T
ENSG0000(	DAP	7.741	17.0081	2.181877	0.000555	T
ENSG0000(	CCT6A	17.6018	51.9935	2.942836	0.000555	T
ENSG0000(	AUTS2	1.3882	2.9418	2.043946	0.000555	T
ENSG0000(	AC092142	0.1519	1.2941	5.534339	0.000556	T
ENSG0000(	SYNGAP1	0.7456	1.7538	2.192289	0.000556	T
ENSG0000(	BCL6	6.8651	21.6543	3.123329	0.000557	T
ENSG0000(	NUAK1	0.49	2.0981	3.725593	0.000558	T
ENSG0000(	SHPRH	1.2888	2.7599	2.05926	0.000558	T

ENSG0000(	NUP93	1.337	3.6994	2.643981	0.000559	T
ENSG0000(	TMEM63A	1.831	4.0169	2.132004	0.000559	T
ENSG0000(	SNHG26	0.5908	2.6981	4.050521	0.000559	T
ENSG0000(	LPCAT1	1.1802	6.4944	5.15107	0.000561	T
ENSG0000(	TTYH3	1.8608	10.3205	5.314412	0.000561	T
ENSG0000(	ACYP1	0.9151	2.9355	2.990346	0.000564	T
ENSG0000(	EHHADH	2.3034	6.4362	2.719564	0.000564	T
ENSG0000(	FBXO39	0.1711	0.4526	2.038362	0.000565	T
ENSG0000(	AP002884.	0.1891	0.6028	2.430993	0.000565	T
ENSG0000(	ALG10B	0.5099	1.7968	3.110018	0.000565	T
ENSG0000(	CCT7	16.6417	35.6878	2.137644	0.000565	T
ENSG0000(	DEGS1	18.8533	42.7952	2.263205	0.000566	T
ENSG0000(	RPL7P32	0.1811	0.8365	3.331555	0.000566	T
ENSG0000(	AGAP2-AS	2.1558	4.5838	2.076337	0.000566	T
ENSG0000(	AC025171	0.2259	0.822	2.829089	0.000567	T
ENSG0000(	SIAH1	2.3142	4.9402	2.087731	0.00057	T
ENSG0000(	GLTP	331.0752	150.6725	0.455265	0.000571	N
ENSG0000(	INTS1	4.9611	10.0491	2.005315	0.000572	T
ENSG0000(	MFSD10	2.9621	6.6793	2.213938	0.000573	T
ENSG0000(	ZNF827	1.0372	3.0876	2.803025	0.000573	T
ENSG0000(	MPHOSPH	4.2223	14.6559	3.4139	0.000573	T
ENSG0000(	EPHB4	4.3462	9.0718	2.06284	0.000574	T
ENSG0000(	TMEM185	6.8687	18.5395	2.674746	0.000574	T
ENSG0000(	AC004223	0.1708	0.9162	3.752585	0.000574	T
ENSG0000(	RF00019	4.0517	20.1868	4.886384	0.000576	T
ENSG0000(	RN7SKP36	0.7321	2.1819	2.742339	0.000577	T
ENSG0000(	TBL2	1.5576	3.7381	2.315456	0.000578	T
ENSG0000(	PHB	8.7351	17.8538	2.032099	0.000578	T
ENSG0000(	CRIP2	12.5606	5.6201	0.451803	0.00058	N
ENSG0000(	SP4	0.8905	2.4587	2.583241	0.00058	T
ENSG0000(	ATF5	2.0019	5.8118	2.812598	0.000581	T
ENSG0000(	NETO2	1.5839	4.6593	2.826355	0.000582	T
ENSG0000(	VARS	5.9962	12.1595	2.011007	0.000582	T
ENSG0000(	FAM133B	1.808	4.7533	2.543658	0.000584	T
ENSG0000(	SCX	0.2505	1.4033	4.289016	0.000584	T
ENSG0000(	ZFP69	0.467	1.0428	2.01552	0.000585	T
ENSG0000(	PIF1	0.1471	0.7342	3.375961	0.000586	T
ENSG0000(	C12orf54	0.6523	4.0886	5.567726	0.000586	T
ENSG0000(	NSMCE4A	3.6642	7.7652	2.089475	0.000586	T
ENSG0000(	AL133215.	0.0803	0.6767	4.30782	0.000587	T
ENSG0000(	SLC35B2	3.02	9.0248	2.924615	0.000589	T
ENSG0000(	UTP15	1.7183	3.7585	2.122037	0.000589	T
ENSG0000(	SNHG20	0.3433	0.9066	2.270697	0.000592	T
ENSG0000(	TIMELESS	1.5184	6.8176	4.274345	0.000593	T
ENSG0000(	ZNF669	1.0532	2.4579	2.218089	0.000593	T
ENSG0000(	LINC00336	1.8596	0.2099	0.158145	0.000594	N
ENSG0000(	AC007336	0.0848	0.563	3.587662	0.000594	T
ENSG0000(	DUS4L	0.6943	1.7296	2.303412	0.000594	T
ENSG0000(	ATP5F1CP	0.1604	0.5792	2.608295	0.000595	T
ENSG0000(	RPTOR	1.9205	4.0029	2.030636	0.000595	T
ENSG0000(	C1orf35	1.0672	2.3276	2.079849	0.000596	T
ENSG0000(	BCAM	3.5582	15.959	4.389864	0.000596	T
ENSG0000(	PTMAP2	3.6086	9.2559	2.522758	0.000597	T
ENSG0000(	TAF6L	1.5266	4.0199	2.532829	0.000598	T
ENSG0000(	PIM1	138.1151	47.6293	0.345326	0.000598	N
ENSG0000(	TAZ	1.2974	2.8788	2.131673	0.000602	T
ENSG0000(	ZFP64	1.2862	4.7686	3.512192	0.000603	T
ENSG0000(	HSPA8P8	0.1763	0.5188	2.239595	0.000603	T

ENSG0000(	RPS20P10	2.8426	8.3148	2.859648	0.000603	T
ENSG0000(	MIR4766	0.9586	5.6916	5.470999	0.000603	T
ENSG0000(	ABCC10	1.4063	3.045	2.087897	0.000605	T
ENSG0000(	RPL7L1	6.2348	12.7054	2.021437	0.000608	T
ENSG0000(	HIST1H4K	23.1167	87.2094	3.760629	0.000608	T
ENSG0000(	MTERF1	1.2742	3.029	2.276961	0.000609	T
ENSG0000(	CAMTA1-	0.3304	1.2903	3.230251	0.00061	T
ENSG0000(	SNHG10	0.7343	3.6396	4.482321	0.00061	T
ENSG0000(	EIF3B	7.4876	16.0736	2.131583	0.000611	T
ENSG0000(	ZBTB11-A	1.012	2.81	2.616906	0.000611	T
ENSG0000(	AC112206	0.0303	2.1381	17.17652	0.000611	T
ENSG0000(	AL590714.	0.399	1.1126	2.43006	0.000611	T
ENSG0000(	ADPGK	2.5944	7.1215	2.680189	0.000612	T
ENSG0000(	WDR90	0.5063	2.0474	3.541811	0.000616	T
ENSG0000(	KIZ	1.3895	5.6061	3.830883	0.000616	T
ENSG0000(	GRWD1	2.3313	5.5156	2.309711	0.000616	T
ENSG0000(	TBC1D19	0.9781	2.3163	2.241258	0.000617	T
ENSG0000(	NF1	2.0973	5.8563	2.710736	0.000617	T
ENSG0000(	AC012073	0.3878	1.5988	3.482575	0.000617	T
ENSG0000(	RF01210	0.2546	8.0264	22.91709	0.000617	T
ENSG0000(	UMPS	1.9375	5.6283	2.811436	0.000618	T
ENSG0000(	AC007431	0.0984	0.7969	4.520665	0.000619	T
ENSG0000(	EFL1P1	0.0358	0.3272	3.145803	0.00062	T
ENSG0000(	C1orf216	0.4578	1.1952	2.321979	0.000621	T
ENSG0000(	AC091271	0.2664	0.8887	2.698417	0.000621	T
ENSG0000(	ALG10	1.1122	3.6659	3.106666	0.000622	T
ENSG0000(	UGGT1	4.0853	10.9207	2.633192	0.000622	T
ENSG0000(	AL163051.	2.1971	5.9092	2.615994	0.000623	T
ENSG0000(	RIN2	4.7881	13.7976	2.84315	0.000623	T
ENSG0000(	DNAJB11	2.2269	10.0416	4.358417	0.000623	T
ENSG0000(	DGUOK	8.9642	19.1561	2.124413	0.000626	T
ENSG0000(	UTP18	4.8349	10.0006	2.046769	0.000628	T
ENSG0000(	FBXO41	0.3723	1.5315	3.454372	0.000628	T
ENSG0000(	SNRPEP4	0.7118	3.0969	3.938039	0.000628	T
ENSG0000(	ENTPD6	3.7829	8.0504	2.09905	0.000629	T
ENSG0000(	MAST2	1.7785	4.0577	2.213308	0.000633	T
ENSG0000(	OIP5	0.6283	4.2265	5.940546	0.000633	T
ENSG0000(	MUS81	1.639	4.0918	2.410466	0.000633	T
ENSG0000(	IBSP	0	0.2614	3.614	0.000634	T
ENSG0000(	PAICS	5.2583	12.0499	2.267492	0.000634	T
ENSG0000(	TTC30B	0.957	2.3331	2.301892	0.000635	T
ENSG0000(	AC021086	0.0697	0.2555	2.094873	0.000636	T
ENSG0000(	DCLRE1A	1.3765	3.5564	2.476397	0.000636	T
ENSG0000(	COL16A1	0.9762	2.9197	2.805891	0.000637	T
ENSG0000(	ZDHHC23	0.8659	2.1242	2.302723	0.000637	T
ENSG0000(	GPAA1	9.4183	20.3445	2.147915	0.000637	T
ENSG0000(	NPM1P27	1.8406	4.8241	2.537411	0.000638	T
ENSG0000(	ZNF765	0.5884	1.348	2.103428	0.00064	T
ENSG0000(	LAPTM4B	8.084	45.8988	5.620577	0.000641	T
ENSG0000(	HOXD10	0.0747	5.1389	29.98798	0.000642	T
ENSG0000(	MEN1	3.5132	7.9163	2.218615	0.000642	T
ENSG0000(	AUNIP	0.3706	2.7656	6.089248	0.000646	T
ENSG0000(	SERPINE2	2.1159	8.9484	4.083397	0.000646	T
ENSG0000(	LRGUK	0.1208	0.6347	3.327446	0.000646	T
ENSG0000(	CLNS1A	9.2365	22.0593	2.373405	0.000646	T
ENSG0000(	HIVEP3	1.885	3.936	2.033249	0.000647	T
ENSG0000(	STK31	0.1095	0.5884	3.285919	0.000647	T
ENSG0000(	MALL	111.0985	27.1159	0.244751	0.000647	N

ENSG0000(	SFXN1	3.1155	6.7833	2.140662	0.000649	T
ENSG0000(	AC010168	0.5109	1.9198	3.306269	0.000649	T
ENSG0000(	FAM171B	0.5325	2.7595	4.520949	0.00065	T
ENSG0000(	KLHL7	1.6002	3.3592	2.034584	0.00065	T
ENSG0000(	PARK7	7.5962	16.5863	2.168122	0.000651	T
ENSG0000(	RN7SL431	0.6049	2.3295	3.446588	0.000652	T
ENSG0000(	EXOSC2	1.7922	3.9619	2.146655	0.000654	T
ENSG0000(	CCDC86	2.5607	8.079	3.074003	0.000655	T
ENSG0000(	SPATS2	1.1579	3.5403	2.89395	0.000655	T
ENSG0000(	AGBL2	0.0822	0.2811	2.091658	0.000656	T
ENSG0000(	UQCC2	2.6087	5.9279	2.225385	0.000659	T
ENSG0000(	NUP133	4.4146	11.1241	2.486178	0.000661	T
ENSG0000(	AFAP1L1	0.7352	2.0941	2.627035	0.000662	T
ENSG0000(	TMEM177	0.7797	1.9339	2.312038	0.000662	T
ENSG0000(	SPC25	1.0031	6.3325	5.831294	0.000662	T
ENSG0000(	TEC	4.95	2.0216	0.420119	0.000663	N
ENSG0000(	MCM7	5.7012	24.3822	4.220196	0.000664	T
ENSG0000(	SLC25A28	9.897	21.5468	2.16533	0.000665	T
ENSG0000(	SNORD46	8.7278	27.0307	3.073325	0.000666	T
ENSG0000(	TBL1XR1	13.3507	40.0136	2.982269	0.000667	T
ENSG0000(	YIF1A	7.6171	15.4347	2.013023	0.000667	T
ENSG0000(	LINC0033(	0.272	0.7251	2.218011	0.000668	T
ENSG0000(	PSMC4	12.2146	27.0799	2.207128	0.000668	T
ENSG0000(	PAFAH1B	3.9749	8.1926	2.035044	0.000668	T
ENSG0000(	TDG	4.9549	10.188	2.035253	0.000669	T
ENSG0000(	NHLRC1	0.3353	1.352	3.335631	0.00067	T
ENSG0000(	IFT140	0.678	1.6167	2.206555	0.000671	T
ENSG0000(	HDAC8	0.217	0.6702	2.429653	0.000673	T
ENSG0000(	FAM229A	0.4142	1.4325	2.980358	0.000673	T
ENSG0000(	ERMARD	1.504	3.1148	2.004239	0.000674	T
ENSG0000(	MFSD12	2.3485	6.6039	2.737962	0.000676	T
ENSG0000(	FAT1	6.0763	25.4185	4.131681	0.000677	T
ENSG0000(	AC004943	0.2581	1.0255	3.142977	0.000678	T
ENSG0000(	POLA2	0.1876	0.8682	3.366481	0.000678	T
ENSG0000(	PET117	0.6784	1.64	2.235355	0.000679	T
ENSG0000(	NAA10	0.7618	1.7553	2.15282	0.000679	T
ENSG0000(	AP001029.	0	0.446	5.46	0.000679	T
ENSG0000(	TIGD3	0.0356	0.2692	2.722714	0.000679	T
ENSG0000(	SLBP	7.109	15.9629	2.228173	0.000679	T
ENSG0000(	AL121899.	2.8474	0.4305	0.179989	0.00068	N
ENSG0000(	NEPRO	1.953	6.6193	3.272918	0.000681	T
ENSG0000(	LINC0236'	0.0222	0.2939	3.223404	0.000681	T
ENSG0000(	AC016700	14.1361	35.5209	2.502153	0.000681	T
ENSG0000(	SPDL1	1.1204	3.9125	3.287856	0.000682	T
ENSG0000(	DHFRP1	0.053	0.3769	3.116993	0.000684	T
ENSG0000(	CRYBB2P	0.5866	1.427	2.224002	0.000687	T
ENSG0000(	TRIM59	0.7991	1.9772	2.31031	0.000687	T
ENSG0000(	LACTB2	1.9812	5.7634	2.817317	0.00069	T
ENSG0000(	XRCC3	0.465	1.9325	3.597345	0.00069	T
ENSG0000(	ZNF736	0.7488	1.8242	2.266965	0.000691	T
ENSG0000(	PTCD1	0.3284	0.8144	2.134454	0.000691	T
ENSG0000(	CSTF1	3.3127	7.9122	2.34776	0.000691	T
ENSG0000(	CHST14	1.1171	2.8518	2.425273	0.000691	T
ENSG0000(	NSUN5P2	0.7136	2.0874	2.688545	0.000691	T
ENSG0000(	ANXA3	11.5664	5.2782	0.460999	0.000691	N
ENSG0000(	PPIH	4.2905	11.0579	2.541373	0.000691	T
ENSG0000(	RNU6-5P	1.746	5.786	3.188516	0.000694	T
ENSG0000(	KDELC1	0.7688	2.2912	2.752302	0.000696	T

ENSG0000(	YBX1	101.3224	246.3803	2.430235	0.000696	T
ENSG0000(	IVNS1ABI	8.2494	24.7935	2.981472	0.000697	T
ENSG0000(	EFNA1	6.1813	14.6433	2.347173	0.000701	T
ENSG0000(	FAAP24	0.3507	1.2231	2.935656	0.000701	T
ENSG0000(	AL109618.	0.3147	0.8348	2.25416	0.000702	T
ENSG0000(	EDEM3	5.2217	10.9188	2.070541	0.000702	T
ENSG0000(	ABCE1	7.9211	17.0053	2.132538	0.000702	T
ENSG0000(	RAE1	2.5408	5.6845	2.190435	0.000704	T
ENSG0000(	TEPSIN	1.3451	2.9388	2.10283	0.000705	T
ENSG0000(	ZNF888	1.5681	4.1925	2.573287	0.000707	T
ENSG0000(	MAML3	5.8674	2.8236	0.489929	0.000707	N
ENSG0000(	AC114956	0.1833	2.7288	9.985175	0.000709	T
ENSG0000(	AL133243.	0.3776	1.7777	3.931533	0.00071	T
ENSG0000(	AC093724	0.0446	0.3037	2.79184	0.000711	T
ENSG0000(	AC099328	0.0439	0.2791	2.634468	0.000712	T
ENSG0000(	CROCC	0.9105	2.1433	2.21999	0.000713	T
ENSG0000(	NEMP2	0.4519	1.793	3.429969	0.000714	T
ENSG0000(	TNFRSF1(	2.709	8.6447	3.113101	0.000714	T
ENSG0000(	RRP12	1.3895	3.9464	2.716616	0.000714	T
ENSG0000(	FBXW8	1.4989	3.1001	2.001438	0.000714	T
ENSG0000(	VCPKMT	2.9241	6.8973	2.313845	0.000715	T
ENSG0000(	EXTL2	0.6252	1.8478	2.68588	0.000715	T
ENSG0000(	ZNF7	0.9616	2.385	2.340806	0.000716	T
ENSG0000(	HSPD1P6	0.0687	0.2423	2.029046	0.000717	T
ENSG0000(	AJUBA	4.0178	10.0132	2.455972	0.000718	T
ENSG0000(	MCM3AP-	0.2065	0.7581	2.799674	0.000718	T
ENSG0000(	ARMCX5-	0.5519	1.3441	2.215217	0.00072	T
ENSG0000(	SEMA6A-.	0.0794	0.2769	2.100892	0.00072	T
ENSG0000(	OTULIN	3.0903	6.7585	2.149798	0.00072	T
ENSG0000(	AL645608.	0.2648	1.2583	3.72341	0.000722	T
ENSG0000(	FOXQ1	6.4058	36.0766	5.560669	0.000724	T
ENSG0000(	AL451165.	1.1686	3.2614	2.649693	0.000725	T
ENSG0000(	TTL	1.5242	4.0241	2.539158	0.000726	T
ENSG0000(	HIST1H2E	43.323	142.1435	3.275764	0.000727	T
ENSG0000(	MIR1537	0.372	4.2165	9.145127	0.000728	T
ENSG0000(	ATF7IP2	0.6247	2.2967	3.307162	0.000728	T
ENSG0000(	ACAT2	5.1887	10.5234	2.008698	0.000728	T
ENSG0000(	CPD	5.5265	11.8478	2.123487	0.000729	T
ENSG0000(	RIOK3	65.1445	28.7217	0.441749	0.000729	N
ENSG0000(	VASN	27.7005	5.6752	0.207737	0.00073	N
ENSG0000(	PCED1A	2.7958	7.421	2.59721	0.000731	T
ENSG0000(	QPCTL	0.7843	2.0471	2.428022	0.000733	T
ENSG0000(	CDCA7L	1.8163	5.6936	3.023326	0.000734	T
ENSG0000(	MARS	5.0592	13.0334	2.545627	0.000735	T
ENSG0000(	PRSS23	0.5265	2.9761	4.909976	0.000735	T
ENSG0000(	PINK1	18.1998	7.9962	0.44242	0.000735	N
ENSG0000(	ADCY3	2.7327	9.2015	3.283616	0.000735	T
ENSG0000(	BLCAP	7.6848	16.1914	2.092719	0.000735	T
ENSG0000(	ALG1L	0.13	16.9035	73.92826	0.000735	T
ENSG0000(	OSMR-AS	0.3139	1.5367	3.954337	0.000736	T
ENSG0000(	AL512326.	0	3.264	33.64	0.000737	T
ENSG0000(	AC138393	0.4814	1.9311	3.493464	0.000737	T
ENSG0000(	STX16	5.9034	11.9138	2.001166	0.000739	T
ENSG0000(	EXOC6	1.1791	3.0062	2.428426	0.00074	T
ENSG0000(	JRKL	1.7775	4.6465	2.528096	0.000741	T
ENSG0000(	AC009948	0.5605	2.2656	3.581529	0.000742	T
ENSG0000(	ZNHIT2	2.247	6.6544	2.877887	0.000744	T
ENSG0000(	PEBP1	104.5776	51.8418	0.496207	0.000744	N

ENSG0000(	PPP3CA	6.385	14.9615	2.322513	0.000744	T
ENSG0000(	ATP23	0.8718	1.8846	2.04219	0.000745	T
ENSG0000(	TIGD1	0.8845	3.2841	3.437379	0.000745	T
ENSG0000(	RTN4R	0.5025	1.1125	2.012448	0.000748	T
ENSG0000(	OPTN	19.7227	8.4318	0.430406	0.00075	N
ENSG0000(	AL117350.	0.2686	1.2837	3.753934	0.000751	T
ENSG0000(	CASP1	3.5173	7.6671	2.147209	0.000752	T
ENSG0000(	AL162431.	0.1628	0.5198	2.358447	0.000755	T
ENSG0000(	METTL4	1.0333	2.3386	2.151769	0.000756	T
ENSG0000(	SLC22A1	0.1213	0.4667	2.560777	0.000756	T
ENSG0000(	SPACA6	0.2358	0.7432	2.511018	0.000756	T
ENSG0000(	ZNF135	0.2864	0.0745	0.451605	0.000757	N
ENSG0000(	TBC1D8-A	0.0759	0.3116	2.339966	0.000758	T
ENSG0000(	OSGIN2	1.463	3.5729	2.349904	0.00076	T
ENSG0000(	CD59	45.1157	20.7116	0.460274	0.000761	N
ENSG0000(	INSYN1	4.0742	0.9416	0.249533	0.000761	N
ENSG0000(	CCDC18-A	0.6306	1.8091	2.613058	0.000761	T
ENSG0000(	AC016394	0.7746	1.8953	2.281386	0.000763	T
ENSG0000(	FBRSL1	2.4232	5.9893	2.413324	0.000763	T
ENSG0000(	HMGB3P9	0.1956	0.5523	2.206698	0.000764	T
ENSG0000(	DNAJC30	2.4912	5.4042	2.12419	0.000766	T
ENSG0000(	PITPNA-A	3.5645	8.5181	2.351781	0.000767	T
ENSG0000(	AC090970	1.0054	3.7903	3.51936	0.000768	T
ENSG0000(	AL133243.	0.1686	0.9108	3.763217	0.000769	T
ENSG0000(	AC027644	2.2546	6.0266	2.601971	0.000771	T
ENSG0000(	EXOC3-AS	0.2968	1.0984	3.020161	0.000772	T
ENSG0000(	RPN1	28.0668	78.8776	2.803925	0.000772	T
ENSG0000(	ATXN2L	9.1411	19.632	2.135244	0.000772	T
ENSG0000(	FRRS1	5.6293	11.6282	2.047056	0.000773	T
ENSG0000(	SKA1	0.9309	4.4522	4.415753	0.000773	T
ENSG0000(	CRACR2A	0.1295	0.5227	2.71329	0.000773	T
ENSG0000(	GLS	2.96	8.5678	2.832614	0.000775	T
ENSG0000(	COL9A2	0.2375	0.8572	2.836148	0.000775	T
ENSG0000(	ZNF865	1.7897	3.7625	2.043975	0.000776	T
ENSG0000(	AC027796	0.4968	2.6625	4.628854	0.000776	T
ENSG0000(	PDCD2L	0.6057	1.849	2.761797	0.000776	T
ENSG0000(	MYEOV	14.0568	5.9757	0.429172	0.000777	N
ENSG0000(	HASPIN	0.5835	2.9655	4.485004	0.000777	T
ENSG0000(	OACYLP	0.0269	0.2798	2.992908	0.000777	T
ENSG0000(	LINC0234	0.0284	0.3416	3.439252	0.00078	T
ENSG0000(	HIST1H2B	5.4045	14.8568	2.717195	0.000786	T
ENSG0000(	AC005332	0.2767	0.7893	2.360765	0.000787	T
ENSG0000(	DDX11	0.4795	2.3221	4.179638	0.000788	T
ENSG0000(	CEP57	2.7642	6.0001	2.129774	0.000789	T
ENSG0000(	ABL2	0.9903	3.8801	3.650463	0.000789	T
ENSG0000(	EMG1	2.1753	4.9495	2.219268	0.00079	T
ENSG0000(	HPGD	39.201	5.8058	0.150271	0.000791	N
ENSG0000(	BFAR	3.0687	7.4908	2.395557	0.000791	T
ENSG0000(	AC106799	0.0022	0.6759	7.591977	0.000792	T
ENSG0000(	DMAP1	1.2996	2.9987	2.21399	0.000792	T
ENSG0000(	CTSD	21.9085	48.1436	2.192044	0.000792	T
ENSG0000(	TCFL5	1.6756	4.3286	2.494143	0.000793	T
ENSG0000(	LINC0110	0.4528	1.4678	2.836107	0.000793	T
ENSG0000(	ID2	4.8718	11.7257	2.378555	0.000795	T
ENSG0000(	RIMBP2	0.1363	0.4061	2.141769	0.000797	T
ENSG0000(	PMM2	1.8033	3.9081	2.105869	0.000798	T
ENSG0000(	Z83843.1	1.202	5.3726	4.203226	0.000799	T
ENSG0000(	ZC3H12C	1.2976	4.1604	3.048369	0.000801	T

ENSG0000(	RPL4P6	0.2749	0.7171	2.179515	0.000802	T
ENSG0000(	UQCRHP2	0.3761	1.0429	2.400546	0.000804	T
ENSG0000(	AC073130	0.0382	0.3665	3.375543	0.000804	T
ENSG0000(	SSBP4	2.7517	6.4774	2.306484	0.000805	T
ENSG0000(	EFNB1	10.056	46.7101	4.609108	0.000808	T
ENSG0000(	BBX	3.8165	8.0772	2.087885	0.000809	T
ENSG0000(	MRPL45P2	0.8598	1.8945	2.078037	0.000809	T
ENSG0000(	PNN	9.1137	20.4336	2.228594	0.00081	T
ENSG0000(	ATP6V0CP	0.7765	4.0521	4.737136	0.000811	T
ENSG0000(	EFTUD2	3.413	8.4359	2.429804	0.000811	T
ENSG0000(	PIGL	0.6903	1.6319	2.191446	0.000812	T
ENSG0000(	GAB1	9.8468	4.5541	0.467899	0.000812	N
ENSG0000(	ODF2	1.6449	3.8472	2.262135	0.000814	T
ENSG0000(	HIST2H3D	19.6335	90.6405	4.598297	0.000814	T
ENSG0000(	PLEKHM1	21.4991	8.9647	0.41968	0.000814	N
ENSG0000(	ZNF644	6.4159	13.2538	2.049418	0.000814	T
ENSG0000(	NXPH4	0.6572	4.125	5.579768	0.000818	T
ENSG0000(	NIP7	4.1111	9.3041	2.233169	0.00082	T
ENSG0000(	SOCS1	0.3976	4.9783	10.20559	0.000823	T
ENSG0000(	AC092910	0.2229	0.7982	2.781666	0.000823	T
ENSG0000(	LINC0100	0.6584	1.8929	2.627769	0.000824	T
ENSG0000(	TXNDC12	0.5767	1.3875	2.198168	0.000827	T
ENSG0000(	FOXRED1	0.9735	2.1458	2.092035	0.000827	T
ENSG0000(	FABP5P11	4.9514	21.2398	4.224532	0.00083	T
ENSG0000(	LSR	15.3886	38.1864	2.471908	0.000832	T
ENSG0000(	HMGN4	9.4864	20.6208	2.161479	0.000832	T
ENSG0000(	OSGEPL1-	0.0611	0.2979	2.469894	0.000832	T
ENSG0000(	CYBC1	2.0284	4.6233	2.219179	0.000836	T
ENSG0000(	UTP4	3.7037	9.971	2.647685	0.000838	T
ENSG0000(	RPS20P14	5.4701	16.0016	2.89072	0.000838	T
ENSG0000(	LZTR1	0.4765	1.1316	2.13634	0.000839	T
ENSG0000(	U2AF1	0.1721	0.492	2.175671	0.000841	T
ENSG0000(	AC092892	0.022	1.9899	17.13033	0.000841	T
ENSG0000(	AC005070	0.1077	0.5474	3.116996	0.000844	T
ENSG0000(	AC104118	0.145	0.5597	2.692653	0.000845	T
ENSG0000(	CCDC82	0.6459	2.2432	3.14144	0.000845	T
ENSG0000(	AL929236.	0.0992	0.5763	3.39508	0.000847	T
ENSG0000(	CGAS	0.7449	5.582	6.725056	0.00085	T
ENSG0000(	IMPAD1	9.1197	18.8375	2.054026	0.00085	T
ENSG0000(	GEMIN6	0.5214	1.1762	2.05375	0.000851	T
ENSG0000(	PHOSPHO	0.4802	1.1429	2.142192	0.000852	T
ENSG0000(	KIAA1586	1.4164	3.1105	2.117185	0.000852	T
ENSG0000(	AL390728.	3.2405	8.7167	2.639335	0.000853	T
ENSG0000(	RANBP9	124.8132	36.5266	0.293216	0.000854	N
ENSG0000(	GPRC5D	1.5223	0.4054	0.311533	0.000855	N
ENSG0000(	AGO2	5.2319	12.1764	2.302444	0.000856	T
ENSG0000(	HSD17B11	2.7707	9.5195	3.350925	0.000857	T
ENSG0000(	HNRNPLP	0.068	0.3422	2.632143	0.000858	T
ENSG0000(	MRPS34	13.3982	29.3723	2.183424	0.000859	T
ENSG0000(	OCIAD2	3.8571	13.1014	3.33613	0.000861	T
ENSG0000(	PELP1	2.4183	6.1454	2.480006	0.000862	T
ENSG0000(	DDX39B	3.1948	8.3587	2.567288	0.000863	T
ENSG0000(	NPL	2.744	9.3478	3.322011	0.000865	T
ENSG0000(	SNORA2A	2.1939	6.3453	2.809756	0.000865	T
ENSG0000(	MRPL28	3.4958	8.3033	2.336976	0.000865	T
ENSG0000(	SLC40A1	7.2098	18.0025	2.47647	0.000867	T
ENSG0000(	GPR52	0.1393	0.7704	3.637275	0.000869	T
ENSG0000(	TICAM1	51.229	17.2824	0.338647	0.000869	N

ENSG0000(	MRPL58	7.4686	16.3543	2.174022	0.000869	T
ENSG0000(	AIFM3	0.0626	0.2842	2.362854	0.00087	T
ENSG0000(	AC010280	0.2413	1.7677	5.472312	0.000871	T
ENSG0000(	GALNT2	5.5262	12.2611	2.19706	0.000872	T
ENSG0000(	KNOP1	1.1661	3.036	2.476898	0.000873	T
ENSG0000(	AC009120	0.7346	2.3722	2.962138	0.000874	T
ENSG0000(	FNDC3B	1.9909	11.1081	5.360419	0.000875	T
ENSG0000(	SNORA20	1.2528	4.9789	3.754361	0.000876	T
ENSG0000(	WDR24	1.4394	3.0118	2.021437	0.000876	T
ENSG0000(	AC016027	0.1036	0.3456	2.188605	0.000878	T
ENSG0000(	ACLY	7.2792	19.2515	2.622439	0.000878	T
ENSG0000(	PRKAB2	2.6625	5.5729	2.053538	0.000878	T
ENSG0000(	LINC0126!	27.3937	4.5244	0.168199	0.000882	N
ENSG0000(	MAP4K2	0.9003	2.7192	2.818354	0.000882	T
ENSG0000(	CSTA	3751.006	1542.768	0.41131	0.000882	N
ENSG0000(	FANCC	0.3338	0.8105	2.098893	0.000884	T
ENSG0000(	AC069499	0.3184	1.8487	4.657505	0.000885	T
ENSG0000(	SGK494	0.1918	0.7577	2.939342	0.000886	T
ENSG0000(	PTOV1-AS	1.3026	4.5252	3.29759	0.000887	T
ENSG0000(	NR2C1	1.1599	2.8887	2.372172	0.00089	T
ENSG0000(	ZNF678	0.5603	2.1377	3.388914	0.000894	T
ENSG0000(	ZDHHC4	2.3457	4.9198	2.0525	0.000894	T
ENSG0000(	UBE2S	8.5348	22.5379	2.621705	0.000894	T
ENSG0000(	PANX1	3.4108	13.4012	3.845619	0.000896	T
ENSG0000(	RPL7P7	0.2133	0.9803	3.448133	0.000897	T
ENSG0000(	MTHFD1L	0.6023	4.3001	6.265271	0.000898	T
ENSG0000(	NUP43	3.5175	8.7718	2.452467	0.000899	T
ENSG0000(	LMO7	41.5229	10.5358	0.255528	0.0009	N
ENSG0000(	LRRC23	0.4045	1.1036	2.385728	0.0009	T
ENSG0000(	TMEM44-.	0.8702	4.0902	4.318903	0.000901	T
ENSG0000(	FAM76B	1.4574	4.6293	3.036664	0.000902	T
ENSG0000(	AURKB	1.5109	10.7727	6.749457	0.000902	T
ENSG0000(	AL163051.	0.2121	0.6688	2.463313	0.000903	T
ENSG0000(	BTBD16	0.0854	0.5076	3.277238	0.000903	T
ENSG0000(	SMIM20	1.9719	5.0896	2.504754	0.000905	T
ENSG0000(	SINHCAF	5.4512	14.2853	2.591386	0.000906	T
ENSG0000(	AL049758.	0.1632	0.9255	3.896277	0.000906	T
ENSG0000(	AL021997.	0.2869	1.5457	4.253554	0.000906	T
ENSG0000(	AC092718	0.1679	0.5201	2.31467	0.000907	T
ENSG0000(	AC100860	0.0095	0.2092	2.823744	0.000909	T
ENSG0000(	RNU6-80P	0.0308	1.2796	10.5474	0.000912	T
ENSG0000(	TRMT61B	2.7431	8.6299	3.070557	0.000914	T
ENSG0000(	CYP4A22-	0.0908	0.6967	4.175577	0.000916	T
ENSG0000(	SLC39A4	0.8895	2.3721	2.498332	0.000917	T
ENSG0000(	FABP5P1	17.4344	58.8478	3.361837	0.000917	T
ENSG0000(	AC093157	0.0886	0.6601	4.030223	0.000918	T
ENSG0000(	HIST1H2E	0.0127	0.6094	6.294587	0.000918	T
ENSG0000(	RN7SL19F	0.2377	1.0002	3.257921	0.000919	T
ENSG0000(	RAB11FIP	1.1095	2.3623	2.0358	0.000921	T
ENSG0000(	RFXAP	0.5241	1.4947	2.555199	0.000922	T
ENSG0000(	VSIG4	0.8749	2.9669	3.145861	0.000924	T
ENSG0000(	Z97832.2	0.0693	0.2593	2.122268	0.000925	T
ENSG0000(	RF00019	0.9986	4.2693	3.977153	0.000928	T
ENSG0000(	RPS14P4	0.1125	1.0469	5.397176	0.000929	T
ENSG0000(	Z95331.1	0.0682	0.2462	2.058264	0.000929	T
ENSG0000(	TRPM2	0.3697	1.1432	2.646796	0.00093	T
ENSG0000(	NNT	4.0277	8.801	2.156407	0.00093	T
ENSG0000(	TK1	4.3257	27.9256	6.332467	0.00093	T

ENSG0000(	LSM4	6.7027	14.2914	2.115542	0.000932	T
ENSG0000(	AC023034	0.0724	2.7571	16.57251	0.000933	T
ENSG0000(	NNT-AS1	0.685	1.572	2.129936	0.000934	T
ENSG0000(	AC093155	0.1147	0.5678	3.110387	0.000934	T
ENSG0000(	WDR4	1.9669	4.7332	2.338381	0.000936	T
ENSG0000(	NAT9	0.8283	2.1476	2.4212	0.000937	T
ENSG0000(	PIGW	2.2204	5.3681	2.356533	0.000938	T
ENSG0000(	DPH1	1.2699	3.0324	2.28659	0.000939	T
ENSG0000(	EPS8L1	83.8118	12.6188	0.151573	0.00094	N
ENSG0000(	TPBG	3.3229	9.5873	2.830144	0.000941	T
ENSG0000(	ADAT1	1.9227	4.8672	2.455727	0.000942	T
ENSG0000(	LINC0197	0.2771	0.0587	0.420843	0.000946	N
ENSG0000(	RNF139-A	0.11	0.5473	3.082381	0.000947	T
ENSG0000(	NOLC1	8.7945	23.0806	2.606172	0.000947	T
ENSG0000(	PAX1	0.6296	0.0791	0.245477	0.000948	N
ENSG0000(	AC073869	1.4803	3.4295	2.233437	0.00095	T
ENSG0000(	ITGA6	14.014	57.4375	4.076626	0.00095	T
ENSG0000(	WDR66	0.0941	1.1166	6.267903	0.000951	T
ENSG0000(	SNAPC5	1.7962	3.7412	2.025736	0.000955	T
ENSG0000(	CDH13	1.1114	4.1519	3.509906	0.000955	T
ENSG0000(	RANP6	0.3792	1.1681	2.646285	0.000956	T
ENSG0000(	IGFL4	0.0178	0.2019	2.562818	0.000956	T
ENSG0000(	ARHGAP2	0.6313	1.9328	2.779707	0.000956	T
ENSG0000(	CCDC160	0.2299	0.0119	0.339194	0.000957	N
ENSG0000(	PLXNA1	3.2208	14.4554	4.3831	0.000957	T
ENSG0000(	RN7SL384	0.0771	0.4724	3.232072	0.000957	T
ENSG0000(	SEZ6L2	0.2628	3.1206	8.877067	0.000957	T
ENSG0000(	LYPD1	0.0748	0.3823	2.759153	0.000959	T
ENSG0000(	SF3B2	7.4041	15.868	2.127903	0.00096	T
ENSG0000(	EGR4	0.0247	0.4317	4.263833	0.00096	T
ENSG0000(	NARS2	2.008	5.3633	2.591698	0.000962	T
ENSG0000(	NOC2L	5.1228	10.478	2.02535	0.000963	T
ENSG0000(	KDELR3	0.8585	3.6943	3.958581	0.000963	T
ENSG0000(	HAUS1	2.5672	5.6294	2.148095	0.000966	T
ENSG0000(	ARHGEF1	2.4711	6.0869	2.406324	0.000968	T
ENSG0000(	RPL26L1	5.4077	11.1286	2.038709	0.000969	T
ENSG0000(	AC024581	0	3.449	35.49	0.000969	T
ENSG0000(	STEAP1B	0.057	0.793	5.687898	0.000969	T
ENSG0000(	BEX4	51.826	20.4496	0.395748	0.00097	N
ENSG0000(	WDR27	0.5988	1.85	2.790498	0.00097	T
ENSG0000(	THAP7	1.9342	4.2512	2.139023	0.000971	T
ENSG0000(	AL121580.	0	2.1725	22.725	0.000971	T
ENSG0000(	MCM2	1.7348	14.7601	8.09903	0.000971	T
ENSG0000(	CDC42SE2	4.094	10.2385	2.465069	0.000972	T
ENSG0000(	STK4	4.6316	9.4244	2.012934	0.000972	T
ENSG0000(	TMEM120	0.5491	1.2542	2.086273	0.000973	T
ENSG0000(	MAGEH1	1.3553	3.1425	2.228063	0.000974	T
ENSG0000(	SNHG1	3.535	13.2876	3.682971	0.000974	T
ENSG0000(	SPINK8	7.0703	0.7389	0.116996	0.000974	N
ENSG0000(	BRIX1	2.433	8.7606	3.498066	0.000976	T
ENSG0000(	AL133375.	0.065	0.6615	4.615152	0.000978	T
ENSG0000(	ASF1B	1.7352	8.6068	4.744333	0.000978	T
ENSG0000(	FAM86C2	0.5146	1.2543	2.203547	0.00098	T
ENSG0000(	GNGT1	0.1233	2.9585	13.69682	0.000981	T
ENSG0000(	AMPD2	1.2966	4.219	3.09251	0.000982	T
ENSG0000(	FOXO3B	0.6222	1.3493	2.006785	0.000982	T
ENSG0000(	FAM133D	0.1117	0.7881	4.195087	0.000983	T
ENSG0000(	CCL17	2.3924	0.8543	0.382884	0.000984	N

ENSG0000(	LAPTM4B	0.0431	0.3985	3.483578	0.000984	T
ENSG0000(	SLC41A3	1.6636	4.1382	2.403153	0.000985	T
ENSG0000(	USP31	2.5714	6.5258	2.480273	0.000985	T
ENSG0000(	CGNL1	14.805	2.0842	0.146541	0.000985	N
ENSG0000(	C11orf95	1.0171	2.5076	2.334258	0.000986	T
ENSG0000(	GNL2	4.0207	9.0908	2.230398	0.000986	T
ENSG0000(	AC133485	3.7931	0.3718	0.121189	0.000989	N
ENSG0000(	AC112236	3.9898	1.2604	0.332632	0.00099	N
ENSG0000(	TOPBP1	3.1427	14.0585	4.366269	0.000992	T
ENSG0000(	TXNDC15	2.3277	4.8644	2.044898	0.000992	T
ENSG0000(	HNRNPL	11.5365	26.3233	2.270726	0.000994	T
ENSG0000(	RPN2	33.7959	82.3205	2.431577	0.000994	T
ENSG0000(	NECAB3	1.0988	3.0517	2.629046	0.001001	T
ENSG0000(	DDAH1	8.5985	2.5199	0.30119	0.001001	N
ENSG0000(	SLC16A5	1.4877	4.11	2.651634	0.001001	T
ENSG0000(	PDCD11	3.6239	7.8904	2.145707	0.001002	T
ENSG0000(	TACO1	2.7499	5.9823	2.134215	0.001004	T
ENSG0000(	MKNK2	87.9606	41.3423	0.470611	0.001005	N
ENSG0000(	SNORA74	0.6238	1.7275	2.524869	0.001007	T
ENSG0000(	RN7SKP8(	2356.987	6205.172	2.632602	0.001008	T
ENSG0000(	TICRR	1.0239	3.77	3.443367	0.001008	T
ENSG0000(	ADGRL3	0.1226	1.0324	5.087152	0.001011	T
ENSG0000(	TSPAN10	0.3122	1.0043	2.679039	0.001012	T
ENSG0000(	M6PR	10.519	22.9319	2.168933	0.001014	T
ENSG0000(	SCARNA2	207.5567	594.8517	2.865073	0.001015	T
ENSG0000(	AL590560.	0.0414	0.2893	2.753182	0.001015	T
ENSG0000(	MRTFA	2.8287	5.9775	2.075153	0.001015	T
ENSG0000(	MCMDC2	0.1196	0.4157	2.348361	0.001016	T
ENSG0000(	ULBP2	2.8913	7.96	2.694481	0.001017	T
ENSG0000(	MROH6	12.0209	4.6343	0.39059	0.001019	N
ENSG0000(	RHOD	110.0554	45.5702	0.414598	0.00102	N
ENSG0000(	HIST1H2B	21.6927	96.7179	4.442676	0.001022	T
ENSG0000(	RPS17	1.5812	3.3877	2.07453	0.001027	T
ENSG0000(	KIAA1211	5.1404	1.6568	0.335242	0.001029	N
ENSG0000(	Z84485.1	0.1971	0.602	2.362841	0.00103	T
ENSG0000(	AL121832.	0.8655	2.3622	2.550181	0.00103	T
ENSG0000(	LINC0123.	0.2806	0.8877	2.595113	0.001032	T
ENSG0000(	AC106798	0.1922	3.2752	11.55099	0.001032	T
ENSG0000(	TUBGCP6	1.8261	4.2361	2.251233	0.001032	T
ENSG0000(	NUDT4	2.9694	6.0746	2.011664	0.001033	T
ENSG0000(	NEMP1	2.1831	7.5616	3.355788	0.001033	T
ENSG0000(	PAGR1	0.9884	2.6667	2.541988	0.001035	T
ENSG0000(	S100A9	20291.21	9442.092	0.465332	0.001039	N
ENSG0000(	SEMA4F	0.2514	0.869	2.757541	0.00104	T
ENSG0000(	CD44	36.9346	75.2521	2.034641	0.001041	T
ENSG0000(	TFDP1	14.1407	30.5583	2.152865	0.001041	T
ENSG0000(	CTU1	0.8316	2.1081	2.370223	0.001043	T
ENSG0000(	AC011731	0.1542	0.7185	3.219906	0.001047	T
ENSG0000(	PTMAP4	6.103	14.7181	2.38886	0.001048	T
ENSG0000(	MTR	2.2824	5.1963	2.223094	0.001049	T
ENSG0000(	HOXA10-/	0.1359	0.9215	4.330225	0.00105	T
ENSG0000(	AL139421.	1.624	5.2024	3.075638	0.001052	T
ENSG0000(	NPW	0.1728	3.4269	12.92852	0.001055	T
ENSG0000(	NDUFA4L	84.5248	22.717	0.269625	0.001057	N
ENSG0000(	BPNT1	2.5629	5.2991	2.027526	0.001058	T
ENSG0000(	AC016582	0.2295	0.0539	0.467071	0.001061	N
ENSG0000(	RPAP3	2.6315	5.6739	2.11382	0.001061	T
ENSG0000(	MLF1	1.3242	12.4836	8.835557	0.001062	T

ENSG0000(	RN7SL566	0.2712	1.0737	3.161907	0.001064	T
ENSG0000(	AC005840	0.2144	0.6755	2.466603	0.001067	T
ENSG0000(	KCNJ15	1.9303	5.8936	2.952076	0.001067	T
ENSG0000(	PUS1	0.7956	2.3323	2.715833	0.001075	T
ENSG0000(	PUDP	1.4866	3.1466	2.046262	0.001075	T
ENSG0000(	TOP3B	1.0902	2.4826	2.169887	0.001075	T
ENSG0000(	NRBP2	1.9747	4.3744	2.156649	0.001077	T
ENSG0000(	C5orf34	0.3554	1.5362	3.592885	0.001077	T
ENSG0000(	CCDC58	3.3599	13.456	3.918032	0.001078	T
ENSG0000(	TSR3	6.6863	13.8351	2.053416	0.001079	T
ENSG0000(	AGAP1-IT	0.3218	0.8823	2.328829	0.001079	T
ENSG0000(	ROBO1	4.4729	9.2131	2.036585	0.001082	T
ENSG0000(	KDM4D	0.1305	0.378	2.073753	0.001083	T
ENSG0000(	MIR4713H	0.6194	3.4769	4.97206	0.001085	T
ENSG0000(	ZNF597	0.4965	1.3835	2.487008	0.001085	T
ENSG0000(	WARS2	2.4311	5.0939	2.052033	0.001086	T
ENSG0000(	AC009948	0.0549	0.2323	2.145255	0.00109	T
ENSG0000(	RNU6-36P	5.1056	13.9476	2.698555	0.001094	T
ENSG0000(	CCDC150	0.0897	0.5321	3.332103	0.001094	T
ENSG0000(	EMC3	18.4855	8.5309	0.464389	0.001095	N
ENSG0000(	GPRC5C	0.4572	1.5309	2.926956	0.001096	T
ENSG0000(	HNRNPA3	0.3983	1.2519	2.713024	0.001099	T
ENSG0000(	AC002076	0	0.2527	3.527	0.001101	T
ENSG0000(	COX6CP1	1.1061	3.8722	3.293425	0.001101	T
ENSG0000(	CPNE1	7.4531	16.4011	2.184679	0.001103	T
ENSG0000(	LINC0205'	0.256	1.26	3.820225	0.001104	T
ENSG0000(	SNHG16	10.4103	21.109	2.017925	0.001105	T
ENSG0000(	STEAP1	5.16	18.5206	3.540038	0.001109	T
ENSG0000(	EARS2	1.6794	3.8218	2.204001	0.001109	T
ENSG0000(	LY6G5C	0.0906	0.3042	2.120672	0.00111	T
ENSG0000(	RN7SKP18	0.3221	2.1787	5.398484	0.001113	T
ENSG0000(	GCSAM	0.2953	0.7238	2.083987	0.001115	T
ENSG0000(	FAM86C1	1.1163	2.4959	2.13426	0.001115	T
ENSG0000(	CDKL2	0.5379	0.1762	0.432983	0.001115	N
ENSG0000(	AL360091.	0.5432	2.3026	3.735386	0.001122	T
ENSG0000(	PLCG1	2.2615	4.6524	2.01245	0.001125	T
ENSG0000(	LINC0064'	0.5021	1.2617	2.261584	0.001125	T
ENSG0000(	ADAT2	0.7015	1.7121	2.260886	0.001125	T
ENSG0000(	ACAD11	0.1476	0.5511	2.629645	0.001125	T
ENSG0000(	SLC27A3	0.8714	2.269	2.438748	0.001128	T
ENSG0000(	LINC0182(	1.3651	0.1161	0.147498	0.001131	N
ENSG0000(	BNC1	5.6223	20.0825	3.526991	0.001132	T
ENSG0000(	EXT1	3.3209	13.4373	3.957233	0.001133	T
ENSG0000(	SMYD4	0.8239	2.0919	2.372443	0.001134	T
ENSG0000(	METTL16	1.6169	3.7335	2.232803	0.001136	T
ENSG0000(	DEGS2	8.0618	3.6024	0.453625	0.001138	N
ENSG0000(	KTI12	0.48	1.2086	2.256207	0.001139	T
ENSG0000(	RAB43P1	0.1034	0.5036	2.967552	0.001139	T
ENSG0000(	XPO4	2.4603	5.1073	2.033863	0.001139	T
ENSG0000(	ZFHX4	0.1678	1.0058	4.129201	0.001141	T
ENSG0000(	AC087392	0.2009	0.965	3.539382	0.001143	T
ENSG0000(	MIR3143	0.4829	4.3049	7.556871	0.001143	T
ENSG0000(	CYP4F35P	1.3339	0.0756	0.122463	0.001144	N
ENSG0000(	CCNF	0.9269	3.4126	3.420586	0.001146	T
ENSG0000(	DEPDC4	0.0924	0.3245	2.206341	0.001147	T
ENSG0000(	RHOB	19.6517	56.0156	2.841052	0.001149	T
ENSG0000(	AL021068.	0.4094	1.0535	2.264429	0.00115	T
ENSG0000(	ENTR1	2.6303	6.7182	2.497235	0.00115	T

ENSG0000(	RBM19	2.2197	5.6396	2.474285	0.001153	T
ENSG0000(	RN7SKP9	164.4937	445.309	2.706112	0.001154	T
ENSG0000(	ZSCAN5A	0.2568	0.7819	2.471693	0.001154	T
ENSG0000(	RBM44	0.0969	0.697	4.04774	0.001155	T
ENSG0000(	MRPS6	2.2453	4.997	2.173283	0.001156	T
ENSG0000(	KLHDC8A	4.3858	0.4685	0.126733	0.001157	N
ENSG0000(	SCEL	482.8315	84.1543	0.174464	0.001164	N
ENSG0000(	PLD3	8.1892	17.6429	2.140484	0.001165	T
ENSG0000(	GAA	3.9737	14.7964	3.656725	0.001166	T
ENSG0000(	PRPF8	16.5725	35.4782	2.133945	0.001167	T
ENSG0000(	SPRY4	0.6762	2.062	2.785365	0.001167	T
ENSG0000(	RALY-AS1	0.1904	0.5536	2.250689	0.001168	T
ENSG0000(	C10orf113	0.6551	1.6931	2.374652	0.001169	T
ENSG0000(	PI4K2A	12.941	4.9614	0.388114	0.001173	N
ENSG0000(	SNORA40	3.2584	10.117	3.042222	0.001176	T
ENSG0000(	KCNC3	0.3369	1.3673	3.358434	0.001177	T
ENSG0000(	DZANK1	0.2057	0.5362	2.081125	0.001177	T
ENSG0000(	BOLA3	1.2208	4.616	3.570563	0.001178	T
ENSG0000(	ARMC7	1.5946	3.4874	2.11696	0.001178	T
ENSG0000(	STAG2	10.4943	24.2771	2.300964	0.00118	T
ENSG0000(	CARD8	0.7368	1.9731	2.477414	0.001181	T
ENSG0000(	IFT81	0.6581	1.5951	2.235985	0.001185	T
ENSG0000(	RNU6-127	0.1316	1.6048	7.360967	0.001186	T
ENSG0000(	AC100774	0.2012	1.0915	3.955843	0.001187	T
ENSG0000(	MYBL2	1.6537	20.1088	11.52352	0.001191	T
ENSG0000(	MHENCR	1.2134	2.9688	2.336531	0.001191	T
ENSG0000(	HSF2BP	0.0947	0.4034	2.585516	0.001192	T
ENSG0000(	ZBED9	0.0917	0.515	3.208138	0.001192	T
ENSG0000(	AC005244	0.8229	2.8954	3.245639	0.001193	T
ENSG0000(	THAP10	0.4755	1.3788	2.569592	0.001196	T
ENSG0000(	MXD1	256.8002	56.5152	0.220378	0.001198	N
ENSG0000(	ZNF425	1.5275	0.5314	0.387957	0.001199	N
ENSG0000(	MIR2052H	0.0262	0.8647	7.644216	0.0012	T
ENSG0000(	HSP90B1	38.7261	112.2753	2.894324	0.0012	T
ENSG0000(	IFT22	1.1122	2.7291	2.333856	0.001201	T
ENSG0000(	SMG1P5	0.3717	0.926	2.175111	0.001204	T
ENSG0000(	TTN-AS1	0.5537	1.394	2.285452	0.001205	T
ENSG0000(	B3GNT8	22.3868	6.6709	0.301106	0.001206	N
ENSG0000(	SNX5P1	0.0578	0.3154	2.632446	0.001206	T
ENSG0000(	TRAPPC9	2.1198	4.4148	2.033877	0.001207	T
ENSG0000(	ANAPC13	7.1464	15.3865	2.13713	0.001208	T
ENSG0000(	SLC38A5	1.1025	7.9007	6.653389	0.001209	T
ENSG0000(	AC131235	0.3032	1.456	3.859127	0.00121	T
ENSG0000(	EIF3CL	0.682	1.7374	2.349616	0.00121	T
ENSG0000(	MYO10	2.7445	8.5577	3.043663	0.001211	T
ENSG0000(	FO681548.	0.0564	0.4355	3.423913	0.001213	T
ENSG0000(	EXPH5	34.9642	11.5261	0.331566	0.001213	N
ENSG0000(	H2AFX	15.6163	41.8789	2.671042	0.001213	T
ENSG0000(	TAF4B	1.4637	3.9924	2.617126	0.001214	T
ENSG0000(	THAP7-AS	0.2956	0.7023	2.028059	0.001214	T
ENSG0000(	RNF207	0.7015	2.0448	2.675983	0.001221	T
ENSG0000(	PHETA1	6.4795	1.8461	0.295782	0.001224	N
ENSG0000(	REX1BD	1.8509	3.8607	2.030191	0.001224	T
ENSG0000(	CBX2	0.5815	2.517	3.840059	0.001225	T
ENSG0000(	AL161891.	0.1523	0.5875	2.724931	0.001225	T
ENSG0000(	SFXN4	4.2605	10.2162	2.36583	0.001228	T
ENSG0000(	HIST1H4B	66.643	146.0602	2.189896	0.001231	T
ENSG0000(	CDC25A	0.5993	2.1876	3.271271	0.001233	T

ENSG0000(	PRR5L	0.2493	0.7536	2.443745	0.001233	T
ENSG0000(	ATP2C2	0.2641	4.359	12.24664	0.001236	T
ENSG0000(	PSMB2	11.9012	28.6181	2.392936	0.001237	T
ENSG0000(	TRAP1	7.7431	16.8706	2.163762	0.001239	T
ENSG0000(	TRIQK	1.5502	3.8221	2.376742	0.001241	T
ENSG0000(	ARHGAP1	1.6223	3.8378	2.286361	0.001242	T
ENSG0000(	CDK11A	0.1209	0.3898	2.217293	0.001243	T
ENSG0000(	SPTBN1	9.7069	20.2895	2.079097	0.001244	T
ENSG0000(	CHKA	0.4859	3.2507	5.718894	0.001244	T
ENSG0000(	CLDN1	36.2396	218.587	6.01787	0.001245	T
ENSG0000(	MEX3A	0.2844	1.0579	3.012227	0.001246	T
ENSG0000(	GDPGP1	0.2357	0.6546	2.24784	0.001248	T
ENSG0000(	AC092944	0.1333	0.5218	2.665238	0.001249	T
ENSG0000(	MSI2	1.2742	3.8315	2.860937	0.00125	T
ENSG0000(	SDF2L1	4.1163	8.8117	2.11363	0.001251	T
ENSG0000(	AL031123.	3.9403	1.7353	0.454248	0.001251	N
ENSG0000(	NAA25	2.9485	6.6235	2.205511	0.001253	T
ENSG0000(	PCID2	2.0584	5.0285	2.376066	0.001257	T
ENSG0000(	CARMIL3	0.1771	0.4839	2.107182	0.001257	T
ENSG0000(	AL078621.	0.2213	1.0107	3.456894	0.001257	T
ENSG0000(	SCHIP1	0.0482	0.9409	7.023617	0.001258	T
ENSG0000(	DISC1-IT1	0.0373	0.5092	4.436999	0.001261	T
ENSG0000(	ALOX12P2	0.3206	2.8218	6.946743	0.001262	T
ENSG0000(	OGT	7.8237	22.2944	2.826255	0.001263	T
ENSG0000(	RNU6-118	2.443	10.4634	4.153913	0.001265	T
ENSG0000(	CCDC24	0.2216	1.9381	6.337376	0.001266	T
ENSG0000(	EDC4	2.6771	5.7669	2.112599	0.001266	T
ENSG0000(	ACER1	12.9476	1.9634	0.158144	0.001267	N
ENSG0000(	NME1	3.8738	8.4547	2.152776	0.001268	T
ENSG0000(	PXYLP1	0.2022	1.0241	3.719722	0.00127	T
ENSG0000(	TWISTNB	2.9998	6.7645	2.214498	0.001271	T
ENSG0000(	TMCO3	1.6341	3.9586	2.340465	0.001271	T
ENSG0000(	DQX1	1.0362	2.9792	2.710086	0.001271	T
ENSG0000(	RNA5SP2(	0.2268	2.1392	6.851897	0.001272	T
ENSG0000(	AC091053	0.1139	0.5434	3.007948	0.001273	T
ENSG0000(	SNORD11	2.4336	8.6261	3.444151	0.001273	T
ENSG0000(	GJB7	0	0.7689	8.689	0.001274	T
ENSG0000(	AL390066.	0.1758	0.5438	2.3343	0.001275	T
ENSG0000(	TMOD3	56.165	17.6817	0.316035	0.001276	N
ENSG0000(	C2CD3	1.1816	2.9258	2.360955	0.001277	T
ENSG0000(	CHCHD7	1.4521	3.6325	2.404806	0.001281	T
ENSG0000(	RHEB	9.5304	19.7531	2.061503	0.001283	T
ENSG0000(	RUNDC3A	0.0405	0.2179	2.262633	0.001285	T
ENSG0000(	ODF4	0.3639	0.0594	0.343609	0.001286	N
ENSG0000(	AC023389	0.2159	0.667	2.427984	0.001288	T
ENSG0000(	RGS11	0.6729	0.1592	0.33536	0.001291	N
ENSG0000(	PARP2	1.7794	4.5522	2.475364	0.001292	T
ENSG0000(	SNORA38	1.7705	7.2831	3.947126	0.001294	T
ENSG0000(	ZNF680	1.3462	3.1665	2.258678	0.001294	T
ENSG0000(	TMPRSS2	21.0374	7.8916	0.378079	0.001299	N
ENSG0000(	TAPBP	25.4041	56.2711	2.210276	0.0013	T
ENSG0000(	BID	1.1556	7.813	6.302166	0.001301	T
ENSG0000(	CBX8	0.3228	0.8845	2.328524	0.001302	T
ENSG0000(	AL390957.	0.0434	0.2582	2.497908	0.001306	T
ENSG0000(	SPATC1L	0.7261	1.8672	2.38131	0.001308	T
ENSG0000(	GRINA	15.1879	36.8903	2.41958	0.00131	T
ENSG0000(	FERMT1	8.7399	21.3933	2.431396	0.00131	T
ENSG0000(	SNORA79.	15.8781	48.3196	3.030373	0.001311	T

ENSG0000(	RNU6-33P	3.0658	14.1306	4.495104	0.001311	T
ENSG0000(	Z93403.1	0.0651	0.7304	5.029679	0.001312	T
ENSG0000(	B3GNT3	38.1355	7.9097	0.209483	0.001314	N
ENSG0000(	CRELD2	1.5295	3.682	2.320957	0.001314	T
ENSG0000(	LINC0134.	0.0447	0.208	2.128542	0.001316	T
ENSG0000(	SRRM5	0.045	0.2288	2.267586	0.001317	T
ENSG0000(	RNF121	2.0694	5.0538	2.37568	0.001319	T
ENSG0000(	AL161785.	2.5341	0.5283	0.238525	0.001319	N
ENSG0000(	EPAS1	16.2853	39.1896	2.397857	0.00132	T
ENSG0000(	COPRS	4.2496	9.0239	2.097641	0.001325	T
ENSG0000(	AC009948	0.396	1.3611	2.945766	0.001328	T
ENSG0000(	TMIE	0.6852	0.2293	0.419384	0.001331	N
ENSG0000(	SNX18P13	12.7526	2.0341	0.166044	0.001331	N
ENSG0000(	LNP1	0.2707	0.8893	2.668735	0.001332	T
ENSG0000(	UROD	4.4578	9.4008	2.084514	0.001334	T
ENSG0000(	AC005306	0.2654	0.8605	2.628626	0.001335	T
ENSG0000(	GPR19	0.0638	0.4947	3.630647	0.001335	T
ENSG0000(	AC090181	0.3493	4.1217	9.396172	0.001338	T
ENSG0000(	ADSL	0.283	0.6748	2.022977	0.001339	T
ENSG0000(	TOE1	1.3931	2.9585	2.048423	0.001339	T
ENSG0000(	LINC0215(	0.1377	0.9035	4.221708	0.00134	T
ENSG0000(	CDC25B	2.4109	19.9357	7.979489	0.001341	T
ENSG0000(	ALPK1	0.9783	2.2347	2.165167	0.001341	T
ENSG0000(	ALG13	2.2914	5.0153	2.13904	0.001342	T
ENSG0000(	MIR210HC	5.3042	1.1871	0.238167	0.001342	N
ENSG0000(	DPH6	0.5739	1.4737	2.335213	0.001346	T
ENSG0000(	HMGB1P2	0.2381	0.9325	3.05383	0.001347	T
ENSG0000(	MELTF-A:	0.4091	1.5169	3.175997	0.001348	T
ENSG0000(	CCNL2	4.7727	10.3617	2.147003	0.001351	T
ENSG0000(	SPINDOC	1.1177	4.3827	3.681284	0.001354	T
ENSG0000(	MINCR	0.399	2.0265	4.261523	0.001355	T
ENSG0000(	MIPOL1	0.3763	1.4863	3.330464	0.001358	T
ENSG0000(	PTGES3P1	1.6816	5.9413	3.390941	0.001362	T
ENSG0000(	TBX1	0.2471	2.8479	8.492942	0.001363	T
ENSG0000(	PFAS	0.9037	2.8437	2.932848	0.001365	T
ENSG0000(	SF1	17.8487	37.3701	2.087622	0.001366	T
ENSG0000(	AC007842	0.1678	0.6783	2.906273	0.001367	T
ENSG0000(	MIR2467	0.6526	3.2298	4.424395	0.00137	T
ENSG0000(	HIST1H3A	27.4453	101.1118	3.674376	0.00137	T
ENSG0000(	DNAJC9-A	0.4286	1.0667	2.207151	0.001372	T
ENSG0000(	PRELID2	0.4177	0.997	2.118988	0.001372	T
ENSG0000(	CD276	1.1002	6.1546	5.211298	0.001373	T
ENSG0000(	AL390879.	0.1966	0.8152	3.085637	0.001374	T
ENSG0000(	METTL3	2.1142	4.6622	2.150754	0.001378	T
ENSG0000(	LIN52	1.2487	3.2109	2.454882	0.001378	T
ENSG0000(	RTN4IP1	1.1679	2.7926	2.28141	0.001379	T
ENSG0000(	EGFR	13.4911	61.6717	4.545011	0.00138	T
ENSG0000(	TP53INP2	125.2865	44.5569	0.356154	0.001382	N
ENSG0000(	AC092933	0.0358	0.6968	5.867452	0.001383	T
ENSG0000(	URB1	2.5857	5.5849	2.116729	0.001384	T
ENSG0000(	TMEM165	9.2202	18.6674	2.013626	0.001385	T
ENSG0000(	SIRPA	6.3511	18.0665	2.816031	0.001386	T
ENSG0000(	RDH10	3.7184	35.6256	9.35617	0.001387	T
ENSG0000(	LRP5	3.9324	9.4441	2.366853	0.001392	T
ENSG0000(	RFWD3	3.9734	9.2802	2.302794	0.001397	T
ENSG0000(	NDRG2	30.9327	7.9998	0.261009	0.001398	N
ENSG0000(	SLC1A4	3.7922	19.0296	4.914855	0.001398	T
ENSG0000(	LINC0017.	0.4353	1.0212	2.094526	0.001399	T

ENSG0000(	CDCA4	5.846	16.1608	2.734746	0.001403	T
ENSG0000(	AL118516.	1.0572	5.2662	4.637228	0.001404	T
ENSG0000(	ZDHHC8	2.7595	5.9247	2.106907	0.001406	T
ENSG0000(	PEMT	4.9224	11.6552	2.340554	0.001407	T
ENSG0000(	CXCL14	46.5349	183.1238	3.928899	0.001407	T
ENSG0000(	RNU5E-4P	1.7491	8.3548	4.572387	0.001407	T
ENSG0000(	ZNF783	0.8177	1.7462	2.011769	0.001408	T
ENSG0000(	AC026368	0.1664	0.8167	3.441066	0.00141	T
ENSG0000(	AL928654.	0.1233	0.4745	2.572772	0.00141	T
ENSG0000(	AL390334.	0.01	1.3109	12.82636	0.001411	T
ENSG0000(	DDX12P	0.5376	2.9111	4.722553	0.001415	T
ENSG0000(	LINC0122(	0.226	1.7121	5.558589	0.001416	T
ENSG0000(	RRP1	2.8856	5.9334	2.020833	0.001419	T
ENSG0000(	ARHGAP4	4.8803	1.9285	0.407305	0.001421	N
ENSG0000(	AC018445	0.0586	0.6886	4.972257	0.001422	T
ENSG0000(	C9orf84	0.0185	1.0249	9.492827	0.001423	T
ENSG0000(	COX17	2.0802	6.2073	2.892991	0.001423	T
ENSG0000(	SNORA80	0.8138	15.989	17.6067	0.001424	T
ENSG0000(	ZNF496	0.8108	2.2756	2.608256	0.001424	T
ENSG0000(	FALEC	12.5058	1.1507	0.099216	0.001424	N
ENSG0000(	HSP90AB4	0.2245	0.7109	2.498921	0.001429	T
ENSG0000(	VASH1-AS	0.5594	1.3623	2.217622	0.001431	T
ENSG0000(	AC022149	0.6403	1.7762	2.534378	0.001431	T
ENSG0000(	RPL26P30	0.2003	0.5604	2.199134	0.001432	T
ENSG0000(	SLC25A11	7.5126	15.3074	2.023934	0.001434	T
ENSG0000(	TMCC2	0.186	0.4975	2.089161	0.001434	T
ENSG0000(	SCARNA6	24.5151	67.0629	2.728524	0.00144	T
ENSG0000(	PWWP2B	11.7559	4.8555	0.417978	0.001443	N
ENSG0000(	CYP2D8P	0.2809	0.9891	2.859281	0.001448	T
ENSG0000(	LYAR	2.9434	6.9373	2.312315	0.001454	T
ENSG0000(	MTERF2	0.8268	1.898	2.155805	0.001456	T
ENSG0000(	C15orf48	98.6907	18.3212	0.186467	0.001459	N
ENSG0000(	KCNIP2	0.1432	0.507	2.495888	0.001459	T
ENSG0000(	AQP7P1	0.6726	0.1996	0.387782	0.001459	N
ENSG0000(	LINC0256	1.1991	4.1157	3.245093	0.00146	T
ENSG0000(	MRE11	1.5436	4.5811	2.848077	0.001461	T
ENSG0000(	MTHFS	0.2593	0.7737	2.431673	0.001461	T
ENSG0000(	AC138409	0.4804	1.3669	2.527395	0.001464	T
ENSG0000(	SEPT4-AS	0.0444	0.2651	2.528393	0.001464	T
ENSG0000(	AC008011	0.0316	0.3551	3.458207	0.001464	T
ENSG0000(	RPS6KA1	2.8965	6.9701	2.359453	0.001467	T
ENSG0000(	HIST2H4A	0.7647	3.8478	4.565514	0.001468	T
ENSG0000(	TMSB15A	0.023	0.813	7.422764	0.001468	T
ENSG0000(	AL049840.	1.7425	4.9966	2.766133	0.001471	T
ENSG0000(	SAV1	1.9822	5.4023	2.642542	0.001472	T
ENSG0000(	FAM155B	0.0825	0.911	5.539726	0.001475	T
ENSG0000(	SAPCD2	1.8579	6.6624	3.453905	0.001475	T
ENSG0000(	DUSP9	0.0638	0.8942	6.069597	0.001476	T
ENSG0000(	AC022364	0.3939	1.4131	3.063576	0.001478	T
ENSG0000(	RCC2	26.7255	54.0195	2.017465	0.00148	T
ENSG0000(	MTHFD1	3.7366	9.6623	2.544519	0.001481	T
ENSG0000(	SLC26A4	0.0152	0.5529	5.667535	0.001482	T
ENSG0000(	RAG1	0.052	0.5662	4.382895	0.001485	T
ENSG0000(	EPHB2	0.0576	1.4018	9.529188	0.001493	T
ENSG0000(	ERI3	4.4254	12.2684	2.733106	0.001494	T
ENSG0000(	TMED8	1.8749	3.8683	2.009368	0.001495	T
ENSG0000(	AC108693	1.1159	4.7471	3.98643	0.001496	T
ENSG0000(	DCK	3.2238	6.6785	2.039383	0.001497	T

ENSG0000	RNU5E-3P	0.0464	1.1397	8.467896	0.001497	T
ENSG0000	RHOT2	4.0572	8.2998	2.020543	0.001497	T
ENSG0000	FAM207A	3.145	6.5162	2.038891	0.001498	T
ENSG0000	RF02271	0.1789	1.8673	7.053783	0.0015	T
ENSG0000	PXDN	0.2921	2.4145	6.412905	0.0015	T
ENSG0000	CHD2	4.2089	9.3916	2.20279	0.001501	T
ENSG0000	AARS	5.2861	23.2286	4.33126	0.001502	T
ENSG0000	YJEFN3	0.1326	0.4318	2.286328	0.001502	T
ENSG0000	ZNF789	0.4486	1.3829	2.703062	0.001503	T
ENSG0000	AC007684	0.2313	2.8175	8.806218	0.001504	T
ENSG0000	AC079416	0.2973	0.7223	2.069721	0.001508	T
ENSG0000	BMS1P15	0.2969	0.0565	0.394306	0.001508	N
ENSG0000	RBBP8	7.3047	17.0183	2.311815	0.00151	T
ENSG0000	SIRPAP1	0.0346	0.2154	2.343239	0.001511	T
ENSG0000	SPICE1	0.2419	0.7446	2.470313	0.001513	T
ENSG0000	SNORD12	2.5827	18.0913	6.780967	0.001514	T
ENSG0000	SUPT16H	6.4695	17.9414	2.746236	0.001516	T
ENSG0000	SART3	2.7944	5.9281	2.082677	0.001518	T
ENSG0000	PAPLN	0.5992	1.8094	2.730835	0.001518	T
ENSG0000	TOP3A	1.6688	4.3822	2.534034	0.001519	T
ENSG0000	PIBF1	2.4462	5.4991	2.199002	0.00152	T
ENSG0000	UBE2V1P	0.2973	1.4918	4.006544	0.001524	T
ENSG0000	DDX46	4.0975	8.5636	2.06399	0.001524	T
ENSG0000	AC006206	0.1081	1.8414	9.329169	0.001525	T
ENSG0000	SLC29A1	2.2585	4.9989	2.161925	0.001526	T
ENSG0000	SETD4	0.9146	2.0217	2.091169	0.001526	T
ENSG0000	CCDC91	3.1725	6.5059	2.01861	0.001529	T
ENSG0000	MIR570	11.147	28.0981	2.507166	0.001529	T
ENSG0000	AC009902	0.8716	2.9747	3.164574	0.00153	T
ENSG0000	TMEM38B	1.1203	2.7057	2.299189	0.001533	T
ENSG0000	AL450336	0.0325	0.2184	2.403019	0.001533	T
ENSG0000	CLPB	0.9247	2.875	2.903289	0.001533	T
ENSG0000	SLC38A7	1.2689	3.0189	2.278399	0.001536	T
ENSG0000	STARD7-A	0.5497	1.8709	3.033554	0.001537	T
ENSG0000	AC011939	0.5376	3.6239	5.840496	0.001537	T
ENSG0000	NKILA	0.0955	0.3799	2.454731	0.001537	T
ENSG0000	PIGZ	0.6554	1.7315	2.424543	0.001537	T
ENSG0000	CEP19	0.3796	2.8008	6.048374	0.001539	T
ENSG0000	RFX5	4.0174	8.7234	2.142954	0.00154	T
ENSG0000	AC006329	1.0855	5.3933	4.633741	0.001545	T
ENSG0000	FP325330.	0.0045	0.4155	4.933014	0.001546	T
ENSG0000	TMEM135	1.2708	3.8209	2.860301	0.001546	T
ENSG0000	SHMT2	5.891	14.7347	2.476164	0.001548	T
ENSG0000	PTTG1	5.3595	18.243	3.359831	0.001549	T
ENSG0000	AC008771	0.7735	2.5982	3.088952	0.00155	T
ENSG0000	TCF7	0.3907	1.1026	2.450785	0.001552	T
ENSG0000	PSAT1	7.1683	21.4824	2.969388	0.001553	T
ENSG0000	HIVEP1	6.5604	13.9348	2.107201	0.001554	T
ENSG0000	ACAD9	6.0785	13.3278	2.173311	0.001557	T
ENSG0000	AC064799	0.2927	0.9552	2.687038	0.001557	T
ENSG0000	TUBA1B	44.5277	101.8497	2.284449	0.001559	T
ENSG0000	GRHL1	44.3747	20.6344	0.466207	0.001562	N
ENSG0000	PHACTR4	45.4366	12.3133	0.272601	0.001563	N
ENSG0000	LPP-AS2	0.3557	1.1179	2.672592	0.001565	T
ENSG0000	B3GALT6	1.9749	4.7568	2.340739	0.001567	T
ENSG0000	MEST	0.9207	7.7442	7.685118	0.001567	T
ENSG0000	DNM1P38	0.03	0.4993	4.61	0.00157	T
ENSG0000	AC116158	0.0743	0.4107	2.930006	0.001571	T

ENSG0000(	HIST2H2E	32.7466	65.9929	2.012169	0.001572	T
ENSG0000(	FBXL6	0.9806	3.2261	3.078012	0.001573	T
ENSG0000(	ANXA9	29.1095	2.2704	0.081152	0.001574	N
ENSG0000(	SELENOW	8.4112	18.1759	2.147277	0.001574	T
ENSG0000(	LOXL4	0.2592	1.7062	5.028396	0.001574	T
ENSG0000(	RCE1	2.5924	5.4178	2.049398	0.001576	T
ENSG0000(	ZNRF1	10.9715	4.6444	0.428524	0.001577	N
ENSG0000(	GRHL2	10.1835	20.9693	2.048845	0.001581	T
ENSG0000(	DCAF16	3.4568	7.4529	2.12351	0.001582	T
ENSG0000(	CNIH2	0.1888	0.8711	3.362535	0.001582	T
ENSG0000(	TRMT13	2.2224	5.4367	2.384042	0.001582	T
ENSG0000(	RPS4XP17	0.8629	1.834	2.008516	0.001585	T
ENSG0000(	PCAT7	0.0025	0.4806	5.66439	0.001585	T
ENSG0000(	HMGB3P6	0.007	0.2209	2.999065	0.001587	T
ENSG0000(	AC015712	0.1504	0.4276	2.107029	0.001587	T
ENSG0000(	CHCHD6	1.0249	2.9838	2.741399	0.001589	T
ENSG0000(	CENPS	0.2547	0.7606	2.426276	0.001589	T
ENSG0000(	MMD	1.0152	3.4031	3.14123	0.00159	T
ENSG0000(	FAM3C2	3.7255	7.5943	2.011319	0.001591	T
ENSG0000(	HIST1H2A	25.3852	95.5822	3.754422	0.001593	T
ENSG0000(	APMAP	6.7196	28.5183	4.196478	0.001596	T
ENSG0000(	AP001160.	0.2286	0.6224	2.198418	0.001597	T
ENSG0000(	NUDC	7.4227	15.078	2.017627	0.001598	T
ENSG0000(	FLCN	0.6882	1.5274	2.064704	0.0016	T
ENSG0000(	CHPF2	2.961	7.9295	2.623162	0.001604	T
ENSG0000(	ERCC4	0.8304	1.8719	2.119411	0.001605	T
ENSG0000(	CASC9	0.0057	12.5167	119.3633	0.001607	T
ENSG0000(	FAM214B	10.2213	3.8177	0.379574	0.001607	N
ENSG0000(	C3AR1	1.0184	4.1371	3.788537	0.00161	T
ENSG0000(	PYROXD2	0.2619	0.7232	2.274662	0.00161	T
ENSG0000(	ANXA11	45.1131	19.4584	0.432583	0.001615	N
ENSG0000(	RRS1	3.1244	10.2619	3.21359	0.001615	T
ENSG0000(	AC009053	0.7426	1.9817	2.470567	0.001617	T
ENSG0000(	EMSY	1.7025	3.6326	2.070791	0.001621	T
ENSG0000(	LIMK1	2.372	5.8203	2.394943	0.001621	T
ENSG0000(	SNORA14.	9.6589	31.3635	3.224083	0.001623	T
ENSG0000(	AGFG1	8.0477	18.2058	2.246744	0.001625	T
ENSG0000(	ZNF182	0.7055	1.5764	2.081192	0.001627	T
ENSG0000(	FAM209A	0.0722	0.3161	2.416376	0.001629	T
ENSG0000(	AP5Z1	0.7496	1.9475	2.409958	0.001629	T
ENSG0000(	MRPL48	1.3845	3.0032	2.090401	0.001629	T
ENSG0000(	HNRNPA1	0.9462	2.2246	2.221946	0.001631	T
ENSG0000(	SOCS4	3.8988	8.1799	2.070596	0.001636	T
ENSG0000(	AC024451	0.3881	2.2421	4.798402	0.001636	T
ENSG0000(	ACP1	6.8589	15.8541	2.292618	0.001636	T
ENSG0000(	SRPRB	8.0705	20.2406	2.489517	0.001636	T
ENSG0000(	E2F4	6.2166	13.972	2.227781	0.001638	T
ENSG0000(	AL390037.	0	0.2392	3.392	0.00164	T
ENSG0000(	TXLNG	2.5903	5.6134	2.123704	0.001643	T
ENSG0000(	ASAP1-IT2	0.093	0.5396	3.31399	0.001647	T
ENSG0000(	AC025031	0.0405	0.3004	2.849822	0.00165	T
ENSG0000(	ZNF594	0.4229	1.2279	2.539491	0.001657	T
ENSG0000(	BCAT2	1.7592	4.8519	2.663457	0.001657	T
ENSG0000(	ZNF365	19.1089	1.5323	0.084976	0.001662	N
ENSG0000(	NMI	2.8784	8.0859	2.748422	0.001663	T
ENSG0000(	ZNF711	0.4719	1.88	3.462144	0.001663	T
ENSG0000(	AC002524	0.1122	0.6563	3.56409	0.001666	T
ENSG0000(	AP004245.	0.1985	1.3785	4.953099	0.00167	T

ENSG0000(	EBLN2	0.5021	1.3313	2.37718	0.001671	T
ENSG0000(	HSPE1P4	0.67	3.1018	4.158182	0.001672	T
ENSG0000(	PPP3CB-A	0.3447	0.7907	2.002923	0.001674	T
ENSG0000(	LINC0157(	4.0699	14.1371	3.414255	0.001674	T
ENSG0000(	RIDA	3.1983	7.5062	2.306097	0.001676	T
ENSG0000(	RASAL1	7.6769	3.2854	0.435315	0.001677	N
ENSG0000(	FIRRE	0.0119	1.2854	12.3807	0.00168	T
ENSG0000(	SNRNP25	1.4717	4.1803	2.723357	0.001684	T
ENSG0000(	PLPPR1	0.0333	0.2756	2.817704	0.001685	T
ENSG0000(	AC018445	0.0393	0.6632	5.478823	0.001685	T
ENSG0000(	PROCR	1.1306	5.1729	4.28482	0.001695	T
ENSG0000(	TMEM97	2.1678	8.0278	3.584002	0.001698	T
ENSG0000(	TRAM2	1.1432	4.8131	3.951979	0.001698	T
ENSG0000(	RNU6-116	0.8411	7.862	8.460312	0.001698	T
ENSG0000(	CD3EAP	1.1317	3.0912	2.590891	0.001702	T
ENSG0000(	LAP3P2	0.0434	0.1991	2.085774	0.001702	T
ENSG0000(	SMAD1	2.0592	4.4915	2.126482	0.001702	T
ENSG0000(	HRASLS	1.6721	3.4558	2.006546	0.001702	T
ENSG0000(	CTSC	3.8517	21.712	5.51965	0.001703	T
ENSG0000(	GIPR	0.2114	0.6502	2.40912	0.001703	T
ENSG0000(	GTF2H3	3.574	7.8392	2.160915	0.001706	T
ENSG0000(	AC036176	2.332	10.6079	4.402919	0.001707	T
ENSG0000(	RPS25P9	0.1335	1.0107	4.756745	0.001708	T
ENSG0000(	AGT	0.0223	0.239	2.771872	0.001709	T
ENSG0000(	ZNF677	0.5399	0.1237	0.349586	0.00171	N
ENSG0000(	POLD3	1.5065	4.2247	2.692001	0.001712	T
ENSG0000(	LRIG1	2.8377	5.913	2.046839	0.001714	T
ENSG0000(	Z94721.3	0.3138	1.0824	2.857419	0.001715	T
ENSG0000(	KRT78	588.6766	29.6232	0.050483	0.001717	N
ENSG0000(	ALYREF	9.8815	23.3214	2.346481	0.001718	T
ENSG0000(	MCCC1	5.6672	12.3444	2.157789	0.001723	T
ENSG0000(	AC114956	0.1437	1.9888	8.571194	0.001724	T
ENSG0000(	RN7SL173	0.0775	0.8658	5.441127	0.001725	T
ENSG0000(	AATF	3.2836	7.8562	2.351401	0.001729	T
ENSG0000(	ZNF641	1.5819	4.1957	2.554076	0.001732	T
ENSG0000(	HERC2	2.3724	4.8734	2.011568	0.001733	T
ENSG0000(	ACD	1.0803	2.7725	2.433703	0.001737	T
ENSG0000(	DNAH1	0.5245	1.1511	2.003363	0.001738	T
ENSG0000(	P2RX4	0.3256	1.2729	3.225799	0.00174	T
ENSG0000(	STRADA	0.2469	0.6167	2.066013	0.001741	T
ENSG0000(	B4GALNT	4.6852	10.2638	2.165803	0.001746	T
ENSG0000(	KPTN	0.8983	3.2679	3.373635	0.001747	T
ENSG0000(	BZW2	7.2827	17.2022	2.343614	0.001749	T
ENSG0000(	AL049830.	0.1655	1.2429	5.058004	0.00175	T
ENSG0000(	C3orf58	6.7877	20.9283	3.053022	0.00175	T
ENSG0000(	GOT2	20.0609	41.73	2.074808	0.001753	T
ENSG0000(	TAS2R46	0.1544	0.9731	4.21816	0.001753	T
ENSG0000(	AC005822	0.3544	1.3508	3.192782	0.001756	T
ENSG0000(	AL049734.	0	0.4093	5.093	0.001759	T
ENSG0000(	FHOD1	0.701	2.4082	3.131336	0.00176	T
ENSG0000(	SGF29	2.5613	5.4628	2.090257	0.001762	T
ENSG0000(	NDUFAB1	9.5371	20.1343	2.099625	0.001763	T
ENSG0000(	AC012442	0.2761	0.9084	2.681202	0.001765	T
ENSG0000(	RNU6-101	0.0763	1.2971	7.92456	0.001769	T
ENSG0000(	AIMP2	5.3819	11.0013	2.025083	0.001769	T
ENSG0000(	HDAC1P2	0.2495	1.4973	4.570243	0.00177	T
ENSG0000(	HPCAL1	1.1246	4.1927	3.50539	0.001771	T
ENSG0000(	ANXA2R	0.3123	0.934	2.507883	0.001772	T

ENSG0000(	PIMREG	0.3442	2.2916	5.384061	0.001775	T
ENSG0000(	SLC25A22	1.3958	2.9779	2.057695	0.001776	T
ENSG0000(	GAS2L3	0.7033	1.7585	2.313581	0.001776	T
ENSG0000(	SLC38A10	2.7848	6.1599	2.16996	0.00178	T
ENSG0000(	MACF1	5.4419	11.1417	2.028492	0.00178	T
ENSG0000(	GNMT	0.09	0.2951	2.079474	0.001783	T
ENSG0000(	COL6A4P1	0.017	0.2276	2.8	0.001784	T
ENSG0000(	RNU6-414	0.3727	2.2787	5.032156	0.001787	T
ENSG0000(	ODF2L	1.6938	4.2983	2.451946	0.001788	T
ENSG0000(	GSDMD	1.1366	4.0222	3.333495	0.00179	T
ENSG0000(	RBM14-RI	0.2899	1.163	3.239292	0.00179	T
ENSG0000(	LINC0051!	0.5833	3.9094	5.867701	0.001792	T
ENSG0000(	AC020978	0	0.2241	3.241	0.001798	T
ENSG0000(	AC011491	0.1369	0.6466	3.151541	0.001803	T
ENSG0000(	ABCC2	0.0491	0.3161	2.790744	0.001805	T
ENSG0000(	FOXD3-AS	0.0583	0.5564	4.146557	0.00181	T
ENSG0000(	AC092120	0.5165	2.1546	3.657097	0.001811	T
ENSG0000(	9-Sep	7.2206	17.0353	2.340696	0.001813	T
ENSG0000(	IQCA1	0.2173	2.0466	6.765206	0.001816	T
ENSG0000(	NXPE3	0.5596	1.6726	2.687386	0.001821	T
ENSG0000(	RAB32	1.5975	7.1228	4.254963	0.001826	T
ENSG0000(	NAIP	0.1076	0.3758	2.291908	0.001828	T
ENSG0000(	DCBLD2	1.4742	3.779	2.464109	0.001828	T
ENSG0000(	KRT16P3	1.0734	2.2768	2.025567	0.001834	T
ENSG0000(	ZNF507	2.0735	4.436	2.086957	0.001838	T
ENSG0000(	SMG1P2	0.5189	1.44	2.488286	0.001844	T
ENSG0000(	ANKRD23	0.0899	0.2896	2.051606	0.001848	T
ENSG0000(	PCSK5	4.5099	1.5426	0.35632	0.00185	N
ENSG0000(	AL133297.	0.11	0.4356	2.550476	0.001857	T
ENSG0000(	TGIF2	1.7024	5.2172	2.950067	0.001858	T
ENSG0000(	AC048341	0.1657	0.5099	2.295446	0.001859	T
ENSG0000(	SNORA74	0.574	2.0822	3.237685	0.001859	T
ENSG0000(	LAMTOR5	0.0913	0.3086	2.135912	0.00186	T
ENSG0000(	CDKN2B	45.9611	15.7866	0.344903	0.001862	N
ENSG0000(	MBIP	3.1352	7.0052	2.196217	0.001866	T
ENSG0000(	PMEPA1	1.6585	6.4071	3.70037	0.001867	T
ENSG0000(	SLC33A1	0.7147	2.5139	3.20842	0.001869	T
ENSG0000(	SMG1	4.842	9.7955	2.002327	0.00187	T
ENSG0000(	AC009303	0.0677	0.3281	2.552773	0.001871	T
ENSG0000(	TMEM208	3.5404	7.8267	2.177426	0.001875	T
ENSG0000(	CTSF	1.9416	4.9773	2.486922	0.001875	T
ENSG0000(	IGSF3	8.5702	20.5387	2.380418	0.001878	T
ENSG0000(	CD320	0.8514	3.5593	3.846227	0.00188	T
ENSG0000(	LBX2	0.1037	0.5868	3.371625	0.001883	T
ENSG0000(	NKRF	1.6932	5.3551	3.042104	0.001885	T
ENSG0000(	ABCF2	0.8573	2.1792	2.380863	0.00189	T
ENSG0000(	EP400P1	0.3229	0.7798	2.080397	0.00189	T
ENSG0000(	TDP2	6.4645	18.2319	2.792581	0.001891	T
ENSG0000(	EIF4A1	0.8118	2.2177	2.541895	0.001892	T
ENSG0000(	ADI1	6.8782	15.0811	2.175504	0.001893	T
ENSG0000(	PC	2.7115	8.2473	2.968985	0.001894	T
ENSG0000(	MIR1255A	0	1.4803	15.803	0.001895	T
ENSG0000(	ERICH6	0.0309	0.1722	2.07945	0.001898	T
ENSG0000(	ETS2	19.8955	68.0767	3.409602	0.001899	T
ENSG0000(	HOMER1	2.0963	4.8101	2.235624	0.001901	T
ENSG0000(	LINC0202!	0.0089	0.2196	2.934803	0.001905	T
ENSG0000(	JAG1	23.6972	57.5164	2.421142	0.001905	T
ENSG0000(	MUM1	1.3557	3.3553	2.373635	0.001905	T

ENSG0000(	COL9A3	0.0792	0.4156	2.877232	0.001909	T
ENSG0000(	RF00003	12.3565	84.9832	6.830426	0.001909	T
ENSG0000(	FABP5P3	0.1371	0.3851	2.045972	0.001911	T
ENSG0000(	ZSCAN16-	0.3301	1.0444	2.660777	0.001912	T
ENSG0000(	NCKAP5L	0.9324	2.2761	2.30153	0.001913	T
ENSG0000(	AC079684	0.2814	1.6049	4.47011	0.001919	T
ENSG0000(	URB1-AS1	1.5504	3.6052	2.245032	0.001919	T
ENSG0000(	FMO2	91.0424	20.3101	0.223936	0.001921	N
ENSG0000(	ZNF503-A	3.4479	0.6125	0.200823	0.001923	N
ENSG0000(	AC079416	0.144	0.4665	2.321721	0.001924	T
ENSG0000(	HTATSF1l	0.4	2.3232	4.8464	0.001926	T
ENSG0000(	AC063965	1.3265	3.5083	2.529478	0.001926	T
ENSG0000(	SLC25A25	0.2953	0.7657	2.189982	0.001928	T
ENSG0000(	VMP1	19.5503	61.6678	3.143352	0.00193	T
ENSG0000(	FGFR4	0.1287	0.4221	2.282903	0.001931	T
ENSG0000(	CECR2	0.1102	2.6399	13.03473	0.001931	T
ENSG0000(	RPS20P33	0.3486	1.1878	2.870709	0.001934	T
ENSG0000(	VCPIP1	4.0926	8.2997	2.003458	0.001937	T
ENSG0000(	RANP1	1.2618	3.3368	2.523719	0.001941	T
ENSG0000(	AC016894	0.0894	1.0313	5.973073	0.001943	T
ENSG0000(	ARAP1	3.5842	7.7922	2.142175	0.001945	T
ENSG0000(	PSORS1C1	0.2479	0.7635	2.482035	0.001946	T
ENSG0000(	ZSCAN31	2.5591	8.0661	3.071001	0.001947	T
ENSG0000(	PLXNB3	0.4091	1.6813	3.49892	0.001949	T
ENSG0000(	AC136469	0.1468	0.4075	2.056321	0.001954	T
ENSG0000(	SND1-IT1	0.1557	0.8908	3.874853	0.001956	T
ENSG0000(	AC005753	0.5638	0.1117	0.318921	0.001957	N
ENSG0000(	AP001350.	0.1382	0.6289	3.060034	0.001959	T
ENSG0000(	ECI1	4.1728	8.4579	2.002879	0.001959	T
ENSG0000(	OSMR	3.1082	16.2545	5.097718	0.00196	T
ENSG0000(	MFSD2A	2.3552	8.4174	3.469127	0.001965	T
ENSG0000(	AL590652.	0.5025	1.3884	2.470373	0.001967	T
ENSG0000(	KRTCAP3	1.6344	7.4728	4.366236	0.001967	T
ENSG0000(	STK24	43.3324	19.933	0.461246	0.001968	N
ENSG0000(	MORN2	2.541	5.2868	2.039682	0.001969	T
ENSG0000(	AC019349	78.9596	26.5406	0.336969	0.001971	N
ENSG0000(	PPIE	1.3735	2.9106	2.043163	0.001971	T
ENSG0000(	PDPR	1.7372	3.7538	2.097649	0.001971	T
ENSG0000(	AC092692	0.0673	0.339	2.624029	0.001974	T
ENSG0000(	KIF3C	0.4208	1.4468	2.970046	0.001975	T
ENSG0000(	ACSF3	0.7031	1.6222	2.14444	0.001977	T
ENSG0000(	ARHGEF4	0.6142	1.3788	2.070568	0.001978	T
ENSG0000(	POFUT1	3.1289	9.5103	2.976339	0.001979	T
ENSG0000(	RGS9BP	0.4135	0.121	0.43038	0.00198	N
ENSG0000(	AL132989.	0.245	1.4831	4.588696	0.001981	T
ENSG0000(	EXOSC3P	0.3795	1.0878	2.477164	0.001984	T
ENSG0000(	AC022217	0.0881	0.4021	2.669325	0.001985	T
ENSG0000(	GTF2IRD2	0.0498	0.7252	5.508678	0.001987	T
ENSG0000(	OVOL1-AS	0.4113	0.0597	0.312341	0.001988	N
ENSG0000(	KLHL31	0.0779	0.321	2.366498	0.001993	T
ENSG0000(	UQCC1	2.0483	4.8815	2.31881	0.001996	T
ENSG0000(	TWNK	1.1644	2.6422	2.168776	0.001998	T
ENSG0000(	RAD9A	1.6587	4.0174	2.341161	0.002001	T
ENSG0000(	KANSL3	2.7714	6.2962	2.227555	0.002001	T
ENSG0000(	SMG1P6	0.4097	1.2859	2.71905	0.002002	T
ENSG0000(	LXN	3.0123	6.8463	2.231886	0.002005	T
ENSG0000(	CNPY3	7.4375	21.0716	2.808836	0.002006	T
ENSG0000(	MIR944	3.0689	17.9383	5.692291	0.002009	T

ENSG0000(	NKAIN2	0.0252	1.512	12.8754	0.002013	T
ENSG0000(	STX17-AS	1.8502	0.7576	0.43975	0.002014	N
ENSG0000(	TNC	7.4184	41.0817	5.477455	0.002014	T
ENSG0000(	KCNJ14	0.18	0.5383	2.279643	0.002015	T
ENSG0000(	AC016876	0.8392	2.0537	2.293122	0.002016	T
ENSG0000(	ISPD	0.3508	1.2612	3.019521	0.002017	T
ENSG0000(	CDC42-IT:	0.5343	1.7077	2.849913	0.002021	T
ENSG0000(	B4GALT1	63.2229	28.2583	0.447836	0.002022	N
ENSG0000(	CELSR3	0.0881	1.008	5.890484	0.002026	T
ENSG0000(	RPS21P4	1.8653	3.8604	2.015163	0.002028	T
ENSG0000(	KYNU	0.4869	2.8803	5.078037	0.002028	T
ENSG0000(	POLR2B	8.8322	18.1613	2.044435	0.002033	T
ENSG0000(	ADCK5	1.4716	3.416	2.23721	0.002034	T
ENSG0000(	DDX10	1.3911	2.976	2.062907	0.002035	T
ENSG0000(	IQCH-AS1	0.3456	0.8613	2.157316	0.002044	T
ENSG0000(	AC096921	0.2026	0.6379	2.438533	0.002044	T
ENSG0000(	AP000696.	0.3626	0.0479	0.319715	0.002048	N
ENSG0000(	CDKL5	4.7991	1.6963	0.366659	0.002052	N
ENSG0000(	TIMM22	2.2471	4.7987	2.087129	0.002052	T
ENSG0000(	C2orf16	0.3185	0.0356	0.324014	0.002052	N
ENSG0000(	AC135977	0.0894	0.4758	3.040127	0.002053	T
ENSG0000(	DLX4	0.0979	0.7709	4.400707	0.002056	T
ENSG0000(	AHSA2P	1.0317	3.2224	2.93576	0.002056	T
ENSG0000(	AC073415	0.531	2.8096	4.611094	0.002059	T
ENSG0000(	PLEKHG4	0.4743	1.8528	3.400313	0.002061	T
ENSG0000(	CFAP45	0.0462	0.26	2.46238	0.002061	T
ENSG0000(	PRDX3	8.8769	20.5733	2.302944	0.002062	T
ENSG0000(	ERVK3-1	0.7958	2.128	2.487162	0.002063	T
ENSG0000(	AL049776.	0.085	0.2733	2.017838	0.002064	T
ENSG0000(	YRDC	3.6574	9.1666	2.466227	0.002066	T
ENSG0000(	HIST2H4B	0.1797	0.8489	3.392563	0.002066	T
ENSG0000(	ZNRF3-IT:	0.1342	0.4562	2.374893	0.002066	T
ENSG0000(	SLC4A1AI	0.0721	0.3482	2.6043	0.002067	T
ENSG0000(	MATR3	0.4592	1.319	2.537554	0.002069	T
ENSG0000(	LENG8	7.0479	14.5986	2.056352	0.002073	T
ENSG0000(	SEC14L5	0.4279	0.1235	0.423376	0.002074	N
ENSG0000(	C8orf33	3.0635	6.8223	2.188178	0.002076	T
ENSG0000(	KMT5C	0.7415	2.0651	2.572906	0.002077	T
ENSG0000(	CAV2	5.1974	11.7296	2.233095	0.00208	T
ENSG0000(	ELP2	1.8262	3.8997	2.076472	0.002081	T
ENSG0000(	TGDS	1.7736	4.1608	2.274125	0.002084	T
ENSG0000(	CAGE1	0.0288	0.1982	2.315217	0.002095	T
ENSG0000(	HOXD9	0.2967	2.0322	5.374842	0.002095	T
ENSG0000(	FAM183A	0.4792	2.5875	4.640021	0.002101	T
ENSG0000(	CSNK2A3	0.421	1.0436	2.19501	0.002103	T
ENSG0000(	MARS2	2.0751	5.48	2.565399	0.002103	T
ENSG0000(	AC010536	0.0559	0.3496	2.8839	0.002104	T
ENSG0000(	HMGB1P5	1.5996	4.4833	2.696693	0.002105	T
ENSG0000(	FCHO2	29.2062	12.2966	0.423003	0.002105	N
ENSG0000(	C8orf37	0.593	1.3633	2.111544	0.002111	T
ENSG0000(	RASAL2	11.2218	4.8918	0.440902	0.002111	N
ENSG0000(	HSPA8P5	0.3183	0.751	2.034425	0.002114	T
ENSG0000(	MIR8058	0.1009	1.9432	10.17023	0.002117	T
ENSG0000(	PSMB3	31.7741	74.6581	2.345418	0.002119	T
ENSG0000(	AASS	2.977	7.1743	2.364088	0.00212	T
ENSG0000(	AC010680	0.6907	2.4364	3.207791	0.002121	T
ENSG0000(	MRPS15	7.8607	16.3164	2.06218	0.002121	T
ENSG0000(	AC023644	0.0048	0.488	5.610687	0.002122	T

ENSG0000(	MIR553	0.73	3.7159	4.59747	0.002125	T
ENSG0000(	AL031736.	0.0117	0.8391	8.407341	0.002125	T
ENSG0000(	SCD5	1.1118	2.7824	2.37861	0.002128	T
ENSG0000(	CCM2	1.1779	2.4951	2.030754	0.002134	T
ENSG0000(	AC018716	0	1.3443	14.443	0.002135	T
ENSG0000(	AC027796	0.1551	0.9666	4.181105	0.002137	T
ENSG0000(	AC008906	0.5388	1.5741	2.620695	0.002138	T
ENSG0000(	SNORD20	3.9595	15.6412	3.87762	0.002139	T
ENSG0000(	LINC0156(	0.4037	1.3913	2.960691	0.002141	T
ENSG0000(	NDUFS6	8.8318	18.2037	2.049273	0.002144	T
ENSG0000(	IRAK1	14.8181	31.1553	2.095126	0.002145	T
ENSG0000(	RF00424	0.0763	0.4104	2.895065	0.002146	T
ENSG0000(	TAX1BP1	42.8089	14.8271	0.347879	0.002146	N
ENSG0000(	QSOX2	1.0031	3.3545	3.131629	0.002157	T
ENSG0000(	AL022328.	0.1212	0.5324	2.858951	0.002158	T
ENSG0000(	CYP4X1	3.6894	12.9276	3.437906	0.00216	T
ENSG0000(	SPACA9	3.1612	1.525	0.498283	0.002161	N
ENSG0000(	LINC0202	0.1007	0.4689	2.834579	0.002163	T
ENSG0000(	CDK4	6.8353	23.9757	3.471472	0.002164	T
ENSG0000(	ARMH4	0.3299	0.7701	2.023959	0.002164	T
ENSG0000(	Z97653.2	1.2332	0.2793	0.284503	0.002166	N
ENSG0000(	SLCO3A1	3.2482	7.3628	2.228899	0.002171	T
ENSG0000(	SPATA41	0.0659	0.2702	2.231465	0.002175	T
ENSG0000(	AC117394	0.112	0.5004	2.832075	0.002176	T
ENSG0000(	LAMA5	4.7344	16.4075	3.414591	0.002177	T
ENSG0000(	MDM1	0.5224	1.2559	2.178503	0.002178	T
ENSG0000(	BBOX1	5.7695	1.4591	0.265627	0.002179	N
ENSG0000(	NRSN2-AS	0.5896	2.9115	4.367024	0.002181	T
ENSG0000(	LINC0167	0.1373	0.4461	2.301306	0.002187	T
ENSG0000(	AIFM2	4.7645	1.8411	0.399034	0.002189	N
ENSG0000(	RF00322	0.0597	1.5917	10.59299	0.002191	T
ENSG0000(	TMEM45E	32.1604	14.0242	0.437819	0.002197	N
ENSG0000(	TUBBP1	0.5506	2.2833	3.663234	0.002198	T
ENSG0000(	RNU6ATA	9.16	24.7522	2.683823	0.002199	T
ENSG0000(	AL354707.	0.0459	0.2	2.056203	0.002202	T
ENSG0000(	AC026401	0.2461	0.9354	2.991621	0.002204	T
ENSG0000(	PRR19	0.1161	0.3871	2.254049	0.002204	T
ENSG0000(	PSRC1	0.7168	1.8831	2.427889	0.002204	T
ENSG0000(	AC024592	6.5646	0.2076	0.046154	0.002206	N
ENSG0000(	ENO3	0.226	0.5627	2.032822	0.002213	T
ENSG0000(	AC008870	0.1654	0.6019	2.644687	0.002213	T
ENSG0000(	AL008729.	0.0748	0.3628	2.647597	0.002215	T
ENSG0000(	AL022722.	0.0824	0.928	5.635965	0.002219	T
ENSG0000(	AP001962.	0.8819	3.4715	3.637336	0.00222	T
ENSG0000(	NUP107	1.5895	5.377	3.241788	0.002221	T
ENSG0000(	ABLIM3	28.3715	3.6877	0.133035	0.002222	N
ENSG0000(	MYZAP	13.7761	1.0423	0.082321	0.002227	N
ENSG0000(	E2F3-IT1	0.4669	2.7206	4.975481	0.002231	T
ENSG0000(	ARHGAP5	7.9347	2.9085	0.374438	0.002234	N
ENSG0000(	BAK1P1	0.1357	0.4211	2.210861	0.002237	T
ENSG0000(	PHTF1	0.4754	1.3371	2.497567	0.00224	T
ENSG0000(	SESN3	9.0918	35.7332	3.898388	0.00224	T
ENSG0000(	ZNF771	0.2779	0.7244	2.18153	0.002245	T
ENSG0000(	VWA8-AS	0	0.2129	3.129	0.002249	T
ENSG0000(	CEP290	0.5456	1.8004	2.943618	0.002249	T
ENSG0000(	LINC0086	0.0361	0.2845	2.825129	0.002251	T
ENSG0000(	FBL	20.2615	42.3968	2.087115	0.002251	T
ENSG0000(	SPON1-AS	0.2157	2.3453	7.745645	0.002253	T

ENSG0000(	AC090282	0.0055	0.2803	3.604739	0.002253	T
ENSG0000(	TFDP2	1.9255	4.183	2.11454	0.002254	T
ENSG0000(	AC117395	0.0162	0.3461	3.839071	0.002259	T
ENSG0000(	ACOT9	0.9303	3.6485	3.638261	0.00226	T
ENSG0000(	LINC0156	0.3549	2.3186	5.316773	0.002266	T
ENSG0000(	ST3GAL3	0.0597	0.2929	2.460238	0.002269	T
ENSG0000(	REXO5	0.4289	1.0274	2.131594	0.002271	T
ENSG0000(	IGFBP6	1.5498	17.9027	10.91205	0.002276	T
ENSG0000(	C12orf60	0.0686	0.2575	2.120403	0.002277	T
ENSG0000(	DUSP10	2.5687	6.9802	2.653052	0.002277	T
ENSG0000(	LINC0196	0.1193	2.2214	10.5855	0.00228	T
ENSG0000(	AL049555.	14.7872	29.6898	2.001034	0.002281	T
ENSG0000(	AL022322.	0.1423	0.5841	2.823359	0.002283	T
ENSG0000(	RORA-AS	0.1319	0.3769	2.05649	0.002286	T
ENSG0000(	AC007681	0.032	0.2064	2.321212	0.002294	T
ENSG0000(	HOXC9	0.168	1.0837	4.416791	0.002305	T
ENSG0000(	SART1	4.2576	10.3599	2.400381	0.002306	T
ENSG0000(	AC092431	0.1433	0.7049	3.308261	0.002308	T
ENSG0000(	SYBU	0.319	1.023	2.680191	0.002308	T
ENSG0000(	RNU1-72P	0	1.2075	13.075	0.002308	T
ENSG0000(	RABGGTI	3.6966	8.3287	2.220065	0.002315	T
ENSG0000(	CD27-AS1	0.5965	1.4671	2.249964	0.002316	T
ENSG0000(	ZNF343	0.7722	1.6831	2.044371	0.002319	T
ENSG0000(	HSD3B7	1.3601	4.2919	3.007945	0.002321	T
ENSG0000(	TMEM64	2.2552	5.5126	2.383067	0.002322	T
ENSG0000(	EHD2	11.0153	23.392	2.113483	0.002324	T
ENSG0000(	BLOC1S3	1.856	4.3677	2.2841	0.002325	T
ENSG0000(	SSSCA1-A	0.6344	1.4644	2.130174	0.002325	T
ENSG0000(	SLC7A5	30.267	94.7156	3.122324	0.002327	T
ENSG0000(	AL158077.	0.0239	0.3503	3.634383	0.002331	T
ENSG0000(	FOXO6	0.2221	0.8858	3.06054	0.002331	T
ENSG0000(	AC007620	0.2255	0.936	3.182796	0.002332	T
ENSG0000(	AL513523.	0.3472	0.8829	2.197898	0.002333	T
ENSG0000(	AL157834.	0.048	0.5472	4.372973	0.002336	T
ENSG0000(	AC005839	0.531	1.2894	2.201902	0.00234	T
ENSG0000(	STAG3L2	0.8594	2.9792	3.209506	0.002342	T
ENSG0000(	AC010186	0.3501	1.1081	2.68407	0.002342	T
ENSG0000(	AL139095.	0	0.3815	4.815	0.002345	T
ENSG0000(	DCBLD1	1.112	3.6203	3.069554	0.002347	T
ENSG0000(	CYP51A1I	0.13	0.4219	2.26913	0.00235	T
ENSG0000(	RGL1	1.2975	4.1322	3.028408	0.002351	T
ENSG0000(	TGM1	272.7402	84.8901	0.311501	0.002352	N
ENSG0000(	NDUFA9P	0.2111	1.1075	3.881389	0.002353	T
ENSG0000(	FNBP1P1	0.6375	1.3919	2.022915	0.002353	T
ENSG0000(	FTX	0.8593	2.328	2.531012	0.002356	T
ENSG0000(	MICA	1.6204	3.6969	2.206987	0.002357	T
ENSG0000(	HNRNPDI	12.7195	28.0438	2.19539	0.002359	T
ENSG0000(	VPS9D1-A	1.5483	5.3689	3.317903	0.002365	T
ENSG0000(	FLVCR2	0.8453	2.554	2.807574	0.002367	T
ENSG0000(	AC253536	0.7871	2.2533	2.652801	0.00237	T
ENSG0000(	PMM1	9.4703	3.8081	0.408357	0.00237	N
ENSG0000(	CALM2	21.2638	43.4864	2.040199	0.002373	T
ENSG0000(	AC018638	0.2747	1.1384	3.305044	0.002373	T
ENSG0000(	TCIRG1	3.281	8.0241	2.402869	0.002373	T
ENSG0000(	AL390728.	1.1211	3.1231	2.639505	0.002373	T
ENSG0000(	LRRC59	9.4069	19.6617	2.078669	0.002375	T
ENSG0000(	STRADB	19.283	4.2098	0.222349	0.002376	N
ENSG0000(	TBCE	0.0753	0.3298	2.451797	0.00238	T

ENSG0000(	SNRPD1	4.1807	8.8045	2.08015	0.00238	T
ENSG0000(	BORA	1.0572	3.9929	3.536899	0.00238	T
ENSG0000(	ZP3	0.5859	1.6443	2.543082	0.002382	T
ENSG0000(	HSPA9	13.8973	32.0831	2.299236	0.002387	T
ENSG0000(	AC022211	0.6787	2.0089	2.708232	0.002391	T
ENSG0000(	AC007038	0.5176	1.7168	2.94171	0.002391	T
ENSG0000(	UPK2	1.4326	0.385	0.316456	0.002394	N
ENSG0000(	NUDT1	1.2307	4.1792	3.215751	0.002397	T
ENSG0000(	AC005785	0.1799	0.7765	3.131476	0.002401	T
ENSG0000(	FLJ13224	0.0865	0.3475	2.399464	0.002402	T
ENSG0000(	AC097263	0.408	1.3492	2.852756	0.002402	T
ENSG0000(	AL021155.	0.5268	2.4362	4.046267	0.002403	T
ENSG0000(	RPS2P45	0.0515	0.2089	2.038944	0.002411	T
ENSG0000(	HIF1A	35.8325	72.8892	2.031286	0.002413	T
ENSG0000(	DHFR2	0.6684	1.8739	2.568844	0.002415	T
ENSG0000(	FAM234B	0.6138	1.9431	2.862286	0.002417	T
ENSG0000(	RHOT1P1	11.9553	1.6853	0.148093	0.002417	N
ENSG0000(	C3orf80	3.415	0.5958	0.197952	0.002424	N
ENSG0000(	NABP2	4.2592	11.9593	2.766402	0.002424	T
ENSG0000(	AC026356	0.1818	0.8753	3.460965	0.002429	T
ENSG0000(	KLRA1P	0.2383	0.7454	2.498965	0.002433	T
ENSG0000(	PARPBP	1.4219	3.2032	2.170445	0.002434	T
ENSG0000(	AC104695	5.4399	2.3025	0.433672	0.002435	N
ENSG0000(	CHCHD2P	0.8135	3.014	3.408867	0.00244	T
ENSG0000(	PJA1	2.665	7.7054	2.822929	0.002448	T
ENSG0000(	AC106786	0.0749	0.7697	4.972556	0.002449	T
ENSG0000(	AC005537	0.0764	0.3837	2.742063	0.002456	T
ENSG0000(	CARD16	1.8259	3.7742	2.011631	0.002461	T
ENSG0000(	AC135626	0.0249	0.6141	5.717374	0.002465	T
ENSG0000(	AL118558.	0.4087	0.9475	2.05917	0.002466	T
ENSG0000(	ESPL1	11.2018	3.416	0.311101	0.002466	N
ENSG0000(	RNU6-120	0.0617	1.1939	8.001855	0.002468	T
ENSG0000(	C5orf38	0.549	1.8437	2.994915	0.002472	T
ENSG0000(	SASH1	51.0384	13.945	0.274647	0.002474	N
ENSG0000(	EIF5AP4	2.4258	5.652	2.277298	0.002477	T
ENSG0000(	NAA15	5.2237	10.5512	2.000714	0.002478	T
ENSG0000(	AC091057	0.2582	1.3371	4.012004	0.002483	T
ENSG0000(	TYMS	3.572	14.7935	4.055964	0.002483	T
ENSG0000(	RAET1L	34.9659	13.1429	0.377657	0.002485	N
ENSG0000(	AC068473	0.0281	0.568	5.214676	0.002486	T
ENSG0000(	AC012615	0.6595	1.4886	2.091639	0.002488	T
ENSG0000(	FMO9P	1.488	0.2659	0.230416	0.00249	N
ENSG0000(	AL691403.	0.0856	0.4313	2.862608	0.002491	T
ENSG0000(	RHEBL1	0.0727	0.333	2.507238	0.002492	T
ENSG0000(	GCLM	3.327	24.0914	7.05906	0.002493	T
ENSG0000(	SNORA15.	0.136	1.7821	7.975	0.002494	T
ENSG0000(	AC006077	0.6001	1.7868	2.695044	0.002494	T
ENSG0000(	PLCB1-IT1	0.1115	1.305	6.643026	0.002495	T
ENSG0000(	LINC0190!	0.0321	0.7791	6.654807	0.002496	T
ENSG0000(	CYP51A1I	0.1869	0.4992	2.088533	0.002498	T
ENSG0000(	KLF3P1	0.1754	0.6657	2.78032	0.002499	T
ENSG0000(	IL36A	832.6124	81.2029	0.097636	0.002503	N
ENSG0000(	ZNF608	1.0285	2.2931	2.120603	0.002506	T
ENSG0000(	SREK1	2.2478	5.1189	2.22289	0.002508	T
ENSG0000(	AP000662.	0.0454	0.2429	2.358322	0.002511	T
ENSG0000(	ADAR	12.6629	33.5335	2.635255	0.002512	T
ENSG0000(	HOXC-AS	0.0602	0.6023	4.383895	0.002512	T
ENSG0000(	ANKS1B	0.3784	0.0989	0.415761	0.002518	N

ENSG0000(	LINC0259:	0.4631	0.0836	0.326052	0.002523	N
ENSG0000(	LMLN	0.8437	2.055	2.283565	0.002524	T
ENSG0000(	DUS2	1.2324	2.6966	2.098919	0.002528	T
ENSG0000(	EN2	0.0104	0.2295	2.984601	0.00253	T
ENSG0000(	DDX5	24.7858	63.4115	2.552118	0.00253	T
ENSG0000(	AC040174	0.1303	0.5441	2.796787	0.002537	T
ENSG0000(	ZNF385C	0.0295	0.1773	2.141313	0.002544	T
ENSG0000(	AC009962	0.0413	0.2124	2.210899	0.002545	T
ENSG0000(	MIR196A2	0	0.3895	4.895	0.00255	T
ENSG0000(	JUN	18.9993	45.235	2.373647	0.002551	T
ENSG0000(	CREM	0.9975	3.493	3.273804	0.002552	T
ENSG0000(	FAUP1	0.2418	0.8149	2.676712	0.002556	T
ENSG0000(	RN7SKP97	0.406	2.5581	5.253162	0.002561	T
ENSG0000(	SPSB1	8.5765	17.7149	2.053236	0.002563	T
ENSG0000(	SPG7	0.7465	1.7042	2.131364	0.002568	T
ENSG0000(	FNDC3A	4.7048	9.6144	2.021812	0.00257	T
ENSG0000(	ZDHHC7	6.2991	13.354	2.102483	0.002572	T
ENSG0000(	MIR628	0	0.6611	7.611	0.002573	T
ENSG0000(	HIST1H2A	13.6163	43.7258	3.195162	0.002575	T
ENSG0000(	BICD1	0.3349	1.3003	3.219821	0.002576	T
ENSG0000(	HERC2P9	0.6066	1.4124	2.140391	0.002577	T
ENSG0000(	HSPD1P4	0.2024	0.9557	3.491071	0.002581	T
ENSG0000(	ATP6V0D2	0.0089	0.394	4.536272	0.002583	T
ENSG0000(	SNORD3B	2.8961	16.8711	5.664397	0.002585	T
ENSG0000(	BX537318	0.1801	0.6337	2.619422	0.002586	T
ENSG0000(	KRT5	751.1329	1747.955	2.326915	0.002588	T
ENSG0000(	USP42	1.9933	4.1794	2.044332	0.002588	T
ENSG0000(	ITGB4	18.3528	54.7948	2.974876	0.002591	T
ENSG0000(	MSH5	0.152	0.5385	2.53373	0.002592	T
ENSG0000(	GASAL1	0.1368	0.3858	2.05152	0.002594	T
ENSG0000(	AL356512.	0.2563	0.8578	2.688184	0.002603	T
ENSG0000(	RNU6-82P	0.742	3.9645	4.827197	0.002603	T
ENSG0000(	SFR1	1.8475	4.2268	2.22172	0.002604	T
ENSG0000(	CCDC77	1.1398	2.83	2.363284	0.002606	T
ENSG0000(	ZNF598	4.8432	10.3897	2.122046	0.002607	T
ENSG0000(	MIR4802	1.8898	5.915	3.022917	0.002613	T
ENSG0000(	SLC52A1	0.2049	1.4384	5.045589	0.002614	T
ENSG0000(	AL121652.	0.42	2.4771	4.955962	0.002616	T
ENSG0000(	POP1	1.0255	3.1953	2.927854	0.002617	T
ENSG0000(	PITX2	0.4649	2.7424	5.031687	0.002618	T
ENSG0000(	NEURL4	1.4496	3.2407	2.155847	0.002619	T
ENSG0000(	AC004865	0.0709	0.3091	2.393798	0.002619	T
ENSG0000(	AL356124.	0.1531	0.5222	2.458317	0.002621	T
ENSG0000(	HSPE1P13	0.7316	2.1328	2.684945	0.002626	T
ENSG0000(	SRP14-AS	0.1393	0.5826	2.852486	0.002629	T
ENSG0000(	KANK1	13.6592	6.6589	0.491228	0.002635	N
ENSG0000(	RNU6-987	0.0816	1.9431	11.25055	0.002635	T
ENSG0000(	AL161431.	0.5916	8.9072	13.02371	0.002636	T
ENSG0000(	LINC0134	0.0091	0.2463	3.174152	0.002637	T
ENSG0000(	TMEM243	1.7748	4.0619	2.219917	0.002637	T
ENSG0000(	RF00139	0.332	4.2374	10.04028	0.00264	T
ENSG0000(	AMDHD1	0.0516	0.2149	2.077177	0.00264	T
ENSG0000(	AL591848.	0.0441	0.2161	2.193616	0.00264	T
ENSG0000(	PLA2G7	0.3057	4.8113	12.10574	0.00264	T
ENSG0000(	CENPT	1.8218	4.2569	2.267093	0.002643	T
ENSG0000(	ITPA	4.984	11.7344	2.327773	0.002645	T
ENSG0000(	Z94721.1	0.5173	2.0116	3.420703	0.002646	T
ENSG0000(	BOC	3.4918	1.3627	0.407233	0.002647	N

ENSG0000(	MED9	0.8613	1.9403	2.122438	0.002648	T
ENSG0000(	SNORA22	0.3005	5.4203	13.78352	0.002651	T
ENSG0000(	SSSCA1	2.3909	5.4996	2.248023	0.002656	T
ENSG0000(	BMS1P13	0.4783	0.0386	0.239668	0.002663	N
ENSG0000(	AL008729.	0.1273	0.6499	3.299164	0.002667	T
ENSG0000(	CHORDC1	1.8817	5.8832	3.019226	0.002668	T
ENSG0000(	AL157834.	0.3215	1.1873	3.054093	0.002668	T
ENSG0000(	AP000526.	0	0.2951	3.951	0.00267	T
ENSG0000(	RNU6-522	0.6261	3.1453	4.469495	0.002671	T
ENSG0000(	RBBP4P1	0.4234	1.2755	2.628009	0.002678	T
ENSG0000(	TP73	1.3374	4.212	2.999861	0.002678	T
ENSG0000(	STOML1	0.279	0.7505	2.244063	0.00268	T
ENSG0000(	RF00019	0.9267	7.9787	7.868608	0.002682	T
ENSG0000(	MUC20-O	1.3743	2.8913	2.028963	0.002682	T
ENSG0000(	RP9	0.9313	2.0648	2.099098	0.002702	T
ENSG0000(	AC099506	0.0173	0.3065	3.465473	0.002704	T
ENSG0000(	CCDC106	0.8329	1.9433	2.190267	0.002711	T
ENSG0000(	AC020661	0.405	1.0095	2.19703	0.002714	T
ENSG0000(	TTC4	0.1382	0.3891	2.053317	0.002716	T
ENSG0000(	AL122001.	0.1066	0.757	4.148112	0.002717	T
ENSG0000(	ZNF195	0.7765	2.2412	2.671078	0.002721	T
ENSG0000(	RPL7P1	4.327	10.4371	2.38019	0.002722	T
ENSG0000(	TMEM44	0.499	2.335	4.065109	0.002728	T
ENSG0000(	SCGB2B2	0.0624	0.2932	2.421182	0.002729	T
ENSG0000(	RPS27AP1	0.8944	3.9896	4.112631	0.002733	T
ENSG0000(	CAPN12	0.2138	1.0095	3.535692	0.002734	T
ENSG0000(	LARGE1	0.5066	1.6274	2.847676	0.002736	T
ENSG0000(	PSMB8	4.6852	12.4994	2.632993	0.002739	T
ENSG0000(	RPL29P14	1.5065	4.0364	2.57479	0.002748	T
ENSG0000(	AC011933	0.3062	1.1117	2.983013	0.002752	T
ENSG0000(	TUT4	1.7168	3.6646	2.072105	0.002752	T
ENSG0000(	ST6GALN	3.3331	7.2715	2.147185	0.002757	T
ENSG0000(	PPIAP45	0.0377	0.4977	4.340595	0.002757	T
ENSG0000(	MSTO2P	0.2739	1.0556	3.090666	0.002758	T
ENSG0000(	AL109806.	0.0634	0.3208	2.575275	0.002761	T
ENSG0000(	AL160408.	0.2967	0.0736	0.43761	0.002761	N
ENSG0000(	LRP12	0.4357	2.4574	4.773941	0.002765	T
ENSG0000(	NAP1L4P1	0.61	2.5021	3.66493	0.002765	T
ENSG0000(	NEB	0.1141	1.2507	6.308734	0.002769	T
ENSG0000(	RBM17P4	0.0771	0.3613	2.604743	0.002774	T
ENSG0000(	CASP5	0.0389	0.5679	4.808495	0.002777	T
ENSG0000(	TNK1	1.0884	3.513	3.040222	0.002778	T
ENSG0000(	HM13-IT1	0.5898	2.3928	3.613801	0.00278	T
ENSG0000(	HSPE1P7	0.0541	0.9063	6.530175	0.00278	T
ENSG0000(	TPM3P8	0.1736	0.7969	3.278143	0.002783	T
ENSG0000(	GJA1P1	0.3535	1.506	3.541345	0.002784	T
ENSG0000(	UAP1L1	0.5484	2.3587	3.791949	0.002794	T
ENSG0000(	NCOA5	5.4364	10.9764	2.00065	0.002795	T
ENSG0000(	LAMA1	0.0377	0.237	2.447349	0.002803	T
ENSG0000(	AC138915	1.9862	0.0714	0.082159	0.002807	N
ENSG0000(	AC109322	0.3244	0.9439	2.459708	0.00281	T
ENSG0000(	AC008895	0.5006	1.216	2.191142	0.002818	T
ENSG0000(	PHF5CP	0.2668	0.8013	2.457197	0.002826	T
ENSG0000(	CDK17	3.1451	7.533	2.352162	0.00283	T
ENSG0000(	GOLIM4	2.4878	10.2829	4.01225	0.002834	T
ENSG0000(	EMC8	2.5362	5.174	2.000607	0.002837	T
ENSG0000(	AC092614	0.1533	0.7323	3.285827	0.002837	T
ENSG0000(	CD1D	0.1781	0.5311	2.269328	0.002841	T

ENSG0000(	PPIAP16	0.1528	0.4708	2.257911	0.002841	T
ENSG0000(	RPS19P3	0.8997	2.1296	2.230269	0.002846	T
ENSG0000(	DFFB	0.2993	0.7186	2.050088	0.00285	T
ENSG0000(	RCC1L	2.8632	6.1268	2.101377	0.002851	T
ENSG0000(	LRRN2	0.6406	0.1792	0.376992	0.002853	N
ENSG0000(	MIR151A	0.3358	3.1544	7.467646	0.002857	T
ENSG0000(	AL356124.	0.029	0.227	2.534884	0.002858	T
ENSG0000(	MIR4746	0.2006	1.2997	4.656354	0.00286	T
ENSG0000(	NTRK2	1.3058	13.861	9.931	0.002862	T
ENSG0000(	CHST3	1.9672	4.7935	2.367212	0.002868	T
ENSG0000(	TRIM60P1	0.4186	1.1147	2.342268	0.002869	T
ENSG0000(	DPY19L1	1.5066	5.0167	3.1848	0.002872	T
ENSG0000(	AL359881.	0.13	0.6961	3.461304	0.002874	T
ENSG0000(	AL596325.	0.6306	1.4371	2.103887	0.002874	T
ENSG0000(	UBA6-AS1	0.6516	1.5179	2.152608	0.002876	T
ENSG0000(	FN3KRP	3.4226	8.4052	2.414467	0.002878	T
ENSG0000(	ZFP41	0.3372	0.9243	2.342864	0.002887	T
ENSG0000(	SCPEP1	9.8383	24.1086	2.435889	0.002889	T
ENSG0000(	MTG2	1.4972	4.1974	2.690584	0.002897	T
ENSG0000(	TGFB2	0.4331	2.1661	4.250797	0.0029	T
ENSG0000(	ARHGEF3	0.1981	0.7115	2.722241	0.002901	T
ENSG0000(	ACTG1P3	0.2734	1.006	2.961971	0.002906	T
ENSG0000(	ASTL	0.0316	0.2961	3.009878	0.002908	T
ENSG0000(	AL137077.	0.0796	0.2754	2.0902	0.002909	T
ENSG0000(	BARD1	4.9386	10.0263	2.009745	0.002915	T
ENSG0000(	CTSZ	9.5251	33.4305	3.483652	0.002917	T
ENSG0000(	NTF4	0.5492	1.4804	2.434381	0.002917	T
ENSG0000(	RNU6-111	0.1036	1.2361	6.562377	0.00292	T
ENSG0000(	MKKS	1.2669	3.1972	2.412174	0.00292	T
ENSG0000(	WDR81	1.2425	3.2322	2.482086	0.00292	T
ENSG0000(	TAF1C	1.7589	4.6396	2.54968	0.00292	T
ENSG0000(	PATZ1	0.9938	2.4962	2.37356	0.002924	T
ENSG0000(	SETP21	0.0631	0.4323	3.263642	0.002928	T
ENSG0000(	MAGEF1	7.0031	24.0582	3.401078	0.002929	T
ENSG0000(	PTRH1	0.4944	1.1982	2.184051	0.002931	T
ENSG0000(	POU5F1P5	0.4261	1.145	2.36647	0.002939	T
ENSG0000(	AC126768	0.434	1.7	3.370787	0.002942	T
ENSG0000(	SREBF1	7.2983	16.6213	2.260154	0.002943	T
ENSG0000(	APOBEC3	0.7417	2.4058	2.97707	0.002949	T
ENSG0000(	AL121761.	35.3234	3.6838	0.106816	0.002949	N
ENSG0000(	AC073439	0.0647	0.5091	3.698239	0.002951	T
ENSG0000(	SNORD56.	1.669	7.9886	4.572414	0.002952	T
ENSG0000(	KHDC1	0.1176	0.3745	2.180607	0.002952	T
ENSG0000(	C21orf58	0.3712	0.9875	2.307937	0.002953	T
ENSG0000(	TEDC1	0.227	1.0702	3.578593	0.002955	T
ENSG0000(	HNRNPA1	1.1678	2.9429	2.400142	0.002958	T
ENSG0000(	AC009061	0.1701	0.5235	2.308404	0.00296	T
ENSG0000(	HDGFL3	1.0222	2.1978	2.047585	0.002961	T
ENSG0000(	STK17B	5.4026	13.1945	2.41604	0.002962	T
ENSG0000(	AL049795.	0.1805	0.6838	2.794296	0.002966	T
ENSG0000(	RPL5P18	0.2444	0.6526	2.18525	0.002968	T
ENSG0000(	EEF1A1P1	0.3055	0.8347	2.305055	0.002975	T
ENSG0000(	AC009303	0.2039	1.1435	4.091807	0.002976	T
ENSG0000(	PPP1R3B	8.4213	3.1685	0.383568	0.002977	N
ENSG0000(	ANKRD36	0.6891	2.4745	3.262578	0.002978	T
ENSG0000(	FKBP14	1.8403	4.2837	2.25929	0.002979	T
ENSG0000(	AC010735	0.0388	0.4761	4.150576	0.002981	T
ENSG0000(	AC108860	0.1643	0.4777	2.185774	0.002983	T

ENSG0000(	RF00019	0.167	2.0422	8.023221	0.002985	T
ENSG0000(	NCOA7	5.6773	33.7027	5.850951	0.002993	T
ENSG0000(	RNU6-789	0.9705	3.0134	2.908361	0.002995	T
ENSG0000(	SUMO4	0.3769	1.047	2.405116	0.003013	T
ENSG0000(	TOMM70	10.9625	23.7425	2.155254	0.003018	T
ENSG0000(	LINC0159.	0.0047	0.5128	5.852913	0.00302	T
ENSG0000(	AC096772	0.4067	1.1241	2.415828	0.00302	T
ENSG0000(	PLTP	4.6419	24.7791	5.246652	0.003021	T
ENSG0000(	AL133330.	0.9776	3.7682	3.589644	0.003024	T
ENSG0000(	HOXC-AS	0.0075	0.2307	3.076279	0.003029	T
ENSG0000(	DUSP2	1.3505	7.8085	5.452258	0.00303	T
ENSG0000(	CLEC11A	0.8726	2.0777	2.23905	0.003031	T
ENSG0000(	GTF2IP20	0.9167	1.9348	2.001377	0.003031	T
ENSG0000(	IL18BP	0.6187	2.0981	3.058439	0.003034	T
ENSG0000(	ARF1P2	0.6709	1.9401	2.646387	0.003035	T
ENSG0000(	PDP1	8.8167	20.2938	2.287147	0.003036	T
ENSG0000(	WRN	2.0625	4.6156	2.180624	0.003039	T
ENSG0000(	MIGA1	1.3644	2.8992	2.048074	0.003045	T
ENSG0000(	AC013394	0.3512	1.3638	3.244238	0.003046	T
ENSG0000(	TMEM41A	3.7797	8.0856	2.109854	0.003052	T
ENSG0000(	UNG	7.4349	15.3886	2.055581	0.003061	T
ENSG0000(	STXBP5-A	1.1986	0.3843	0.37294	0.003072	N
ENSG0000(	VRK2	7.5738	15.3326	2.011077	0.003072	T
ENSG0000(	GABRE	2.6479	15.2523	5.586921	0.003075	T
ENSG0000(	KRT86	0.0244	0.1756	2.215434	0.003075	T
ENSG0000(	DNAJC19I	0.2416	0.9926	3.198478	0.003076	T
ENSG0000(	TAS2R15F	0.3228	1.792	4.474929	0.003078	T
ENSG0000(	AC079416	0.2217	0.8718	3.020827	0.003081	T
ENSG0000(	HOXD13	0	1.0293	11.293	0.003081	T
ENSG0000(	AP000347.	0.0845	0.2794	2.056369	0.003081	T
ENSG0000(	AF212831.	0.0481	1.3286	9.646185	0.003085	T
ENSG0000(	SMYD3	0.6005	2.3257	3.462812	0.003089	T
ENSG0000(	NOS1AP	0.1015	0.6784	3.863027	0.003094	T
ENSG0000(	SEC14L2	3.838	1.3589	0.370467	0.003099	N
ENSG0000(	AC027279	0.1733	0.614	2.612514	0.003105	T
ENSG0000(	SNHG4	0.9125	2.4704	2.538667	0.003106	T
ENSG0000(	AL671277.	0.0926	0.2885	2.017134	0.003107	T
ENSG0000(	AC023906	0.1027	0.608	3.492847	0.003109	T
ENSG0000(	AC013652	0.1182	1.5477	7.551329	0.003112	T
ENSG0000(	RASA2-IT	0.436	1.8663	3.66847	0.003113	T
ENSG0000(	UBE2V2P.	0.3695	0.8814	2.090309	0.003113	T
ENSG0000(	AL356432.	0.1304	0.4297	2.299045	0.003113	T
ENSG0000(	TNFAIP8	2.4418	5.1785	2.076678	0.003115	T
ENSG0000(	DUSP6	3.7617	13.9757	3.644949	0.003121	T
ENSG0000(	HGH1	1.95	4.9493	2.463073	0.003124	T
ENSG0000(	AC008537	0.0421	0.442	3.814215	0.003124	T
ENSG0000(	GSDME	0.2844	0.8393	2.443548	0.003126	T
ENSG0000(	AL080276.	0.2087	0.6054	2.285066	0.003126	T
ENSG0000(	TFAP2C	11.0589	22.7132	2.044395	0.003127	T
ENSG0000(	MAPRE3	1.3344	3.4699	2.488776	0.003127	T
ENSG0000(	MRPL35P.	0.2334	1.1843	3.85213	0.003129	T
ENSG0000(	AL450384.	0.2764	0.9147	2.695802	0.003136	T
ENSG0000(	AL445524.	3.2199	7.5201	2.29528	0.003136	T
ENSG0000(	AC009502	0.0092	0.2372	3.087912	0.00314	T
ENSG0000(	CBWD5	0.4472	1.2888	2.538012	0.003141	T
ENSG0000(	SEMA6D	0.1859	0.5365	2.226303	0.003141	T
ENSG0000(	WNT5B	2.1279	0.8565	0.429328	0.003143	N
ENSG0000(	CHSY1	2.8216	8.9825	3.108742	0.003144	T

ENSG0000(	SNORA11	1.7187	4.8973	2.747732	0.003152	T
ENSG0000(	CLDN7	70.0269	34.7941	0.497585	0.003153	N
ENSG0000(	ALDH5A1	2.1821	4.9405	2.208711	0.003156	T
ENSG0000(	SDCBP2-A	0.2024	0.7451	2.794643	0.003173	T
ENSG0000(	SOAT1	1.2221	3.9583	3.069586	0.003181	T
ENSG0000(	ITGA6-AS	0.0297	0.184	2.189668	0.003182	T
ENSG0000(	AL445437.	0.0198	0.7494	7.09015	0.003184	T
ENSG0000(	G3BP1	9.2265	18.7362	2.019643	0.003185	T
ENSG0000(	AC008957	0.1409	0.786	3.677875	0.003185	T
ENSG0000(	NDUFAF8	3.6953	9.966	2.652228	0.003187	T
ENSG0000(	PPP1R3G	0.6981	0.2145	0.394061	0.003189	N
ENSG0000(	COQ7	2.1547	4.4401	2.013616	0.003194	T
ENSG0000(	NDUFB2-A	0.2361	0.5886	2.048795	0.003196	T
ENSG0000(	AL391994.	0.355	1.1681	2.787033	0.003201	T
ENSG0000(	AC234775	0.1862	0.5816	2.381551	0.003207	T
ENSG0000(	AL158152.	1.2742	3.1591	2.371634	0.003209	T
ENSG0000(	AC067930	0.4962	0.1376	0.398524	0.003214	N
ENSG0000(	TRIO	2.1379	6.5073	2.952455	0.003217	T
ENSG0000(	SLC38A6	0.5611	2.4591	3.870973	0.003222	T
ENSG0000(	ZNF542P	0.8916	0.301	0.404397	0.003224	N
ENSG0000(	HAP1	0.089	1.394	7.904762	0.003224	T
ENSG0000(	SCLY	0.0786	0.2602	2.016797	0.003224	T
ENSG0000(	TOMM34	4.4714	10.5398	2.327471	0.003229	T
ENSG0000(	AC087741	0.1805	0.5396	2.280214	0.003231	T
ENSG0000(	ST20	0.3874	1.4742	3.229791	0.003232	T
ENSG0000(	SYT14	0.0017	0.3471	4.396264	0.003241	T
ENSG0000(	NIPSNAP2	4.6547	14.8294	3.139925	0.003243	T
ENSG0000(	MIR3144	0	4.481	45.81	0.003248	T
ENSG0000(	TRAF3	2.4197	5.8481	2.360638	0.00325	T
ENSG0000(	AGPAT4	0.6721	1.4489	2.006087	0.003251	T
ENSG0000(	NAT14	0.3675	1.4681	3.354225	0.003255	T
ENSG0000(	RNU6-1	3.3762	11.8179	3.428428	0.003257	T
ENSG0000(	TMPRSS1	407.6095	33.8332	0.083229	0.003262	N
ENSG0000(	AC025458	1.4006	3.495	2.395708	0.003263	T
ENSG0000(	AP002807.	0.4886	1.2177	2.238702	0.003264	T
ENSG0000(	TM4SF19	0.3093	1.6677	4.318837	0.003266	T
ENSG0000(	GATA4	0	0.3985	4.985	0.003267	T
ENSG0000(	CCT2	11.7331	26.5287	2.250357	0.003272	T
ENSG0000(	FGF14	0.2277	0.0497	0.45682	0.003277	N
ENSG0000(	AC097626	0.126	0.4758	2.547788	0.003285	T
ENSG0000(	AL021707.	0.6182	2.638	3.812309	0.003286	T
ENSG0000(	FAM83D	55.1324	26.6659	0.484605	0.003291	N
ENSG0000(	APH1B	0.5319	1.1667	2.004589	0.003292	T
ENSG0000(	AC078845	0.0563	0.2316	2.121561	0.003295	T
ENSG0000(	PPP2R5B	1.8494	5.2538	2.746384	0.003301	T
ENSG0000(	ANKK1	0.0719	0.5739	3.920303	0.003304	T
ENSG0000(	AC106782	0.2971	1.1945	3.259884	0.003304	T
ENSG0000(	AC092747	0.1361	0.4977	2.531554	0.003305	T
ENSG0000(	RCN1	1.4539	4.3897	2.889311	0.003305	T
ENSG0000(	RABL2A	0.4907	1.1953	2.192822	0.003305	T
ENSG0000(	PVR	2.5574	8.0614	3.071197	0.003307	T
ENSG0000(	MIR590	3.1259	8.8901	2.78685	0.003312	T
ENSG0000(	IL18	133.1211	20.8547	0.157293	0.003313	N
ENSG0000(	ATXN7L2	0.4452	1.0894	2.181585	0.003314	T
ENSG0000(	DMRT2	0.0588	1.1447	7.838161	0.003329	T
ENSG0000(	RNU4-51P	0.2008	3.4382	11.76263	0.003332	T
ENSG0000(	AC019211	0.2043	0.5727	2.210647	0.003336	T
ENSG0000(	AHR	4.2347	15.5794	3.617182	0.003338	T

ENSG0000(	ZNF205	1.4392	3.1311	2.099207	0.00334	T
ENSG0000(	HIST1H2B	16.8135	47.9734	2.842309	0.003342	T
ENSG0000(	AC068282	0.1605	0.4388	2.06833	0.003346	T
ENSG0000(	ZNF37BP	0.4978	1.1207	2.041987	0.003346	T
ENSG0000(	RN7SL192	0.1967	1.0295	3.806876	0.003347	T
ENSG0000(	PCAT1	0.0962	0.4874	2.993884	0.003349	T
ENSG0000(	AC022306	0.0613	0.2523	2.184129	0.003351	T
ENSG0000(	RPL7P14	0.1829	0.8572	3.383528	0.003353	T
ENSG0000(	ESAM	15.5775	2.0883	0.139582	0.003354	N
ENSG0000(	DDX51	0.7635	1.6355	2.009844	0.003357	T
ENSG0000(	AL137918.	0.0281	0.4645	4.406714	0.003359	T
ENSG0000(	TAP2	2.357	5.7396	2.37672	0.00336	T
ENSG0000(	ZBTB25	0.7098	1.6889	2.209064	0.003364	T
ENSG0000(	RNF169	26.3874	9.673	0.368968	0.003365	N
ENSG0000(	XPO6	5.8808	14.9884	2.522806	0.003368	T
ENSG0000(	RF00019	0.12	2.1686	10.31182	0.003372	T
ENSG0000(	TYK2	2.798	6.3182	2.2147	0.003373	T
ENSG0000(	TIPARP-A	0.1672	0.6331	2.743638	0.003383	T
ENSG0000(	AHCYL2	3.4647	7.1092	2.022386	0.003383	T
ENSG0000(	PRR34-AS	2.7769	6.4007	2.25962	0.003385	T
ENSG0000(	C9orf152	1.3638	0.2587	0.245047	0.003391	N
ENSG0000(	PHLDA1	65.735	26.1408	0.398584	0.003393	N
ENSG0000(	ZNF26	0.2953	0.7157	2.063496	0.003395	T
ENSG0000(	C5orf66-A	36.3275	11.0505	0.306101	0.003396	N
ENSG0000(	PI15	0.4003	1.4007	2.9996	0.003397	T
ENSG0000(	CEACAM1	48.3322	18.656	0.387263	0.003401	N
ENSG0000(	LINC0193	0.0967	0.8612	4.886629	0.003401	T
ENSG0000(	HIST2H3A	0.086	0.7027	4.315591	0.003402	T
ENSG0000(	LENG8-AS	0.9698	2.6203	2.542812	0.003402	T
ENSG0000(	CYP2D6	0.1735	0.5326	2.31298	0.003406	T
ENSG0000(	ZNF707	0.5227	1.4738	2.527381	0.003412	T
ENSG0000(	GVQW2	0.1006	0.3326	2.15653	0.003417	T
ENSG0000(	BAZ1A	7.955	17.8248	2.225301	0.003418	T
ENSG0000(	SNCA	0.6199	2.0767	3.023614	0.003423	T
ENSG0000(	FDPSP3	0.0791	0.4379	3.00335	0.003426	T
ENSG0000(	AL138902.	0.0855	0.716	4.398922	0.003428	T
ENSG0000(	MSH6	1.4812	6.2859	4.038642	0.003433	T
ENSG0000(	LRRCC1	1.2117	3.1941	2.511321	0.003437	T
ENSG0000(	SLC5A12	0.0029	0.6899	7.676385	0.003437	T
ENSG0000(	ZNF518B	1.7114	4.7185	2.660097	0.003443	T
ENSG0000(	SLC51A	0.0168	0.3388	3.756849	0.003443	T
ENSG0000(	RCBTB1	1.1427	2.839	2.365012	0.003446	T
ENSG0000(	IGFLR1	0.0794	0.2774	2.103679	0.003451	T
ENSG0000(	ADGRV1	0.1139	0.5984	3.265077	0.003452	T
ENSG0000(	CTNS	1.4373	3.9688	2.646718	0.003453	T
ENSG0000(	RF00004	1.8803	6.5329	3.349442	0.003457	T
ENSG0000(	RNU6-101	4.4179	13.3829	2.984329	0.003459	T
ENSG0000(	DYNC2H1	0.8872	10.7105	10.95067	0.00346	T
ENSG0000(	AC009716	0.1009	0.3472	2.225983	0.003465	T
ENSG0000(	AP5S1	0.9073	2.2775	2.36027	0.003466	T
ENSG0000(	PTPN6	2.136	6.1104	2.77746	0.003467	T
ENSG0000(	AL645568.	0.0346	0.2093	2.29792	0.003473	T
ENSG0000(	NMRAL2I	0.609	8.3113	11.86361	0.003475	T
ENSG0000(	LINC0112(	0.2792	0.8112	2.402954	0.003486	T
ENSG0000(	APOL2	2.0808	7.6212	3.540536	0.003488	T
ENSG0000(	MIR3685	1.8351	9.3675	4.892512	0.003492	T
ENSG0000(	TUBGCP3	2.2259	5.4099	2.368932	0.003501	T
ENSG0000(	AL512306.	0.0293	0.2635	2.811292	0.003502	T

ENSG0000(	ADRB2	2.9219	7.2615	2.43605	0.003503	T
ENSG0000(	GAP43	0.0127	0.4925	5.25732	0.003503	T
ENSG0000(	AP002495.	0.3678	1.4281	3.266567	0.003506	T
ENSG0000(	CHODL	0.1923	1.7932	6.476907	0.00351	T
ENSG0000(	AGAP6	0.9609	2.564	2.511076	0.003512	T
ENSG0000(	EXOSC4	4.338	9.8846	2.249797	0.003514	T
ENSG0000(	AC090286	0.3181	1.5585	3.966754	0.003517	T
ENSG0000(	TM4SF4	0.2969	1.3308	3.604938	0.003518	T
ENSG0000(	AC008393	0.1247	0.3775	2.125056	0.003534	T
ENSG0000(	CAHM	0.1534	0.5758	2.66693	0.003538	T
ENSG0000(	AL359541.	0.0919	0.3729	2.464304	0.00354	T
ENSG0000(	AL391361.	6.8421	0.9844	0.156206	0.003543	N
ENSG0000(	ASH1L-AS	0.5953	1.7593	2.674098	0.003546	T
ENSG0000(	ENO1P3	0.1073	0.4004	2.413893	0.003547	T
ENSG0000(	SNORD3B	4.0675	18.2724	4.408494	0.00355	T
ENSG0000(	AC078883	0.3064	1.1109	2.979577	0.00355	T
ENSG0000(	AC105460	0	0.3879	4.879	0.003558	T
ENSG0000(	LINC0173(	0.0309	0.3678	3.57372	0.003561	T
ENSG0000(	CTTNBP2	7.6966	2.6462	0.35223	0.003562	N
ENSG0000(	CRISP3	557.027	17.687	0.031926	0.003563	N
ENSG0000(	BRD9P2	0.2642	2.9718	8.434377	0.003564	T
ENSG0000(	AL023875.	0.0169	0.2032	2.59367	0.003566	T
ENSG0000(	AC020891	0.1724	0.8843	3.613436	0.00357	T
ENSG0000(	PSMA6	0.5183	1.3896	2.409186	0.003577	T
ENSG0000(	KCNQ1OT	0.1337	0.3829	2.066324	0.003584	T
ENSG0000(	AC090518	0.0197	0.1945	2.460317	0.003585	T
ENSG0000(	AC132938	0.0535	0.2345	2.179153	0.003588	T
ENSG0000(	AC097347	0.0361	1.8103	14.036	0.003592	T
ENSG0000(	AC009075	0.0408	0.7405	5.96946	0.0036	T
ENSG0000(	KRT18P31	0.5253	1.313	2.259715	0.003601	T
ENSG0000(	CKS1BP7	0.2177	1.5019	5.042178	0.003606	T
ENSG0000(	AL021807.	0.2273	1.093	3.644974	0.003606	T
ENSG0000(	RNA5SP38	0.774	2.1582	2.583753	0.003606	T
ENSG0000(	ZFP90	1.3833	2.8959	2.019753	0.003609	T
ENSG0000(	ATG2A	2.5425	6.1346	2.359357	0.003609	T
ENSG0000(	MARCKSI	4.6689	21.5636	4.542683	0.003612	T
ENSG0000(	DZIP3	0.9286	2.263	2.297297	0.003615	T
ENSG0000(	FOXK1	4.3326	9.6918	2.209042	0.003616	T
ENSG0000(	PRR22	0.3198	0.9874	2.590281	0.003619	T
ENSG0000(	AC104763	0.2691	1.4055	4.07884	0.003623	T
ENSG0000(	BCL11A	1.515	3.4448	2.194923	0.003623	T
ENSG0000(	AC145098	0.0327	0.1727	2.055011	0.003631	T
ENSG0000(	DUXAP10	0.0053	0.2598	3.416904	0.003633	T
ENSG0000(	MIR623	1.2263	4.8408	3.725251	0.003639	T
ENSG0000(	RAC2	3.8353	13.1944	3.378243	0.003639	T
ENSG0000(	AC079601	0.104	0.5946	3.404902	0.003639	T
ENSG0000(	COTL1	2.8773	10.6243	3.602022	0.003642	T
ENSG0000(	CDK2	3.3369	9.9749	2.931392	0.003658	T
ENSG0000(	SIVA1	1.3219	3.883	2.801182	0.003658	T
ENSG0000(	AL606490.	0.0239	0.2486	2.813559	0.003666	T
ENSG0000(	AC015883	0.0896	0.5536	3.447257	0.003668	T
ENSG0000(	ATP6V0A4	4.3582	1.1753	0.286057	0.003675	N
ENSG0000(	RN7SKP18	13.6386	37.6507	2.747784	0.003682	T
ENSG0000(	SYCP2	0.1728	1.0226	4.115103	0.003687	T
ENSG0000(	PRRT3	1.5287	0.4312	0.32615	0.003688	N
ENSG0000(	SNORD10(	4.0302	10.8613	2.653939	0.003691	T
ENSG0000(	AC022007	1.3313	0.1713	0.189548	0.003693	N
ENSG0000(	RNU6-100	0.7334	2.9324	3.638589	0.003701	T

ENSG0000(	TBC1D30	0.0925	0.5429	3.33974	0.003706	T
ENSG0000(	SS18L1	2.4879	5.635	2.216083	0.003706	T
ENSG0000(	CDCA4P4	0.4617	1.8932	3.548513	0.003717	T
ENSG0000(	PPAT	1.2121	4.7624	3.705815	0.003717	T
ENSG0000(	H1FX	24.8548	63.8863	2.564088	0.003718	T
ENSG0000(	LINC0161!	0.061	0.7826	5.481988	0.003719	T
ENSG0000(	RPS6KA2-	0.09	0.5694	3.523158	0.00372	T
ENSG0000(	GEMIN8P4	0.4315	1.2228	2.488805	0.003724	T
ENSG0000(	AC116913	0.1342	0.4369	2.292485	0.003726	T
ENSG0000(	AEN	1.5663	4.2215	2.593471	0.003732	T
ENSG0000(	C2orf48	0.0513	0.7622	5.698612	0.003736	T
ENSG0000(	RPL7P47	0.558	1.3388	2.186626	0.00374	T
ENSG0000(	AC133134	4.4203	9.2506	2.06858	0.003741	T
ENSG0000(	RF00019	6.3236	23.0298	3.600753	0.003742	T
ENSG0000(	RBP1	1.4797	8.6211	5.520732	0.003748	T
ENSG0000(	RAB3IP	0.806	2.3009	2.65	0.003751	T
ENSG0000(	STEAP3	3.4764	7.9989	2.26454	0.003753	T
ENSG0000(	HSP90B3P	0.0409	0.2867	2.7445	0.003755	T
ENSG0000(	HMGB1P2	0.0328	0.6064	5.319277	0.003756	T
ENSG0000(	GBP6	377.9048	162.2502	0.429492	0.003757	N
ENSG0000(	HUS1B	0.1141	0.3849	2.26483	0.003764	T
ENSG0000(	FLVCR1-I	0.1109	0.4643	2.675676	0.003765	T
ENSG0000(	RNU7-151	0.0596	1.2669	8.564536	0.003768	T
ENSG0000(	AL360271.	0.0419	0.2606	2.541226	0.003774	T
ENSG0000(	RNA5SP1(	0.3902	1.729	3.73113	0.003778	T
ENSG0000(	PGAM1P7	0.1206	0.5917	3.135539	0.003783	T
ENSG0000(	KRT16P6	1.3518	7.8955	5.507301	0.00379	T
ENSG0000(	RINL	0.5535	1.7286	2.798164	0.003791	T
ENSG0000(	PEG13	0.1128	0.3562	2.143797	0.003799	T
ENSG0000(	PACRGL	1.2633	2.7641	2.100858	0.003799	T
ENSG0000(	CEP128	0.4564	1.6527	3.150072	0.003803	T
ENSG0000(	SLC16A6	8.718	3.6759	0.428204	0.003807	N
ENSG0000(	ZNF689	0.8701	1.9056	2.067416	0.003812	T
ENSG0000(	GPR137C	0.0452	0.2032	2.088154	0.003818	T
ENSG0000(	TFAP2A-A	0.0635	0.2642	2.227523	0.003821	T
ENSG0000(	HOXC8	0.1228	0.4205	2.336176	0.003822	T
ENSG0000(	SNHG11	0.7082	1.8132	2.367236	0.003828	T
ENSG0000(	PPP2R3A	4.3077	8.8556	2.031808	0.003832	T
ENSG0000(	ECE1	3.2338	15.4944	4.677665	0.003832	T
ENSG0000(	SALL4	0.0496	0.4274	3.525401	0.003834	T
ENSG0000(	RF00019	0.7042	3.2265	4.136409	0.003835	T
ENSG0000(	RNU6-3P	5.9083	18.0458	3.020122	0.003849	T
ENSG0000(	AC138305	1.0797	3.2113	2.8069	0.003852	T
ENSG0000(	SNX18	4.8315	12.1475	2.483524	0.003852	T
ENSG0000(	ARHGAP8	0.1672	0.6436	2.782934	0.003857	T
ENSG0000(	LYSMD2	1.6503	3.5749	2.099583	0.00386	T
ENSG0000(	THEM4	0.597	2.8394	4.217217	0.003861	T
ENSG0000(	RNU4ATA	0.5415	2.2944	3.732502	0.003862	T
ENSG0000(	TRMT61A	1.6505	4.2592	2.49026	0.003863	T
ENSG0000(	ATM	1.5231	3.2008	2.033639	0.003866	T
ENSG0000(	SP100	2.0994	5.9597	2.75516	0.003867	T
ENSG0000(	AC091057	0.3115	1.3487	3.520535	0.003868	T
ENSG0000(	IFT88	0.7779	1.6825	2.030413	0.003868	T
ENSG0000(	HMGB3P4	0.3067	0.9691	2.628719	0.003874	T
ENSG0000(	AC005339	0.2208	1.0093	3.457918	0.003878	T
ENSG0000(	CD70	0.0255	0.231	2.63745	0.003879	T
ENSG0000(	AC100782	0	0.3711	4.711	0.003895	T
ENSG0000(	DTX4	0.9038	3.7553	3.840705	0.003897	T

ENSG0000(	CRCT1	2652.112	246.8785	0.093122	0.003902	N
ENSG0000(	SNORD13	17.8385	89.9772	5.021445	0.003905	T
ENSG0000(	MRPS23	1.3682	3.0313	2.132748	0.003905	T
ENSG0000(	AL021707.	0.1922	0.8444	3.232033	0.003905	T
ENSG0000(	AF038458.	0.0189	0.3671	3.928511	0.003909	T
ENSG0000(	AC013468	0.0325	0.2744	2.82566	0.003917	T
ENSG0000(	ANKRD10	2.9036	9.9537	3.347217	0.003926	T
ENSG0000(	TSNARE1	1.4176	4.5118	3.038877	0.003932	T
ENSG0000(	AC015982	0.8082	2.1791	2.509469	0.003938	T
ENSG0000(	AC005674	0.5037	1.6084	2.829882	0.003956	T
ENSG0000(	AC007566	0.6391	1.7792	2.542552	0.003957	T
ENSG0000(	AC073548	0.3222	0.7659	2.050924	0.003961	T
ENSG0000(	AC121764	4.3976	1.1204	0.271345	0.003966	N
ENSG0000(	SNORD4A	2.6648	8.9025	3.256113	0.003971	T
ENSG0000(	PDE10A	0.075	0.5101	3.486286	0.003976	T
ENSG0000(	LINC0125	0.0558	0.2648	2.341463	0.003977	T
ENSG0000(	BRWD1-IT	0.16	0.8036	3.475385	0.003979	T
ENSG0000(	GNA12	1.7447	6.2074	3.419201	0.00398	T
ENSG0000(	PAPPA	0.3849	1.5097	3.319654	0.00398	T
ENSG0000(	MIR301A	1.1251	7.094	5.872174	0.003982	T
ENSG0000(	DLX6	0.0043	0.3111	3.941515	0.003986	T
ENSG0000(	FAM71A	0.0149	0.2911	3.403829	0.00399	T
ENSG0000(	WNT3A	0.405	1.5664	3.299802	0.003993	T
ENSG0000(	AL731559.	0.0948	0.4733	2.943018	0.003995	T
ENSG0000(	RPL7P23	4.0838	8.7862	2.123954	0.004	T
ENSG0000(	SLX4IP	2.2524	5.0103	2.172377	0.004001	T
ENSG0000(	PER2	7.7648	21.4595	2.741265	0.004003	T
ENSG0000(	ORAI1	2.4789	6.5045	2.560976	0.004014	T
ENSG0000(	SCARNA1	1.1701	4.1206	3.323045	0.004014	T
ENSG0000(	CRLF1	0.3583	1.1819	2.797076	0.004021	T
ENSG0000(	LINC0083	0.3781	2.1462	4.69818	0.004027	T
ENSG0000(	RPL9P16	0.0658	0.3764	2.873341	0.00403	T
ENSG0000(	GPR34	1.2772	3.7215	2.774833	0.004044	T
ENSG0000(	AC242376	0.2538	1.0458	3.238553	0.004045	T
ENSG0000(	DLX5	1.7195	4.3978	2.471998	0.004045	T
ENSG0000(	HTR3B	0.5744	0.0868	0.276987	0.004049	N
ENSG0000(	AC096720	0.1095	0.3759	2.271599	0.004051	T
ENSG0000(	XPOT	8.6925	18.6406	2.13143	0.004055	T
ENSG0000(	ZDHHC17	1.5331	3.4899	2.198212	0.004056	T
ENSG0000(	GRAMD1/	1.2052	4.2979	3.369522	0.00407	T
ENSG0000(	AL596087.	0.1972	0.716	2.745626	0.004075	T
ENSG0000(	IL7R	1.9146	7.6357	3.839819	0.004078	T
ENSG0000(	AKR1B15	0.1752	3.4812	13.01308	0.004083	T
ENSG0000(	AHCTF1P.	0.0432	0.2819	2.666899	0.00409	T
ENSG0000(	SPECC1	4.3857	1.5653	0.371246	0.004091	N
ENSG0000(	KC6	0.0575	2.2002	14.60444	0.004092	T
ENSG0000(	SCARNA2	0.3456	1.2007	2.918986	0.004104	T
ENSG0000(	SPINK7	535.3756	38.5658	0.072208	0.004104	N
ENSG0000(	AP002026.	0.3491	0.9258	2.284124	0.004107	T
ENSG0000(	DUSP5	191.4079	48.0949	0.25166	0.004108	N
ENSG0000(	RN7SKP4	0.4359	1.3559	2.716738	0.004113	T
ENSG0000(	AL096701.	0.0365	0.1968	2.174359	0.004113	T
ENSG0000(	AL122023.	1.5512	0.4642	0.341691	0.004121	N
ENSG0000(	KIAA0895	1.089	2.4164	2.1164	0.004121	T
ENSG0000(	AL133367.	0.1335	0.3949	2.119486	0.004121	T
ENSG0000(	LMO2	5.2697	1.8966	0.371827	0.004137	N
ENSG0000(	AC112484	0.1467	0.4497	2.228212	0.004138	T
ENSG0000(	Z83840.1	0.9496	2.7232	2.689787	0.00414	T

ENSG0000(	KY	0.0476	0.2465	2.347561	0.004141	T
ENSG0000(	KCNAB1	0.538	0.1777	0.435266	0.004142	N
ENSG0000(	RNU5B-1	11.8924	38.2283	3.196049	0.004143	T
ENSG0000(	AC018616	0.02	1.1301	10.25083	0.004144	T
ENSG0000(	TTYH1	0.5258	0.0762	0.28156	0.004146	N
ENSG0000(	AC004816	38.6908	8.8024	0.229498	0.004146	N
ENSG0000(	CYCSP24	0.1474	0.6671	3.100647	0.004154	T
ENSG0000(	ADCY10P	0.3024	1.0751	2.920229	0.004159	T
ENSG0000(	AC060780	0.3576	0.9412	2.27535	0.004169	T
ENSG0000(	KLHDC7A	0.9394	0.3036	0.388301	0.00417	N
ENSG0000(	AC025048	0.2126	1.2931	4.456494	0.004171	T
ENSG0000(	BREA2	0.0697	0.3181	2.46376	0.004179	T
ENSG0000(	MS4A14	0.0745	0.3247	2.433811	0.004179	T
ENSG0000(	AL162742.	0.0198	0.2177	2.65192	0.004183	T
ENSG0000(	AC118549	3.217	6.5523	2.005517	0.004183	T
ENSG0000(	SLC5A3	2.2368	5.8872	2.562136	0.004189	T
ENSG0000(	MCF2L	0.5924	1.3275	2.06167	0.004193	T
ENSG0000(	ZC3H11B	0.2853	0.7183	2.1238	0.004194	T
ENSG0000(	RN7SL648	1.3872	5.6159	3.843397	0.004198	T
ENSG0000(	PCP4L1	5.8665	29.0762	4.890003	0.004203	T
ENSG0000(	FABP5	264.0276	733.6145	2.777879	0.004206	T
ENSG0000(	PFDN2	10.5867	25.3903	2.385236	0.004206	T
ENSG0000(	CSGALNA	2.7786	6.3445	2.238762	0.004214	T
ENSG0000(	MYDGF	6.4876	13.1091	2.005146	0.004217	T
ENSG0000(	AC109326	1.1884	6.017	4.747749	0.004218	T
ENSG0000(	AC090527	0.1136	0.3866	2.27809	0.004229	T
ENSG0000(	NAT10	4.0585	8.2868	2.016785	0.004231	T
ENSG0000(	TNFRSF25	1.0621	2.24	2.013596	0.004238	T
ENSG0000(	AC018716	0.0038	0.7193	7.893064	0.004239	T
ENSG0000(	RF00402	0.7049	5.6916	7.195428	0.004249	T
ENSG0000(	SMPD4P1	0.0143	0.7837	7.731409	0.004254	T
ENSG0000(	CHAMP1	1.918	4.0215	2.042369	0.004256	T
ENSG0000(	AC007240	0.0434	0.3723	3.293584	0.004259	T
ENSG0000(	AL353795.	0.153	0.5318	2.497233	0.004259	T
ENSG0000(	SECISBP2	19.9983	6.8019	0.343407	0.004261	N
ENSG0000(	EYA4	0.0182	0.5077	5.141286	0.004263	T
ENSG0000(	AL513175.	0.2534	1.0204	3.170345	0.004264	T
ENSG0000(	LINC0163	0	0.2143	3.143	0.004265	T
ENSG0000(	SLAMF9	0.0406	0.6662	5.449502	0.004266	T
ENSG0000(	BUD23	1.9101	4.6258	2.351027	0.004267	T
ENSG0000(	RF00019	0.1004	0.7226	4.10479	0.004268	T
ENSG0000(	AC002075	0	0.7724	8.724	0.004268	T
ENSG0000(	SGTB	0.8187	1.9133	2.191466	0.00427	T
ENSG0000(	AC091152	0.1402	0.4845	2.433389	0.004277	T
ENSG0000(	ICK	5.2641	14.9451	2.804776	0.004277	T
ENSG0000(	CPT1C	0.1037	0.3463	2.190967	0.004279	T
ENSG0000(	AL137003.	0.5445	1.6395	2.698991	0.004281	T
ENSG0000(	AC233968	0.187	0.4836	2.033449	0.004289	T
ENSG0000(	ZNF431	8.9768	2.1193	0.244502	0.004295	N
ENSG0000(	WWC1	9.0432	3.0534	0.34489	0.004312	N
ENSG0000(	AP003108.	0.1725	0.4599	2.054679	0.004313	T
ENSG0000(	AC093673	2.0246	6.0391	2.889532	0.004314	T
ENSG0000(	RPL7P24	0.0276	0.311	3.221003	0.004322	T
ENSG0000(	ENGASE	0.9446	2.7072	2.687344	0.004325	T
ENSG0000(	AC098934	0.0262	0.2127	2.477813	0.004333	T
ENSG0000(	ADH5P4	0.2347	0.8201	2.749029	0.004334	T
ENSG0000(	AL096678.	0.0887	0.3793	2.540011	0.004335	T
ENSG0000(	AC127024	0.4252	1.4643	2.978484	0.004353	T

ENSG0000(	LBR	7.7133	15.9164	2.049889	0.00436	T
ENSG0000(	MMACHC	0.645	1.853	2.621477	0.004364	T
ENSG0000(	GUSBP4	0.06	0.323	2.64375	0.004377	T
ENSG0000(	MIR604	2.6052	8.4359	3.155367	0.004382	T
ENSG0000(	P4HA2	1.3544	3.037	2.156903	0.004386	T
ENSG0000(	CNTLN	0.5517	1.467	2.404481	0.004391	T
ENSG0000(	ELOVL6	7.2056	3.1344	0.442729	0.004391	N
ENSG0000(	WBP11	6.9124	14.0101	2.012164	0.004394	T
ENSG0000(	AC122710	0.0178	0.1886	2.449915	0.004405	T
ENSG0000(	MTCO2P1	0.0693	0.7431	4.979917	0.004407	T
ENSG0000(	AC009301	0.1346	0.5668	2.842285	0.004411	T
ENSG0000(	ZNF232	0.609	1.5067	2.26615	0.004414	T
ENSG0000(	AL357079.	0.1444	0.677	3.179214	0.004418	T
ENSG0000(	ZDHHC11	0.3508	2.1057	4.892857	0.004421	T
ENSG0000(	ABCC4	1.0062	3.4115	3.174381	0.004432	T
ENSG0000(	WDR5B	0.7992	2.6313	3.037478	0.004434	T
ENSG0000(	SLC25A35	0.3984	1.0831	2.373796	0.004448	T
ENSG0000(	GLA	2.6781	11.1511	4.049926	0.00445	T
ENSG0000(	PRNCR1	0.1154	0.6296	3.387187	0.00446	T
ENSG0000(	XRCC6P2	0.5852	1.3374	2.097782	0.004463	T
ENSG0000(	NRSN2	1.0432	3.608	3.243527	0.004466	T
ENSG0000(	INTS13	5.1209	10.5846	2.046505	0.004476	T
ENSG0000(	AMN	0.7903	2.2483	2.63765	0.004478	T
ENSG0000(	HUNK	0.0849	0.3599	2.48729	0.004479	T
ENSG0000(	RPL7P49	0.238	0.7514	2.518935	0.004481	T
ENSG0000(	MRPL45P1	0.0157	0.3181	3.613656	0.004484	T
ENSG0000(	AC110769	0.6238	2.1227	3.070876	0.004486	T
ENSG0000(	MSANTD3	1.9568	4.1847	2.083187	0.004492	T
ENSG0000(	ADAP2	1.0575	2.8391	2.539179	0.004496	T
ENSG0000(	AC245060	59.2666	17.2328	0.291962	0.004503	N
ENSG0000(	HIST2H3C	0.1717	1.4437	5.681634	0.004504	T
ENSG0000(	PCBP1-AS	2.7902	1.2531	0.468168	0.004505	N
ENSG0000(	UNC93A	2.0122	0.5084	0.288041	0.004508	N
ENSG0000(	SCARNA3	1.2959	5.7912	4.22036	0.004509	T
ENSG0000(	AC092139	0.5472	2.4125	3.882108	0.00451	T
ENSG0000(	GSPT2	0.7882	1.8735	2.221909	0.00451	T
ENSG0000(	LINC0254:	1.8776	6.4266	3.300263	0.004512	T
ENSG0000(	AC115618	2.2182	5.4387	2.389224	0.004522	T
ENSG0000(	MTCYBP2	0.0672	0.3621	2.763756	0.004523	T
ENSG0000(	IFI30	0.0821	0.495	3.267435	0.004532	T
ENSG0000(	TENT5A	1.4652	5.4018	3.515078	0.004534	T
ENSG0000(	DLG2	0.8049	0.3032	0.445574	0.004538	N
ENSG0000(	GSTM2	0.4442	1.8678	3.61595	0.00454	T
ENSG0000(	AL591846.	0.0201	0.555	5.453789	0.004542	T
ENSG0000(	THNSL1	0.8299	2.3127	2.59458	0.004561	T
ENSG0000(	ROBO3	0.2612	1.0482	3.178848	0.004571	T
ENSG0000(	AC106820	0.0193	0.2795	3.181056	0.004574	T
ENSG0000(	CLEC18B	0.3222	0.1064	0.488868	0.004575	N
ENSG0000(	AC004980	0.5631	1.5973	2.559644	0.004576	T
ENSG0000(	PKMP4	0.065	0.3714	2.85697	0.004578	T
ENSG0000(	AP005212.	0.8871	0.0547	0.156722	0.004588	N
ENSG0000(	ULBP3	1.142	3.261	2.706119	0.004588	T
ENSG0000(	MIR4754	0.3774	1.3717	3.08274	0.00459	T
ENSG0000(	LAMB1	2.7811	7.4717	2.628059	0.004592	T
ENSG0000(	GLIPR1	1.7958	4.8038	2.586665	0.004593	T
ENSG0000(	AC100827	0.1047	0.4723	2.795799	0.004598	T
ENSG0000(	SNORA5C	3.1082	15.9154	4.99202	0.004598	T
ENSG0000(	MAP10	0.2736	0.8961	2.666221	0.004599	T

ENSG0000(	CCDC173	0.0161	0.2808	3.279931	0.004601	T
ENSG0000(	NFE2L3	1.4174	4.583	3.0862	0.004603	T
ENSG0000(	TMEM123	29.2884	380.3864	12.94682	0.004605	T
ENSG0000(	PLCE1	0.2347	1.4354	4.587392	0.004606	T
ENSG0000(	RRM2B	3.2297	7.0112	2.135688	0.004614	T
ENSG0000(	RNASE10	0.0109	0.7134	7.334536	0.004623	T
ENSG0000(	MSNP1	0.0573	0.2868	2.458996	0.004625	T
ENSG0000(	MAP2	2.303	0.7311	0.345859	0.004627	N
ENSG0000(	SNRPGP9	0.6977	2.3138	3.02595	0.004631	T
ENSG0000(	AC018868	1.9989	4.9138	2.388775	0.004638	T
ENSG0000(	MIR28	0.4328	3.3245	6.427365	0.004645	T
ENSG0000(	TGFA-IT1	0.2334	1.1079	3.622975	0.004646	T
ENSG0000(	IL12RB2	0.2897	1.382	3.802925	0.004655	T
ENSG0000(	FXYD3	0.7059	3.0915	3.960169	0.004664	T
ENSG0000(	TSFM	1.1782	2.6865	2.180019	0.004671	T
ENSG0000(	HRK	0.0423	0.6475	5.252987	0.004675	T
ENSG0000(	SNORA2B	0.9739	3.8114	3.642239	0.004693	T
ENSG0000(	AL139353.	0.0896	0.6803	4.115506	0.004696	T
ENSG0000(	DENND4A	2.6701	7.984	2.918306	0.0047	T
ENSG0000(	VSIG2	31.6641	2.7139	0.088587	0.0047	N
ENSG0000(	AL121832.	0.0562	0.2739	2.393726	0.004705	T
ENSG0000(	MIR659	0.6639	1.9206	2.645111	0.004705	T
ENSG0000(	ZDHHC11	0.6321	2.6744	3.789646	0.004706	T
ENSG0000(	SMAD3	8.3664	20.4999	2.433136	0.004712	T
ENSG0000(	AC007823	0.1707	0.5577	2.429627	0.004725	T
ENSG0000(	SNHG6	16.7665	37.1681	2.209593	0.004731	T
ENSG0000(	AC002347	0.0372	0.3034	2.940233	0.004732	T
ENSG0000(	AC011445	0.0434	0.2207	2.236402	0.004733	T
ENSG0000(	GCNT2	0.565	1.5962	2.550677	0.00474	T
ENSG0000(	AC036214	0.7943	1.8605	2.192217	0.004761	T
ENSG0000(	RPS20P2	2.6649	5.5019	2.026077	0.004778	T
ENSG0000(	PLOD3	0.9056	3.6761	3.755072	0.004787	T
ENSG0000(	TFAP2A-A	0.193	0.6428	2.535154	0.00479	T
ENSG0000(	AC015911	2.7394	6.7122	2.399169	0.004795	T
ENSG0000(	CYP2S1	10.5654	57.2054	5.373019	0.004797	T
ENSG0000(	AC025219	0.6148	4.3898	6.281198	0.004805	T
ENSG0000(	AC034102	0.0393	0.4347	3.838478	0.004806	T
ENSG0000(	AC138965	0.0084	0.2594	3.315498	0.004811	T
ENSG0000(	LINC-PINT	0.7125	3.1039	3.943262	0.004811	T
ENSG0000(	AC110749	0.7351	1.5734	2.003832	0.004813	T
ENSG0000(	ABCA1	2.8564	7.4784	2.563388	0.004814	T
ENSG0000(	LINC0142	0.1261	0.4122	2.265369	0.004818	T
ENSG0000(	HERC2P2	1.6898	3.6275	2.082635	0.004824	T
ENSG0000(	AL023775.	0.024	0.2313	2.671774	0.004828	T
ENSG0000(	AL356273.	0.1766	1.0236	4.062184	0.004832	T
ENSG0000(	AC099499	0.1898	0.7124	2.803313	0.004832	T
ENSG0000(	AP000654.	0.0282	0.2633	2.833853	0.004841	T
ENSG0000(	9-Mar	0.9431	2.3033	2.303998	0.004859	T
ENSG0000(	OLA1P1	0.4906	1.671	2.998645	0.004864	T
ENSG0000(	AC064836	0.2026	0.6982	2.637806	0.004867	T
ENSG0000(	RNU6-121	0	0.3593	4.593	0.004868	T
ENSG0000(	AC091544	0	0.2141	3.141	0.004872	T
ENSG0000(	RNU6-920	0.1957	1.4312	5.178221	0.004873	T
ENSG0000(	SLC39A6	7.2611	26.7303	3.644876	0.004881	T
ENSG0000(	CSAD	1.3217	3.0868	2.241542	0.004889	T
ENSG0000(	PPP4R3A	9.3762	19.664	2.085646	0.004896	T
ENSG0000(	FMO4	0.9269	3.2499	3.262148	0.004899	T
ENSG0000(	CFAP44	0.2341	0.9041	3.005388	0.004914	T

ENSG0000(	DDTL	0.5351	1.2257	2.087388	0.004915	T
ENSG0000(	AC011586	0	0.2878	3.878	0.004916	T
ENSG0000(	ENPEP	0.0359	0.499	4.407653	0.004934	T
ENSG0000(	LINC0111'	1.1003	0.3842	0.403399	0.004935	N
ENSG0000(	KRTAP4-1	0	0.3699	4.699	0.004938	T
ENSG0000(	FADS3	0.2266	1.4268	4.674832	0.004939	T
ENSG0000(	AC005786	0.0455	0.2762	2.585567	0.004939	T
ENSG0000(	PLEKHG4	0.1776	0.8629	3.46866	0.004939	T
ENSG0000(	RF00100	34.4498	96.3373	2.791255	0.004941	T
ENSG0000(	LRP8	0.8814	3.992	4.169554	0.004944	T
ENSG0000(	ZNF761	1.3715	3.2137	2.25192	0.004945	T
ENSG0000(	AC105460	0.0374	6.5139	48.1361	0.004945	T
ENSG0000(	AC090114	0.5084	1.8138	3.145628	0.004952	T
ENSG0000(	AC023818	0.0295	0.1924	2.257915	0.004952	T
ENSG0000(	AL365217.	0.1114	0.3268	2.018921	0.004954	T
ENSG0000(	MC5R	0.2983	0.0089	0.273412	0.004959	N
ENSG0000(	HIST1H4E	18.1603	39.1746	2.150819	0.00496	T
ENSG0000(	PAIP2B	10.11	4.3313	0.434016	0.004962	N
ENSG0000(	AC007191	0.3447	1.0152	2.507758	0.004962	T
ENSG0000(	CCDC40	0.0968	0.2949	2.006606	0.004964	T
ENSG0000(	AP003469.	0.3651	1.0783	2.533434	0.004978	T
ENSG0000(	SNORD15.	29.1532	72.3628	2.47709	0.004983	T
ENSG0000(	RNU6-9	9.2459	22.8991	2.460876	0.004987	T
ENSG0000(	AC055764	0.2659	1.43	4.18147	0.00499	T
ENSG0000(	IL15RA	0.8677	2.7913	2.987806	0.00499	T
ENSG0000(	AC142086	0.7498	0.0894	0.222876	0.005007	N
ENSG0000(	AL136038.	0.5129	1.1832	2.093653	0.005008	T
ENSG0000(	SLAMF6P	0.6471	0.1573	0.344398	0.00501	N
ENSG0000(	MTAPP2	0.0679	0.2896	2.320429	0.005011	T
ENSG0000(	NUDT15	4.3635	10.4671	2.367447	0.005013	T
ENSG0000(	HNRNPUI	1.1257	4.1731	3.486253	0.005028	T
ENSG0000(	MIR7845	0.9314	3.2311	3.229688	0.005031	T
ENSG0000(	AC073335	0.5948	1.3333	2.062896	0.005048	T
ENSG0000(	SLC35E4	0.4954	1.361	2.453813	0.005049	T
ENSG0000(	AC087623	0.3232	1.2625	3.219518	0.00505	T
ENSG0000(	AL589986.	7.6103	2.069	0.281312	0.005051	N
ENSG0000(	PRR3	0.686	2.0848	2.779644	0.005053	T
ENSG0000(	AL158163.	0.1858	0.5814	2.384185	0.005057	T
ENSG0000(	ATP11C	1.6046	4.7907	2.869119	0.005058	T
ENSG0000(	KCNMB3	0.0454	0.3753	3.268913	0.005065	T
ENSG0000(	AC135279	0.0474	0.2142	2.131615	0.005065	T
ENSG0000(	AC078802	0.4349	1.654	3.279118	0.005067	T
ENSG0000(	AC012254	0.0276	0.2004	2.354232	0.005069	T
ENSG0000(	CHST6	0.4436	0.1443	0.449411	0.005086	N
ENSG0000(	ESR1	0.1018	0.4261	2.607037	0.005088	T
ENSG0000(	CCDC61	0.9925	2.124	2.035698	0.005091	T
ENSG0000(	AC004801	0.1081	0.3371	2.100432	0.005095	T
ENSG0000(	CTHRC1	0.0903	1.2699	7.198634	0.005096	T
ENSG0000(	AP000936.	1.5451	5.4301	3.361559	0.005098	T
ENSG0000(	NFATC1	0.4105	3.6507	7.347111	0.005105	T
ENSG0000(	APOC1	0.3736	2.2694	5.002956	0.005112	T
ENSG0000(	HNRNPD	11.2782	23.3922	2.064668	0.005114	T
ENSG0000(	AL354993.	0.0265	0.1835	2.241107	0.005114	T
ENSG0000(	EVA1A	0.0407	0.3175	2.967306	0.005116	T
ENSG0000(	RHEBP1	0.4796	1.6024	2.937198	0.005118	T
ENSG0000(	PDCL3P5	0.2637	0.7207	2.25653	0.005131	T
ENSG0000(	AC007038	0.6346	2.1377	3.046148	0.005132	T
ENSG0000(	MIR548D1	0.8113	5.1949	5.810271	0.005138	T

ENSG0000(	AL714022.	0.131	0.6413	3.209091	0.005138	T
ENSG0000(	DTX3L	9.0553	25.408	2.786146	0.005147	T
ENSG0000(	PLXND1	1.5566	4.0278	2.49173	0.00515	T
ENSG0000(	ERF	5.6	14.9402	2.638632	0.005151	T
ENSG0000(	PPIAP6	0.5119	1.1477	2.039059	0.005159	T
ENSG0000(	AC098820	0.0678	0.3948	2.948749	0.005159	T
ENSG0000(	IRX4	2.7208	5.8155	2.0971	0.005166	T
ENSG0000(	CBR1	32.4622	70.7112	2.174644	0.005177	T
ENSG0000(	SLCO6A1	0.0289	0.5735	5.224981	0.005183	T
ENSG0000(	GRHL3	62.3263	30.0479	0.482936	0.005199	N
ENSG0000(	NDUFAF4	0	0.6892	7.892	0.005211	T
ENSG0000(	PCK2	2.1843	5.0578	2.257935	0.005212	T
ENSG0000(	MRAP2	0.6726	4.3226	5.724308	0.005219	T
ENSG0000(	SERPINH1	2.4323	9.2454	3.690479	0.005224	T
ENSG0000(	AL033523.	0	1.2274	13.274	0.005227	T
ENSG0000(	UCA1	1.5424	0.3512	0.27472	0.005228	N
ENSG0000(	HIST1H1A	11.7112	43.1862	3.664844	0.005247	T
ENSG0000(	YBX1P2	0.7329	2.5156	3.140353	0.005247	T
ENSG0000(	SAMD12	1.4699	3.9916	2.606281	0.005249	T
ENSG0000(	CRYBB3	0.3387	1.4025	3.424892	0.005249	T
ENSG0000(	DUXAP9	0.01	0.3525	4.113636	0.005253	T
ENSG0000(	GPR158	0.0011	0.6524	7.442136	0.005261	T
ENSG0000(	AC110921	0.0448	0.2584	2.475138	0.005261	T
ENSG0000(	ALG1L2	0.0505	0.4893	3.915615	0.005262	T
ENSG0000(	MIR572	0	1.3059	14.059	0.005265	T
ENSG0000(	STAB1	1.7876	5.4036	2.91566	0.005268	T
ENSG0000(	TPT1P3	0.2267	0.0431	0.438017	0.005277	N
ENSG0000(	AKR1B1	2.5223	13.474	5.176372	0.005278	T
ENSG0000(	GABRA4	0.1749	0.0333	0.484904	0.005292	N
ENSG0000(	AC009961	0.0173	0.3854	4.138107	0.005293	T
ENSG0000(	CTU2	0.7618	1.8092	2.215363	0.005294	T
ENSG0000(	AC129492	0.0667	0.4588	3.35213	0.0053	T
ENSG0000(	AL807757.	0.1367	1.127	5.183777	0.005302	T
ENSG0000(	PTPRJ	1.1566	3.6368	2.973739	0.005313	T
ENSG0000(	LINC0049.	0.4038	3.2253	6.600437	0.005315	T
ENSG0000(	AL691520.	0	0.8758	9.758	0.005326	T
ENSG0000(	CYCSP55	0.6153	2.1219	3.106249	0.005341	T
ENSG0000(	FAM20C	1.4608	4.7411	3.101679	0.005352	T
ENSG0000(	IRAK3	0.5479	1.6361	2.67958	0.005378	T
ENSG0000(	MIR3125	0.4417	2.9092	5.555104	0.005379	T
ENSG0000(	UPK1A	24.6337	3.9562	0.163995	0.005388	N
ENSG0000(	AC098613	0.1307	0.4355	2.321196	0.005398	T
ENSG0000(	SAMD5	7.1542	3.0723	0.437305	0.005398	N
ENSG0000(	RF00012	3.3713	19.1737	5.5523	0.005403	T
ENSG0000(	WDR18	4.5327	9.7973	2.1364	0.005408	T
ENSG0000(	MIR9-3HG	0.0329	0.3641	3.492099	0.005408	T
ENSG0000(	PGAM5	4.4606	11.7103	2.589637	0.005417	T
ENSG0000(	RF00494	1.2167	9.3442	7.172629	0.005418	T
ENSG0000(	AP000873.	0.5261	1.4558	2.484907	0.005421	T
ENSG0000(	PARP14	4.5454	18.4772	3.999053	0.005427	T
ENSG0000(	ZSCAN12I	0.1495	0.9832	4.341483	0.00543	T
ENSG0000(	AL445205.	0.0321	0.8527	7.211961	0.005433	T
ENSG0000(	HNRNPCF	0.3162	0.8485	2.278952	0.005439	T
ENSG0000(	ANOS1	0.6377	1.8509	2.644571	0.005444	T
ENSG0000(	AC104088	0.6466	3.2024	4.423252	0.005448	T
ENSG0000(	THAP12	1.7636	8.0945	4.397135	0.00545	T
ENSG0000(	MNS1	0.3316	1.5258	3.766914	0.00545	T
ENSG0000(	USP36	2.2089	5.58	2.460046	0.005451	T

ENSG0000(	LYG1	0.1429	0.7894	3.661589	0.005452	T
ENSG0000(	AC134349	0.4126	1.6146	3.344908	0.005454	T
ENSG0000(	ANKRD26	0.0629	0.4018	3.080417	0.00546	T
ENSG0000(	AP003390.	0.3616	1.314	3.063258	0.005463	T
ENSG0000(	OVOL2	1.3366	3.7197	2.658847	0.005468	T
ENSG0000(	Z82243.1	1.161	3.4608	2.823791	0.00547	T
ENSG0000(	MIR3128	1.7448	6.7483	3.712218	0.005478	T
ENSG0000(	PHACTR2	27.2445	9.3255	0.344695	0.005479	N
ENSG0000(	CHCHD3	4.1176	9.4487	2.264013	0.005479	T
ENSG0000(	ISYNA1	1.0754	2.9702	2.612047	0.005487	T
ENSG0000(	C19orf73	0.5792	1.3791	2.177709	0.005491	T
ENSG0000(	FO704657.	0.1445	0.6732	3.162372	0.005507	T
ENSG0000(	C2orf66	0.0484	0.2452	2.326146	0.005508	T
ENSG0000(	GUSBP2	0.4318	1.2176	2.477623	0.00551	T
ENSG0000(	RNU5A-1	16.1951	69.5682	4.275408	0.005514	T
ENSG0000(	AF064858.	0.0522	0.574	4.428384	0.005518	T
ENSG0000(	GHDC	0.7341	2.156	2.704712	0.005523	T
ENSG0000(	AL049840.	0.0126	0.4806	5.156306	0.005524	T
ENSG0000(	KRT8P51	0.0261	1.2897	11.02062	0.005525	T
ENSG0000(	SNORA24	0.3134	1.1786	3.092888	0.005525	T
ENSG0000(	ZNF41	1.4938	3.3266	2.149956	0.005529	T
ENSG0000(	TRPV1	0.2715	0.7931	2.404038	0.00554	T
ENSG0000(	HNRNPA1	0.1536	0.5221	2.453076	0.005542	T
ENSG0000(	BTC	2.6461	1.2632	0.496413	0.005542	N
ENSG0000(	CYP2D7	0.2395	0.7291	2.442121	0.005548	T
ENSG0000(	RNU6-954	11.0795	2.2163	0.207192	0.005556	N
ENSG0000(	PPRC1	4.672	9.9615	2.108445	0.005561	T
ENSG0000(	CACYBPP	0.3681	1.4918	3.400555	0.005563	T
ENSG0000(	AC107959	0.0211	0.316	3.435178	0.005571	T
ENSG0000(	RPL36A	0.3579	1.2479	2.943656	0.005582	T
ENSG0000(	AC005746	0.1493	0.4194	2.083434	0.005595	T
ENSG0000(	CASC15	0.0822	0.3372	2.399561	0.005597	T
ENSG0000(	AC026356	0.0357	0.214	2.313928	0.005599	T
ENSG0000(	AL136018.	0.0161	4.4504	39.1938	0.005614	T
ENSG0000(	AC034187	0.5606	1.7374	2.781411	0.005615	T
ENSG0000(	AGPAT4-I	0.1669	0.5367	2.385538	0.005616	T
ENSG0000(	PRKAG2	2.0898	4.3911	2.050918	0.005618	T
ENSG0000(	RN7SKP12	0.1764	0.7311	3.006874	0.005619	T
ENSG0000(	ILF3-DT	1.1638	2.7689	2.270059	0.005625	T
ENSG0000(	TATDN1P	0.1125	0.3656	2.191059	0.005627	T
ENSG0000(	RF00019	0.5845	3.0437	4.592695	0.005628	T
ENSG0000(	ADGRB1	0.1651	0.6331	2.765372	0.005634	T
ENSG0000(	P2RX6P	0.4668	0.0317	0.232357	0.005635	N
ENSG0000(	DDX47	0.1575	0.6934	3.081165	0.005636	T
ENSG0000(	AC016205	0.201	0.9098	3.354817	0.005644	T
ENSG0000(	LUC7L	2.0263	4.6814	2.248695	0.005648	T
ENSG0000(	FAM216A	0.4535	1.1409	2.241915	0.005657	T
ENSG0000(	RNU5D-1	2.9554	18.2516	6.006284	0.005657	T
ENSG0000(	LARGE-IT	0.0729	0.4745	3.32273	0.005662	T
ENSG0000(	AC067904	0.2582	0.7797	2.455891	0.005663	T
ENSG0000(	AP001429.	0.4129	1.4791	3.078768	0.005666	T
ENSG0000(	PILRB	0.1811	0.5595	2.34614	0.005671	T
ENSG0000(	CLC	0.0774	0.8736	5.488162	0.005674	T
ENSG0000(	GABPB1-I	0.8734	1.9999	2.157284	0.005676	T
ENSG0000(	HSBP1L1	7.6692	3.6675	0.484928	0.005676	N
ENSG0000(	SLC25A37	1.7344	7.7964	4.304623	0.005681	T
ENSG0000(	TCTE3	0.3364	0.8068	2.07791	0.005684	T
ENSG0000(	AC027682	0.0739	0.4494	3.159287	0.005685	T

ENSG0000	AC092969	0	0.5664	6.664	0.005688	T
ENSG0000	AC004846	0.2541	0.7879	2.507484	0.005691	T
ENSG0000	AC087362	0.1291	0.5343	2.76866	0.005702	T
ENSG0000	LINC0184	0.011	0.2498	3.151351	0.005724	T
ENSG0000	UVRAG	1.7316	4.59	2.560603	0.005729	T
ENSG0000	RNU6-729	0.1115	0.4613	2.653901	0.00573	T
ENSG0000	RF00019	0.4988	1.8294	3.222111	0.00573	T
ENSG0000	MTHFD2	3.6392	17.318	4.658216	0.00573	T
ENSG0000	AL031963.	0.1627	0.4811	2.212029	0.005734	T
ENSG0000	RF00019	0.0333	0.9957	8.219805	0.005739	T
ENSG0000	AL031666.	0.0572	0.2466	2.204835	0.005742	T
ENSG0000	ELMO3	4.2982	9.3839	2.156314	0.005744	T
ENSG0000	U40455.1	0.0423	0.218	2.234715	0.005765	T
ENSG0000	AF064858.	0.1296	1.2153	5.728659	0.005767	T
ENSG0000	TBCAP3	0.2858	1.0184	2.898911	0.00577	T
ENSG0000	AC067931	0.2754	0.9044	2.675546	0.005775	T
ENSG0000	MRPL42	1.9118	4.0728	2.074162	0.005776	T
ENSG0000	MET	3.1537	16.4158	5.076006	0.005783	T
ENSG0000	CDH11	0.1873	0.9944	3.809259	0.005783	T
ENSG0000	AP000941.	0.0581	0.2207	2.028463	0.005785	T
ENSG0000	FABP5P10	1.0427	4.0706	3.649777	0.00579	T
ENSG0000	SNRPF	1.9449	4.4877	2.243484	0.005793	T
ENSG0000	P2RY1	10.6597	29.9558	2.793368	0.005795	T
ENSG0000	AL627308.	0.067	1.022	6.718563	0.0058	T
ENSG0000	SYNE2	8.5578	18.587	2.158401	0.005808	T
ENSG0000	RF00019	0.4709	2.6706	4.853039	0.005816	T
ENSG0000	SCARNA1	0.1805	1.5311	5.814973	0.005819	T
ENSG0000	ADIPOR2	31.596	15.419	0.48962	0.005824	N
ENSG0000	SLC16A9	10.9877	4.9312	0.453764	0.005828	N
ENSG0000	HMCES	6.0896	13.5642	2.207606	0.005833	T
ENSG0000	TCP11L2	31.5028	6.3007	0.202536	0.005836	N
ENSG0000	LINC0106	0.0799	0.4887	3.272374	0.00584	T
ENSG0000	SGK3	1.9135	4.8149	2.440973	0.00584	T
ENSG0000	LYPD6	0.1728	0.8565	3.506232	0.005842	T
ENSG0000	AC009054	0.1462	0.6252	2.945573	0.005849	T
ENSG0000	REV3L	2.3561	5.0572	2.099752	0.00585	T
ENSG0000	AC005072	0.1337	0.8168	3.922978	0.005853	T
ENSG0000	TAB3	13.0269	5.83	0.451744	0.005856	N
ENSG0000	AL024507.	0.0678	0.3188	2.495828	0.005856	T
ENSG0000	LGALS3B	25.7911	67.2405	2.600913	0.005857	T
ENSG0000	AL024508.	0.1868	0.7432	2.940028	0.005861	T
ENSG0000	AL080317.	0.1819	0.6068	2.507272	0.005861	T
ENSG0000	C8orf44	0.3495	1.4004	3.337931	0.005867	T
ENSG0000	AC074194	0.3093	1.2918	3.40044	0.005876	T
ENSG0000	N4BP2L2-	0.5339	1.2553	2.138034	0.005878	T
ENSG0000	CYP2E1	2.648	0.8713	0.353457	0.00588	N
ENSG0000	STRIP2	0.1753	1.2377	4.859063	0.005894	T
ENSG0000	SEPT7P2	0.9531	2.208	2.191625	0.005894	T
ENSG0000	TPM3P7	0.0631	0.4341	3.274678	0.005895	T
ENSG0000	LRRC14	1.3819	3.288	2.286254	0.005903	T
ENSG0000	MYOSLID	0.1571	1.2	5.056398	0.00591	T
ENSG0000	AC098487	0.0297	0.1737	2.110254	0.005913	T
ENSG0000	RN7SKP18	11.0794	29.306	2.630374	0.005918	T
ENSG0000	CD207	12.6204	2.1121	0.173902	0.005923	N
ENSG0000	AC007920	0.5091	0.0623	0.266459	0.005933	N
ENSG0000	MAMSTR	0.3957	1.0709	2.362114	0.005938	T
ENSG0000	RASL11B	0.0612	0.5257	3.881514	0.005939	T
ENSG0000	SPHK2	0.9131	1.9437	2.017274	0.005946	T

ENSG0000(	RF00493	4.5365	15.5688	3.379446	0.005949	T
ENSG0000(	TNFSF10	25.222	70.2389	2.777778	0.005954	T
ENSG0000(	AC021028	0.0454	0.3079	2.805365	0.005956	T
ENSG0000(	AC131532	0.2677	4.9126	13.63231	0.005964	T
ENSG0000(	RNU1-122	1.7123	4.9443	2.783369	0.005974	T
ENSG0000(	MED17	0.1865	0.6626	2.66178	0.005978	T
ENSG0000(	AC020663	0.3283	0.8828	2.294653	0.005983	T
ENSG0000(	CMAHP	0.2748	1.0893	3.173159	0.005987	T
ENSG0000(	THBS2	2.4556	13.9343	5.491587	0.005992	T
ENSG0000(	AC084824	0.3246	1.2688	3.22374	0.006007	T
ENSG0000(	AC104113	0.138	0.5327	2.658403	0.006009	T
ENSG0000(	AC090589	0.3847	1.1384	2.554982	0.006012	T
ENSG0000(	MTCP1	0.1002	0.3073	2.034466	0.006015	T
ENSG0000(	DMXL2	0.8929	3.5671	3.693323	0.00602	T
ENSG0000(	RHBDL1	0.2036	0.8586	3.157444	0.00602	T
ENSG0000(	ZIC5	0.0038	1.3965	14.41715	0.006042	T
ENSG0000(	AP001931.	0.8401	2.5545	2.823636	0.006047	T
ENSG0000(	ANXA5	17.6703	39.1069	2.206316	0.006047	T
ENSG0000(	TMEM251	1.547	4.1812	2.599393	0.006058	T
ENSG0000(	RHOQP3	0.0667	0.2524	2.113977	0.006063	T
ENSG0000(	HMGB3	1.9425	11.1323	5.49929	0.006068	T
ENSG0000(	CD86	1.1987	4.0544	3.198891	0.006072	T
ENSG0000(	NUDT16P.	0.5813	2.211	3.392045	0.006074	T
ENSG0000(	ZNF667-A	0.7873	0.2095	0.348811	0.006088	N
ENSG0000(	AC018926	0.1178	0.4219	2.396235	0.006098	T
ENSG0000(	DLEU2L	0.0644	0.2845	2.338808	0.006099	T
ENSG0000(	LNCOC1	0.034	0.1816	2.101493	0.006099	T
ENSG0000(	AC120114	0.1253	0.3798	2.129605	0.006105	T
ENSG0000(	FGFR2	4.9121	10.7187	2.158516	0.006105	T
ENSG0000(	CXCL6	0.0675	7.2298	43.76	0.006107	T
ENSG0000(	AC108047	0.4157	1.4675	3.039558	0.006109	T
ENSG0000(	PLPPR2	2.1391	4.6494	2.12112	0.006114	T
ENSG0000(	PMS2P3	1.4519	3.1937	2.122366	0.00612	T
ENSG0000(	MIR1910	0.5987	5.2976	7.725204	0.006121	T
ENSG0000(	SIGLEC7	0.1826	0.631	2.586695	0.00613	T
ENSG0000(	AP000238.	0.0214	0.2562	2.934102	0.006133	T
ENSG0000(	SNRPGP1(	4.0931	9.0961	2.193151	0.006138	T
ENSG0000(	ALOX12	85.8859	18.0845	0.211482	0.006141	N
ENSG0000(	AC011369	0.1106	0.519	2.939221	0.006157	T
ENSG0000(	LNX1	18.8402	3.2225	0.175421	0.006162	N
ENSG0000(	BCL7A	0.8937	2.1063	2.220288	0.006166	T
ENSG0000(	PLEKHG7	0.0044	0.34	4.214559	0.00617	T
ENSG0000(	GALNT1	55.6857	24.5745	0.442309	0.006182	N
ENSG0000(	AC008267	0.0403	0.1832	2.018532	0.006182	T
ENSG0000(	PCDHGA1	0.0711	0.3079	2.383986	0.006195	T
ENSG0000(	DGKH	1.9551	5.9961	2.966328	0.006198	T
ENSG0000(	KCNH8	0.0401	0.244	2.455389	0.006202	T
ENSG0000(	DST	8.9401	27.4606	3.048705	0.006208	T
ENSG0000(	GSDMB	0.3472	1.5923	3.784213	0.00621	T
ENSG0000(	AF111169.	0.133	0.4025	2.156652	0.006215	T
ENSG0000(	AC008467	0.1244	0.4239	2.33467	0.006216	T
ENSG0000(	AC092279	0.7579	1.6428	2.031472	0.006217	T
ENSG0000(	S100P	62.8302	29.8888	0.476541	0.006225	N
ENSG0000(	RF00017	0	0.2259	3.259	0.006238	T
ENSG0000(	RF00019	3.5843	11.2149	3.071113	0.006238	T
ENSG0000(	AL035411.	0.2803	1.3659	3.854588	0.00624	T
ENSG0000(	ASPHD1	0.0939	0.4739	2.959773	0.006241	T
ENSG0000(	LINC0156(	0.1244	0.461	2.5	0.006244	T

ENSG0000(	CDC42P4	0.0704	0.2669	2.153169	0.006251	T
ENSG0000(	AL162311.	0.0884	0.6	3.715499	0.006251	T
ENSG0000(	LAMB4	1.3233	0.1743	0.192721	0.006256	N
ENSG0000(	AC063952	0	0.6183	7.183	0.006256	T
ENSG0000(	BLVRB	62.3845	27.7467	0.445658	0.006256	N
ENSG0000(	AC103563	0.4502	0.0601	0.290985	0.006258	N
ENSG0000(	AC096649	0.0208	0.379	3.965232	0.00626	T
ENSG0000(	MORF4L2	0.0498	0.2533	2.358478	0.006261	T
ENSG0000(	AFDN-DT	0.5336	0.2151	0.497317	0.006263	N
ENSG0000(	HSPE1P11	0.2227	1.6212	5.333747	0.00627	T
ENSG0000(	AL360181.	0.1418	0.7316	3.439206	0.00627	T
ENSG0000(	NFIL3	3.3002	11.2503	3.338127	0.006272	T
ENSG0000(	FBLIM1	4.6719	10.432	2.207087	0.006272	T
ENSG0000(	POLR2J3	0.3306	0.9573	2.455411	0.006283	T
ENSG0000(	DCAF4	0.8404	1.9603	2.190876	0.00629	T
ENSG0000(	AL356215.	0.108	0.4209	2.504327	0.006291	T
ENSG0000(	AC069185	0.1459	0.4574	2.266775	0.006291	T
ENSG0000(	AC008026	0.1113	0.3879	2.309039	0.006297	T
ENSG0000(	UCP2	2.0408	8.0362	3.800542	0.006297	T
ENSG0000(	PHF24	0.0315	0.1818	2.142966	0.006306	T
ENSG0000(	RN7SL246	0.386	1.1243	2.519136	0.006314	T
ENSG0000(	RPL7P26	1.2438	2.7858	2.147492	0.006317	T
ENSG0000(	COPZ2	1.2827	3.0512	2.279019	0.006323	T
ENSG0000(	ARPC1B	0.1757	0.7105	2.93979	0.006324	T
ENSG0000(	TARSL2	1.1512	2.4502	2.038203	0.006325	T
ENSG0000(	LRRC20	14.115	2.1634	0.159226	0.006328	N
ENSG0000(	HNRNPLP	0.4263	1.4611	2.966179	0.00635	T
ENSG0000(	LRRC8C	0.5633	2.0737	3.277099	0.006352	T
ENSG0000(	LINC0113(	0.0286	1.1555	9.76283	0.006354	T
ENSG0000(	AC116049	0.0216	0.521	5.106908	0.006359	T
ENSG0000(	AL353807.	0.1015	0.6094	3.520596	0.006361	T
ENSG0000(	IMPDH1	3.9203	9.4478	2.374897	0.006375	T
ENSG0000(	RNU1-146	0	0.4915	5.915	0.006376	T
ENSG0000(	PLEKHA4	1.7216	3.8632	2.17567	0.006381	T
ENSG0000(	ACTG1P1	0.2391	0.5931	2.04394	0.006393	T
ENSG0000(	MTA1	3.6621	7.6526	2.060711	0.006398	T
ENSG0000(	TMEM63C	0.9653	2.8308	2.75115	0.006399	T
ENSG0000(	AP002498.	1.5563	0.2176	0.191753	0.006407	N
ENSG0000(	GCOM2	0.0837	0.3111	2.237888	0.006415	T
ENSG0000(	SLC35A2	3.7129	8.3088	2.205356	0.006422	T
ENSG0000(	SGPP2	15.3564	35.5447	2.306145	0.006424	T
ENSG0000(	FAM90A1	0.0345	0.2258	2.422305	0.006433	T
ENSG0000(	BTN3A1	1.6097	5.3779	3.204012	0.006436	T
ENSG0000(	AL591845.	0.2318	0.5813	2.053345	0.006441	T
ENSG0000(	AC005392	126.4647	10.3801	0.082804	0.006444	N
ENSG0000(	OSTCP5	0.0188	0.1894	2.436027	0.006459	T
ENSG0000(	CEBPB	10.1943	50.5283	4.918091	0.006463	T
ENSG0000(	AL137003.	0.5857	1.8589	2.856789	0.006463	T
ENSG0000(	ZNF426	24.5546	5.4624	0.225613	0.006463	N
ENSG0000(	TP53RK	2.9178	5.9549	2.006395	0.006468	T
ENSG0000(	AC131157	0.021	0.4068	4.18843	0.006478	T
ENSG0000(	SNTB1	2.1104	0.9145	0.458967	0.00648	N
ENSG0000(	NSRP1P1	0.0717	0.2895	2.268492	0.006485	T
ENSG0000(	HS3ST3B1	1.1961	3.5654	2.828023	0.00649	T
ENSG0000(	GJA1	37.7994	111.8129	2.952894	0.006495	T
ENSG0000(	RN7SL336	0.0972	0.7863	4.494422	0.006497	T
ENSG0000(	SMAP2	4.9634	10.8459	2.161769	0.006498	T
ENSG0000(	AL109923.	0.4178	1.4468	2.987254	0.006507	T

ENSG0000(	RAD52	0.7183	1.6667	2.158988	0.006513	T
ENSG0000(	METRN	1.0952	2.4558	2.138387	0.006514	T
ENSG0000(	COA4	5.9453	12.9155	2.152995	0.006515	T
ENSG0000(	RF00017	0.0589	0.4323	3.349906	0.006519	T
ENSG0000(	AP000769.	0.7621	3.862	4.595755	0.006529	T
ENSG0000(	AC008873	0.2713	1.1501	3.366819	0.00653	T
ENSG0000(	AC005831	0.4486	1.2481	2.457346	0.006532	T
ENSG0000(	CCDC69	10.3168	3.5031	0.345893	0.006538	N
ENSG0000(	LBX2-AS1	0.456	2.2885	4.295863	0.006542	T
ENSG0000(	LINC0245(	0	0.2625	3.625	0.006543	T
ENSG0000(	AC079336	0.0672	0.3336	2.593301	0.006544	T
ENSG0000(	CA3-AS1	0.171	0.6329	2.704428	0.006547	T
ENSG0000(	AC009961	0.0846	0.2815	2.066631	0.006557	T
ENSG0000(	AL109809.	0.0934	0.3474	2.31334	0.006561	T
ENSG0000(	GPC3	0.1513	3.4044	13.94509	0.006562	T
ENSG0000(	KRT79	1.1966	0.1332	0.179855	0.006565	N
ENSG0000(	AL121769.	0.2208	0.6789	2.427993	0.006569	T
ENSG0000(	AL359504.	0.1332	0.4936	2.545455	0.006571	T
ENSG0000(	ARVCF	0.4666	1.3087	2.486234	0.006572	T
ENSG0000(	LURAP1L	0.6421	2.0926	2.954588	0.006589	T
ENSG0000(	RF00091	0.0923	1.0703	6.085803	0.006593	T
ENSG0000(	SPNS2	106.2064	14.0698	0.133292	0.006595	N
ENSG0000(	AC005332	0.4725	1.2278	2.319301	0.006603	T
ENSG0000(	PRC1-AS1	0.0511	0.2037	2.009927	0.006606	T
ENSG0000(	AC132192	0.4022	1.0093	2.208881	0.006607	T
ENSG0000(	PCF11-AS	0.2586	0.8928	2.768544	0.006614	T
ENSG0000(	LAMB3	12.9126	69.0222	5.311944	0.006617	T
ENSG0000(	ZNF428	2.3493	4.8424	2.017883	0.00662	T
ENSG0000(	MPPED2	0.2763	1.1648	3.361148	0.00662	T
ENSG0000(	AC099811	0.2338	1.3423	4.320851	0.006621	T
ENSG0000(	ZNF469	0.0954	0.3322	2.211873	0.006624	T
ENSG0000(	AC009093	0.043	0.26	2.517483	0.006624	T
ENSG0000(	BNIP3P1	0.6492	0.2179	0.424319	0.006624	N
ENSG0000(	AL031274.	0.3821	1.0157	2.31425	0.006627	T
ENSG0000(	CACNA2D	1.4811	3.9	2.529884	0.006633	T
ENSG0000(	TAF1D	6.4103	17.4579	2.696942	0.006638	T
ENSG0000(	AL022324.	0.2441	0.6927	2.303691	0.00664	T
ENSG0000(	3-Mar	0.4818	1.3112	2.425576	0.00664	T
ENSG0000(	LINC0066(	0.0178	0.3599	3.904075	0.006643	T
ENSG0000(	SEC14L1P	0.1946	0.499	2.033265	0.006643	T
ENSG0000(	TMEM263	1.7851	5.0002	2.705533	0.006648	T
ENSG0000(	AC010894	0.2069	0.6248	2.361681	0.006657	T
ENSG0000(	AC010186	0.8862	3.1182	3.263233	0.00666	T
ENSG0000(	AC010980	0.0519	0.7182	5.386438	0.006661	T
ENSG0000(	OXTR	0.0308	0.2331	2.546636	0.006661	T
ENSG0000(	GAPT	0.541	1.202	2.031201	0.006664	T
ENSG0000(	AC103923	0.0302	0.1805	2.154378	0.006673	T
ENSG0000(	SDCBP2	38.0137	16.5431	0.43667	0.006674	N
ENSG0000(	SULT1A1	0.2221	0.835	2.902825	0.006681	T
ENSG0000(	ESYT1	8.5104	17.3175	2.022844	0.006687	T
ENSG0000(	F2RL2	0.1416	0.7641	3.576573	0.006688	T
ENSG0000(	AC084125	0.4364	2.6841	5.190343	0.006707	T
ENSG0000(	RNF223	27.9487	2.9063	0.107181	0.006714	N
ENSG0000(	MRPL53	0.4212	1.0773	2.258826	0.00673	T
ENSG0000(	ST6GAL1	0.9424	5.7748	5.63584	0.006734	T
ENSG0000(	AC011479	0.0718	0.4121	2.980792	0.006745	T
ENSG0000(	AC040904	0.1404	1.2818	5.74792	0.006746	T
ENSG0000(	ITGA2B	0.0317	0.1774	2.106302	0.006747	T

ENSG0000	AC093382	0.4623	1.4329	2.726125	0.006753	T
ENSG0000	UBBP1	0.1825	0.5391	2.262301	0.006753	T
ENSG0000	DDX24	4.6796	9.8009	2.071491	0.006754	T
ENSG0000	AC254562	0.1104	0.6116	3.382129	0.006765	T
ENSG0000	SNORA31	0.7424	2.7012	3.325261	0.006773	T
ENSG0000	RUNX2	0.5044	1.368	2.428855	0.006773	T
ENSG0000	FSD1L	0.1327	0.4209	2.238505	0.00678	T
ENSG0000	LGALS3	226.9058	78.2521	0.345155	0.006784	N
ENSG0000	AC000068	0.128	0.4146	2.257018	0.006789	T
ENSG0000	Z93930.2	0.5148	1.6594	2.861744	0.006796	T
ENSG0000	RNU1-4	0.0721	1.4838	9.202789	0.0068	T
ENSG0000	AC002553	0.0788	0.781	4.927293	0.006814	T
ENSG0000	AC107308	0.0497	2.0424	14.31129	0.006816	T
ENSG0000	SCARNA1	0.0711	1.844	11.36178	0.006821	T
ENSG0000	RPS15AP3	0.022	0.1903	2.379508	0.00683	T
ENSG0000	PMFBP1	0.1053	0.6265	3.538724	0.006838	T
ENSG0000	AC009271	0.1355	4.6087	19.99448	0.006839	T
ENSG0000	DOC2B	1.7376	7.4376	4.101872	0.006842	T
ENSG0000	RNU6-7	3.6693	11.8217	3.162842	0.006854	T
ENSG0000	PHLDB2	2.5845	20.9717	7.849395	0.006856	T
ENSG0000	HSPE1P3	0.4693	1.8766	3.471983	0.006859	T
ENSG0000	SNORD19	0.7997	3.3598	3.845504	0.006862	T
ENSG0000	CCDC190	0.6963	12.7377	16.12169	0.006862	T
ENSG0000	AC090425	0.2227	0.7364	2.591881	0.006864	T
ENSG0000	GINS2	0.7354	3.8898	4.775916	0.006866	T
ENSG0000	C1orf226	0.7017	2.2914	2.982911	0.006869	T
ENSG0000	CEP83-DT	0.0328	0.1689	2.024849	0.00687	T
ENSG0000	AC019097	0.0481	0.2114	2.102633	0.00689	T
ENSG0000	AC009262	0.0096	1.648	15.94891	0.006897	T
ENSG0000	AC010997	0.1019	0.3332	2.145617	0.006901	T
ENSG0000	FOXD2-AS	0.1398	0.7426	3.513761	0.006906	T
ENSG0000	RPS6KA5	0.6862	1.606	2.169931	0.006906	T
ENSG0000	CA2	6.9758	20.2023	2.869259	0.00692	T
ENSG0000	RGS17P1	0.546	1.9251	3.13483	0.00692	T
ENSG0000	GABRA3	0.7304	2.0612	2.602601	0.00692	T
ENSG0000	MIR503HG	0.0449	0.3502	3.10697	0.006922	T
ENSG0000	ABCA13	0.3261	11.8476	28.03943	0.006929	T
ENSG0000	BTN3A3	1.1758	3.6137	2.910879	0.006931	T
ENSG0000	RPL34P6	0.0856	1.1351	6.654634	0.006939	T
ENSG0000	AC006027	0.3524	1.1504	2.763926	0.006947	T
ENSG0000	CNTRL	1.1846	2.6015	2.102989	0.006956	T
ENSG0000	TOLLIP-A	0.2513	0.6648	2.177057	0.006962	T
ENSG0000	AL354893.	0.6236	2.0029	2.906164	0.006966	T
ENSG0000	AC096649	0.0401	0.2385	2.416131	0.006969	T
ENSG0000	AL121672.	0.2154	0.5706	2.126189	0.006973	T
ENSG0000	CRTAC1	3.8925	0.2859	0.096656	0.006983	N
ENSG0000	HIST2H3D	0.7887	0.2996	0.449646	0.006985	N
ENSG0000	HSPD1P1	0.3278	1.7097	4.230248	0.006985	T
ENSG0000	AC007848	0.0475	0.46	3.79661	0.006986	T
ENSG0000	AC067852	0.1924	0.6706	2.635431	0.006993	T
ENSG0000	DIAPH2	2.6891	6.5567	2.386684	0.007002	T
ENSG0000	SNORA71.	12.8045	39.6502	3.080336	0.007019	T
ENSG0000	PCSK9	0.3378	2.0507	4.912517	0.007035	T
ENSG0000	LRP4	1.2162	3.6274	2.83194	0.007051	T
ENSG0000	OR5AW1P	0	0.2479	3.479	0.007055	T
ENSG0000	CASP7	2.7647	7.2453	2.564073	0.007058	T
ENSG0000	LRFN2	0.3998	0.1044	0.408964	0.007062	N
ENSG0000	TNFRSF21	29.2717	68.3628	2.33091	0.007063	T

ENSG0000(	AL671883.	0.3788	1.3473	3.022765	0.007065	T
ENSG0000(	AL662797.	0.2059	0.7118	2.653808	0.007067	T
ENSG0000(	AC005726	0.076	0.4869	3.334659	0.007068	T
ENSG0000(	LINC0231(	2.0738	0.4789	0.266308	0.007069	N
ENSG0000(	ISY1	1.1008	2.5638	2.218354	0.007071	T
ENSG0000(	MUC15	15.1403	5.0936	0.340781	0.007072	N
ENSG0000(	KIAA1024	0.1431	0.4116	2.104484	0.007086	T
ENSG0000(	AC016405	0.1506	0.5098	2.43336	0.007087	T
ENSG0000(	AL731684.	0.2346	0.0117	0.333831	0.007092	N
ENSG0000(	TNIP1	33.4996	13.6161	0.408222	0.007103	N
ENSG0000(	RNU6-127	0.0948	0.827	4.758727	0.007113	T
ENSG0000(	AC005351	0.2521	0.9937	3.10622	0.007121	T
ENSG0000(	PSTK	0.1363	0.397	2.103259	0.007123	T
ENSG0000(	AL139317.	0.2253	0.8005	2.768214	0.007125	T
ENSG0000(	IRF9	0.3272	1.3765	3.456227	0.007125	T
ENSG0000(	FSIP2-AS1	0.1274	2.0633	9.513193	0.007127	T
ENSG0000(	GPBAR1	0.0567	0.2487	2.225271	0.007128	T
ENSG0000(	CROCCP2	0.786	1.9376	2.299774	0.007132	T
ENSG0000(	CTLA4	0.2741	2.1595	6.039829	0.007135	T
ENSG0000(	MRPL14	7.5387	17.8115	2.344836	0.007146	T
ENSG0000(	YBX1P4	0.0056	0.2408	3.227273	0.007151	T
ENSG0000(	AL353803.	0.0406	0.1973	2.114509	0.007164	T
ENSG0000(	PRR7	0.9616	2.4505	2.402506	0.007165	T
ENSG0000(	AC019080	0.0169	2.2509	20.11035	0.00717	T
ENSG0000(	AC074033	21.5415	2.2347	0.107881	0.007178	N
ENSG0000(	SDCBPP3	0.1235	0.5492	2.904698	0.00718	T
ENSG0000(	LINC0256(	0.04	0.371	3.364286	0.007193	T
ENSG0000(	SMARCD1	4.5757	9.663	2.08803	0.007197	T
ENSG0000(	CLDN16	0.1034	0.7225	4.043756	0.007205	T
ENSG0000(	LBHD1	0.3084	0.7494	2.079824	0.007209	T
ENSG0000(	PCDHB9	0.1359	1.1042	5.104705	0.007216	T
ENSG0000(	AC083798	0.3994	0.9553	2.113136	0.007225	T
ENSG0000(	SLURP2	19.2798	7.0208	0.367434	0.007228	N
ENSG0000(	PCDHB12	0.0854	0.4001	2.697411	0.007231	T
ENSG0000(	GPR75	1.1887	2.8839	2.315434	0.007238	T
ENSG0000(	LCAT	0.469	1.0749	2.064851	0.007255	T
ENSG0000(	AC121320	0.052	0.4151	3.388816	0.007257	T
ENSG0000(	DDHD1	0.45	1.4417	2.803091	0.00726	T
ENSG0000(	SNORD83.	2.647	9.1854	3.380197	0.007261	T
ENSG0000(	TMEM80	13.7035	3.3203	0.247785	0.007266	N
ENSG0000(	SLC38A2	37.4468	98.4085	2.623619	0.007268	T
ENSG0000(	MIR6509	0.042	0.9072	7.092958	0.00728	T
ENSG0000(	AC005520	0.2691	1.0173	3.027093	0.007282	T
ENSG0000(	SP110	1.179	4.3702	3.495074	0.00729	T
ENSG0000(	TIGD5	0.7746	1.8995	2.286188	0.007292	T
ENSG0000(	RN7SL750	0.0133	0.3886	4.312445	0.007298	T
ENSG0000(	NOL6	3.7805	7.7927	2.033939	0.0073	T
ENSG0000(	MIR205	23.5403	92.5447	3.918931	0.007304	T
ENSG0000(	AC006262	5.7508	0.9487	0.17924	0.007311	N
ENSG0000(	FAM72C	0.1516	0.4979	2.376391	0.007318	T
ENSG0000(	AC099550	0.0229	0.1917	2.373474	0.007333	T
ENSG0000(	RNF217-A	0.1401	0.9403	4.332778	0.007339	T
ENSG0000(	AC010733	0.0863	0.5981	3.747182	0.007341	T
ENSG0000(	TDO2	0.0687	0.4768	3.419087	0.007343	T
ENSG0000(	AL117332.	0.2642	0.8592	2.633718	0.007346	T
ENSG0000(	ARTN	0.1233	1.5527	7.401254	0.007351	T
ENSG0000(	RSL24D1F	0.2149	0.8134	2.900603	0.007352	T
ENSG0000(	BTN3A2	1.3814	3.5339	2.453017	0.007352	T

ENSG0000(	AL133245.	0.0445	0.245	2.387543	0.007367	T
ENSG0000(	SNORD12(	5.8372	14.801	2.509769	0.007372	T
ENSG0000(	BIRC2	5.5768	45.978	8.116897	0.007375	T
ENSG0000(	NKX2-5	0	0.5908	6.908	0.007381	T
ENSG0000(	AC013731	0.2083	0.6237	2.347389	0.007385	T
ENSG0000(	RF00019	0.396	4.2588	8.787903	0.00739	T
ENSG0000(	SCAMP5	0.2518	1.3998	4.263218	0.007396	T
ENSG0000(	MIR548U	0.3689	2.2212	4.950309	0.0074	T
ENSG0000(	RPS3AP37	0.0086	0.2848	3.543278	0.007406	T
ENSG0000(	PAM16	0.363	1.0228	2.425054	0.007415	T
ENSG0000(	HLA-C	51.7452	127.4405	2.460025	0.007419	T
ENSG0000(	GPX8	0.7169	1.8978	2.445587	0.007429	T
ENSG0000(	AC002553	0.4419	2.095	4.050563	0.007432	T
ENSG0000(	IL22RA1	10.8171	4.8613	0.454452	0.007439	N
ENSG0000(	RN7SL141	0.331	2.7247	6.553828	0.007447	T
ENSG0000(	CD24	1045.24	475.0136	0.454506	0.007451	N
ENSG0000(	RF00019	1.206	5.7643	4.490276	0.007456	T
ENSG0000(	NPTN-IT1	0.8129	1.7933	2.07394	0.00746	T
ENSG0000(	ZNF696	0.5336	1.3775	2.331913	0.007466	T
ENSG0000(	MIR4744	0.2765	1.7687	4.963347	0.007467	T
ENSG0000(	AC020978	0.0876	0.496	3.176972	0.007468	T
ENSG0000(	AC097641	0.2435	0.75	2.474527	0.007481	T
ENSG0000(	MIR499B	0.0615	1.0932	7.388235	0.007488	T
ENSG0000(	PYCARD	7.3802	15.6578	2.106601	0.007507	T
ENSG0000(	RNF166	1.1715	2.8611	2.328824	0.007542	T
ENSG0000(	LIME1	0.0925	0.3264	2.215065	0.007548	T
ENSG0000(	ZNF22	2.0768	5.0033	2.344405	0.007551	T
ENSG0000(	SLC29A2	1.0449	3.7251	3.34099	0.007553	T
ENSG0000(	AC009120	0.4794	1.2183	2.275285	0.007556	T
ENSG0000(	U73166.1	0.0708	0.3128	2.416862	0.007561	T
ENSG0000(	AC241520	0.1346	0.5835	2.91347	0.007563	T
ENSG0000(	AC012676	0.0864	0.2919	2.102468	0.007564	T
ENSG0000(	ARFRP1	1.3775	3.0196	2.111404	0.007579	T
ENSG0000(	SULT1C3	0.2885	0.0751	0.450708	0.007579	N
ENSG0000(	SATB2	0.2676	0.6726	2.101741	0.007586	T
ENSG0000(	AKR1E2	0.8944	2.5594	2.674377	0.007588	T
ENSG0000(	LINC0198(	0.01	1.7953	17.23	0.007591	T
ENSG0000(	GMEB2	2.6391	5.4024	2.008835	0.007595	T
ENSG0000(	HCP5	5.4949	14.9188	2.684373	0.007595	T
ENSG0000(	AC099336	4.9839	10.5423	2.093334	0.007597	T
ENSG0000(	GSTO1	11.0147	24.3463	2.199457	0.007597	T
ENSG0000(	ZNF197-A	0.1337	0.4783	2.47454	0.007599	T
ENSG0000(	RGS5	0.1702	0.5807	2.519245	0.0076	T
ENSG0000(	OFCC1	0.005	1.5221	15.44857	0.0076	T
ENSG0000(	ST3GAL2	0.6167	1.4417	2.151109	0.007605	T
ENSG0000(	AC005332	1.2027	3.079	2.440316	0.007607	T
ENSG0000(	NME2P1	0.6874	1.8037	2.417704	0.007621	T
ENSG0000(	CARD19	1.4141	2.9566	2.018757	0.007621	T
ENSG0000(	AC022400	1.5983	3.5746	2.163693	0.007624	T
ENSG0000(	RNASE2	0.0109	0.7903	8.027953	0.007642	T
ENSG0000(	ZNF101	3.7459	1.4527	0.403729	0.007642	N
ENSG0000(	C12orf65	0.5682	1.4223	2.27821	0.007661	T
ENSG0000(	DDIT3	1.6804	4.8212	2.764098	0.007672	T
ENSG0000(	AC009088	0.1762	0.512	2.215786	0.007673	T
ENSG0000(	SPATA20	2.1334	4.6754	2.138175	0.007676	T
ENSG0000(	PWP2	0.1323	0.365	2.001722	0.007677	T
ENSG0000(	TFAP2E	0.3784	1.003	2.305602	0.007689	T
ENSG0000(	SNORA36(	0.4189	1.2832	2.665639	0.007703	T

ENSG0000(	LIMCH1	3.4117	1.5854	0.479938	0.007714	N
ENSG0000(	AC087276	1.5535	4.7603	2.939401	0.007722	T
ENSG0000(	FHL2	3.0087	6.3764	2.083315	0.007729	T
ENSG0000(	RNF216-IT	0.2191	0.8319	2.920401	0.007731	T
ENSG0000(	AL358779.	0.1332	0.5405	2.746569	0.007738	T
ENSG0000(	MUC22	25.5015	2.0232	0.082933	0.007746	N
ENSG0000(	STARD4	1.0798	3.459	3.016613	0.007746	T
ENSG0000(	NLGN2	1.1875	3.7513	2.991301	0.007751	T
ENSG0000(	FANCG	1.5654	3.9671	2.442116	0.007751	T
ENSG0000(	AC026877	0.2118	0.6776	2.493906	0.007755	T
ENSG0000(	HTR2C	0	0.2268	3.268	0.007755	T
ENSG0000(	AC093495	0.1209	0.3655	2.107288	0.007763	T
ENSG0000(	EDN3	3.1933	0.4938	0.180305	0.007776	N
ENSG0000(	AC002558	0.2007	0.6482	2.488194	0.007779	T
ENSG0000(	IAH1	1.8299	3.8455	2.044406	0.007783	T
ENSG0000(	TMEM95	0.058	0.3457	2.820886	0.007788	T
ENSG0000(	MED12L	0.0802	0.5621	3.674251	0.007801	T
ENSG0000(	NPAS1	0.112	0.3925	2.323113	0.007814	T
ENSG0000(	RMI2	0.9015	3.3198	3.414678	0.007817	T
ENSG0000(	AC074367	0.0325	0.2717	2.805283	0.007818	T
ENSG0000(	AL355377.	0.056	0.2157	2.023718	0.007831	T
ENSG0000(	AL590133.	0.1169	0.4144	2.3716	0.007832	T
ENSG0000(	RF00003	8.56	34.0751	3.946316	0.007842	T
ENSG0000(	AC080013	0.0675	0.7024	4.790448	0.007848	T
ENSG0000(	ELF3	36.7613	17.5475	0.478754	0.007862	N
ENSG0000(	RF00003	3.0158	12.7177	4.113775	0.007864	T
ENSG0000(	ZNF880	1.3156	0.5769	0.478172	0.007868	N
ENSG0000(	HENMT1	0.6394	3.1742	4.428185	0.007871	T
ENSG0000(	SYCP2L	0.0322	0.6618	5.762481	0.007897	T
ENSG0000(	DDX39B-A	0.0912	0.3345	2.27249	0.007921	T
ENSG0000(	CYP4F60P	0.5368	0.0982	0.311244	0.007921	N
ENSG0000(	ARHGAP2	0.8539	3.0751	3.328546	0.007928	T
ENSG0000(	AC012676	0.2494	0.7959	2.56411	0.007933	T
ENSG0000(	AL445231.	0.1053	0.3332	2.110083	0.007939	T
ENSG0000(	AL390728.	0.0931	0.3016	2.079751	0.00794	T
ENSG0000(	POLE4	3.6447	9.2329	2.492296	0.007942	T
ENSG0000(	AC098828	0.0418	0.2455	2.43653	0.007942	T
ENSG0000(	SEC23A-A	0.1083	0.4652	2.713394	0.007952	T
ENSG0000(	ZC2HC1A	2.1785	5.7488	2.566952	0.007964	T
ENSG0000(	RNASEH1	2.3542	5.1219	2.12774	0.007969	T
ENSG0000(	MAP1LC3	10.334	4.1339	0.405779	0.007988	N
ENSG0000(	P3H2	2.5775	6.7768	2.568366	0.007989	T
ENSG0000(	AL732292.	0.0906	0.6214	3.78489	0.007991	T
ENSG0000(	RPL29P19	0.0577	0.706	5.11097	0.007994	T
ENSG0000(	AC010335	0.1552	0.7269	3.240204	0.007999	T
ENSG0000(	TAS2R30	0.3478	1.3913	3.330281	0.008006	T
ENSG0000(	NTAN1P2	0.3393	1.0405	2.596176	0.008007	T
ENSG0000(	AVIL	0.2165	0.5835	2.159558	0.008028	T
ENSG0000(	AL355488.	0.2957	1.1667	3.201162	0.008039	T
ENSG0000(	BGN	8.2515	23.7527	2.856098	0.008064	T
ENSG0000(	PRTFDC1	0.5447	3.6306	5.786567	0.008069	T
ENSG0000(	LINC0024:	0.0467	0.3094	2.790729	0.008077	T
ENSG0000(	MIR421	4.8327	14.3912	2.937783	0.00808	T
ENSG0000(	AP000320.	0.1425	0.5822	2.813196	0.00808	T
ENSG0000(	GNS	6.6019	14.0605	2.112908	0.008105	T
ENSG0000(	C19orf81	0.0961	0.4263	2.683835	0.008108	T
ENSG0000(	AL008718.	0.6618	2.5098	3.425834	0.008111	T
ENSG0000(	TMEM147	0.7132	1.6309	2.128505	0.008114	T

ENSG0000(	TIGD2	1.3898	3.0395	2.10733	0.008116	T
ENSG0000(	PTGES3P3	0.1972	0.7415	2.831427	0.008121	T
ENSG0000(	PGM2L1	0.3244	1.7215	4.291942	0.008124	T
ENSG0000(	AC097461	0.1264	0.512	2.70318	0.00814	T
ENSG0000(	MAPK12	0.0766	0.6251	4.105889	0.00814	T
ENSG0000(	NDUFB4P	0.2243	1.1386	3.819303	0.00814	T
ENSG0000(	NSUN5P1	0.5306	1.3896	2.362195	0.008141	T
ENSG0000(	SNORA11	2.1867	6.8732	3.04946	0.008143	T
ENSG0000(	TCF7L1	1.5473	3.2212	2.016148	0.008144	T
ENSG0000(	LINC0143	0.2147	0.7651	2.748967	0.008157	T
ENSG0000(	AC009145	0.0248	0.2249	2.603365	0.008168	T
ENSG0000(	AC008438	0.2535	0.8176	2.595757	0.008171	T
ENSG0000(	AC094085	0.0528	0.2192	2.089005	0.008176	T
ENSG0000(	HSPE1P28	0.8252	2.1841	2.468764	0.008177	T
ENSG0000(	SFXN3	1.2794	2.7267	2.049224	0.00818	T
ENSG0000(	MAP3K11	2.9299	6.3278	2.121456	0.008184	T
ENSG0000(	AC018766	0.0572	0.3275	2.719466	0.008196	T
ENSG0000(	HES6	0.1211	0.7673	3.922659	0.008196	T
ENSG0000(	REL	4.1843	8.8851	2.097215	0.008202	T
ENSG0000(	AL357033.	15.1643	1.4745	0.103149	0.008204	N
ENSG0000(	AC006270	0.1229	0.6078	3.175415	0.008205	T
ENSG0000(	LHX2	0.0052	0.3618	4.389734	0.008209	T
ENSG0000(	MAPK8IP2	0.605	1.4339	2.175745	0.008213	T
ENSG0000(	AL592295.	0.2402	0.8094	2.673133	0.008222	T
ENSG0000(	LINC0247	0.1325	0.9215	4.393548	0.00823	T
ENSG0000(	SCARNA7	144.8842	381.879	2.634625	0.008232	T
ENSG0000(	LINC0173	0.0214	0.4658	4.660626	0.008234	T
ENSG0000(	RTN4RL1	0.3146	0.9787	2.601785	0.008234	T
ENSG0000(	AC091185	0.4973	1.2423	2.247279	0.008244	T
ENSG0000(	SPIRE2	0.3143	0.9448	2.521844	0.008248	T
ENSG0000(	AC022211	0.517	1.3728	2.387034	0.00826	T
ENSG0000(	MIEF2	3.5743	1.6153	0.466837	0.008279	N
ENSG0000(	CSPG5	0.0499	0.2144	2.097398	0.008291	T
ENSG0000(	SNHG15	1.4885	4.0586	2.617941	0.008293	T
ENSG0000(	AL132780.	0.2226	0.7753	2.713267	0.008293	T
ENSG0000(	EMILIN2	0.3857	2.4172	5.182623	0.008303	T
ENSG0000(	SNORD53	1.7743	9.0501	4.881876	0.008311	T
ENSG0000(	RN7SL113	0.1337	0.4441	2.328199	0.008311	T
ENSG0000(	HNRNPA3	0.077	0.2926	2.218079	0.008312	T
ENSG0000(	ZFPM2-AS	0.0047	1.3382	13.73639	0.008317	T
ENSG0000(	AC009054	0.8423	2.0439	2.275178	0.008318	T
ENSG0000(	SLC18B1	4.2342	9.1551	2.135365	0.00833	T
ENSG0000(	AC015802	0.1254	0.3568	2.026619	0.008341	T
ENSG0000(	BMP2	1.6938	9.2451	5.209667	0.008343	T
ENSG0000(	AC090617	0.0756	0.669	4.379271	0.008347	T
ENSG0000(	GPLD1	0.2734	0.9155	2.719604	0.008348	T
ENSG0000(	NARF-IT1	0.0609	0.3285	2.663145	0.008351	T
ENSG0000(	PAK1	4.7425	14.0401	2.92	0.008356	T
ENSG0000(	AP005264.	0.7187	0.1094	0.255771	0.008375	N
ENSG0000(	AC010287	0.478	1.1469	2.157266	0.008385	T
ENSG0000(	AL355472.	0.1223	0.7963	4.031939	0.008392	T
ENSG0000(	AL356056.	0.2101	0.5352	2.048371	0.008392	T
ENSG0000(	GALC	1.2492	3.606	2.746813	0.0084	T
ENSG0000(	KRT80	46.4064	21.7732	0.470327	0.008419	N
ENSG0000(	AC002558	0.2391	0.8621	2.837216	0.008427	T
ENSG0000(	AP003419.	0.0925	0.5708	3.484675	0.00843	T
ENSG0000(	AC025575	0.0112	1.4126	13.60252	0.008444	T
ENSG0000(	AC244093	0.315	0.8793	2.359759	0.008447	T

ENSG0000(	TNNT1	1.0681	3.267	2.882459	0.008447	T
ENSG0000(	BX470102	0.208	0.8469	3.074351	0.008451	T
ENSG0000(	ATF3	4.8302	31.8655	6.483611	0.008461	T
ENSG0000(	ANKRD35	10.2035	4.6523	0.461232	0.008462	N
ENSG0000(	AC079807	0.3937	1.0551	2.33968	0.008477	T
ENSG0000(	GRPEL2	100.5503	21.374	0.213353	0.008477	N
ENSG0000(	MIR3682	1.5496	12.262	7.493938	0.00849	T
ENSG0000(	ZIC2	0.0784	2.796	16.23318	0.008491	T
ENSG0000(	H2AFZP2	0.0434	0.4246	3.658298	0.0085	T
ENSG0000(	DMPK	5.0892	2.4855	0.498246	0.00852	N
ENSG0000(	PLAC8	15.0196	3.7064	0.251753	0.008538	N
ENSG0000(	AC090912	0.3817	1.1912	2.680507	0.008541	T
ENSG0000(	IGF2BP2	0.224	7.5077	23.48056	0.008546	T
ENSG0000(	KIF26B	0.1154	0.6802	3.622098	0.008547	T
ENSG0000(	FAM69A	1.7721	4.2086	2.30148	0.00855	T
ENSG0000(	HSPE1P2	0.2699	1.2728	3.711273	0.008575	T
ENSG0000(	ADRA2B	0.0266	0.275	2.962085	0.008584	T
ENSG0000(	LINC0169	0.0389	0.2917	2.820014	0.008585	T
ENSG0000(	LBH	1.6626	5.7203	3.302111	0.008593	T
ENSG0000(	ABCC1	12.3769	38.3017	3.077824	0.008608	T
ENSG0000(	ZNRD1AS	0.1713	0.4446	2.007372	0.008615	T
ENSG0000(	CFHR3	0.0069	0.3708	4.404116	0.008616	T
ENSG0000(	AP000873.	0.2565	0.7832	2.477419	0.008619	T
ENSG0000(	IL18R1	0.5048	1.2358	2.208664	0.008624	T
ENSG0000(	AC016542	0.2971	1.5715	4.209267	0.008637	T
ENSG0000(	RF00151	0.2726	1.6134	4.598497	0.00864	T
ENSG0000(	RMND5A	46.8102	22.523	0.482262	0.008654	N
ENSG0000(	AC073089	0.0168	0.2024	2.589041	0.008655	T
ENSG0000(	AC017071	0.1365	0.4039	2.130655	0.008664	T
ENSG0000(	PRICKLE1	0.3467	0.9827	2.423774	0.008665	T
ENSG0000(	RNU6-134	0	0.6264	7.264	0.008666	T
ENSG0000(	ABHD2	6.4668	18.9259	2.897286	0.008667	T
ENSG0000(	ERO1A	252.593	50.4893	0.200201	0.00868	N
ENSG0000(	LILRB1	0.3061	1.5301	4.014036	0.008703	T
ENSG0000(	MIR3142H	0.1455	0.9675	4.348269	0.008716	T
ENSG0000(	AGMAT	0.1701	0.7334	3.085524	0.008721	T
ENSG0000(	PLEKHF1	7.7749	2.9048	0.381567	0.008741	N
ENSG0000(	AC092117	0.3161	0.7377	2.013218	0.008744	T
ENSG0000(	SELENOT	0.3249	1.3035	3.30313	0.008756	T
ENSG0000(	AC023790	0.0645	0.2448	2.096049	0.008757	T
ENSG0000(	AL354890.	0.2173	0.6914	2.49417	0.008762	T
ENSG0000(	MTND4P3	0.085	0.3376	2.365405	0.008764	T
ENSG0000(	LRRC70	0.051	0.2587	2.375497	0.008766	T
ENSG0000(	RN7SL20F	0.1257	0.4018	2.223305	0.008778	T
ENSG0000(	CNTF	0.2239	0.5926	2.138314	0.008787	T
ENSG0000(	KCNQ5	0.2049	0.7659	2.839948	0.008788	T
ENSG0000(	COL23A1	0.0422	0.3146	2.915612	0.008794	T
ENSG0000(	CTC-338M	0.391	1.0726	2.388187	0.0088	T
ENSG0000(	SLC4A11	3.0352	9.1942	2.964468	0.008804	T
ENSG0000(	AC007683	0.6821	1.8241	2.460171	0.008814	T
ENSG0000(	AL592295.	0.5571	1.333	2.180794	0.008814	T
ENSG0000(	AC025171	0.5159	1.1705	2.062835	0.008818	T
ENSG0000(	KCNJ2	1.6109	6.5926	3.911742	0.008831	T
ENSG0000(	HEY1	0.3341	3.2933	7.816862	0.008847	T
ENSG0000(	C6orf223	0.0024	0.2327	3.249023	0.008851	T
ENSG0000(	AC092821	0.0489	0.2371	2.263936	0.008865	T
ENSG0000(	CXCL1	0.8235	94.4772	102.4117	0.008887	T
ENSG0000(	RBM38	3.8379	8.4941	2.182407	0.008888	T

ENSG0000(	ZNFX1	4.5992	11.932	2.560436	0.008899	T
ENSG0000(	TAS2R64F	0.1797	1.17	4.540579	0.008901	T
ENSG0000(	RNU6-42P	0.4752	2.3582	4.273644	0.008905	T
ENSG0000(	IL17RB	0.0652	0.3671	2.827482	0.008917	T
ENSG0000(	FOXN2	3.2534	13.6459	4.099093	0.008918	T
ENSG0000(	LINC0143:	0.0304	0.2596	2.757669	0.008929	T
ENSG0000(	AC080013	0.0432	0.7959	6.256285	0.00893	T
ENSG0000(	PKD1P6	0.6918	1.4865	2.003663	0.008931	T
ENSG0000(	LRRFIP1P	0.3695	1.2716	2.921406	0.008944	T
ENSG0000(	B3GNTL1	0.2307	0.7076	2.442093	0.008947	T
ENSG0000(	TRIB1	15.9878	42.4486	2.644774	0.008954	T
ENSG0000(	C17orf113	0.0968	0.3628	2.351626	0.008984	T
ENSG0000(	AP000676.	0.0704	0.3476	2.626761	0.008988	T
ENSG0000(	RNU6-195	0.4945	2.8626	4.983347	0.008997	T
ENSG0000(	TAS1R3	2.1385	0.2531	0.15774	0.009007	N
ENSG0000(	EPHX1	37.8618	94.821	2.500435	0.009019	T
ENSG0000(	ASNS	0.0821	0.2717	2.041186	0.009021	T
ENSG0000(	AL359918.	0.1951	0.7449	2.863097	0.009026	T
ENSG0000(	RPL5P16	0	0.2808	3.808	0.009042	T
ENSG0000(	RTEL1	0.0754	0.2552	2.025086	0.009044	T
ENSG0000(	EIF4A1P7	0.0963	0.3096	2.086602	0.009045	T
ENSG0000(	AC037198	0.2581	2.8977	8.371125	0.009049	T
ENSG0000(	SUMO2P6	0.2124	0.5746	2.159411	0.009051	T
ENSG0000(	TTYH2	0.3692	1.1596	2.684569	0.009057	T
ENSG0000(	AL121906.	0.1746	0.6346	2.675164	0.009063	T
ENSG0000(	FUNDC2P	0.1223	0.5535	2.939721	0.00907	T
ENSG0000(	RF00561	2.5243	5.9521	2.306177	0.009071	T
ENSG0000(	CNBD2	0.1681	0.4587	2.083924	0.009071	T
ENSG0000(	AP002387.	0.4436	1.7904	3.477557	0.009077	T
ENSG0000(	LINC0152:	0.112	0.36	2.169811	0.009084	T
ENSG0000(	LRRC73	0.0828	0.2967	2.170131	0.009093	T
ENSG0000(	MIR548A/	0.0832	1.8019	10.38155	0.009112	T
ENSG0000(	FAM174B	2.6746	1.1088	0.435666	0.009141	N
ENSG0000(	PARVB	0.3457	1.0599	2.602423	0.009146	T
ENSG0000(	RF00569	2.7544	12.3955	4.377628	0.009173	T
ENSG0000(	TCEA1P4	0.1632	0.5761	2.568769	0.009187	T
ENSG0000(	MIR99B	0.917	2.8459	2.896657	0.009191	T
ENSG0000(	AC083880	0.3541	1.107	2.658005	0.009202	T
ENSG0000(	FAM96AP	0.3564	2.3616	5.393514	0.009208	T
ENSG0000(	YWHAQP	0.0318	0.1826	2.144158	0.009211	T
ENSG0000(	CBX3P9	0.4386	2.0386	3.970665	0.009219	T
ENSG0000(	B3GALNT	1.6221	4.0626	2.417165	0.009221	T
ENSG0000(	AL138963.	0.4975	1.4296	2.56	0.009223	T
ENSG0000(	MCTP1	0.3022	1.3292	3.553456	0.009231	T
ENSG0000(	AC009078	0.117	0.373	2.179724	0.009236	T
ENSG0000(	PRSS8	59.4041	16.9508	0.286548	0.009237	N
ENSG0000(	TMEM79	50.5569	24.6984	0.489536	0.00924	N
ENSG0000(	CD99L2	1.3249	2.9027	2.107306	0.00925	T
ENSG0000(	AC087500	0.2237	0.5951	2.147359	0.009261	T
ENSG0000(	ASH1L-IT	0.1629	0.6734	2.941803	0.009263	T
ENSG0000(	CD151	7.6535	17.5918	2.281782	0.009267	T
ENSG0000(	OR7E100P	0.0821	0.3266	2.342669	0.009275	T
ENSG0000(	PLA2R1	1.9006	3.9215	2.010147	0.009289	T
ENSG0000(	KRTAP5-1	0.0073	0.2167	2.951538	0.009297	T
ENSG0000(	SLC9A2	0.3245	1.6479	4.11755	0.009303	T
ENSG0000(	MIR548T	0.1128	1.1497	5.87265	0.009309	T
ENSG0000(	AC068025	0.0422	0.2131	2.201828	0.009316	T
ENSG0000(	GTF2IP23	1.376	3.1233	2.183808	0.009318	T

ENSG0000(	GCC2-AS1	0.0954	0.5082	3.11259	0.009328	T
ENSG0000(	LINC0120(	0.5157	0.1713	0.440637	0.009337	N
ENSG0000(	AC016597	0.1463	0.5131	2.489241	0.009337	T
ENSG0000(	ZNF101P2	0.1398	0.5294	2.624687	0.009338	T
ENSG0000(	VPS16	2.6898	5.5063	2.009571	0.009356	T
ENSG0000(	PDPN	1.2946	10.6833	7.732181	0.009363	T
ENSG0000(	AC106791	0.5811	1.7533	2.721039	0.009366	T
ENSG0000(	RF01169	2.0102	14.4899	6.913989	0.009371	T
ENSG0000(	AC025198	0.1241	0.3854	2.165997	0.009376	T
ENSG0000(	UBE2SP1	0.2673	0.9108	2.751974	0.009377	T
ENSG0000(	CDK5RAP	0.3012	0.9732	2.674975	0.009391	T
ENSG0000(	TDRD5	0.1294	2.0431	9.342197	0.009405	T
ENSG0000(	PGAM1P1	0.0295	0.1952	2.279537	0.009411	T
ENSG0000(	AC005921	0.3262	1.3195	3.330596	0.009412	T
ENSG0000(	RF00019	0.0254	0.3928	3.929825	0.00942	T
ENSG0000(	FAM86HP	0.5386	2.0875	3.425462	0.009426	T
ENSG0000(	AC011611	1.8272	0.1458	0.127543	0.009439	N
ENSG0000(	TEAD4	1.0451	5.5447	4.929438	0.009455	T
ENSG0000(	SUMF2	7.7572	16.3763	2.096968	0.009461	T
ENSG0000(	SCAMP1-/	0.5647	1.6201	2.587784	0.009462	T
ENSG0000(	AL139393.	1.55	0.4754	0.348727	0.009468	N
ENSG0000(	LINC0064:	1.1243	2.7464	2.32492	0.009475	T
ENSG0000(	AL596220.	0.3024	1.1161	3.022117	0.009482	T
ENSG0000(	AP003500.	2.958	0.7149	0.266481	0.009485	N
ENSG0000(	SNORA28	15.9611	32.5049	2.030054	0.009486	T
ENSG0000(	MORF4L1	0.1197	0.4274	2.400546	0.009487	T
ENSG0000(	RNU6-72P	0.6237	2.482	3.567777	0.009502	T
ENSG0000(	AC134043	0.0604	0.6332	4.571072	0.009509	T
ENSG0000(	RSF1-IT2	0.1726	0.8714	3.563463	0.009515	T
ENSG0000(	FEZ1	0.1574	0.5413	2.491453	0.009518	T
ENSG0000(	LINC0135!	0.3987	1.2266	2.660116	0.009519	T
ENSG0000(	AC010680	0.365	0.9219	2.197634	0.009537	T
ENSG0000(	AL031777.	1.9809	0.7904	0.427892	0.009539	N
ENSG0000(	SLC19A1	0.167	0.9113	3.78764	0.009543	T
ENSG0000(	RPP21	0.1384	0.4349	2.243708	0.009553	T
ENSG0000(	AL132780.	0.0912	0.353	2.369247	0.009556	T
ENSG0000(	PRKCA	0.8194	1.7531	2.015554	0.009613	T
ENSG0000(	RNU6-196	0.7492	2.6181	3.200777	0.009623	T
ENSG0000(	UGT8	0.6725	2.6684	3.583689	0.009623	T
ENSG0000(	AL139241.	0.1329	0.4792	2.486904	0.009641	T
ENSG0000(	PLA2G15	0.7501	1.7743	2.204799	0.009645	T
ENSG0000(	MIR2052	0	1.7272	18.272	0.009648	T
ENSG0000(	AL139184.	0.0324	0.2237	2.444864	0.009648	T
ENSG0000(	AC009093	0.0321	0.2878	2.935655	0.009655	T
ENSG0000(	CD14	2.513	13.7331	5.293953	0.00967	T
ENSG0000(	CBX6	3.6833	9.174	2.451299	0.009671	T
ENSG0000(	AC092045	0.1273	0.4623	2.473823	0.009679	T
ENSG0000(	SNORD70	0.4889	2.8389	4.990491	0.009685	T
ENSG0000(	DCUN1D5	6.3101	55.7608	8.714497	0.009696	T
ENSG0000(	AL162615.	0.1545	0.5934	2.724558	0.009706	T
ENSG0000(	AC118754	2.313	0.4341	0.221343	0.009707	N
ENSG0000(	AC118658	0.3245	1.1918	3.04311	0.009717	T
ENSG0000(	FAM24B	0.2379	0.6406	2.191773	0.009728	T
ENSG0000(	CERNA3	0.0207	0.2403	2.819387	0.009731	T
ENSG0000(	CLEC2B	5.2086	13.837	2.625363	0.009732	T
ENSG0000(	TFEC	0.1412	0.7081	3.350332	0.009735	T
ENSG0000(	RPL7P15	0.4883	1.1719	2.161992	0.009737	T
ENSG0000(	LINC0232:	0	0.5467	6.467	0.009742	T

ENSG0000(	AP000251.	0.188	1.0774	4.088194	0.009749	T
ENSG0000(	RNU6-32P	0.182	1.7265	6.47695	0.009756	T
ENSG0000(	AL161669.	0	0.2204	3.204	0.009771	T
ENSG0000(	HS3ST6	14.4304	4.3425	0.305738	0.00978	N
ENSG0000(	CSF1	1.288	3.6138	2.675648	0.00981	T
ENSG0000(	SLC39A13	1.1566	2.4368	2.018781	0.009812	T
ENSG0000(	RF00019	1.1577	4.2141	3.43015	0.009816	T
ENSG0000(	GOLGA8E	0.6919	2.2996	3.030181	0.009835	T
ENSG0000(	ANKRD36	0.966	3.8775	3.731238	0.009838	T
ENSG0000(	PNPLA3	0.0829	0.5534	3.572444	0.00985	T
ENSG0000(	AC025161	0.126	0.7883	3.930531	0.009877	T
ENSG0000(	DLEU7-AS	0.0429	0.5477	4.53254	0.009889	T
ENSG0000(	MIR3188	0.3569	1.411	3.307069	0.009897	T
ENSG0000(	RN7SKP13	0.2099	0.9583	3.414973	0.009902	T
ENSG0000(	ZNF692	1.544	3.8022	2.373601	0.009915	T
ENSG0000(	GSR	17.2135	36.9054	2.137373	0.009922	T
ENSG0000(	AC013489	0.0293	0.1716	2.100541	0.009933	T
ENSG0000(	LINC0158'	0.4762	0.0359	0.235856	0.009934	N
ENSG0000(	LINC0154'	0.2248	0.5583	2.026786	0.009936	T
ENSG0000(	ULBP1	0.0715	0.6159	4.174344	0.009978	T
ENSG0000(	RRM2P3	0.0851	0.4649	3.051864	0.009989	T
ENSG0000(	TGFBI	2.9383	13.4029	4.444229	0.00999	T
ENSG0000(	PARP10	1.4131	4.1533	2.810984	0.00999	T
ENSG0000(	AC016700	0.2265	0.9322	3.161409	0.009996	T
ENSG0000(	AC026250	0.0609	0.2442	2.139217	0.009998	T
