# Supplementary material for: Genomic and transcriptional characterization of early esophageal squamous cell carcinoma
Source: BMC Med Genomics. 2023 Jul 1;16:153. doi: 10.1186/s12920-023-01588-7 (PMC10315050; doi:10.1186/s12920-023-01588-7)
Supplement: Supplementary file 4 — Additional file 4: TableS3. Clinical data of the patients in this study. Table S4. TCGA samples used in the study. Table S5. Primers and RNA sequences used in this study. [file 12920_2023_1588_MOESM4_ESM.docx]

| Table S3: Clinical data of the patients in this study | | | |  |  |
| --- | --- | --- | --- | --- | --- |
| Patient ID | Tumor tissue | NT tissue | Sex | Age | Stage |
| 1 | ESCA01T | ESCA01N | M | 55 | Stage I |
| 7 | ESCA07T | ESCA07N | M | 64 | Stage I |
| 10 | ESCA10T | ESCA10N | F | 67 | Stage I |
| 14 | ESCA14T | ESCA14N | M | 38 | Stage I |
| 15 | ESCA15T | ESCA15N | F | 62 | Stage I |
| 16 | ESCA16T | ESCA16N | M | 57 | Stage I |
| 17 | ESCA17T | ESCA17N | M | 55 | Stage I |
| 18 | ESCA18T | ESCA18N | M | 75 | Stage I |
| 19 | ESCA19T | ESCA19N | M | 60 | Stage I |
| 20 | ESCA20T | ESCA20N | M | 69 | Stage I |

| Table S4: TCGA samples used in the study | | |  |
| --- | --- | --- | --- |
| SAMPLE_ID | PATIENT_ID | AJCC_PATHOLOGIC_TUMOR_STAGE | ONCOTREE_CODE |
| TCGA-2H-A9GF-01A | TCGA-2H-A9GF | Stage III | ESCA |
| TCGA-2H-A9GH-01A | TCGA-2H-A9GH | Stage IIB | ESCA |
| TCGA-2H-A9GI-01A | TCGA-2H-A9GI | Stage III | ESCA |
| TCGA-2H-A9GJ-01A | TCGA-2H-A9GJ | Stage I | ESCA |
| TCGA-2H-A9GK-01A | TCGA-2H-A9GK | Stage III | ESCA |
| TCGA-2H-A9GL-01A | TCGA-2H-A9GL | Stage III | ESCA |
| TCGA-2H-A9GM-01A | TCGA-2H-A9GM | Stage IIB | ESCA |
| TCGA-2H-A9GN-01A | TCGA-2H-A9GN | Stage III | ESCA |
| TCGA-2H-A9GO-01A | TCGA-2H-A9GO | Stage IVA | ESCA |
| TCGA-2H-A9GQ-01A | TCGA-2H-A9GQ | Stage III | ESCA |
| TCGA-2H-A9GR-01A | TCGA-2H-A9GR | Stage IIA | ESCA |
| TCGA-IC-A6RE-01A | TCGA-IC-A6RE | Stage IIB | ESCA |
| TCGA-IC-A6RF-01A | TCGA-IC-A6RF | Stage IA | ESCC |
| TCGA-IG-A3I8-01A | TCGA-IG-A3I8 | Stage IIA | ESCC |
| TCGA-IG-A3QL-01A | TCGA-IG-A3QL | Stage IIA | ESCC |
| TCGA-IG-A3Y9-01A | TCGA-IG-A3Y9 | Stage IIIA | ESCC |
| TCGA-IG-A3YA-01A | TCGA-IG-A3YA | Stage IIIA | ESCC |
| TCGA-IG-A3YB-01A | TCGA-IG-A3YB | Stage IIIA | ESCC |
| TCGA-IG-A3YC-01A | TCGA-IG-A3YC | Stage IIIA | ESCC |
| TCGA-IG-A4P3-01A | TCGA-IG-A4P3 | Stage IIB | ESCC |
| TCGA-IG-A4QS-01A | TCGA-IG-A4QS | Stage IIIB | ESCA |
| TCGA-IG-A4QT-01A | TCGA-IG-A4QT | Stage IIA | ESCC |
| TCGA-IG-A50L-01A | TCGA-IG-A50L | Stage IIIA | ESCC |
| TCGA-IG-A51D-01A | TCGA-IG-A51D | Stage IIB | ESCC |
| TCGA-IG-A5B8-01A | TCGA-IG-A5B8 | Stage IB | ESCC |
| TCGA-IG-A5S3-01A | TCGA-IG-A5S3 | Stage IIB | ESCC |
| TCGA-IG-A625-01A | TCGA-IG-A625 | Stage IIIB | ESCC |
| TCGA-IG-A6QS-01A | TCGA-IG-A6QS | Stage IIB | ESCC |
| TCGA-IG-A7DP-01A | TCGA-IG-A7DP | Stage IIIA | ESCA |
| TCGA-IG-A8O2-01A | TCGA-IG-A8O2 | Stage IIIB | ESCC |
| TCGA-IG-A97H-01A | TCGA-IG-A97H | Stage IIA | ESCC |
| TCGA-IG-A97I-01A | TCGA-IG-A97I | Stage IIA | ESCC |
| TCGA-JY-A6F8-01A | TCGA-JY-A6F8 | Stage I | ESCA |
| TCGA-JY-A6FA-01A | TCGA-JY-A6FA | Stage IIB | ESCC |
| TCGA-JY-A6FB-01A | TCGA-JY-A6FB | Stage I | ESCA |
| TCGA-JY-A6FD-01A | TCGA-JY-A6FD | Stage IIA | ESCC |
| TCGA-JY-A6FE-01A | TCGA-JY-A6FE | Stage III | ESCC |
| TCGA-JY-A6FG-01A | TCGA-JY-A6FG | Stage III | ESCC |
| TCGA-JY-A6FH-01A | TCGA-JY-A6FH | Stage IIB | ESCA |
| TCGA-JY-A938-01A | TCGA-JY-A938 | Stage IIB | ESCA |
| TCGA-JY-A939-01A | TCGA-JY-A939 | Stage IIB | ESCA |
| TCGA-JY-A93C-01A | TCGA-JY-A93C | Stage IIIB | ESCA |
| TCGA-JY-A93D-01A | TCGA-JY-A93D | Stage IIIC | ESCA |
| TCGA-JY-A93E-01A | TCGA-JY-A93E | Stage IIIA | ESCA |
| TCGA-JY-A93F-01A | TCGA-JY-A93F | Stage IB | ESCC |
| TCGA-KH-A6WC-01A | TCGA-KH-A6WC | Stage IA | ESCC |
| TCGA-L5-A43C-01A | TCGA-L5-A43C | [Not Available] | ESCA |
| TCGA-L5-A43E-01A | TCGA-L5-A43E | Stage I | ESCA |
| TCGA-L5-A43H-01A | TCGA-L5-A43H | Stage III | ESCC |
| TCGA-L5-A43I-01A | TCGA-L5-A43I | Stage IIIA | ESCA |
| TCGA-L5-A43J-01A | TCGA-L5-A43J | Stage IIB | ESCC |
| TCGA-L5-A43M-01A | TCGA-L5-A43M | [Not Available] | ESCA |
| TCGA-L5-A4OE-01A | TCGA-L5-A4OE | Stage IIIB | ESCA |
| TCGA-L5-A4OF-01A | TCGA-L5-A4OF | Stage IIB | ESCA |
| TCGA-L5-A4OG-01A | TCGA-L5-A4OG | Stage I | ESCA |
| TCGA-L5-A4OH-01A | TCGA-L5-A4OH | Stage I | ESCA |
| TCGA-L5-A4OI-01A | TCGA-L5-A4OI | Stage IIIC | ESCA |
| TCGA-L5-A4OJ-01A | TCGA-L5-A4OJ | Stage I | ESCA |
| TCGA-L5-A4OM-01A | TCGA-L5-A4OM | Stage IA | ESCC |
| TCGA-L5-A4ON-01A | TCGA-L5-A4ON | Stage IIB | ESCA |
| TCGA-L5-A4OO-01A | TCGA-L5-A4OO | Stage IIIC | ESCA |
| TCGA-L5-A4OP-01A | TCGA-L5-A4OP | Stage IA | ESCA |
| TCGA-L5-A4OQ-01A | TCGA-L5-A4OQ | Stage IIIA | ESCA |
| TCGA-L5-A4OR-01A | TCGA-L5-A4OR | Stage IA | ESCA |
| TCGA-L5-A4OS-01A | TCGA-L5-A4OS | Stage IIB | ESCA |
| TCGA-L5-A4OT-01A | TCGA-L5-A4OT | Stage IV | ESCA |
| TCGA-L5-A4OU-01A | TCGA-L5-A4OU | Stage IIA | ESCA |
| TCGA-L5-A4OW-01A | TCGA-L5-A4OW | Stage IIB | ESCA |
| TCGA-L5-A4OX-01A | TCGA-L5-A4OX | Stage IIB | ESCA |
| TCGA-L5-A88S-01A | TCGA-L5-A88S | Stage IB | ESCC |
| TCGA-L5-A88T-01A | TCGA-L5-A88T | Stage IIB | ESCA |
| TCGA-L5-A88V-01A | TCGA-L5-A88V | Stage III | ESCA |
| TCGA-L5-A88W-01A | TCGA-L5-A88W | Stage IIA | ESCC |
| TCGA-L5-A88Y-01A | TCGA-L5-A88Y | [Not Available] | ESCA |
| TCGA-L5-A88Z-01A | TCGA-L5-A88Z | Stage IIA | ESCC |
| TCGA-L5-A891-01A | TCGA-L5-A891 | [Not Available] | ESCA |
| TCGA-L5-A893-01A | TCGA-L5-A893 | Stage I | ESCA |
| TCGA-L5-A8NE-01A | TCGA-L5-A8NE | Stage IIB | ESCA |
| TCGA-L5-A8NF-01A | TCGA-L5-A8NF | Stage IVA | ESCA |
| TCGA-L5-A8NG-01A | TCGA-L5-A8NG | Stage III | ESCA |
| TCGA-L5-A8NH-01A | TCGA-L5-A8NH | Stage IV | ESCA |
| TCGA-L5-A8NI-01A | TCGA-L5-A8NI | Stage III | ESCA |
| TCGA-L5-A8NJ-01A | TCGA-L5-A8NJ | Stage III | ESCA |
| TCGA-L5-A8NK-01A | TCGA-L5-A8NK | Stage IIA | ESCC |
| TCGA-L5-A8NL-01A | TCGA-L5-A8NL | Stage III | ESCA |
| TCGA-L5-A8NM-01A | TCGA-L5-A8NM | Stage IIB | ESCA |
| TCGA-L5-A8NN-01A | TCGA-L5-A8NN | Stage III | ESCA |
| TCGA-L5-A8NQ-01A | TCGA-L5-A8NQ | Stage IIA | ESCC |
| TCGA-L5-A8NR-01A | TCGA-L5-A8NR | Stage III | ESCA |
| TCGA-L5-A8NS-01A | TCGA-L5-A8NS | Stage IIB | ESCA |
| TCGA-L5-A8NT-01A | TCGA-L5-A8NT | Stage IIB | ESCA |
| TCGA-L5-A8NU-01A | TCGA-L5-A8NU | Stage IIA | ESCA |
| TCGA-L5-A8NV-01A | TCGA-L5-A8NV | Stage IIA | ESCA |
| TCGA-L5-A8NW-01A | TCGA-L5-A8NW | [Discrepancy] | ESCA |
| TCGA-L7-A56G-01A | TCGA-L7-A56G | [Not Available] | ESCC |
| TCGA-L7-A6VZ-01A | TCGA-L7-A6VZ | Stage IIIC | ESCA |
| TCGA-LN-A49K-01A | TCGA-LN-A49K | Stage IIA | ESCC |
| TCGA-LN-A49L-01A | TCGA-LN-A49L | Stage IIA | ESCC |
| TCGA-LN-A49M-01A | TCGA-LN-A49M | Stage IIA | ESCC |
| TCGA-LN-A49N-01A | TCGA-LN-A49N | Stage IIB | ESCC |
| TCGA-LN-A49O-01A | TCGA-LN-A49O | Stage IIA | ESCC |
| TCGA-LN-A49P-01A | TCGA-LN-A49P | Stage IIA | ESCC |
| TCGA-LN-A49R-01A | TCGA-LN-A49R | Stage III | ESCC |
| TCGA-LN-A49S-01A | TCGA-LN-A49S | Stage IIA | ESCC |
| TCGA-LN-A49U-01A | TCGA-LN-A49U | Stage IIA | ESCC |
| TCGA-LN-A49V-01A | TCGA-LN-A49V | Stage IIA | ESCC |
| TCGA-LN-A49W-01A | TCGA-LN-A49W | Stage III | ESCC |
| TCGA-LN-A49X-01A | TCGA-LN-A49X | Stage IIA | ESCC |
| TCGA-LN-A49Y-01A | TCGA-LN-A49Y | Stage IIA | ESCC |
| TCGA-LN-A4A1-01A | TCGA-LN-A4A1 | Stage IIA | ESCC |
| TCGA-LN-A4A2-01A | TCGA-LN-A4A2 | Stage IIA | ESCC |
| TCGA-LN-A4A3-01A | TCGA-LN-A4A3 | Stage III | ESCC |
| TCGA-LN-A4A4-01A | TCGA-LN-A4A4 | Stage III | ESCC |
| TCGA-LN-A4A5-01A | TCGA-LN-A4A5 | Stage IIA | ESCC |
| TCGA-LN-A4A6-01A | TCGA-LN-A4A6 | Stage II | ESCC |
| TCGA-LN-A4A8-01A | TCGA-LN-A4A8 | Stage IIA | ESCC |
| TCGA-LN-A4A9-01A | TCGA-LN-A4A9 | Stage IIA | ESCC |
| TCGA-LN-A4MQ-01A | TCGA-LN-A4MQ | Stage III | ESCC |
| TCGA-LN-A4MR-01A | TCGA-LN-A4MR | Stage IIA | ESCC |
| TCGA-LN-A5U5-01A | TCGA-LN-A5U5 | Stage IV | ESCC |
| TCGA-LN-A5U6-01A | TCGA-LN-A5U6 | Stage IIB | ESCC |
| TCGA-LN-A5U7-01A | TCGA-LN-A5U7 | Stage IIA | ESCC |
| TCGA-LN-A7HV-01A | TCGA-LN-A7HV | Stage IIA | ESCC |
| TCGA-LN-A7HW-01A | TCGA-LN-A7HW | Stage IIA | ESCC |
| TCGA-LN-A7HX-01A | TCGA-LN-A7HX | Stage IIA | ESCC |
| TCGA-LN-A7HY-01A | TCGA-LN-A7HY | Stage III | ESCC |
| TCGA-LN-A7HZ-01A | TCGA-LN-A7HZ | Stage IIA | ESCC |
| TCGA-LN-A8HZ-01A | TCGA-LN-A8HZ | Stage IIA | ESCC |
| TCGA-LN-A8I0-01A | TCGA-LN-A8I0 | Stage IIA | ESCC |
| TCGA-LN-A8I1-01A | TCGA-LN-A8I1 | Stage IIA | ESCC |
| TCGA-LN-A9FO-01A | TCGA-LN-A9FO | Stage IIA | ESCC |
| TCGA-LN-A9FP-01A | TCGA-LN-A9FP | Stage IIA | ESCC |
| TCGA-LN-A9FQ-01A | TCGA-LN-A9FQ | Stage IIA | ESCC |
| TCGA-LN-A9FR-01A | TCGA-LN-A9FR | Stage IIB | ESCC |
| TCGA-M9-A5M8-01A | TCGA-M9-A5M8 | Stage IIA | ESCA |
| TCGA-Q9-A6FU-01A | TCGA-Q9-A6FU | Stage IIIB | ESCC |
| TCGA-Q9-A6FW-01A | TCGA-Q9-A6FW | Stage IIIB | ESCA |
| TCGA-R6-A6DN-01B | TCGA-R6-A6DN | [Not Available] | ESCA |
| TCGA-R6-A6DQ-01B | TCGA-R6-A6DQ | [Not Available] | ESCA |
| TCGA-R6-A6KZ-01A | TCGA-R6-A6KZ | [Not Available] | ESCA |
| TCGA-R6-A6L4-01A | TCGA-R6-A6L4 | [Not Available] | ESCA |
| TCGA-R6-A6L6-01B | TCGA-R6-A6L6 | [Not Available] | ESCA |
| TCGA-R6-A6XG-01B | TCGA-R6-A6XG | [Not Available] | ESCA |
| TCGA-R6-A6XQ-01B | TCGA-R6-A6XQ | [Not Available] | ESCA |
| TCGA-R6-A6Y0-01B | TCGA-R6-A6Y0 | [Not Available] | ESCA |
| TCGA-R6-A6Y2-01B | TCGA-R6-A6Y2 | [Not Available] | ESCA |
| TCGA-R6-A8W5-01B | TCGA-R6-A8W5 | Stage IVA | ESCA |
| TCGA-R6-A8W8-01B | TCGA-R6-A8W8 | [Not Available] | ESCA |
| TCGA-R6-A8WC-01A | TCGA-R6-A8WC | [Not Available] | ESCA |
| TCGA-R6-A8WG-01A | TCGA-R6-A8WG | [Not Available] | ESCA |
| TCGA-RE-A7BO-01A | TCGA-RE-A7BO | Stage IIB | ESCA |
| TCGA-S8-A6BV-01A | TCGA-S8-A6BV | Stage IIIA | ESCA |
| TCGA-S8-A6BW-01A | TCGA-S8-A6BW | Stage IB | ESCC |
| TCGA-V5-A7RB-01A | TCGA-V5-A7RB | [Not Available] | ESCA |
| TCGA-V5-A7RC-01B | TCGA-V5-A7RC | [Not Available] | ESCC |
| TCGA-V5-A7RE-01A | TCGA-V5-A7RE | Stage IB | ESCA |
| TCGA-V5-AASV-01A | TCGA-V5-AASV | Stage IIB | ESCC |
| TCGA-V5-AASW-01A | TCGA-V5-AASW | [Not Available] | ESCA |
| TCGA-V5-AASX-01A | TCGA-V5-AASX | [Not Available] | ESCA |
| TCGA-VR-A8EO-01A | TCGA-VR-A8EO | Stage IIA | ESCC |
| TCGA-VR-A8EP-01A | TCGA-VR-A8EP | Stage IIIB | ESCC |
| TCGA-VR-A8EQ-01A | TCGA-VR-A8EQ | Stage III | ESCA |
| TCGA-VR-A8ER-01A | TCGA-VR-A8ER | Stage III | ESCC |
| TCGA-VR-A8ET-01A | TCGA-VR-A8ET | Stage IIA | ESCC |
| TCGA-VR-A8EU-01A | TCGA-VR-A8EU | Stage IV | ESCC |
| TCGA-VR-A8EW-01A | TCGA-VR-A8EW | Stage IIIB | ESCC |
| TCGA-VR-A8EX-01A | TCGA-VR-A8EX | Stage IVA | ESCC |
| TCGA-VR-A8EY-01A | TCGA-VR-A8EY | Stage IIA | ESCC |
| TCGA-VR-A8EZ-01A | TCGA-VR-A8EZ | Stage IIIC | ESCC |
| TCGA-VR-A8Q7-01A | TCGA-VR-A8Q7 | Stage IIIA | ESCC |
| TCGA-VR-AA4D-01A | TCGA-VR-AA4D | Stage IIB | ESCA |
| TCGA-VR-AA4G-01A | TCGA-VR-AA4G | Stage IIIA | ESCC |
| TCGA-VR-AA7B-01A | TCGA-VR-AA7B | Stage IV | ESCC |
| TCGA-VR-AA7D-01A | TCGA-VR-AA7D | Stage IIIC | ESCC |
| TCGA-VR-AA7I-01A | TCGA-VR-AA7I | Stage III | ESCC |
| TCGA-X8-AAAR-01A | TCGA-X8-AAAR | [Not Available] | ESCA |
| TCGA-XP-A8T6-01A | TCGA-XP-A8T6 | Stage IIB | ESCC |
| TCGA-XP-A8T7-01A | TCGA-XP-A8T7 | Stage IIA | ESCC |
| TCGA-XP-A8T8-01A | TCGA-XP-A8T8 | Stage IIB | ESCC |
| TCGA-Z6-A8JD-01A | TCGA-Z6-A8JD | Stage IIB | ESCC |
| TCGA-Z6-A8JE-01A | TCGA-Z6-A8JE | Stage IIIA | ESCC |
| TCGA-Z6-A9VB-01A | TCGA-Z6-A9VB | Stage IIIA | ESCC |
| TCGA-Z6-AAPN-01A | TCGA-Z6-AAPN | Stage IIA | ESCC |
| TCGA-ZR-A9CJ-01B | TCGA-ZR-A9CJ | Stage IIIC | ESCA |
| TCGA-L5-A4OR-11A | TCGA-L5-A4OR | NA | normal |
| TCGA-L5-A4OG-11A | TCGA-L5-A4OG | NA | normal |
| TCGA-L5-A4OQ-11A | TCGA-L5-A4OQ | NA | normal |
| TCGA-IC-A6RF-11A | TCGA-IC-A6RF | NA | normal |
| TCGA-L5-A4OJ-11A | TCGA-L5-A4OJ | NA | normal |
| TCGA-IC-A6RE-11A | TCGA-IC-A6RE | NA | normal |
| TCGA-V5-A7RE-11A | TCGA-V5-A7RE | NA | normal |
| TCGA-V5-AASX-11A | TCGA-V5-AASX | NA | normal |
| TCGA-L5-A4OF-11A | TCGA-L5-A4OF | NA | normal |
| TCGA-L5-A4OO-11A | TCGA-L5-A4OO | NA | normal |
| TCGA-L5-A43C-11A | TCGA-L5-A43C | NA | normal |

| Table S5. Primers and RNA sequences used in this study | | |
| --- | --- | --- |
| mRNA | Forward primer(5'-3') | Reverse primer(5'-3') |
| HOXC10 | CTATCCGTCCTACCTCTCGCA | CCTGCCAACAGGTTGTTCC |
| HOXC11 | AGTTGCACTTACTACATGCCC | GGCCGAGTAGGGATAGGAGA |
| HOXB7 | CGAGTTCCTTCAACATGCACT | TTTGCGGTCAGTTCCTGAGC |
| HOXA7 | CGTTCCGGGCTTATACAATGT | CTCGTCCGTCTTGTCGCAG |
| HOXD8 | GGAAGACAAACCTACAGTCGC | TCCTGGTCAGATAGGGGTTAAAA |
| HOXD10 | GACATGGGGACCTATGGAATGC | CGGATCTGTCCAACTGTCTACT |
| HOXD9 | GGACTCGCTTATAGGCCATGA | GCAAAACTACACGAGGCGAA |
| HOXC9 | ACTCGCTCATCTCTCACGACA | GACGGAAAATCGCTACAGTCC |
| HOXD13 | CTTCGGCAACGGCTACTACAG | TGACACGTCCATGTACTTCTCC |
| HOXC8 | ACCGGCCTATTACGACTGC | TGCTGGTAGCCTGAGTTGGA |
| HOXA9 | TACGTGGACTCGTTCCTGCT | CGTCGCCTTGGACTGGAAG |
